# Schiff Bases and Stereocontrolled Formation of Fused 1,3-Oxazolidines from 1-Amino-2-Indanol: A Systematic Study on Structure and Mechanism

**DOI:** 10.3390/molecules28041670

**Published:** 2023-02-09

**Authors:** Esther Matamoros, Mark E. Light, Pedro Cintas, Juan C. Palacios

**Affiliations:** 1 Department of Organic and Inorganic Chemistry, Faculty of Sciences and IACYS-Green Chemistry and Sustainable Development Unit, University of Extremadura, 06006 Badajoz, Spain; 2 Department of Chemistry, Faculty of Engineering and Physical Sciences, University of Southampton, Southampton SO17 1BJ, UK

**Keywords:** Schiff bases, oxazolidines, tautomeric equilibria, DFT calculations, stereochemistry, aminoindanols

## Abstract

This paper thoroughly explores the formation of Schiff bases derived from salicylaldehydes and a conformationally restricted amino alcohol (1-amino-2-indanol), as well as the generation of 1,3-oxazolidines, a key heterocyclic core present in numerous bioactive compounds. We provide enough evidences, both experimental-including crystallographic analyses and DFT-based calculations on imine/enamine tautomerism in the solid state and solution. In the course of imine formation, a pentacyclic oxazolidine–oxazine structure could be isolated with complete stereocontrol, whose configuration has been determined by merging theory and experiment. Mechanistic studies reveal that, although oxazolidines can be obtained under kinetic conditions, the prevalence of imines obeys to thermodynamic control as they are the most stable structures. The stereochemical outcome of imine cyclization under acylating conditions leads to formation of 2,4-*trans*-oxazolidines.

## 1. Introduction

**Scheme 1:**
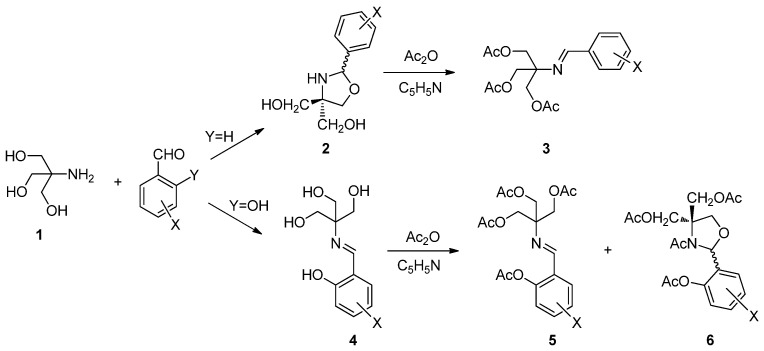
Ring-closure and ring-opening reactions under acetylating conditions.

For more than a decade, we have extensively investigated the chemistry of Schiff bases derived from aminopolyols and arylaldehydes, and disclosing a series of stereoelectronic effects that correlate with structure. It is particularly relevant to the interconversion between acyclic and heterocyclic frameworks with varied stereocontrol. Thus, the condensation of tris(hydroxymethyl) methylamine (TRIS, **1**) with substituted benzaldehydes gives rise to 1,3-oxazolidines (**2**), which afford the corresponding per-*O*-acetylated imines (**3**) upon conventional acetylation conditions (Scheme 1) [1,2]. However, the reaction of TRIS with salicylaldehydes produces Schiff bases (**4**), which lead to a mixture of peracetylated imines (**5**) and *N*-acyl-1,3-oxazolidines (**6**) after acetylation (Scheme 1).

**Figure 1:**
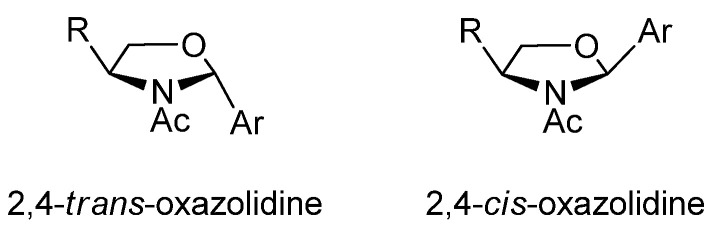
2,4-Oxazolidine diastereomers.

The above results are evidence that product selection is substrate-dependent and we were prone to investigate the stereochemical outcome under acylating conditions. In general, the formation of oxazolidines and related heterocycles proceed with high diastereoselectivity (often close to 100%). A key point lies in the configuration at the new chiral center formed (C-2), whose assignment in previous literature has been misleading. In fact, due to the reversibility of this transformation, the stereoselectivity can be controlled thermodynamically, thus results are strongly dependent on the experimental conditions. It has now been established that 2,4-*cis*-oxazolidine is the major diastereomer obtained through equilibrium conditions (Figure 1). Since *N*-alkyloxazolidines are the only stable products (NH-oxazolidines exist mainly as open imines), one can conclude that the most stable isomer with substituents at positions 2, 3 and 4 is the one showing 2,3-*trans* and 3,4-*trans* relationships.

**Figure 2:**
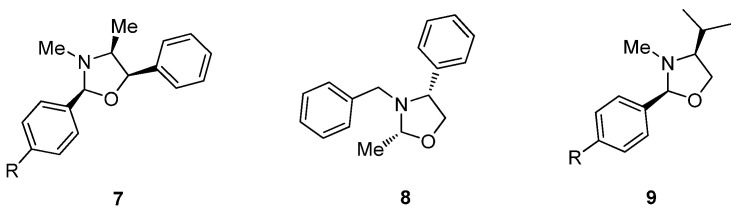
Previous examples of 2,4-*cis*-configured oxazolidines.

Surprisingly, the first X-ray analysis of a chiral oxazolidine, described as early as 1971, was controversial because the authors reported that the condensation of ephedrine and 4-bromobenzaldehyde in refluxing benzene gave rise to a 2,4-*trans*-oxazolidine as the sole product [3]. A subsequent synthetic and crystallographic revision proved this assignment to be wrong [4], and evidenced the opposite *cis* configuration. A plausible explanation for this discrepancy would be that the first authors crystallized the minor diastereoisomer present in the reaction mixture, enriched however in the other isomer. The relative 2,4-*cis* configuration has now been widely accepted and is usually observed under various experimental conditions, as exemplified by the condensation of aryl and alkyl aldehydes with ephedrine (**7**), *N*-methyl or *N*-benzyl-phenylglycinol (**8**) and *N*-methylvalinol (**9**) (Figure 2).

**Figure 3:**
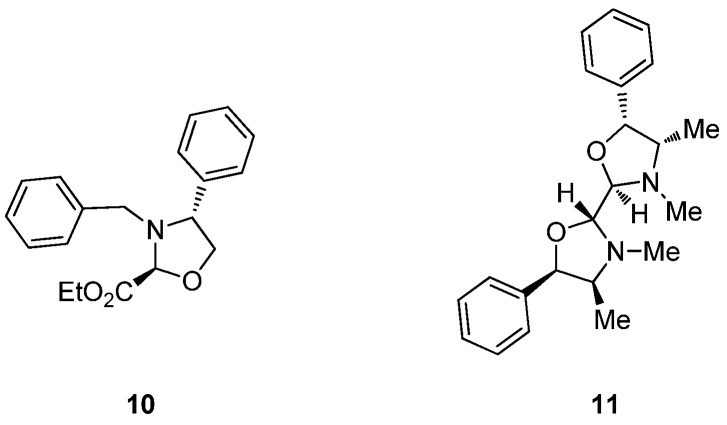
Reported examples of 2,4-*trans*-configured oxazolidines.

That said, some exceptions to the 2,4-*cis* configuration have been reported as well. Thus, the formation of the 2,4-*trans* isomer (**10**) has been observed in the reaction of methyl hemiacetal derived from ethyl glyoxylate with *N*-benzyl-phenylglycinol in CH_2_Cl_2_ at reflux and in the presence of molecular sieves [5]. In contrast, the *cis* configuration has been reported by other research groups in the condensation of glyoxylate with *N*-Boc-ephedrine under different conditions [6,7]. A *trans* configuration was also proposed for compound **11**, obtained by double condensation of ephedrine with glyoxal in THF-water [8] (Figure 3). These exceptions could be justified on the basis of both structural and experimental factors. In a comprehensive study, Agami and Rizk showed that condensation with aromatic aldehydes bearing electron-withdrawing groups led to *trans*-oxazolidines as kinetic products, and the rate of isomerization to the more stable *cis* isomer is largely influenced by the solvent system [9].

**Scheme 2:**
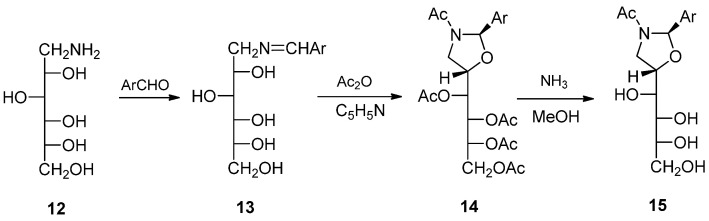
Chiral *trans*-configured oxazolidines obtained from d-glucamine and aryl aldehydes.

On the other hand, little data are available on the relative stereochemistry between the substituents at C-2 and C-5 after ring closure. To our knowledge, we described the only antecedent on the synthesis of chiral oxazolidines (**14**, **15**) from 1-amino-1-deoxyhexitols with aromatic aldehydes [10]. Condensation with d-glucamine (1-amino-1-deoxy-d-glucitol, **12**) leads initially to the corresponding imines **13**, which under acylating conditions produce chiral *N*-acyl oxazolidines **14** (Scheme 2). In this case, the stereochemistry with which the formation of oxazolidine takes place is 2,5-*trans*. The same is true for oxazolidines derived from *N*-methyl-d-glucamine [11]. 

**Scheme 3:**
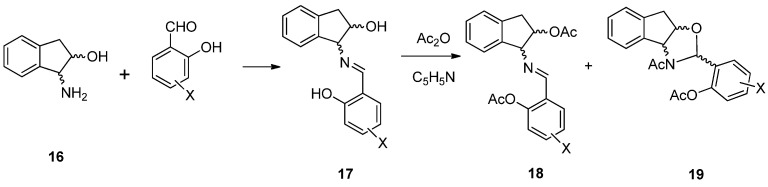
General synthesis of imines and oxazolidines derived from 1-amino-2-indanol.

In all the above cases, the aminopolyols employed were conformationally flexible substances. We reasoned a conformationally restricted partner might provide a different bias in the steric outcome and, to this end, 1-amino-2-indanol (**16**) was chosen. In principle, Scheme 3 provides a general strategy to the target compounds in line with the aforementioned results, i.e., Schiff bases **17** should be obtained by reaction of **16** with substituted salicylaldehydes. Subsequent acetylation would possibly lead to a mixture of the acetylated imine **18** and/or the corresponding oxazolidine **19**. As we shall see, however, through this detailed analysis, the transformation and products are endowed with tautomeric and configurational complexity, whose elucidation in both solution and solid state required a combination of spectroscopic and computational methods, which ultimately unveiled a marked stereoselection supplied by the rigid framework.

## 2. Results and Discussion

### 2.1. Imine Synthesis

**Figure 4:**
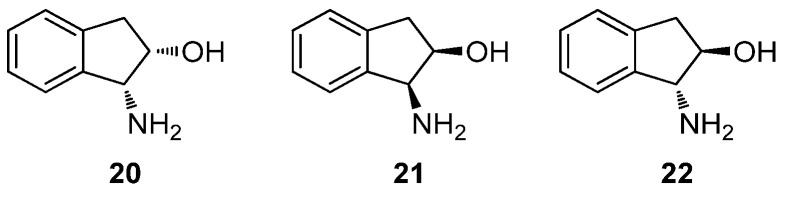
1-Amino 2-indanols used in the synthesis of Schiff bases.

Because compound **16** has two stereogenic centers, this chiral aminopolyol can exist in different stereoisomeric structures. Accordingly, the synthesis of the Schiff bases was carried out with (1*R*,2*S*)-1-amino-2-indanol (**20**), (1*S*,2*R*)-1-amino-2-indanol (**21**) and (1*R*,2*R*)-1-amino-2-indanol (**22**) (Figure 4). The choice is based on the relative disposition of the amino and hydroxyl groups, which lie in a *cis* arrangement in the first two and therefore they are spatially close; conversely, the *trans* disposition in **22** keeps them away. These configurational variations allow us to evaluate the influence of such spatial arrangements on the stereochemistry of ring-closing reactions. 

**Figure 5:**
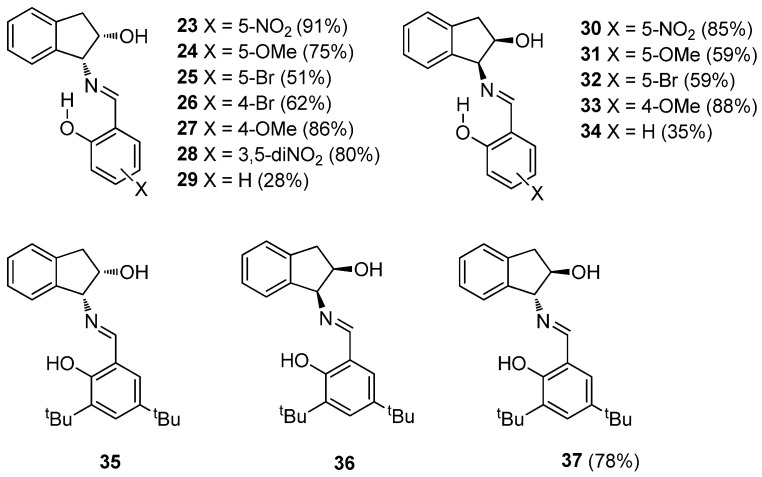
Synthesized Schiff bases.

All reactions were conducted by mixing equimolar amounts of the aminoalcohol and the corresponding substituted salicylaldehyde in absolute ethanol. The mixture was stirred at room temperature until the appearance of a solid, which can easily be isolated by filtration and usually purified by recrystallization, such as in the case of adducts **23**–**34**. To isolate salicylimines **29** and **34**, the reaction had to be carried out in ethyl ether because the use of ethanol as solvent resulted in the formation of an unexpected side product, whose structure will be discussed later. Moreover, the *trans*-configured imine **37** could be synthesized from the corresponding substituted salicylaldehyde, whereas enantiomerically pure *cis* imines **35** and **36** are commercially available. Yields ranged from moderate to good, although they were not optimized (see Experimental and Supplementary Information, Table S1) (Figure 5).

### 2.2. Solid-State Structures

The structures assigned to the above-mentioned Schiff bases are supported by their elemental microanalyses and spectroscopic data. The main problem associated with an accurate structural elucidation of Schiff bases stems from tautomerism because these substances can exhibit imine or enamine structure, both in solid state and in solution, which are not necessarily identical. 

**Figure 6:**
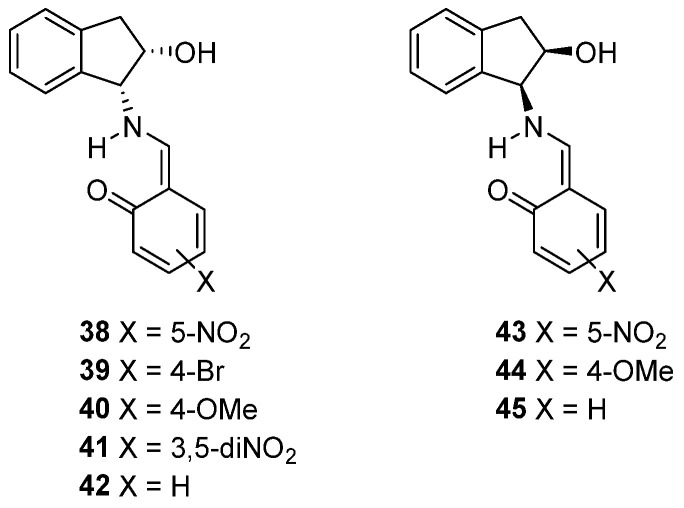
Synthesized Schiff bases with an enamine structure.

Solid-state structures can be inferred from FT-IR and Raman spectroscopies and a conclusive statement of these vibrational spectra is that the actual solid-state structures for compounds **23**, **26**–**30**, **33** and **34** correspond to enamine tautomers **38**–**45**, respectively (see Supplementary Material) (Figure 6).

**Figure 7:**
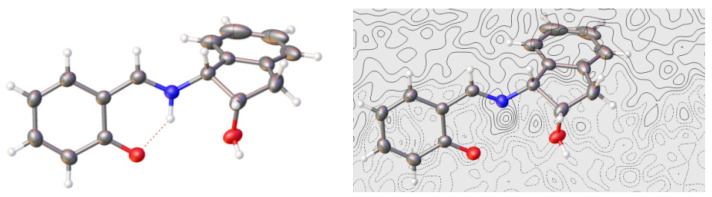
X-Ray diffraction pattern and electron density difference map for compound **42** (Color code: carbon, black; oxygen, red; nitrogen, blue; hydrogen, white).

Gratifyingly, crystals suitable for X-ray diffraction could be obtained by slow evaporation for compounds **24** and **40**–**42**, thereby determining with confidence their crystal structures. Crystallographic analyses evidenced an imine structure for **24**, while enamine tautomers were found for **40**–**42**. The exact position of the amine/enamine hydrogen atom was determined from the corresponding Fourier maps of electron density differences for the four refinements without including the hydrogen in the model. The simplest Schiff base, **42**, derives from unsubstituted salicylaldehyde (X = H), whose electron density difference map shows that the proton is located on the nitrogen atom (Figure 7).

**Figure 8:**
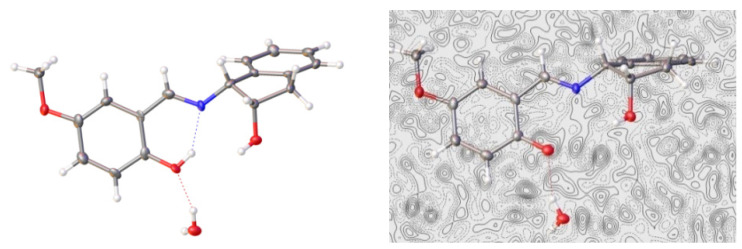
X-Ray diffraction pattern and electron density difference map of **24** (Color code: carbon, black; oxygen, red; nitrogen, blue; hydrogen, white).

For compound **24**, the structural elucidation by single-crystal diffraction showed the presence of one water molecule in the crystal lattice (Figure 8). 

**Figure 9:**
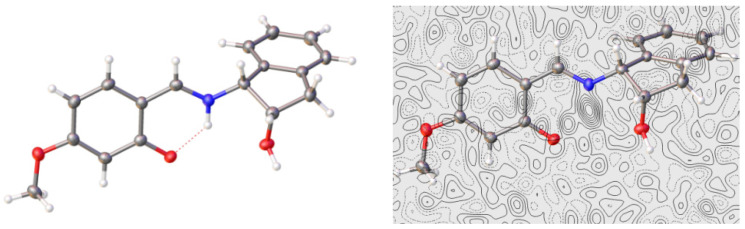
X-Ray diffraction pattern and electron density difference map of **40** (Color code: carbon, black; oxygen, red; nitrogen, blue; hydrogen, white).

The imine structure obtained for **24** and enamine for **40** (Figure 9) matched with the differences observed in their IR spectra, as noted above (Figures S17 and S24). It is noteworthy that the only difference between compounds **24** and **40** is the position occupied by the methoxy group at the aromatic ring, a key structural element favoring the formation of either imine or enamino tautomers (*vide infra*).

**Figure 10:**
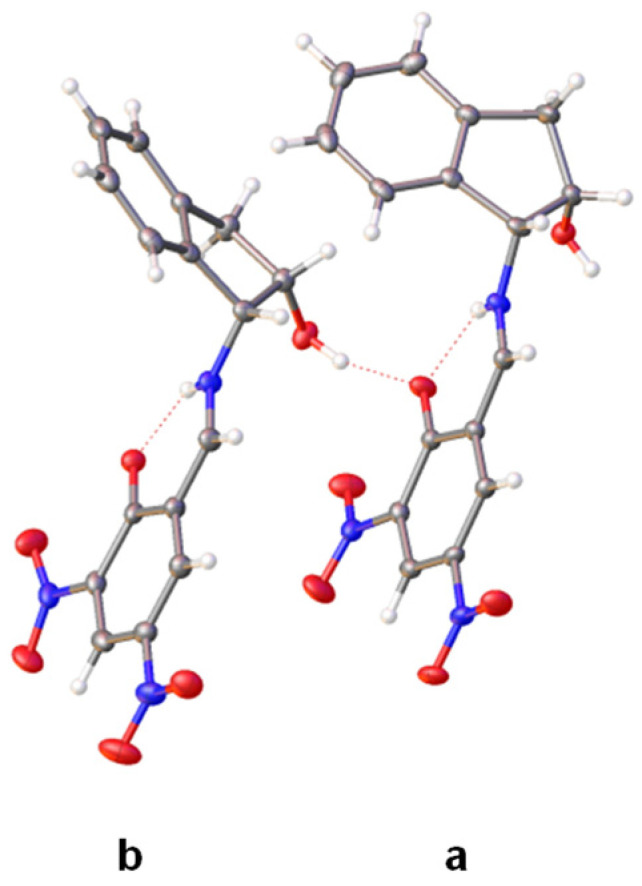
X-Ray diffraction structure of **41** having two enamine tautomers (molecules **a** and **b**) in the unit cell (Color code: carbon, black; oxygen, red; nitrogen, blue; hydrogen, white).

In both cases, the methoxy group is essentially coplanar with the ring to which it is attached, as shown by dihedral angles: −0.21° in **24** and 0.08° in **40**. In this arrangement, the delocalization provided by a lone pair from the oxygen atom is maximal. However, even though the Fourier map of electron density differences for **40** shows the proton located mainly on the nitrogen atom, a residual electron density is observed near the oxygen atom, indicating that the proton in question is delocalized between the two positions—that is, it is partially bound to both atoms: oxygen and nitrogen (Figure 9). This suggests the coexistence of imine and enamine tautomers in rapid equilibrium within the crystal lattice. For dinitro derivative **41**, X-ray diffraction analysis shows the presence of two molecules in the unit cell and both molecules have an enamine structure (Figure 10). 

**Figure 11:**
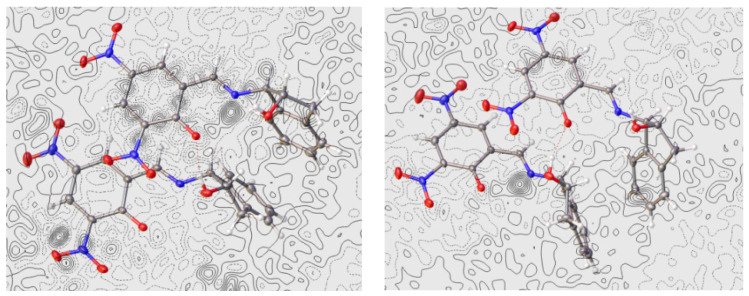
Electronic density difference maps of the two molecules of **41** (see main text and Figure 12) (Color code: carbon, black; oxygen, red; nitrogen, blue; hydrogen, white).

**Figure 12:**
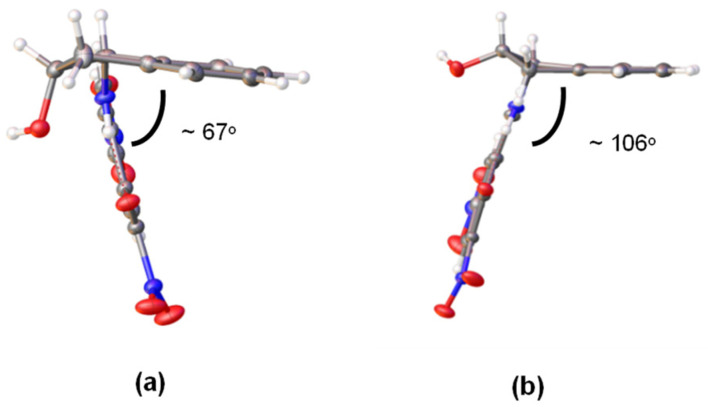
(**a**) Conformations and (**b**) relative dispositions of the aromatic rings of **41** in crystalline state (Color code: carbon, black; oxygen, red; nitrogen, blue; hydrogen, white).

The Fourier difference map places the proton in each molecule primarily on the nitrogen atom, thus evidencing an enamine structure (Figure 11). Like compound **40**, however, a residual electronic density is located near the oxygen atom as well. These two molecules differ only by the conformation adopted by the pentagonal ring of indanol moieties. Thus, in one of them, the ring shows a geometry close to an *E*_2_ conformation and the angle formed by the two benzene residues is ~67° (Figure 12a). The other molecule has a ^2^*E* conformation and the angle between the benzene rings is considerably greater (~106°) (Figure 12b). The nitro groups at C-5 are practically coplanar with the 1,3-cyclohexadiene ring, showing small dihedral angles of 1.62° in the first molecule (**41a**) and 2.85° in **41b**. In contrast, due to steric effects, the nitro groups at C-3 deviate from the planarity forming angles of 9.66° and −12.60°, respectively.

**Figure 13:**
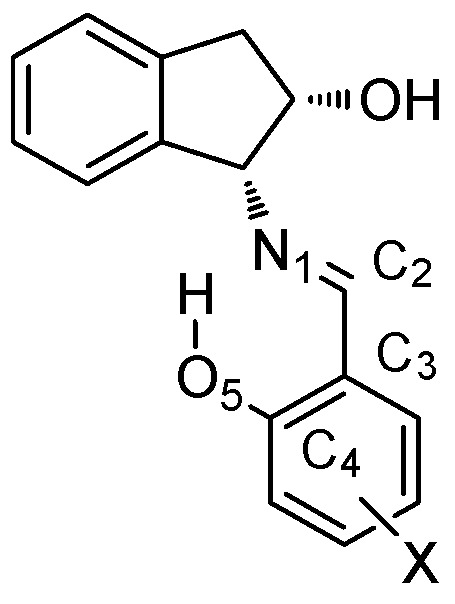
Atom numbering employed for the tautomeric system.

All structures elucidated by single-crystal diffraction exhibit a strong intramolecular hydrogen bond leading to a six-membered ring whose geometric characteristics are collected in Table S2. Table 1 compares some bonding distances obtained by X-ray analyses of **24** and **40**–**41**, with the average values of similar structures described as phenoliminic tautomers [12,13,14,15,16,17,18,19,20,21,22,23,24,25,26,27,28,29,30,31] and ketoamines [32,33,34] in the Cambridge Structural Database (Figure 13). The analysis of bond distances in compounds **40** and **42** indicates that the values of d_C2-C3_ and d_C4-O5_ are in full agreement with an enamine structure. The value of d_N1-C2_ for compound **40** is consistent with an enamine structure, while the corresponding value in **42** lies between the extreme values expected for imine and enamine tautomers.

**Scheme 4:**
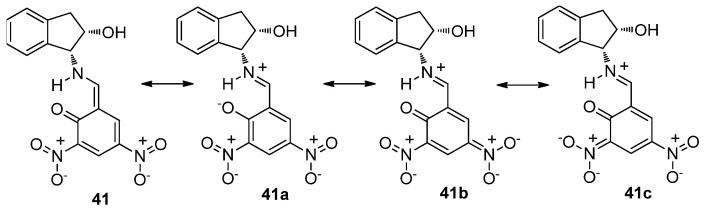
Resonance structures (neutral and zwitterionic) for compound **41**.

The d_C2-C3_ and d_C4-O5_ distances measured for **24** are between the limiting values of imine and enamine structures, although d_N1-C2_ clearly agrees with a ketoamine form. It is evident that this ambivalence encountered in the bond distances of **24** shows the existence of a very fast interconversion equilibrium between both structures, even at 100 K, and the conditions employed for recording X-ray diffraction spectra. The coexistence of two molecules in the crystal lattice of **41** is singular. The distances d_N1-C2_ and d_C2-C3_ clearly correspond to an iminic structure; however, the short distance d_C4-O5_ corresponds to a C=O bond. These results do not agree with a neutral enaminic structure such as **41** (Scheme 4), but do agree with a zwitterionic structure such as **41a**–**41c**.

The resonance form **41b** should greatly contribute to a global description of this compound, because the nitro group at C-3 is not coplanar with the aromatic ring. Therefore, the delocalization of the negative charge on this group in structure **41c** must be less important than in **41b**. In the latter, the bonds in which the oxygen and nitrogen atoms are involved show olefinic character, N1=C2 and C4=O5; accordingly, the distances measured by X-ray diffraction are significantly short (1285 Å and 1289 Å for N=C, 1259 Å and 1263 Å for C=O). It should be mentioned that solid-state ^13^C and ^15^N NMR spectra have also been used to assign imine/enamine structures, in view of a few diagnostic chemical shifts of such nuclei [35,36,37,38,39,40].

### 2.3. Structures in Solution

Imine and enamine structures can be assessed in solution through NMR analyses. Thus, for an imine structure, we would observe a singlet signal for the phenolic OH, whereas in an enamine form, the NH proton would be coupled with the protons of neighboring CHs bonds. Nonetheless, in view of a rapid dynamic equilibrium in solution, the recorded signals will average over time, resulting in resonances attributable to both structures. Therefore, the value measured for coupling constants could vary between 0 Hz and a maximum of ~14 Hz. However, the chemical shift of the aromatic carbon linked to the phenolic group is a more reliable indicator of the extent of such an equilibrium. In general, these resonances lie between the limiting chemical shifts of a phenolic carbon for imines and the carbonyl group of enamines (~155 ppm and ~180 ppm, respectively) [36,41,42,43,44,45,].

Table 2 shows the characteristic proton and carbon resonances for compounds **23**–**34** and **37**, for which either imine or enamine structures can be attributed to the prevalent tautomers. Since all spectra are similar, only two-dimensional COSY and HMQC correlations allow us to assign some diagnostic peaks with confidence (2D-NMR spectra for **27** are displayed in Figures S5 and S6).

Despite half of the compounds having enamine structures in the solid state, ^1^H NMR spectra in DMSO-*d*_6_ at room temperature evidenced the prevalence of imino tautomers because the HC=N proton remains decoupled. Compound **41** represents a notable exception because, even if the NH proton appears as a broad singlet, the ethylene signal is a doublet with a high coupling constant (^3^*J* = 14 Hz) (Figure S7). However, if we look at ^13^C NMR data, compounds **38** and **43** are compatible with an enamine structure because they exhibit carbon resonances at ~178 ppm, which correspond unequivocally to enamine carbonyls, even though their ethylene protons lack coupling constants.

Nevertheless, a clear-cut structural assignment is not an easy task. As mentioned, the tautomeric equilibrium dictates that the observed chemical shifts (*δ_exp_*) do actually correspond to average contributions of the imine (*δ_i_*) and enamine (*δ_e_*) forms (Equation (1)).

(1) $$\delta_{exp}=n_{i}\delta_{i}+n_{e}\delta_{e}$$

where *n_i_* and *n_e_* are the populations of molecules with imine and enamine structures, respectively (*n_i_* + *n_e_* = 1). The same applies to the coupling constant analysis, which can be represented by Equation (2) for the tautomeric equilibrium [1].

(2) $$J_{exp}=n_{i}J_{i}+n_{e}J_{e}$$

However, the presence of acid or basic substances capable of promoting a fast proton exchange can result in partial or totally decoupled spectra. This could account for the behavior of compound **38**, which appears to have an imine structure according to its proton spectrum, while carbon resonances point to an enamino tautomer.

### 2.4. Theoretical Calculations on Imine–Enamine Stability

**Scheme 5:**
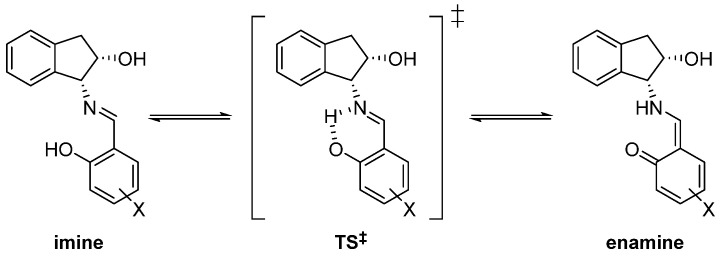
Tautomeric imine–enamine equilibrium trough a cyclic transition state (‡).

In order to gain further insight into the ease with which the imine–enamine transformation takes place, DFT calculations [46,47,48,49,50,51,52,53] were performed at the M06-2X/6-311G (d,p) level of theory [54,55,56], as implemented in the Gaussian package [57], and modeling solvation effects with the SMD method [58]. Compounds **24**/**31**, **29**/**34**, **38**/**43**, **40**/**44** and **41** were used as models because **38**, **40** and **41** have an enamine structure and, conversely, **24** and **29** display well-defined imine structures (Scheme 5). Table 3 and Table 4 collect the relative energies for imine and enamine structures, together with the corresponding transition structures (TS^‡^) characterized by imaginary frequencies, calculated in the gas phase and in DMSO (ε = 46.8).

Results found in the gas phase indicate that the most stable form is the imine structure. This contrasts with the existence of stable enamino tautomers in the solid state. Because no intermolecular interactions are present in the gas phase, the calculated relative stability expresses the intrinsic stability of each species. Therefore, the inversion of stability encountered in the solid phase reasonably has its origin in packing interactions that stabilize the crystal arrangement. However, results in DMSO (Table 4) agree with those observed experimentally, where compounds **38** and **41** have an enamine structure. That is, the interaction with solvent molecules can also cause the relative imine–enamine stabilities to be reversed.

**Figure 14:**
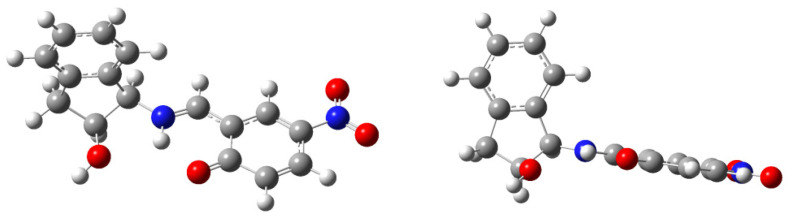
Computed enamine structure **38**, showing coplanarity of aryl and nitro groups.

In addition, calculations suggest that electron-donating groups favor an imine structure, whereas enamines form in the presence of electron-withdrawing substituents. In compound **38**, the nitro group at C-5 increases the acidity of the hydroxyl and concomitantly decreases the basicity of the carbonyl oxygen, thus favoring an enamine structure (*δ*_C2_ = 178.1 ppm). Figure 14 shows how the intense delocalization of the nitro group makes it coplanar with the hexadienic ring.

**Figure 15:**
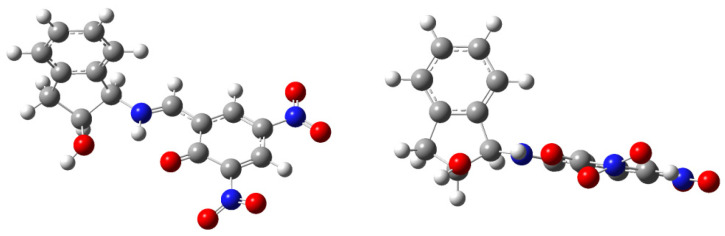
Computed structure of compound **41** with different arrangements of nitro groups.

It could be expected that the presence of two nitro groups would reinforce the aforementioned effect and, as a result, compound **41** should have an enamine structure characterized by *δ*_C2_ similar to that of **38** or even higher. Surprisingly, this is not the case (δ_C2_ = 170.1 ppm), the reason being that a steric hindrance inhibits delocalization. Thus, the electronic effect of the second nitro group is significantly attenuated by the adjacent carbonyl group, which forces the former to adopt a non-coplanar disposition with the ring (Figure 15).

The interconversion barriers between the two tautomeric forms are always lower than 6.3 kcal·mol^−1^ (<4.7 kcal·mol^−1^ in DMSO), low enough for these transformations to proceed fast at room temperature. This ease of interconversion explains the landscapes in the Fourier maps of electron density differences, in which electron densities at the hydrogen atom are shared between the oxygen and nitrogen atoms. In other words, there must be a rapid equilibrium within the crystal lattice between imine and enamine tautomers.

In the gas phase, the energies of activation in the endothermic direction of the interconversion—that is, when going from enamine to imine—are generally negative (−0.4 ≤ ΔG^‡^ = ΔG_TS_^‡^ − ΔG_enamine_ < 0.0 kcal·mol^−1^). The saddle points show an almost identical energy to the enaminic tautomers, and therefore the structure of the transition state is essentially coincidental with the ground structure. This curious—but not unusual—behavior has been described for imines derived from salicylaldehydes [59], acetophenones [60], aminoacroleins [61], vinamidines [62], gossypol [63] and a naturally-occurring aldehyde. This variational effect is associated with low activation barriers and high imaginary vibration frequencies (~1000 cm^−1^), as discussed previously in detail [59,60].

### 2.5. Electronic Effect of the Substituents on Imine–Enamine Tautomeric Equilibria

The tautomerization constant of the imine–enamine balance in solution [59], defined as *K*_T_ = [enamine]/[imine] = *n_e_*/*n_i_*, is determined by:

*K*_T_ = *n_e_*/*n_i_* = (*δ_exp_* − *δ_i_*)/(*δ_e_* − *δ_exp_*) (3)

Constants can be calculated by choosing appropriate *δ_i_*/*δ_e_* values that represent “pure” imine or enamine structures.

**Scheme 6:**
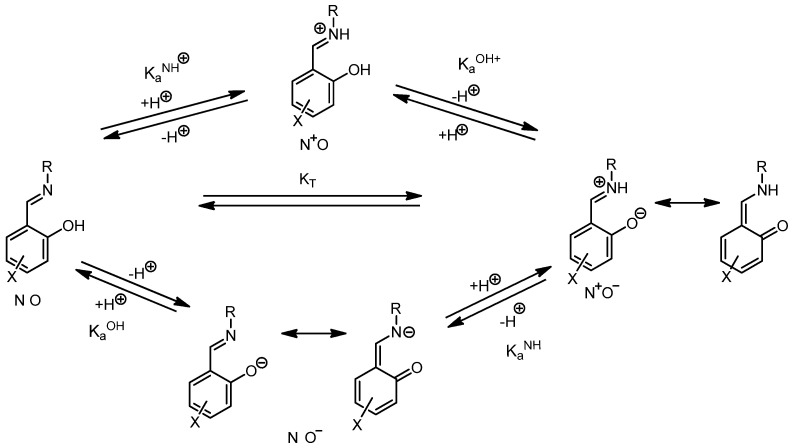
General view of phenolimine–ketoamine tautomeric equilibria.

Scheme 6 shows the equilibrium landscape related to the phenolimine–ketoamine tautomerism in solution. To study the influence exerted by the substituents of the salicylaldehyde core on the tautomeric equilibrium, we have applied the formalism developed for salicylimines [59] to the present case. 

The analysis of substituent effects on these equilibria leads to a modified Hammett Equation (4):

log *K*_T_ = ρ(σ_x_^OH^ − σ_x_^NH^) + c = ρσ_ef_ + c (4)

where σ_x_^OH^ = σ_para_^x^ if the substituent is placed at C-5 and σ_x_^OH^ = σ_meta_^x^ if it is at C-4; similarly, σ_x_^NH^ = σ_para_^x^ when the substituent is located at C-4 and σ_x_^NH^ = σ_meta_^x^ when it is at C-5. For polysubstitution, the additive Equation (5) can be used:

log *K*_T_ = ρΣσ_ef_ + c (5)

The quantitative analysis was performed on imines **23**–**29** using the spectroscopic data collected in Table 2 and Table S3. By inserting the values *δ_e_* = 178.06 ppm from **23** and *δ_i_* = 154.62 ppm from **24** into Equation (3), we obtained Equation (6), whose graphical representation is shown in Figure 13a. Despite the limited number of compounds used, a very good fit could be obtained (*r* = 0.9690) thanks to the large range of σ_ef_ values (Δσ_ef_ ~0.65).

*K*_T_ = *n_e_*/*n_i_* = (*δ_exp_* − 154.62)/(178.06 − *δ_exp_*) (6)

**Figure 16:**
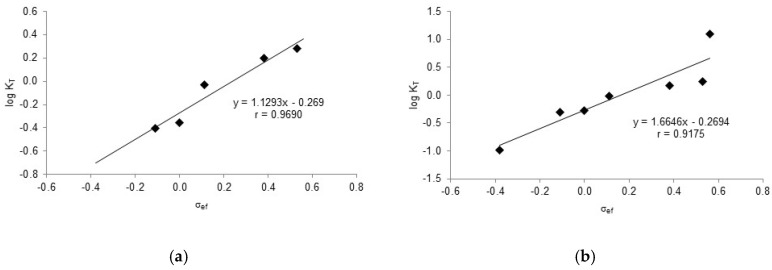
Linear regression analyses for tautomeric constants of substituted imines/enamines versus ^13^C NMR chemical shifts. (**a**) δ_e_ = 178.06 ppm, δ_i_ = 154.62 ppm. (**b**) δ_e_ = 180.12 ppm, δ_i_ = 152.00 ppm.

To obtain a more general linear relationship for tautomeric equilibria, the lowest (152.00 ppm) [64] and the highest (180.18 ppm) [59] chemical shifts found in imines/enamines derived from salicylaldehydes were taken into account, leading to Equation (7). A plot of the latter results in Figure 16b exhibiting a good fit (*r* = 0.9175), yet it is slightly less accurate than that of Figure 16a.

*K*_T_ = *n_e_*/*n_i_* = (*δ_exp_* − 152.00)/(180.18 − *δ_exp_*) (7)

Similarly, a good correlation could be obtained by plotting the chemical shift of the corresponding phenolic carbon (C2) versus δ_ef_, as summarized by Equation (8) and displayed in Figure S1.

δ_C2_ = 20.384 σ_ef_ + 162.62 (*r* = 0.9506) (8)

However, the linear relationships involving the imine carbon against σ_OH_ and σ_NH_, Equations (9) and (10) respectively, resulted in poorer correlations (Figure S2a,b). In contrast, a plot of log *K*_T_ against C-2 chemical shifts produces a pretty good correlation (Equation (11), Figure S3); likewise, an accurate fit arises from correlating the chemical shifts of imine carbon and imine hydrogen (r = 0.9482, Figure S4), which evidence similar responses of such atoms to the electronic effects of substituents.

δ_CN_ = 1.1595.σ_OH_ + 165.04 (*r* = 0.8216) (9)

δ_CN_ = 1.5391.σ_NH_ + 165.05 (*r* = 0.8324) (10)

log *K*_T_ = 0.084.δ_C2_ + 165.05 (*r* = 0.9931) (11)

### 2.6. Reaction of 1-Amino-2-Indanol with Salicylaldehyde

**Scheme 7:**
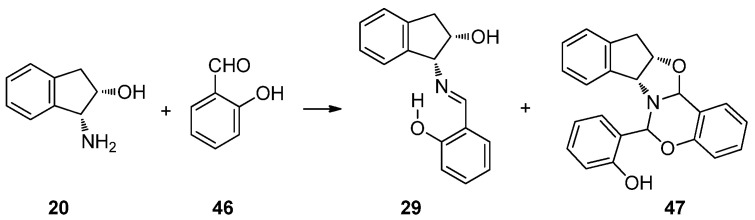
Reaction of (1*R*,2*S*)-1-amino-2-indanol with salicylaldehyde.

As introduced earlier (Section 2.1), the reaction of (1*R*,2*S*)-1-amino-2-indanol (**20**) with salicylaldehyde (**46**) in ethanol afforded not only the corresponding imine (**29**), but also an unknown product detected by thin-layer chromatography. Because the reaction mixture was reluctant to crystallize, it was brought to dryness after one week, giving rise to an oil that crystallized from ethyl ether. The latter was a mixture of white and yellow crystals whose chromatographic analysis (ethyl acetate:petroleum ether 1:5 v/v) showed two spots with R_f_ = 0.25 and 0.70, which clearly indicated the formation of two structurally different substances. Fortunately, their separation could be achieved after successive recrystallizations. The yellow solid had the lowest R_f_ value and corresponds to imine **29**, while white crystals belonged to the fastest moving spot, further elucidated as **47** and generated by condensation of two salicylaldehyde molecules and one indanol moiety (Scheme 7).

**Figure 17:**
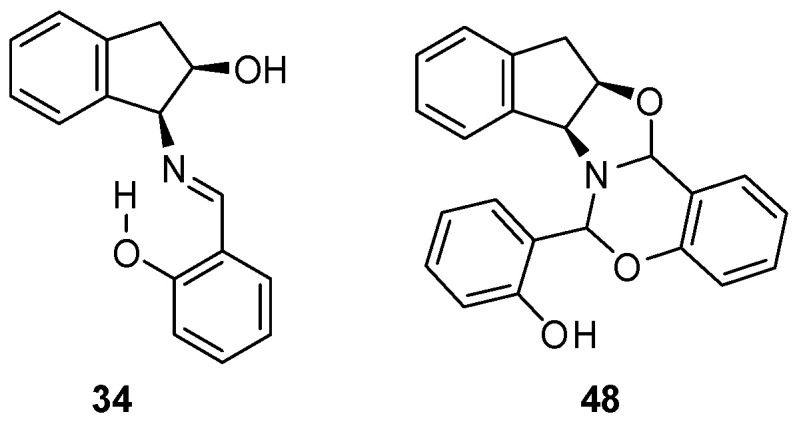
Structures of **34** and **48**, the enantiomers of **29** and **47**, respectively.

Analogously, starting from (1*S*,2*R*)-1-amino-2-indanol, the enantiomeric derivatives **34** and **48** could be isolated (Figure 17). Both analytical and spectroscopic data support the structures assigned to such compounds. In the FT-IR spectra, the main difference is that imine **29** presents an absorption at 1634 cm^−1^, attributable to the C=N double bond, while for **47**, there are no absorptions in the range of 2000–1618 cm^−1^, thus evidencing the absence of either C=N or C=O bonds (Figures S26 and S31).

Figures S10 and S11 collect ^1^H NMR and 2D-COSY spectra of **47**. In the former, the most deshielded signal at 9.91 ppm should be assigned to the only phenolic proton, which can be exchanged with D_2_O. In the range of 8.0–6.5 ppm, a total of twelve protons are consistent with three aromatic rings. The remaining four protons of the non-aromatic residue of indanol are upfield shifted. An interesting signal appears as a singlet at 5.75 ppm, which, together with the absence of any peak attributable to the imine group (HC=N), points to a saturated carbon. In addition, that chemical shift is similar to that of other oxazolidines described previously [1,2,65,66,67,68,69], which suggests the existence of this heterocycle in **47**. However, it is much more relevant a second singlet at 4.92 ppm, which could be consistent with an oxazine ring. Further structural information stems from the ^13^C NMR spectrum of **47**, which has signals corresponding to three aromatic rings between 157–116 ppm. The 2D-experiment provided by the HMQC spectrum correlates the proton signals at 5.75 and 4.92 ppm with those of two carbons at 86.72 and 81.12 ppm, which are typical of saturated carbons and lie in the range observed for the C-2 atom in oxazolidine and oxazine rings (Figures S12 and S13).

To further confirm that the structure of **47** involves a fragment of indanol and two from salicylaldehyde, its high-resolution mass spectrum (chemical ionization mode) was recorded. A peak at *m/z* 358.1457 was detected, which agrees with the empirical formula C_23_H_20_NO_3_ (calculated *m/z* 358.1443) corresponding to the [M+H]^+^ ion of **47**.

**Figure 18:**
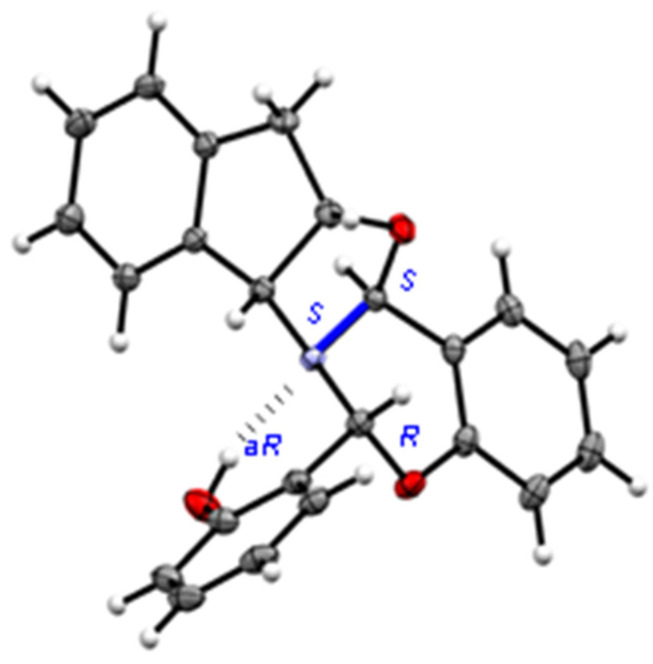
X-ray diffraction of **47** showing the configuration of chiral centers.

All the above data allowed us to propose a condensed polycyclic structure for **47**, in which an oxazolidine plus a six-membered oxazine ring would have been formed, each containing a new chiral carbon. The fusion of rings generates a rigid structure and, so long as the nitrogen atom is pyramidal, its inversion is impeded and behaves as another stereogenic center. However, spectroscopic data alone are insufficient to determine the stereochemistry of the three new chiral centers, affording potentially up to eight (2^3^) diastereoisomers. At last, X-ray diffractometry unambiguously confirmed the structure proposed for **47**, thereby making it possible to establish the absolute configurations (Figure 18). 

**Figure 19:**
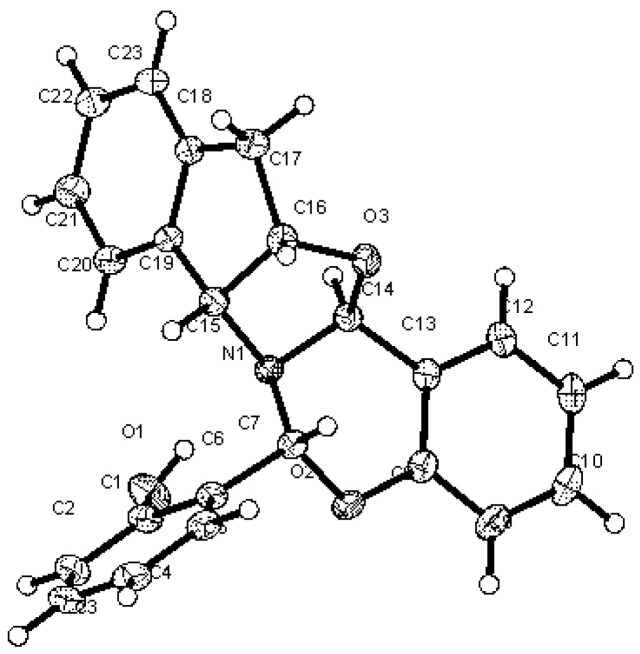
Crystallographic numbering for compound **47**.

It is worth noting that the fused rings show the following conformational arrangements:

(a) The cyclopentane ring exhibits an *E*_2_ conformation [70], i.e., the two benzene carbons and the two saturated carbons forming the cyclopentane ring are virtually coplanar because the dihedral angle Φ_C15,C19,C18,C17_ is close to zero (0.29°) (Table 5, Figure 19).
(b) The oxazolidine fragment shows a ^3^*T*_2_ conformation.
(c) The oxazine ring adopts an unusual conformation for a six-membered heterocycle because five atoms lie approximately in the same plane, which is reflected by the low values of the dihedral angles Φ_O2,C8,C13,C14_ (2.56°) and Φ_O2,C8,C13,C14_ (9.12°), whereas the carbon atom between the oxygen and nitrogen atoms lies above that plane.

The nitrogen atom shows a strong pyramidalization, which can be defined as the difference between the sum of the three bond angles sharing a common nitrogen atom (Σθ°) and a planar (360°) system. The calculated pyramidalization (360° − Σθ°) is ~35°, somewhat larger than that of an ideal tetrahedral system such as methane or ammonium ion: 31.5° (Table 6) [71]. As a consequence of pyramidalization and the lack of inversion, the nitrogen atom behaves in practice as a chiral center with absolute (*S*)-configuration.

The X-ray diffraction pattern of **47** shows a hydrogen bridge between the phenolic hydroxyl and the nitrogen atom. The geometrical data of this hydrogen bridge are gathered in Table S4. This intramolecular hydrogen bond anchors the conformation of the *orto*-hydroxyphenyl group, strongly restricting its rotational mobility and creating a new source of chirality: an atropisomeric axis of chirality *aR* [72,73].

### 2.7. On the Stability of Diastereomeric Combinations

**Figure 20:**
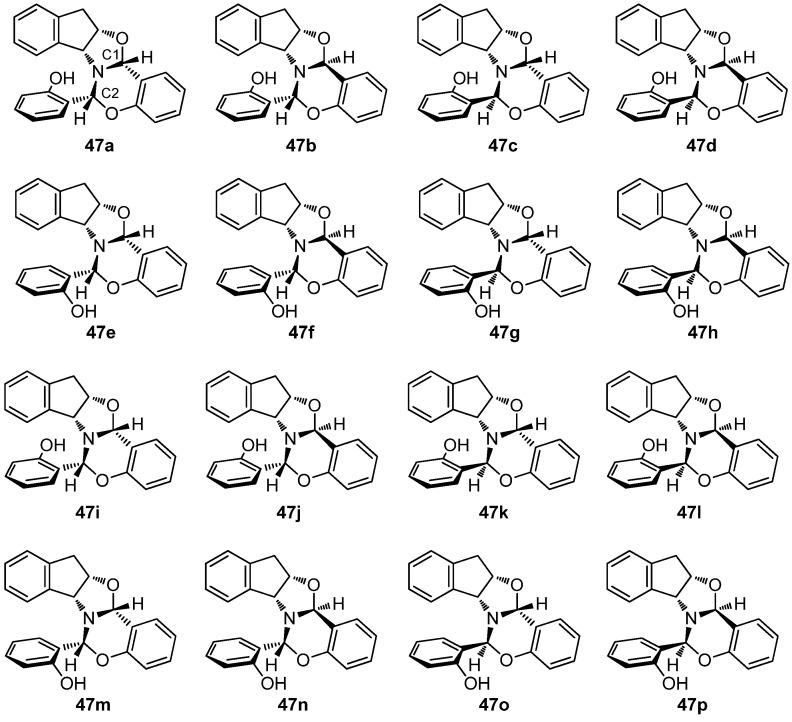
Diastereomeric combinations of compound **47** having multiple stereogenic elements.

The stereochemical complexity shown by **47** (or **48**) arises from the coexistence of four new stereogenic elements, leaving aside the pre-existing chirality of the indanol moiety, namely two new chiral carbons, a chiral pyramidal nitrogen, and a chiral axis due to the intramolecular hydrogen bond that largely restricts the conformational mobility. In other words, there can potentially be up to sixteen diastereoisomers (2^4^), whose discrimination is indeed challenging. Using theoretical calculations, we evaluated the relative stability of all the sixteen diastereoisomers (Figure 20), both in the gas phase and in ethanol, in an attempt to elucidate why one and only one diastereomer was actually isolated. Computational results are summarized in Table 7.

**Figure 21:**
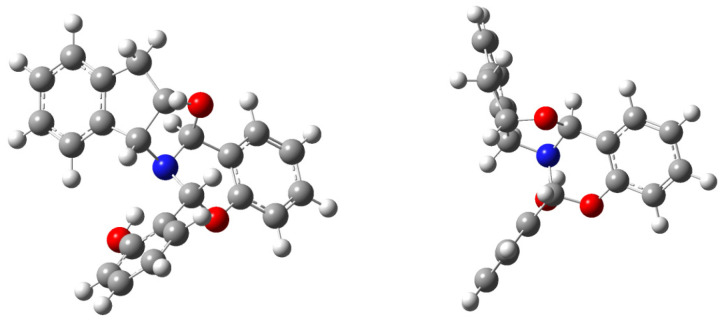
Calculated structure of **47b** (frontal and lateral views).

Moreover, from the viewpoint of structural diversity, all diastereoisomers of **47** can also form intramolecular hydrogen bonds with the nitrogen or the oxygen atom, whose geometries are collected in Tables S5 and S6. Of all possible stereoisomeric combinations, the most stable, both in the gas phase and in ethanol, is **47b**, which is in fact the experimentally observed diastereomer. The remaining diastereomers are less stable, up to ~15 kcal·mol^−1^. The calculated structure of **47b** (Figure 21) is coincidental with that determined by single-crystal X-ray diffraction analysis (Figure 18).

### 2.8. Hydrogen Bonding Energy

The chemical shift displacement of the hydroxyl proton can be estimated by Schaefer’s correlation [74] according to Equation (12), giving rise to a value of 6.02 kcal.mol^−1^ for the strength of the intramolecular bonding in **47b**.

*E_HB_* = −*δ_exp_* + 3.89 ± 0.2 = −9.91 + 3.89 ± 0.2 = −6.02 ± 0.2 kcal·mol^−1^ (12)

In contrast, the empirical relationship of Musin and Mariam [75], using the value of d_D···A_ (2.736 Å) as determined by X-ray diffraction, results in Equation (13):

*E*_HB_ (kcal·mol^−1^) = −5.554 × 10^5^ e^−4.12d^_D…A_ = −7.06 kcal·mol^−1^ (13)

**Figure 22:**
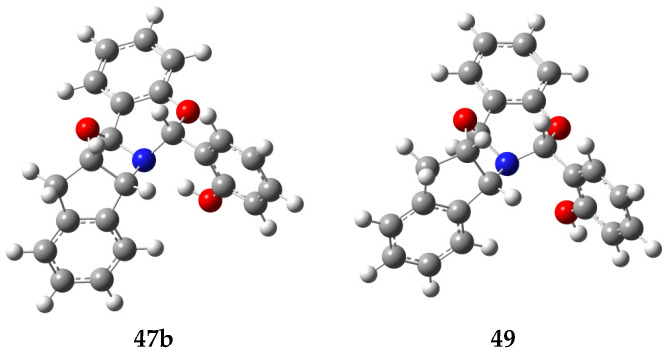
Computer-generated structures **47b** (H-bonded) and **49**.

The latter (7.06 kcal·mol^−1^) is slightly higher than the energies calculated in the gas phase and ethanol (6.37 and 6.14 kcal·mol^−1^, respectively). Furthermore, the stabilizing effect supplied by H-bonding manifests itself by computing the relative energies for **47b** with intramolecular hydrogen bond against **49**, which is lacking that interaction (Table 8, Figure 22). The latter is generated by 180°-rotation around the phenolic bond. Structure **47b** becomes more stable by ~9 kcal·mol^−1^ in the gas phase and by ~4 kcal·mol^−1^ when solvation is included. Although the intramolecular H-bond is not especially strong, it suffices to stabilize **47b** to a significant extent and reduce the conformational flexibility.

### 2.9. Reaction Mechanism: Experiment and Rationale

**Figure 23:**
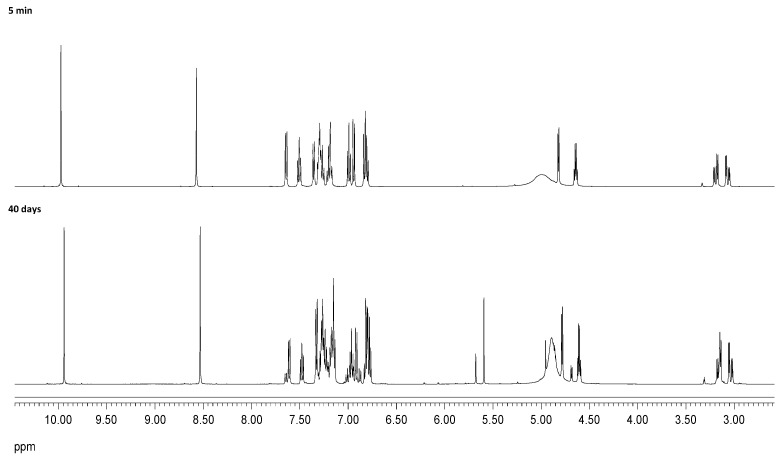
Temporal recording (^1^H NMR) for the reaction of **20** and salicylaldehyde in CD_3_OD.

**Figure 24:**
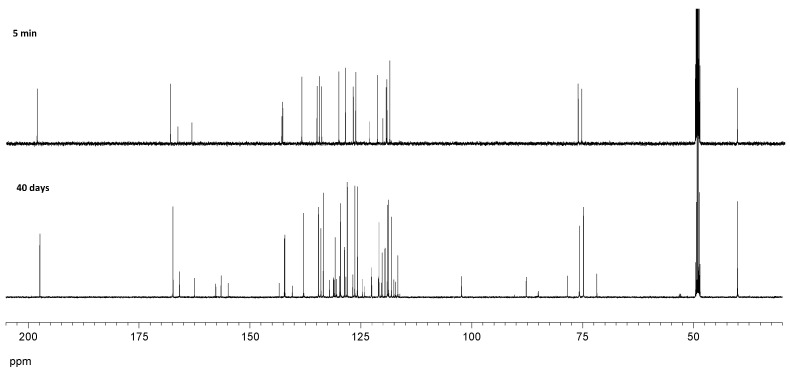
Temporal recording (^13^C NMR) for the reaction of **20** and salicylaldehyde in CD_3_OD.

In order to rationalize the formation and isolation of compound **47b**, a series of additional experiments and calculations were undertaken. (a) Because this substance contains two units of salicylaldehyde and one indanol fragment, the reaction was repeated in ethanol using a 1:2 indanol-aldehyde ratio and monitored by TLC. Formation of **29** could be observed almost immediately, while compound **47b** appeared after five days. (b) When the reaction was conducted in diethyl ether using the aforementioned 1:2 molar ratio, formation of **47b** was not detected at all after two weeks, and only imine **29** could be isolated by crystallization. (c) The same reaction was monitored by ^1^H NMR in MeOH-*d*_4_ over time, which evidenced the rapid formation of imine (**29**). After 24 h, two proton signals at ~5.7 and 4.9 ppm attributable to the chiral carbons of **47b** were observed, in a very low proportion nevertheless. After 40 days, the proportion of the latter increased significantly up to a 4:1 ratio between **29** and **47b**. Given the low solubility of **47b** in methanol, it slowly crystallized on standing. Figure 23 and Figure 24 show the ^1^H NMR and ^13^C NMR monitoring of the reaction mixture after 5 min and after 40 days at room temperature. The initial mixture only contained imine and salicylaldehyde mixtures, whereas signals corresponding to **47b** appeared progressively after 24 h. 

The singlet signal appearing at ~5.7 ppm correlates (HMQC spectrum) with the carbon signal resonating at 102.3 ppm. Chemical shifts similar to those encountered in oxazolidines suggested the intermediacy of this heterocycle. However, the signal is also accompanied by new peaks in the aromatic zone, which are consistent with the incorporation of salicylaldehyde. In fact, the addition of this substance to the imine in methanol resulted in the formation of **47b** after one week. Thus, it is plausible to assume that imine **29** is formed first and subsequent reaction with salicylaldehyde produces **47b**. As previously noted, this transformation is highly diastereoselective without observing other isomers. Attempts to synthesize analogs of **47b** by varying the substituents at the aromatic ring were unsuccessful.

**Scheme 8:**
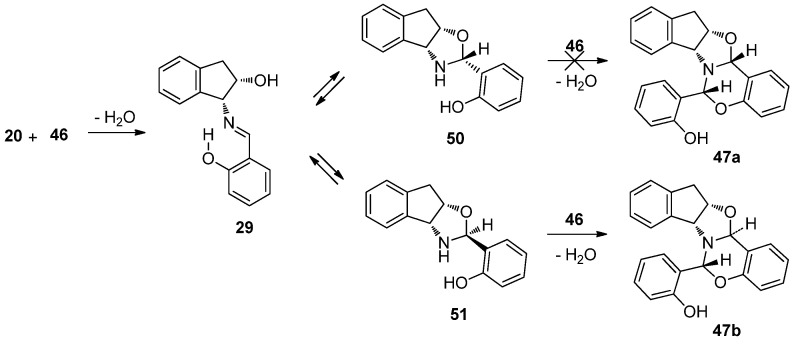
Competitive equilibria between imine and oxazolidines toward **47b**.

To shed light upon the ease with which the intermediate is formed with respect to the starting reagents, the relative stability of such candidates, i.e., imine **29** or diastereomeric oxazolidines (**50** and/or **51**), was evaluated as well (Scheme 8). 

**Figure 25:**
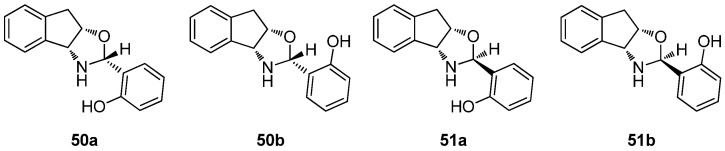
Structures studied theoretically (DFT method).

Four different structures should be taken into account depending on whether the chiral center formed is *R* (**50**) or *S* (**51**), and depending on whether the hydrogen bond involving the aromatic OH is oriented towards the nitrogen (**a**) or the oxygen atoms of the oxazine ring (**b**) (Figure 25).

Table 9 shows the calculation of relative energies, which favor formation of imine in both the gas phase and ethanol (exothermic pathway). In addition, the imine structure is less stable than the corresponding oxazolidines; however, experiments verified the formation of imine **29** and adduct **47b**. The enhanced stability of **50a** and **51a** could be justified by the intramolecular H-bond with the nitrogen atom (geometrical parameters are tabulated in Table S7).

Finally, the stability of structure **47b** has been calculated with respect to the corresponding reaction intermediates, which are imine **29** and oxazolidines **50** and **51**. Although in the gas phase **47b** is not the most stable structure, it becomes more stable than the intermediate in ethanol. Formation of **47b** is presumably driven by a combination of both its thermodynamic stability and its insolubility in the reaction mixture, thereby leading to spontaneous separation by crystallization. Overall, the equilibria involving other competing species are shifted towards adduct **47b**.

### 2.10. Acetylation of Indanol Imines 

**Scheme 9:**
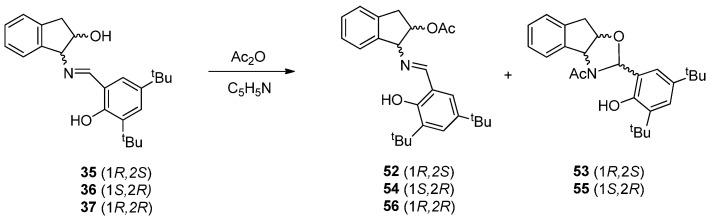
Acetylation of indanol imines.

The acetylation of compounds **35**–**37** was carried out with the aim of studying their transformation into *N*-acetyl oxazolidines under acylating conditions. Compound **35** was chosen as the model to optimize the reaction conditions, which involved the treatment with acetic anhydride in pyridine at room temperature for 24 h, followed by precipitation in ice water. The crude product was found to be a mixture of the acetylated imine **52** and *N*-acetyl oxazolidine **53**. By fractional crystallization from ethanol, compound **52** was isolated as a yellow crystalline solid, followed by **53** as colorless crystals. Likewise, compounds **54** and **55** could be obtained from **36** (Scheme 9). However, when the reaction was carried out with *trans*-imine **37**, only acetyl imine **56** was formed. Probably the origin of this behavior is due to the *trans* arrangement of the C=N and OH groups, which prevent the closure of a five-membered ring. Interestingly, in all the isolated products **52**–**56**, the phenolic hydroxyl remained unacetylated. At first glance, we conjectured that they underwent deacetylation during the ice water treatment used for isolation; however, as we shall see later, the reason is a strong steric hindrance experienced by the phenolic hydroxyl, flanked by the imine function and a bulky *tert*-butyl group.

As is customary, structures assigned to **52**–**56** are supported by analytical and spectroscopic data. The main difference among these heterocyclic derivatives, as inferred from FT-IR spectra (Table 10), is that imines **52**, **54** and **56** show two absorption bands at ~1738 cm^−1^ and ~1627 cm^−1^, corresponding to the stretching vibrations of the C=O (acetate) and C=N (imine) bonds, respectively; for oxazolidines **53** and **55**, that spectral zone only shows the stretching vibration of the amide bond at ~1622 cm^−1^ (Figure S14 highlights this difference in compounds **52** and **53**).

Moreover, NMR data recorded in the solution allowed us to differentiate between both structures (Tables S8 and S9). Assignments were aided by COSY and HMQC two-dimensional correlations. ^1^H NMR spectra of **52**, **54** and **56** display a singlet signal at 8.5 ppm corresponding to the iminic proton, while oxazolidines **53** and **55** show the proton of the new chiral center at ~6.6 ppm. The phenolic hydroxyl signal in imines appears at a a much lower field (~13.6 ppm) than in oxazolidines (~9.2 ppm), owing to the existence of a strong intramolecular hydrogen bond in the first compounds. Acetate groups of imines appear at ~2.1 ppm and acetamido groups in oxazolidines at ~2.4 ppm (Tables S8 and S9). Likewise, ^13^C NMR spectra show the iminic carbon at ~167 ppm, while the C-2 resonance in the oxazolidine derivatives appears at ~87 ppm (see Figures S15 and S16 for ^1^H and ^13^C NMR spectra of compounds **52** and **53**).

**Figure 26:**
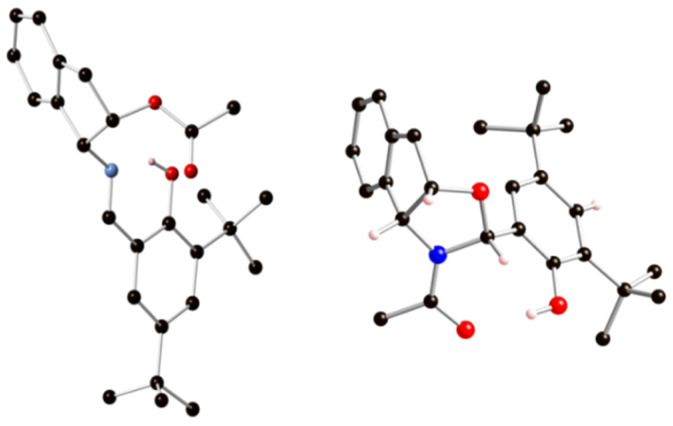
Crystal structures (ball-and-stick models) of **52** (**left**) and **53** (**right**).

Finally, unequivocal solid-state structures of compounds **52** and **53** could be determined by X-ray diffraction (Figure 26). As noted above, compound **52** arises from monoacetylation of imine **35**, while the phenolic hydroxyl remains unprotected as a result of a crowding environment. The position of the hydrogen atom on oxygen evidences an imine tautomer having an intramolecular H-bonded structure.

When comparing the experimental bond lengths (X-ray data) of **52** collected in Table 11 with those of Table 1, both geometries agree with a phenoliminic structure. The values of 1.288 Å and 1.365 Å correspond to typical C=N and C-O bond lengths of imines.

The crystallographic analysis was also instrumental in unambiguously verifying the *N*-acetyl oxazolidine skeleton of **53** and the absolute (*R*)-configuration at the newly created chiral carbon atom. Remarkably, no chirality can be assigned to the nitrogen atom, essentially when it is flat and has a low degree of pyramidalization (<2°) due to the intense delocalization of the lone pair on the carbonyl group.

P = 360 − (120.1 + 128.3 + 109.9) = 1.7°

Like imines, the phenolic OH group of oxazolidines remains unprotected upon acetylation. In lieu of an intramolecular hydrogen bridge involving the endocyclic oxygen leading to a six-membered ring, the OH group is capable of closing an eight-membered ring with the amide carbonyl group (Table S10). In addition, this H-bonding anchors a *Z*-configuration around the amide bond.

### 2.11. Imine Formation versus Oxazolidine Rings

**Figure 27:**
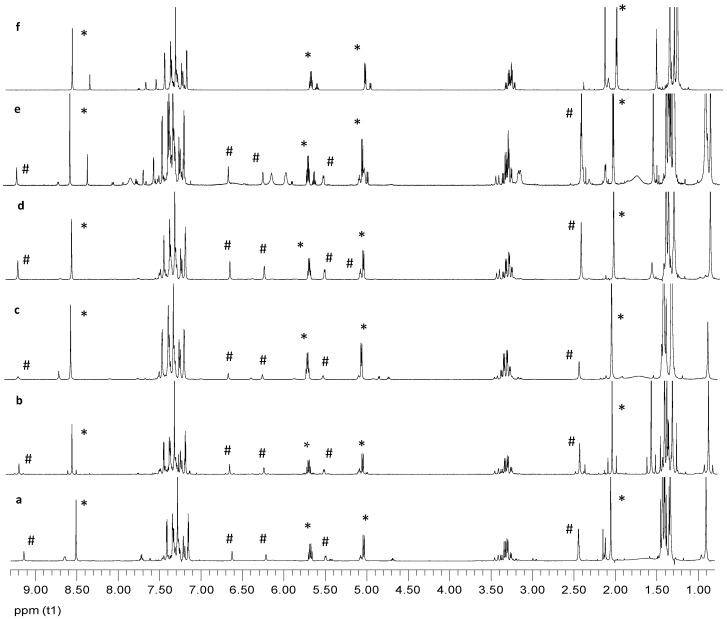
^1^H NMR spectra recorded in CDCl_3_ of acetylation experiments on compound **35** (compounds **52**, *, and **53**, #).

The concomitant formation of imines and oxazolidines through acetylation suggests that their synthesis could be dictated by the interplay of thermodynamic and kinetic conditions. To this end, we performed a series of acetylation experiments on compound **35** at different temperatures and conditions. Results are summarized below and illustrated by NMR spectra (Figure 27).

(a) When imine **35** is acetylated in pyridine at room temperature and worked-up as usual (poured into ice-water after 24 h), the crude product consisted in *O*-acetylated imine (**52**) and *N*-acetylated oxazolidine (**53**) in a 5:2 ratio.
(b) Acetylation of **35** under the above conditions and then brought to dryness to avoid the aqueous work-up resulted in the same 5:2 ratio of **52** and **53**. Clearly, the phenolic hydroxyl substituents do not undergo acetylation, thus evidencing a strong steric hindrance imposed by adjacent groups. It is known that this acetylation reaction is very sensitive to steric crowding when the tetrahedral intermediate leading to acetate is generated [76,77].
(c) Acetylation of imine **35** in pyridine at −20 °C and worked up after 24 h by pouring the reaction into ice water resulted in a crude mixture of **52**:**53** in 7:1 ratio.
(d) Acetylation of imine **35** in pyridine at 70 °C for 2 h followed by aqueous work-up decreased the **52**:**53** ratio to 2:1.
(e) When imine **35** is acetylated in pyridine at higher temperatures (110–120 °C) for 2 h followed by aqueous work-up, the **52**:**53** ratio decreased further (1:1). Moreover, additional signals appeared in the proton NMR spectrum, most likely due to thermal decomposition.
(f) Finally, to determine whether or not imine–oxazolidine interconversion takes place, compound **52** was subjected to acetylation (in pyridine at 110–120 °C) for 2 h, followed by product isolation after aqueous work-up. NMR monitoring gave no indication of oxazolidine (**53**) formation, albeit peaks accounting for a new side product (4:1 ratio) could be observed. The latter might be ascribed to per-*O*-diacetylated imine. Thus, at a high temperature, the formation of oxazolidine with respect to imine is favored, which was a behavior pointing to kinetic control during the formation of compounds **52** and **53**.

**Figure 28:**
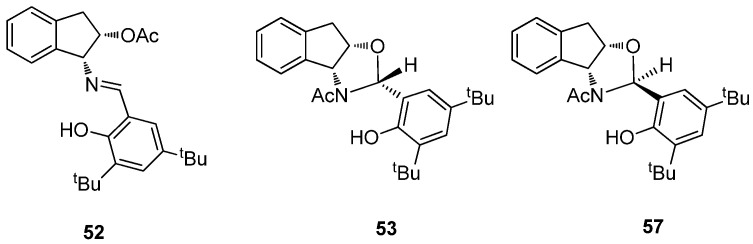
Stereochemistry of **52**, **53** and **57**.

A theoretical analysis to determine the relative stability of imine and oxazolidine structures was also carried out. Table 12 shows such results for imine **52**, (*R*)-configured (**53**) and (*S*)-configured (**57**) oxazolidines, computed in the gas phase and pyridine as solvent (Figure 28). The imine structure is invariably the most stable species, followed by oxazolidine **53**, whose configuration is coincidental with the experimental result. It is surprising that the intramolecular hydrogen bond is established with the acetamido group (**53**) and not with the endocyclic oxygen atom through a six-membered cycle (**53a**). However, calculations support the former, whose arrangement is more stable than that of **53a**. The geometric parameters of hydrogen bonds for structures **52**, **53** and **53a** are collected in Table S11. 

**Figure 29:**
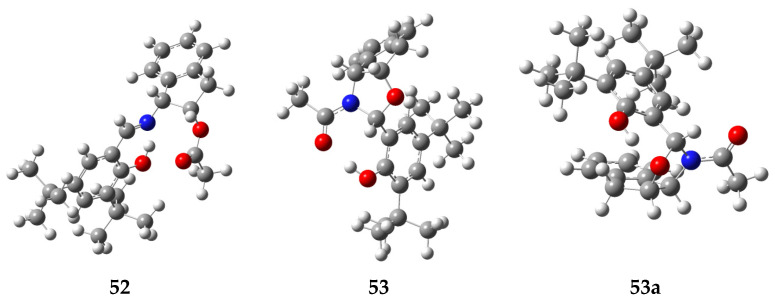
DFT-calculated structures of **52**, **53** and **53a**.

Notably, the computed structures for **52** and **53**, shown in Figure 29, are practically coincidental with those determined by single-crystal X-ray diffraction. The fact that imine **52** is experimentally formed at a greater extent, and that the proportion of oxazolidine **53** increases as temperature increases, would apparently be consistent with a kinetically controlled transformation. However, calculations show that **52** is actually more stable than **53** and the overall transformation proceeds in fact under thermodynamic control.

## 3. Materials and Methods

### 3.1. General Methods

All solvents and chemicals were purchased from commercial suppliers and used without further purification. Solvents were evaporated below 40 °C at pressures between 15 and 30 mm Hg. Melting points were measured on an Electrothermal 9100 apparatus in capillary tubes and are uncorrected. Optical rotations were determined on a Perkin-Elmer 141 polarimeter at different wavelengths (Na and Hg lamps), as specified. Elemental analyses for C, H, N and S were performed using a Leco CHNS-932 analyzer. FTIR spectra were recorded on a Thermo IR-300 spectrophotometer (4000–600 cm^−1^ range) using KBr pellets. All reactions were monitored by thin-layer chromatography (TLC) on Aldrich Polygram Sil G/UV254 plates (7 × 3 cm). ^1^H NMR (500, 400 MHz) and ^13^C NMR (125, 100 MHz) spectra were obtained using Bruker Avance spectrometers, which were recorded at room temperature in CDCl_3_ and DMSO-*d*_6_. Chemical shifts are reported in parts per million (*δ*) downfield from tetramethylsilane (Me_4_Si, TMS) as internal reference. Coupling constants (*J* values) are given in Hz and standard abbreviations were used to indicate spin multiplicities, namely s = singlet, bs = broad singlet, d = doublet, t = triplet, q = quartet, dd = doublet of doublet, m = multiplet. Carbon chemical shifts in the decoupled ^13^C{^1^H} NMR spectra are reported relative to CHCl_3_ (*δ*_C_ 77.00 ppm, central line of triplet). The nature of the carbons (C, CH, CH_2_ and CH_3_) was determined by recording the DEPT spectra. The latter, along with 2D NMR correlations, enabled the assignment of signals. Computational data were obtained using the Gaussian09 program package [57]. All structures were optimized through density functional theory (DFT) [46,47,48,49,50,51,52,53] using the M06-2X method [54] and the 6-311G(d,p) basis set [55,56]. The stationary points were confirmed by frequency analysis at the above-mentioned level of theory at 298.15 K. Solvent effects were calculated using the solvation model density (SMD) [58]. Intrinsic reaction coordinate (IRC) analysis validated that all the transition structures belonged to the reaction path.

Compounds **35** and **36** were purchased from Aldrich and used as received.

### 3.2. Computation

Computational data were obtained using the Gaussian09 program package [57]. All structures were optimized through density functional theory (DFT) [46,47,48,49,50,51,52,53] using the M06-2X method [54] and the 6-311G(d,p) basis set [55,56]. In all cases, frequency calculations were also carried out to confirm the existence of true stationary points on the potential energy surface. All thermal corrections were calculated at the standard values of 1 atm at 298.15 K. Solvent effects were calculated using the solvation model density (SMD) [58].

### 3.3. X-ray Data Collection and Structural Refinement

Suitable crystals were selected and mounted on a MITIGEN holder in perfluoroether oil, using a Rigaku AFC12 FRE-HF diffractometer. The crystal was kept at T = 100(2) K during data collection. Using Olex2 [78], the structure was solved with the ShelXT structure solution program [79], using the Direct Methods solution method. The model was refined with a version of ShelXL [80] using Least Squares minimization (see Tables S12–S18 for a survey of crystallographic data).

### 3.4. Synthetic Procedures: Method A

A solution of 1-amino-2-indanol (1.0 mmol) in the minimum amount of ethanol was added to a solution of the corresponding salicylaldehyde (1.0 mmol) in ethanol. The mixture was kept under stirring at room temperature and, after a few minutes, the formation of a precipitate could be observed. This solid phase was filtered, washed with cold ethanol, dried, and recrystallized from ethanol.

### 3.5. Synthetic Procedures: Method B

A solution of 1-amino-2-indanol (1.0 mmol) in diethyl ether was added to a solution of the corresponding salicylaldehyde (2.0 mmol) in diethyl ether. The mixture was stirred at room temperature for 24 h and then evaporated under reduced pressure to afford the corresponding imine.

### 3.6. Synthetic Procedures: Method C

A solution of 1-amino-2-indanol (1.0 mmol) in ethanol was added to a solution of the corresponding salicylaldehyde (2.0 mmol) in ethanol. The mixture was stirred at room temperature for 24 h. Then, it was stored at 5 °C for one week, evaporated to dryness, and the resulting oil crystallized from diethyl ether. 

*(1R,2S)-1-[(2-Hydroxy-5-methoxybenzylidene)amino]-2-indanol* (**24**). Following procedure A and from 5-methoxysalicylaldehyde, compound **24** was obtained (75%); m.p. 103–105 °C; [α]_D_^20^ − 16.4°; [α]_578_^20^ − 14.5°; [α]_546_^20^ − 4.2° (*c* 0.5, DMSO); IR (KBr) $\bar{\nu }$_max_/cm^−1^ 3362 (OH), 1647 (C=N), 1623, 1588 (C=C), 1531, 1490 (arom); Raman $\bar{\nu }$_max_/cm^−1^ 1639 (C=N), 1619 (C=C); ^1^H NMR (500 MHz, DMSO-*d_6_*) δ 13.05 (s, 1H, OH), 8.67 (s, 1H, CH=N), 7.31 (d, *J* = 7.5 Hz, 1H, H-arom), 7.26 (dt, *J* = 1.5 Hz, *J* = 8.0 Hz, 1H, H-arom), 7.24 (m, 2H, H-arom), 7.10 (d, *J* = 3.5 Hz, 1H, H-arom), 6.95 (dd, *J* = 3.0 Hz, *J* = 9.0 Hz, 1H, H-arom), 6.79 (d, *J* = 9 Hz, 1H, H-arom), 5.18 (d, *J_2,OH_* = 5.0 Hz, 1H, C2-OH), 4.74 (d, *J_1,2_* = 5.5 Hz, 1H, H-1), 4.54 (q, *J* = 6.0 Hz, 1H, H-2), 3.73 (s, 3H, CH_3_), 3.10 (dd, *J_2,3_* = 6.5 Hz, *J_3,3′_* = 15.5 Hz, 1H, H-3), 2.93 (dd, *J_2,3′_* = 6.0 Hz, *J_3,3′_* = 15.5 Hz, 1H, H-3′); ^13^C NMR (125 MHz, DMSO-*d_6_*) δ 165.19 (CH=N), 154.62 (C-OH), 151.15, 141.82, 140.89, 127.76, 126.39, 124.85, 124.43, 119.11, 118.35, 116.99, 114.65 (C-arom), 74.06, 73.83 (C1, C2), 55.30 (CH_3_), 39.54 (C3). Anal. Calculated for C_17_H_17_NO_3_: C, 72.07; H, 6.05; N, 4.94. Found: C, 71.87; H, 6.15; N, 4.82. 

*(1R,2S)-1-[(5-Bromo-2-hydroxybenzylidene)amino]-2-indanol* (**25**). Using procedure A and from 5-bromosalicylaldehyde: (51%); m.p. 148–150 °C; [α]_D_^22^ − 20.8°; [α]_578_^22^ − 18.1°; [α]_546_^22^ − 5.6° (*c* 0.5, DMSO); IR (KBr) $\bar{\nu }$_max_/cm^−1^ 3330 (OH), 1648 (C=N), 1627, 1605 (C=C), 1514, 1499, 1477 (arom); Raman $\bar{\nu }$_max_/cm^−1^ 1638 (C=N), 1625 (C=C); ^1^H NMR (500 MHz, DMSO-*d_6_*) δ 13.92 (bs, 1H, OH), 8.68 (s, 1H, CH=N), 7.70 (d, *J* = 3.0 Hz, 1H, H-arom), 7.44 (dd, *J* = 2.5 Hz, *J* = 8.5 Hz, 1H, H-arom), 7.31 (d, *J* = 7.0 Hz, 1H, H-arom), 7.26 (dt, *J* =1.5 Hz, *J* = 6.5 Hz, 1H, H-arom), 7.19 (m, 2H, H-arom), 6.79 (d, *J* = 8.5 Hz, 1H, H-arom), 5.28 (d, *J_2,OH_* = 5.0 Hz, 1H, C2-OH), 4.79 (d, *J_1,2_* = 5.5 Hz, 1H, H-1), 4.55 (q, *J* = 5.5 Hz, 1H, H-2), 3.11 (dd, *J_2,3_* = 6.0 Hz, *J_3,3′_* = 16.0 Hz, 1H, H-3), 2.91 (dd, *J_2,3′_* = 6.0 Hz, *J_3,3′_* = 15.5 Hz, 1H, H-3′); ^13^C NMR (125 MHz, DMSO-*d_6_*) δ 164.54 (CH=N), 161.26 (C-OH), 141.48, 141.06, 134.83, 133.61, 128.05, 126.60, 125.05, 124.58, 120.13, 119.39, 108.32 (C-arom), 73.79, 73.47 (C1, C2), 39.02 (C3). Anal. Calculated for C_16_H_14_BrNO_2_: C, 57.85; H, 4.25; N, 4.22. Found: C, 57.66; H, 4.27; N, 4.11.

*(1S,2R)-1-[(2-Hydroxy-5-methoxybenzylidene)amino]-2-indanol* (**31**). Using procedure A and from 5-methoxysalicylaldehyde: (59%); m.p. 100–102 °C; [α]_D_^22^ + 13.1°; [α]_578_^22^ + 11.3°; [α]_546_^22^ + 1.4° (*c* 0.5, DMSO); IR (KBr) $\bar{\nu }$_max_/cm^−1^ 3332 (OH), 1645 (C=N), 1624, 1588 (C=C), 1523, 1492, 1475 (arom); Raman $\bar{\nu }$_max_/cm^−1^ 1639 (C=N), 1619 (C=C); ^1^H NMR (500 MHz, DMSO-*d_6_*) δ 13.05 (s, 1H, OH), 8.67 (s, 1H, CH=N), 7.31 (d, *J* = 7.5 Hz, 1H, H-arom), 7.26 (dt, *J* = 1.5 Hz, *J* = 7.0 Hz, 1H, H-arom), 7.24 (m, 2H, H-arom), 7.10 (d, *J* = 3.0 Hz, 1H, H-arom), 6.95 (dd, *J* = 3.5 Hz, *J* = 9.0 Hz, 1H, H-arom), 6.78 (d, *J* = 9.0 Hz, 1H, H-arom), 5.18 (d, *J_2,OH_* = 5.0 Hz, 1H, C2-OH), 4.74 (d, *J_1,2_* = 5.0 Hz, 1H, H-1), 4.54 (q, *J* = 5.5 Hz, 1H, H-2), 3.73 (3H, s, CH_3_), 3.10 (dd, *J_2,3_* = 6.0 Hz, *J_3,3′_* = 15.5 Hz, 1H, H-3), 2.93 (dd, *J_2,3′_* = 6.0 Hz, *J_3,3′_* = 15.5 Hz, 1H, H-3′); ^13^C NMR (125 MHz, DMSO-*d_6_*) δ 165.53 (CH=N), 154.95 (C-OH), 151.48, 142.15, 141.23, 128.09, 126.73, 125.18, 124.76, 119.44, 118.68, 117.32, 114.98 (C-arom), 74.39, 73.17 (C1, C2), 55.63 (CH_3_) 39.19 (C3); ^1^H NMR (500 MHz, CDCl_3_) δ 12.42 (sa, 1H, OH), 8.55 (s, 1H, CH=N), 7.30 (m, 2H, H-arom), 7.23 (dt, *J* = 1.5 Hz, *J* = 7.5 Hz, 1H, H-arom), 7.24 (d, *J* = 7.0 Hz, 1H, H-arom), 6.95 (dd, *J* = 3.5 Hz, *J* = 9.0 Hz, 1H, H-arom), 6.89 (d, *J* = 9.0 Hz, 1H, H-arom), 6.84 (d, *J* = 3.0 Hz, 1H, H-arom), 4.80 (d, *J_1,2_* = 5.5 Hz, 1H, H-1), 4.69 (c, *J* = 5.5 Hz, 1H, H-2), 3.79 (s, 3H, CH_3_), 3.24 (dd, *J_2,3_* = 6.0 Hz, *J_3,3′_* = 16.0 Hz, 1H, H-3), 3.09 (dd, *J_2,3′_* = 5.5 Hz, *J_3,3′_* = 16.0 Hz, 1H, H-3′); ^13^C NMR (125 MHz, CDCl_3_) δ 166.60 (CH=N), 155.13 (C-OH), 152.15, 140.71, 140.65, 128.62, 127.06, 125.48, 124.84, 120.06, 118.29, 117.91, 115.13 (C-arom), 75.66, 75.27 (C1, C2), 55.94 (CH_3_), 39.60 (C3).

*(1S,2R)-1-[(5-Bromo-2-hydroxybenzylidene)amino]-2-indanol* (**32**). Using procedure A and from 5-bromosalicylaldehyde: (59%); m.p. 147–149 °C; [α]_D_^25^ + 17.2°; [α]_578_^25^ + 14.4°; [α]_546_^25^ + 2.8° (*c* 0.5, DMSO); IR (KBr) $\bar{\nu }$_max_/cm^−1^ 3329 (OH), 1648 (C=N), 1627, 1605 (C=C), 1515, 1477 (arom); Raman $\bar{\nu }$_max_/cm^−1^ 1638 (C=N), 1625 (C=C); ^1^H NMR (500 MHz, DMSO-*d_6_*) δ 13.91 (1H, s, OH), 8.69 (s, 1H, CH=N), 7.72 (d, *J* = 2.5 Hz, 1H, H-arom), 7.44 (dd, *J* = 2.5 Hz, *J* = 9.0 Hz, 1H, H-arom), 7.31 (d, *J* = 7.5 Hz, 1H, H-arom), 7.26 (dt, *J* = 2.0 Hz, *J* = 8.5 Hz, 1H, H-arom), 7.19 (m, 2H, H-arom), 6.79 (d, *J* = 9.0 Hz, 1H, H-arom), 5.24 (d, *J_2,OH_* = 5.0 Hz, 1H, C2-OH), 4.79 (d, *J_1,2_* = 5.5 Hz, 1H, H-1), 4.55 (q, *J* = 5.5 Hz, 1H, H-2), 3.11 (dd, *J_2,3_* = 6.0 Hz, *J_3,3′_* = 15.5 Hz, 1H, H-3), 2.91 (dd, *J_2,3′_* = 5.5 Hz, *J_3,3′_* = 15.5 Hz, 1H, H-3′); ^13^C NMR (125 MHz, DMSO-*d_6_*) δ 164.06 (CH=N), 160.77 (C-OH), 140.98, 140.57, 134.34, 133.09, 127.56, 126.11, 124.56, 124.09, 119.64, 118.91, 107.83 (C-arom), 73.29, 72.97 (C1, C2), 38.52 (C3). Anal. Calculated for C_16_H_14_BrNO_2_: C, 57.85; H, 4.25; N, 4.22. Found: C, 57.56; H, 4.31; N, 3.93.

*(1R,2R)-1-[(3,5-Di-tert-butyl-2-hydroxybenzylidene)amino]-2-indanol* (**37**). Using procedure A and from 3,5-di-*tert*-butylsalicylaldehyde: (78%); m.p. 69–71 °C; [α]_D_^25^ − 48.5°; [α]_578_^25^ − 49.5°; [α]_546_^25^ + 50.9° (*c* 0.5, DMSO); IR (KBr) $\bar{\nu }$_max_/cm^−1^ 3339 (OH), 1627 (C=N), 1596, 1475 (arom); Raman $\bar{\nu }$_max_/cm^−1^ 1622 (C=N), 1591 (C=C); ^1^H NMR (500 MHz, DMSO-*d_6_*) δ 13.96 (s, 1H, OH), 8.75 (s, 1H, CH=N), 7.39 (d, *J* = 2.0 Hz, 1H, H-arom),7.34 (s, 1H, H-arom), 7.23 (m, 3H, H-arom), 7.08 (d, *J* = 7.5 Hz, 1H, H-arom), 5.53 (d, *J_2,OH_* = 5.5 Hz, 1H, C2-OH), 4.63 (d, *J_1,2_* = 6.0 Hz, 1H, H-1), 4.39 (q, *J_1,2_* = *J_2,3_* = *J_2,OH_* = 6.5 Hz, 1H, H-2), 3.23 (dd, *J_2,3_* = 7.0 Hz, *J_3,3′_* = 15.5 Hz, 1H, H-3), 2.84 (dd, *J_2,3′_* = 8.0 Hz, *J_3,3′_* = 15.5 Hz, 1H, H-3′), 1.37 (s, 9H, CH_3_), 1.29 (s, 9H, CH_3_); ^13^C NMR (125 MHz, DMSO-*d*_6_) δ 167.79 (CH=N), 157.37 (C-OH), 141.50, 139.66, 139.53, 135.45, 127.85, 126.72, 126.52, 126.14, 124.68, 123.78, 117.78 (C-arom), 79.17, 78.94 (C1,C2), 38.92 (C3), 34.43, 33.76 (C-(CH_3_)_3_), 31.19, 29.14 (CH_3_). Anal. Calculated for C_28_H_33_NO_3_: C, 76.62; H, 8.12; N, 3.44. Found: C, 76.31; H, 7.95; N, 3.33.

*(6Z)-6-[(1R,2S)-(2-hydroxyindan-1-yl)amino]methylen]-4-nitrocyclohexa-2,4-dienone* (**38**). Using procedure A and from 5-nitrosalicylaldehyde, compound **38** was obtained: (91%); m.p. 212–214 °C; [α]_D_^20^ + 35.2°; [α]_578_^20^ + 40.9°; [α]_546_^20^ + 63.9° (*c* 0.5, DMSO); IR (KBr) $\bar{\nu }$_max_/cm^−1^ 3174 (OH), 1655 (C=O), 1611 (C=C), 1544 (NO_2_), 1515, 1441 (arom), 1330 (NO_2_); Raman $\bar{\nu }$_max_/cm^−1^ 1655 (C=O), 1603 (C=C); ^1^H NMR (500 MHz, DMSO-*d_6_*) δ 14.49 (bs, 1H, NH), 8.94 (s, 1H, CH-N), 8.53 (d, *J* =3.0 Hz, 1H, H-cyclo), 8.04 (dd, *J* = 3.0 Hz, *J* =9.5 Hz, 1H, H-cyclo), 7.33 (m, 3H, H-arom), 7.27 (m, 1H, H-arom), 6.58 (d, *J* = 9.5 Hz, 1H, H-cyclo), 5.74 (d, *J_2,OH_* = 2.5 Hz, 1H, C2-OH), 5.20 (d, *J_1,2_* = 4.5 Hz, 1H, H-1), 4.65 (bs, 1H, H-2), 3.18 (dd, *J_2,3_* = 5.5 Hz, *J_3,3′_* = 16.0 Hz, 1H, H-3), 2.91 (dd, *J_2,3′_* = 2.5 Hz, *J_3,3′_* = 16.0 Hz, 1H, H-3′); ^13^C NMR (125 MHz, DMSO-*d_6_*) δ 178.06 (C=O), 166.86 (C-NH), 140.99, 138.77, 133.45, 132.74, 129.12, 128.55, 126.70, 125.19, 124.36, 122.75, 113.30 (C-arom), 72.04, 68.09 (C1, C2), 39.93 (C3). Anal. Calculated for C_18_H_14_N_2_O_4_: C, 64.42; H, 4.73; N, 9.39. Found: C, 64.66; H, 4.81; N, 9.64.

*(6Z)-6-[(1R,2S)-(2-hydroxyindan-1-yl)amino]methylen]-5-bromocyclohexa-2,4-dienone* (**39**)**.** Using procedure A and from 5-bromosalicylaldehyde: (62%); m.p. 179–181 °C; [α]_D_^20^ + 109.01°; [α]_578_^20^ + 121.10°; [α]_546_^20^ + 170.11° (*c* 0.5, DMSO); IR (KBr) $\bar{\nu }$_max_/cm^−1^ 3174 (OH), 1642 (C=N), 1597, 1502, 1454 (arom); Raman $\bar{\nu }$_max_/cm^−1^ 1641 (C=N);^1^H NMR (500 MHz, DMSO-*d_6_*) δ 14.32 (bs, 1H, OH), 8.69 (s, 1H, CH=N), 7.39 (d, *J* = 8.0 Hz, 1H, H-arom), 7.32 (d, *J* = 7.0 Hz, 1H, H-arom), 7.28 (m, 1H, H-arom), 7.21 (m, 2H, H-arom), 6.97 (d, *J* = 1.5 Hz, 1H, H-arom), 6.92 (dd, *J* = 2.0 Hz, *J* = 8.5 Hz, 1H, H-arom), 5.33 (d, *J_2,OH_* = 5.0 Hz, 1H, C2-OH), 4.87 (d, *J_1,2_* = 5.0 Hz, 1H, H-1), 4.56 (q, *J* = 5.5 Hz, 1H, H-2), 3.12 (dd, *J_2,3_* = 6.0 Hz, *J_3,3′_* = 16.0 Hz, 1H, H-3), 2.91 (dd, *J_2,3′_* = 5.0 Hz, *J_3,3′_* = 15.5 Hz, 1H, H-3′); ^13^C NMR (125 MHz, DMSO-*d_6_*) δ 165.89 (C-OH), 165.23 (CH=N), 141.03, 133.75, 128.15, 126.64, 125.11, 124.53, 120.88, 119.60, 116.78 (C-arom), 73.39, 71.99 (C1, C2), 39.09 (C3). Anal. Calculated for C_16_H_14_BrNO_2_: C, 57.85; H, 4.25; N, 4.22. Found: C, 57.73; H, 4.29; N, 4.19.

*(6Z)-6-[(1R,2S)-(2-hydroxyindan-1-yl)amino]methylen]-5-methoxycyclohexa-2,4-dienone***(40).** Using procedure A and from 4-methoxysalicylaldehyde: (86%); m.p. 169–171 °C; [α]_D_^22^ + 157.9°; [α]_578_^22^ + 172.5°; [α]_546_^22^ + 228.4° (*c* 0.5, DMSO); IR (KBr) $\bar{\nu }$_max_/cm^−1^ 3182 (OH), 1644 (C=O), 1616 (C=C), 1514, 1484 (arom); Raman $\bar{\nu }$_max_/cm^−1^ 1638 (C=O), 1608 (C=C); ^1^H NMR (500 MHz, DMSO-*d_6_*) δ 14.01 (s, 1H, OH), 8.49 (s, 1H, CH=N), 7.31 (d, *J* = 7.5 Hz, 1H, H-cyclo), 7.26 (m, 2H, H-arom), 7.21 (m, 2H, H-arom), 6.30 (dd, *J* = 2.5 Hz, *J* = 8.5 Hz, 1H, H-cyclo), 6.20 (d, *J* = 2.5 Hz, 1H, H-cyclo), 5.30 (d, *J_2,OH_* = 4.5 Hz, 1H, C2-OH), 4.81 (d, *J_1,2_* = 5.5 Hz, 1H, H-1), 4.54 (q, *J* = 4.5 Hz, 1H, H-2), 3.74 (s, 3H, CH_3_), 3.11 (dd, *J_2,3_* = 6.0 Hz, *J_3,3′_* = 16.0 Hz, 1H, H-3), 2.90 (dd, *J_2,3′_* = 4.5 Hz, *J_3,3′_* = 15.5 Hz, 1H, H-3′); ^13^C NMR (125 MHz, DMSO-*d_6_*) δ 168.92 (C-OH), 164.03 (CH=N), 163.93, 141.48, 140.94, 133.56, 127.99, 126.59, 125.07, 124.40, 111.68, 105.49, 101.24 (C-arom), 73.33, 71.26 (C1, C2), 55.07 (CH_3_) 39.35 (C3). Anal. Calculated for C_17_H_17_NO_3_: C, 72.07; H, 6.05; N, 4.94. Found: C, 71.83; H, 6.05; N, 4.91.

*(6Z)-6-[(1R,2S)-(2-hydroxyindan-1-yl)amino]methylen]-4,6-dinitrocyclohexa-2,4-dienone***(41).** Using procedure A and from 3,5-dinitrosalicylaldehyde: (80%); m.p. 145–147 °C; [α]_D_^25^ + 211.5°; [α]_578_^25^ + 234.34°; [α]_546_^25^ + 334.9°; (*c* 0.5, DMSO); IR (KBr) $\bar{\nu }$_max_/cm^−1^ 3407, 3350 (OH), 1650 (C=O), 1616 (C=C), 1556 (NO_2_) 1531 (arom); Raman $\bar{\nu }$_max_/cm^−1^ 1666 (C=O); ^1^H NMR (500 MHz, DMSO-*d_6_*) δ 13.86 (bs, 1H, NH), 9.10 (d, *J* = 14.0 Hz, 1H, CH-N), 8.82 (d, *J* = 2.5 Hz, 1H, H-cyclo), 8.76 (d, *J* = 3.0 Hz, 1H, H-cyclo), 7.36 (m, 3H, H-arom), 7.28 (dt, *J* = 2.5 Hz, *J* = 6.5 Hz, 1H, H-arom), 5.94 (d, *J_2,OH_* = 4.5 Hz, 1H, C2-OH), 4.37 (t, *J_1,2_ = J_NH,1_* = 7.0 Hz, 1H, H-1), 4.70 (m, 1H, H-2), 3.22 (dd, *J_2,3_* = 5.5 Hz, *J_3,3′_* = 16.5 Hz, 1H, H-3), 2.94 (dd, *J_2,3′_* = 5.0 Hz, *J_3,3′_* = 16.5 Hz, 1H, H-3′); ^13^C NMR (125 MHz, DMSO-*d_6_*) δ 169.99 (C=O), 167.97 (CH-N), 141.36,140.83, 137.67, 137.31, 129.88, 129.04, 127.00, 126.96, 125.46, 124.70, 117.06 (C-arom), 71.79, 67.51 (C1, C2), 39.68 (C3). Anal. Calculated for C_16_H_13_N_3_O_6_: C, 55.98; H, 3.82; N, 12.24. Found: C, 55.98; H, 3.85; N, 12.25.

*(6Z)-6-[(1R,2S)-(2-hydroxyindan-1-yl)amino]methylen]cyclohexa-2,4-dienone***(42).** Using procedure B and from salicylaldehyde: (28%); m.p. 113–115 °C; [α]_D_^22^ + 16.3°; [α]_578_^22^ + 19.4°; [α]_546_^22^ + 36.3 (*c* 0.5, DMSO); IR (KBr) $\bar{\nu }$_max_/cm^−1^ 3015 (OH), 1634 (C=O), 1607 (C=C), 1513, 1473 (arom); Raman $\bar{\nu }$_max_/cm^−1^ 1635 (C=O); ^1^H NMR (500 MHz, DMSO-*d_6_*) δ 13.73 (s, 1H, OH), 8.71 (s, 1H, CH=N), 7.50 (dd, *J* = 2.0 Hz, *J* = 8.0 Hz, 1H, H-arom), 7.33 (dd, *J* = 2.0 Hz, *J* = 8.5 Hz, 2H, H-arom), 7.27 (m, 1H, H-arom), 7.20 (m, 2H, H-arom), 6.87 (dt, *J* = 1.5 Hz, *J* = 7.5 Hz, 1H, H-arom), 6.84 (d, *J* = 8.0 Hz, 1H, H-arom), 5.19 (d, *J_2,OH_* = 5.5 Hz, 1H, C2-OH), 4.78 (d, *J_1,2_* = 5.5 Hz, 1H, H-1), 4.56 (q, *J* = 6.0 Hz, 1H, H-2), 3.13 (dd, *J_2,3_* = 6.0 Hz, *J_3,3′_* = 15.5 Hz, 1H, H-3), 2.94 (dd, *J_2,3′_* = 6.0 Hz, *J_3,3′_* = 15.5 Hz, 1H, H-3′); ^13^C NMR (125 MHz, DMSO-d6) δ 166.24 (CH=N), 161.80 (C-OH), 142.48, 141.60, 132.81, 132.28, 128.50, 127.12, 125.58, 125.15, 119.26, 118.64, 117.14 (C-arom), 74.50, 74, 45 (C1,C2), 39.85 (C3); ^1^H NMR (500 MHz, CD_3_OD) δ 8.61 (s, 1H, CH=N), 7.38 (dd, *J* = 1.5 Hz, *J* = 3.0 Hz, 1H, H-arom), 7.31 (m, 3H, H-arom), 7.21 (m, 2H, H-arom), 6.82 (m, 2H, H-arom), 4.85 (d, *J_1,2_* = 5.0 Hz, 1H, H-1), 4.64 (q, *J_1,2_* = *J_2,3_* = 5.5 Hz, 1H, H-2), 3.21 (dd, *J_2,3_* = 6.0 Hz, *J_3,3′_* = 16 Hz, 1H, H-3), 3.06 (dd, *J_2,3′_* = 5.0 Hz, *J_3,3′_* = 15.5 Hz, 1H, H-3′); ^13^C NMR (125 MHz, CD_3_OD) δ 167.33 (CH=N), 162.49 (C-OH), 142.22, 142.10, 134.53, 129.56, 128.05, 126.33, 125.74, 122.62, 120.91, 119.04, 118.75 (C-arom), 75.76, 74.76 (C1,C2), 40.09 (C3). Anal. Calculated for C_16_H_15_NO_2_: C, 75.87; H, 5.97; N, 5.53. Found: C, 75.98; H, 5.98; N, 5.71.

*(6Z)-6-[(1S,2R)-(2-hydroxyindan-1-yl)amino]methylen]-4-nitrocyclohexa-2,4-dienone***(43).** Using procedure A and from 5-nitrosalicylaldehyde, compound **43** was obtained: (85%); m.p. 219–221 °C; [α]_D_^22^ − 45.5°; [α]_578_^22^ − 52.5°; [α]_546_^22^ − 80.4°; [α]_436_^22^ 43.7° (*c* 0.5, DMSO); IR (KBr) $\bar{\nu }$_max_/cm^−1^ 3209 (OH), 1656 (C=O), 1610 (arom), 1543 (NO_2_), 1514, 1444 (arom), 1330 (NO_2_); Raman $\bar{\nu }$_max_/cm^−1^ 1655 (C=O), 1603 (C=C); ^1^H NMR (500 MHz, DMSO-*d_6_*) δ 14.50 (bs, 1H, NH), 8.95 (s, 1H, CH-N), 8.54 (d, *J* = 3.0 Hz, 1H, H-cyclo), 8.05 (dd, *J* = 3.0 Hz, *J* = 9.5 Hz, 1H, H-cyclo), 7.33 (m, 3H, H-arom), 7.27 (m, 1H, H-arom), 6.59 (d, *J* = 10.0 Hz, 1H, H-cyclo), 5.75 (d, *J_2,OH_* = 3.0 Hz, 1H, C2-OH), 5.20 (d, *J_1,2_* = 5.0 Hz, 1H, H-1), 4.66 (sa, 1H, H-2), 3.19 (dd, *J_2,3_* = 5.5 Hz, *J_3,3′_* = 16.5 Hz, 1H, H-3), 2.91 (dd, *J_2,3′_* = 3.0 Hz, *J_3,3′_* = 16.5 Hz, 1H, H-3′); ^13^C NMR (125 MHz, DMSO-*d_6_*) δ 178.10 (C=O), 166.88 (C-NH), 140.99, 138.78, 133.45, 132.76, 129.14, 128.56, 126.71, 125.20, 124.37, 122.79, 113.30 (C-arom), 72.05, 68.10 (C1, C2), 39.96 (C3). 

*(6Z)-6-[(1S,2R)-(2-hydroxyindan-1-yl)amino]methylen]-5-methoxycyclohexa-2,4-dienone***(44).** Using procedure A and from 4-methoxysalicylaldehyde: (88%); m.p. 170–172 °C; [α]_D_^22^ − 167.6°; [α]_578_^22^ − 182.5°; [α]_546_^22^ − 241.1° (*c* 0.5, DMSO); IR (KBr) $\bar{\nu }$_max_/cm^−1^ 3186 (OH), 1644 (C=O), 1615 (C=C), 1514, 1484, 1456 (arom); Raman $\bar{\nu }$_max_/cm^−1^ 1638 (C=O), 1608 (C=C);^1^H NMR (500 MHz, DMSO-*d_6_*) δ 14.01 (s, 1H, OH), 8.49 (s, 1H, CH=N), 7.31 (d, *J* = 7.0 Hz, 1H, H-cyclo), 7.26 (m, 2H, H-arom), 7.20 (m, 2H, H-arom), 6.30 (dd, *J* = 2.0 Hz, *J* = 8.5 Hz, 1H, H-cyclo), 6.20 (d, *J* = 2.0 Hz, 1H, H-cyclo), 5.29 (d, *J_2,OH_* = 4.0 Hz, 1H, C2-OH), 4.81 (d, *J_1,2_* = 5.0 Hz, 1H, H-1), 4.54 (q, *J* = 4.0 Hz, 1H, H-2), 3.74 (s, 3H, CH_3_), 3.10 (dd, *J_2,3_* = 6.0 Hz, *J_3,3′_* = 16.0 Hz, 1H, H-3), 2.89 (dd, *J_2,3′_* = 5.0 Hz, *J_3,3′_* = 16.0 Hz, 1H, H-3′); ^13^C NMR (125 MHz, DMSO-*d_6_*) δ 168.88 (C-OH), 164.03 (CH=N), 163.92, 141.48, 140.94, 133.54, 127.98, 126.58, 125.06, 124.39, 111.68, 105.48, 101.23 (C-arom), 73.32, 71.26 (C1, C2), 55.06 (CH_3_) 36.68 (C3). Anal. Calculated for C_17_H_17_NO_3_: C, 72.07; H, 6.05; N, 4.94. Found: C, 71.83; H, 6.05; N, 4.91.

*(6Z)-6-[(1S,2R)-(2-hydroxyindan-1-yl)amino]methylen]cyclohexa-2,4-dienone***(45).** Using procedure B and from salicylaldehyde: (35%); m.p. 110–112 °C; [α]_D_^18^ − 15.2°; [α]_578_^18^ − 17.9°; [α]_546_^18^ − 33.6 (*c* 0.5, DMSO); IR (KBr) $\bar{\nu }$_max_/cm^−1^ 3119 (OH), 1639 (C=O), 1611 (C=C), 1513, 1490 (arom); Raman $\bar{\nu }$_max_/cm^−1^ 1635 (C=O); ^1^H NMR (500 MHz, DMSO-*d_6_*) δ 13.74 (s, 1H, OH), 8.71 (s, 1H, CH=N), 7.49 (dd, *J* = 1.0 Hz, *J* = 7.5 Hz, 1H, H-arom), 7.33 (m, 2H, H-arom), 7.27 (m, 1H, H-arom), 7.20 (m, 2H, H-arom), 6.90 (t, *J* = 7.5 Hz, 1H, H-arom), 6.84 (d, *J* = 8.0 Hz, 1H, H-arom), 5.21 (d, *J_2,OH_* = 5.0 Hz, 1H, C2-OH), 4.78 (d, *J_1,2_* = 5.5 Hz, 1H, H-1), 4.56 (q, *J* = 5.5 Hz, 1H, H-2), 3.12 (dd, *J_2,3_* = 6.5 Hz, *J_3,3′_* = 16.0 Hz, 1H, H-3), 2.94 (dd, *J_2,3′_* = 5.5 Hz, *J_3,3′_* = 15.5 Hz, 1H, H-3′); ^13^C NMR (125 MHz, DMSO-*d*_6_) δ 165.87 (CH=N), 161.41 (C-OH), 141.22, 132.44, 131.89, 128.12, 126.74, 125.20, 124.77, 118.26, 116.76 (C-arom), 74.11, 74.08 (C1,C2), 39.27 (C3). Anal. Calculated for C_16_H_15_NO_2_: C, 75.87; H, 5.97; N, 5.53. Found: C, 75.47; H, 5.90; N, 5.35.

*Reaction of (1R,2S)-1-amino-2-indanol with salicylaldehyde***.** Using procedure C and from salicylaldehyde, compound **47** was obtained: (88%); m.p. 157–158 °C; [α]_D_^18^ + 26.1°; [α]_578_^18^ + 27.0°; [α]_546_^18^ + 31.7°; [α]_436_^18^ + 66.0° (*c* 0.5, CHCl_3_); IR (KBr) $\bar{\nu }$_max_/cm^−1^ 3187 (OH), 1619, 1589 (arom), 1253, 1221 (C-O-C), 1027 (C-O); ^1^H NMR (500 MHz, DMSO-*d*_6_) δ 9.91 (s, 1H, OH), 7.81 (dd, *J* = 1.5 Hz, *J* = 7.5 Hz, 1H, H-arom), 7.32 (m, 2H, H-arom), 7.27 (m, 1H, H-arom), 7.24 (m, 2H, H-arom), 7.20 (m, 2H, H-arom), 7.05 (dt, *J* = 1.0 Hz, *J* = 8.0 Hz, 1H, H-arom), 6.99 (m, 2H, H-arom), 6.93 (d, *J* = 8.0 Hz, 1H, H-arom), 5.75 (s, 1H, H-2 oxazine), 4.92 (s, 1H, H-2 oxazolidine), 4.83 (dt, *J_2,3′_* = 1.0 Hz, *J_1,2_* = *J_2,3_ =* 6.0 Hz, 1H, H-2 indanol), 4.61 (d, *J_1,2_* = 5.5 Hz, 1H, H-1 indanol), 3.23 (dd, *J_2,3_* = 6.5 Hz, *J_3,3′_* = 18.0 Hz, 1H, H-3 indanol), 3.14 (d, *J_3,3′_* = 17.5 Hz, 1H, H-3′ indanol); ^13^C NMR (125 MHz, DMSO-*d*_6_) δ 156.66 (C-OH), 153.85, 142.60, 140.05, 130.94, 130.42, 130.04, 129.57, 128.89, 127.48, 125.73, 125.20, 123.88, 121.72, 120.07, 119.73, 116.57, 116.12 (C-arom), 86.72 (C2 oxazolidine), 81.12 (C2 oxazine), 77.17 (C2 indanol), 70.53 (C1 indanol), 40.17 (C3 indanol); ^1^H NMR (500 MHz, CD_3_OD) δ 7.69 (dd, *J* = 1.5 Hz, *J* = 7.5 Hz, 1H, H-arom), 7.32 (m, 6H, H-arom), 7.22 (m, 1H, H-arom), 7.24 (m, 2H, H-arom), 7.04 (m, 2H, H-arom), 6.94 (t, *J* = 8.5 Hz, 2H, H-arom), 5.73 (s, 1H, H-2 oxazine), 5.02 (s, 1H, H-2 oxazolidine), 4.77 (d, *J_1,2_* = *J_2,3_* = 6.0 Hz, 1H, H-2 indanol), 4.62 (s, 1H, H-1 indanol), 3.25 (d, *J_2,3_* = 5.5 Hz, 2H, H-3, H-3′ indanol); ^13^C NMR (125 MHz, CD_3_OD) δ 157.76(C-OH), 155.11, 143.55, 140.64, 132.00, 131.26, 130.98, 130.58, 129.83, 128.42, 126.82, 125.85, 124.38, 122.69, 121.07, 120.49, 117.63, 117,08 (C-arom), 87.94 (C2 oxazolidine) 84.73 (C2 oxazine) 78.70 (C2 indanol), 71.97 (C1 indanol), 40.19 (C3 indanol). HRMS-CI (C_23_H_19_NO_3_ [M+H]^+^): calculated 358.1457; found 358.1443.

*Reaction of (1S,2R)-1-amino-2-indanol with salicylaldehyde.* Using procedure C and from salicylaldehyde, compound **48** was obtained: (20%); m.p. 152–154 °C; [α]_D_^18^ − 23.9°; [α]_578_^18^ − 25.2°; [α]_546_^18^ − 29.3°; [α]_436_^18^ − 61.0° (*c* 0.5, CHCl_3_); IR (KBr) $\bar{\nu }$_max_/cm^−1^ 3201 (OH), 1617, 1587 (arom), 1250, 1225 (C-O-C), 1025 (C-O); ^1^H NMR (500 MHz, DMSO-*d*_6_) δ 9.91 (s, 1H, OH), 7.81 (dd, *J* = 1.5 Hz, *J* = 8.0 Hz, 1H, H-arom), 7.31 (m, 2H, H-arom), 7.27 (m, 1H, H-arom), 7.23 (m, 2H, H-arom), 7.20 (m, 2H, H-arom), 7.05 (t, *J* = 7.5 Hz, 1H, H-arom), 6.98 (m, 2H, H-arom), 6.93 (d, *J* = 8.5 Hz, 1H, H-arom), 5.75 (s, 1H, H-2 oxazine), 4.91 (s, 1H, H-2 oxazolidine), 4.83 (t, *J_1,2_* = *J_2,3_ =* 6.0 Hz, 1H, H-2), 4.60 (d, *J_1,2_* = 6.0 Hz, 1H, H-1), 3.22 (dd, *J_2,3_* = 6.0 Hz, *J_3,3′_* = 18.0 Hz, 1H, H-3), 3.13 (d, *J_3,3′_* = 18.0 Hz, 1H, H-3′); ^13^C NMR (125 MHz, DMSO-*d*_6_) δ 156.65 (C-OH), 153.84, 142.60, 140.03, 130.94, 130.42, 130.04, 129.57, 128.89, 127.48, 125.72, 125.20, 123.86, 121.72, 120.07, 119.72, 116.57, 116.10 (C-arom), 86.71 (C2 oxazolidine), 81.10 (C2 oxazine), 77.16 (C2 indanol), 70.53 (C1 indanol), 39.30 (C3 indanol). 

### 3.7. General Acetylation Procedure 

For a solution of the corresponding imine (1.0 mmol) in pyridine (3.0 mL), acetic anhydride (2.0 mL) was added. The mixture was kept at room temperature for 24 h and then was poured into ice water. The resulting white solid was filtered and washed with water. The mixture containing imine and oxazolidine was purified by fractional crystallization from ethanol. The first crystalline material corresponds to acetylated imine, while the mother liquors contain the oxazolidine derivative. 

*(1R,2S)-2-O-Acetyl-1-[(3,5-di-tert-butyl-2-hydroxybenzylidene)amino]-2-indanol***(52).** Following the above procedure and **35**: 45%; m.p. 144–146 °C; [α]_D_^20^ − 100.0°; [α]_578_^20^ − 103.1°; [α]_546_^20^ − 114.2° (*c* 0.5, CHCl_3_); IR (KBr) $\bar{\nu }$_max_/cm^−1^ 1739 (C=O), 1627 (C=N), 1594, 1473 (arom); ^1^H NMR (400 MHz, CDCl_3_) δ 13.64 (s, 1H, OH), 8.52 (s, 1H, CH=N), 7.41 (d, *J* = 2.4 Hz, 1H, H-arom), 7.34 (m, 2H, H-arom), 7.27 (m, 1H, H-arom), 7.20 (d, *J* = 7.6 Hz, 1H, H-arom), 7.15 (d, *J* = 2.8 Hz, 1H, H-arom), 5.68 (1H, c, *J_1,2_* = *J_2,3_ = J_2,3′_* 5.6 Hz, H-2), 5.04 (d, *J_1,2_* = 6.0 Hz, 1H, H-1), 3.35 (dd, *J_2,3_* = 6.8 Hz, *J_3,3′_* = 16.4 Hz, 1H, H-3), 2.28 (dd, *J_2,3′_* = 5.2 Hz, *J_3,3′_* = 16.4 Hz, 1H, H-3′), 2.07 (s, 3H, OCH_3_), 1.44 (s, 9H, CH_3_), 1.34 (s, 9H, CH_3_); ^13^C NMR (100 MHz, CDCl_3_) δ 171.01 (C=O), 167.00 (CH=N), 158.28 (C-OH), 140.79, 140.04, 139.85, 136.76, 128.66, 127.35,127.30, 126.27,125.07, 124.95, 117.79 (C-arom), 76.06, 73.17 (C1, C2), 36.84 (C3), 35.05, 34.16 (C-(CH_3_)_3_), 31.52, 29.38 (CH_3_), 20.97 (OCH_3_). ^1^H NMR (400 MHz, DMSO-*d*_6_) δ 14.13 (s, 1H, OH), 8.79 (s, 1H, CH=N), 7.36 (m, 4H, H-arom), 7.25 (t, *J* = 6.4 Hz, 1H, H-arom), 7.18 (d, *J* = 7.2 Hz, 1H, H-arom), 5.64 (sa, 1H, H-2), 5.10 (d, *J_1,2_* = 4.8 Hz, 1H, H-1), 3.36 (m, 1H, H-3), 3.11 (dd, *J_2,3′_* = 2.4 Hz, *J_3,3′_* = 16.8 Hz, 1H, H-3′), 1.95 (s, 3H, OCH_3_), 1.36 (s, 9H, CH_3_), 1.29 (s, 9H, CH_3_); ^13^C NMR (100 MHz, DMSO-*d*_6_) δ 170.29 (C=O), 168.99 (CH=N), 158.35 (C-OH), 141.56, 140.37, 140.11, 136.18, 128.82, 127.57, 127.11, 127.08, 125.42, 124.89, 118.20 (C-arom), 76.39, 72.59 (C1,C2), 37.11 (C3), 35.04, 34.34 (C-(CH_3_)_3_), 31.76, 29.68 (CH_3_), 21.05 (OCH_3_). Anal. Calculated for C_26_H_33_NO_3_: C, 76.62; H, 8.16; N, 3.44. Found: C, 76.35; H, 8.07; N, 3.35.

*N-Acetyl-3,5-di-tert-butyl-2-{(2R,3aR,8aS)-3,3a,8,8a-tetrahydro-2H-indeno [1,2-d]oxazol-2-yl}phenol***(53).** Following the general procedure from compound **35**: 11%; m.p. 164–166 °C; [α]_D_^20^ − 208.8°; [α]_578_^20^ − 217.8°; [α]_546_^20^ − 250.3°; [α]_436_^20^ − 449.7° (*c* 0.5, CHCl_3_); IR (KBr) $\bar{\nu }$_max_/cm^−1^ 3118 (OH), 1622 (C=O), 1602, 1480 (arom); ^1^H NMR (400 MHz, CDCl_3_) δ 9.15 (s, 1H, OH), 7.46 (d, *J* = 7.6 Hz, 1H, H-arom), 7.40 (t, *J* = 7.2 Hz, 1H, H-arom), 7.33 (d, *J* = 7.6 Hz, 1H, H-arom), 7.27 (m, 1H, H-arom), 7.16 (d, *J* = 2.4 Hz, 1H, H-arom), 6.63 (s, 1H, CH), 6.23 (d, *J* = 2.4 Hz, 1H, H-arom), 5.50 (d, *J_1,2_* = 4.8 Hz, 1H, H-1), 5.06 (t, *J_1,2_* = *J_2,3′_* = 4.4 Hz, 1H, H-2), 3.43 (d, *J_3,3′_* = 17.2 Hz, 1H, H-3), 3.28 (dd, *J_2,3′_* = 4.4 Hz, *J_3,3′_* = 17.2 Hz, 1H, H-3′), 2.44 (s, 3H, OCH_3_), 1.41 (s, 9H, CH_3_), 0.91 (s, 9H, CH_3_); ^13^C NMR (100 MHz, CDCl_3_) δ 170.14 (C=O), 151.95 (C-OH), 141.66, 141.17, 139.91, 138.03, 129.36, 127.77, 126.05, 125.26, 124.93, 124.46, 121.25 (C-arom), 86.57 (CH) 82.34 (C2), 66.61 (C1), 37.09 (C3), 35.04, 33.97 (C-(CH_3_)_3_), 31.20, 29.82 (CH_3_), 23.22 (OCH_3_). Anal. Calculated for C_26_H_33_NO_3_: C, 76.62; H, 8.16; N, 3.44. Found: C, 76.37; H, 8.08; N, 3.45.

*(1S,2R)-2-O-Acetyl-1-[(3,5-di-tert-butyl-2-hydroxybenzylidene)amino]-2-indanol***(54).** Following the general procedure from compound **36**: 44%; m.p. 145–147 °C; [α]_D_^20^ + 115.2°; [α]_578_^20^ + 117.9°; [α]_546_^20^ + 132.4° (*c* 0.5, CHCl_3_); IR (KBr) $\bar{\nu }$_max_/cm^−1^ 1739 (C=O), 1627 (C=N), 1595, 1473 (arom); ^1^H NMR (400 MHz, CDCl_3_) δ 13.62 (s, 1H, OH), 8.51 (s, 1H, CH=N), 7.42 (d, *J* = 2.4 Hz, 1H, H-arom), 7.34 (m, 2H, H-arom), 7.28 (m, 1H, H-arom), 7.21 (d, *J* = 7.2 Hz, 1H, H-arom), 7.16 (d, *J* = 2.4 Hz, 1H, H-arom), 5.69 (c, *J_1,2_* = *J_2,3_ = J_2,3′_ =* 5.6 Hz, 1H, H-2), 5.04 (d, *J_1,2_* = 6.0 Hz, 1H, H-1), 3.36 (dd, *J_2,3_* = 6.4 Hz, *J_3,3′_* = 16.4 Hz, 1H, H-3), 2.28 (dd, *J_2,3′_* = 5.2 Hz, *J_3,3′_* = 16.4 Hz, 1H, H-3′), 2.06 (s, 3H, OCH_3_), 1.44 (s, 9H, CH_3_), 1.35 (s, 9H, CH_3_); ^13^C NMR (100 MHz, CDCl_3_) δ 170.97 (C=O), 166.98 (CH=N), 158.29 (C-OH), 140.79, 140.05, 139.85, 136.77, 128.66, 127.34, 127.30, 126.26, 125.06, 124.95, 117.81 (C-arom), 76.05, 73.15 (C1, C2), 36.83 (C3), 35.05, 34.16 (C-(CH_3_)_3_), 31.51, 29.39 (CH_3_), 20.94 (OCH_3_). Anal. Calculated for C_26_H_33_NO_3_: C, 76.62; H, 8.16; N, 3.44. Found: C, 76.39; H, 8.09; N, 3.34.

*N-Acetyl-3,5-di-tert-butyl-2-{(2S,3aS,8aR)-3,3a,8,8a-tetrahydro-2H-indeno [1,2-d]oxazol-2-yl}phenol***(55).** Following the general procedure from compound **36**: 10%; m.p. 166–168 °C; [α]_D_^20^ + 242.8°; [α]_578_^20^ + 255.1°; [α]_546_^20^ + 292.2°; [α]_436_^20^ + 523.5° (*c* 0.5, CHCl_3_); IR (KBr) $\bar{\nu }$_max_/cm^−1^ 3117 (OH), 1623 (C=O), 1602, 1480 (arom); ^1^H NMR (500 MHz, CDCl_3_) δ 9.15 (s, 1H, OH), 7.46 (d, *J* = 7.5 Hz, 1H, H-arom), 7.40 (t, *J* = 7.0 Hz, 1H, H-arom), 7.33 (d, *J* = 7.5 Hz, 1H, H-arom), 7.27 (m, 1H, H-arom), 7.16 (d, *J* = 2.0 Hz, 1H, H-arom), 6.63 (s, 1H, CH), 6.22 (d, *J* = 2.0 Hz, 1H, H-arom), 5.50 (d, *J_1,2_* = 4.5 Hz, 1H, H-1), 5.07 (t, *J_1,2_* = *J_2,3′_* = 4.5 Hz, 1H, H-2), 3.43 (d, *J_3,3′_* =17.5 Hz, 1H, H-3), 3.29 (dd, *J_2,3′_* = 4.0 Hz, *J_3,3′_* = 17.5 Hz, 1H, H-3′), 2.45 (s, 3H, OCH_3_), 1.41 (s, 9H, CH_3_), 0.91 (s, 9H, CH_3_); ^13^C NMR (125 MHz, CDCl_3_) δ 170.26 (C=O), 151.96 (C-OH), 141.65, 141.17, 139.92, 138.03, 129.35, 127.77, 126.04, 125.26, 124.93, 124.44, 121.26 (C-arom), 86.59 (CH) 82.34 (C2), 66.63 (C1), 37.10 (C3), 35.04, 33.97 (C-(CH_3_)_3_), 31.21, 29.83 (CH_3_), 23.21 (OCH_3_).

*(1R,2R)-2-O-Acetyl-1-[(3,5-di-tert-butyl-2-hydroxybenzylidene)amino]-2-indanol***(56).** Following the general procedure from compound **37**: 64%; m.p. 106–108 °C; [α]_D_^25^ − 48.1°; [α]_578_^25^ − 48.5°; [α]_546_^25^ − 51.3°; [α]_436_^25^ − 28.7° (*c* 0.5, CHCl_3_); IR (KBr) $\bar{\nu }$_max_/cm^−1^ 1737 (C=O), 1625 (C=N), 1598, 1460 (arom); ^1^H NMR (500 MHz, CDCl_3_) δ 13.25 (sa, 1H, OH), 8.55 (s, 1H, CH=N), 7.42 (d, *J* = 2.0 Hz, 1H, H-arom), 7.27 (m, 3H, H-arom), 7.16 (m, 2H, H-arom), 5.45 (c, *J_1,2_* = *J_2,3_ = J_2,3′_ =* 6.0 Hz, 1H, H-2), 4.88 (d, *J_1,2_* = 5.5 Hz, 1H, H-1), 3.63 (dd, *J_2,3_* = 7.0 Hz, *J_3,3′_* = 16.0 Hz, 1H, H-3), 2.93 (dd, *J_2,3′_* = 6.0 Hz, *J_3,3′_* = 16.5 Hz, 1H, H-3′), 2.08 (s, 3H, OCH_3_), 1.44 (s, 9H, CH_3_), 1.35 (s, 9H, CH_3_); ^13^C NMR (125 MHz, CDCl_3_) δ 170.81 (C=O), 167.31 (CH=N), 158.08 (C-OH), 140.37, 140.29, 139.49, 136.90, 128.67, 127.50, 127.32, 126.32, 125.04, 124.55, 117.68 (C-arom), 81.19, 77.62 (C1, C2), 37.14 (C3), 35.06, 34.19 (C-(CH_3_)_3_), 31.52, 29.2 (CH_3_), 21.17 (OCH_3_).

## 4. Conclusions

In conclusion, a series of Schiff bases from chiral and conformationally rigid 1-amino-2-indanol enantiomers have been obtained and fully characterized by spectroscopic methods. Crystal data reveal the tautomeric preference and electronic delocalization in the solid phase, as does NMR spectroscopy for structures in solution, which were further assessed by computational analyses. Good correlations have been obtained between tautomeric equilibria and chemical shifts of phenolimine or ketoamine signals, thus revealing the influence of substitution patterns. Reaction of *cis*-amino indanols and salicylaldehyde exhibit a high level of stereocontrol, as disclosed by the formation of a single diastereomeric adduct arising from two aldehyde molecules. All configurations of the stereogenic elements through the oxazolidine–oxazine fragment created could be determined by single-crystal diffraction analysis with structures computed by DFT methods. The geometrical and energy features of intramolecular H-bonding present in such structures have also been discussed. Finally, the mechanism of 1,3-oxazolidine formation and the evaluation of imine/oxazolidine mixtures under acetylating conditions have been investigated by NMR spectroscopy and corroborated by theoretical calculations as well. All data point to interplay between kinetic and thermodynamic control, even if imines constitute the thermodynamically generated structures. It is worth mentioning that cyclization of imines under acylating conditions leads to oxazolidines having a 1,4-*trans* arrangement.

## Figures and Tables

**Table 1:** Comparative bond lengths [a] of **24** and **40**–**41** with average distances [b] of phenoliminic and keto-amino structures.

	Compound					Model Structure [c]	
Bond	24	40	41a	41b	42	Phenolimine	Ketoamine
N1-C2	1.322	1.310	1.285	1.289	1.299	1.287 (1.266, 1.317)	1.327 (1.303, 1.347)
C2-C3	1.422	1.414	1.439	1.433	1.414	1.445 (1.426, 1.457)	1.407 (1.399, 1.413)
C3-C4	1.443	1.433	1.459	1.454	1.437		
C4-O5	1.309	1.298	1.259	1.263	1.281	1.349 (1.323, 1.399)	1.294 (1.279, 1.301)

[a] In Å at 100 K; [b] Determined at different temperatures; [c] Minimal and maximal values in parentheses.

**Table 2:** Selected NMR chemical shifts for **23**–**34** and **37** [a].

Compound	Structure [b]	δ_OH/NH_ [c]	δ_N-CH_ [c]	δ_C-OH/C=O_ [d]	δ_N-CH_ [d]
**23**	**38**	14.49 bs	8.94 s	178.06	166.86
**24**	**24**	13.05 s	8.67 s	154.62	165.19
**25**	**25**	13.92 bs	8.68 s	161.26	164.54
**26**	**26**	14.32 bs	8.69 s	165.89	165.23
**27**	**40**	14.01 bs	8.49 s	168.92	164.03
**28**	**41**	13.86 bs	9.10 d	169.99	167.97
**29**	**29**	13.73 s	8.71 s	161.80	166.24
**30**	**43**	14.50 bs	8.95 s	178.10	166.88
**31**	**31**	13.05 s	8.67 s	154.95	165.53
**32**	**32**	13.91 bs	8.69 s	160.77	164.06
**33**	**44**	14.01 s	8.49 s	168.88	164.03
**34**	**34**	13.74 s	8.71 s	161.41	165.87
**37**	**37**	13.96 s	8.75 s	157.37	167.79

[a] In DMSO-*d*_6_, δ in ppm; [b] Structure in solution; [c] At 500 MHz, bs = broad singlet, s = singlet, d = doublet; [d] At 125 MHz.

**Table 3:** Relative energies calculated in the gas phase (kcal·mol^−1^) [a].

	Imine		TS ^‡^			Enamine	
Compound	ΔE	ΔG	ΔE	ΔG	ṽ ^‡^ [b]	ΔE	ΔG
**24**/**31**	0.00	0.00	8.31	6.24	−966.8	6.28	5.63
**29/34**	0.00	0.00	8.23	5.34	−849.7	6.69	6.51
**38**/**43**	0.00	0.00	6.20	3.85	−961.2	4.25	3.96
**40**/**44**	0.00	0.00	6.75	4.11	−995.3	4.42	4.47
**41**	0.00	0.00	4.07	1.64	−957.6	1.36	1.67

[a] M06-2X/6-311g(d,p); [b] Imaginary frequency of transition state (‡) in cm^−1^.

**Table 4:** Relative energies calculated in DMSO (kcal·mol^−1^) [a].

	Imine		TS ^‡^			Enamine	
Comp.	ΔE	ΔG	ΔE	ΔG	ṽ ^‡^ [b]	ΔE	ΔG
**24/31**	0.00	0.00	7.01	4.68	−919.2	3.24	3.84
**29/34**	0.00	0.00	5.15	3.05	−983.1	2.74	2.89
**38/43**	0.00	0.00	3.13	1.20	−1056.2	−0.57	−0.10
**40/44**	0.00	0.00	4.23	2.50	−1099.9	0.73	1.32
**41**	0.00	0.00	[c]	[c]	[c]	−4.44	−4.05

[a] M06-2X/6-311g(d,p); [b] Imaginary frequency of transition state (‡) in cm^−1^; [c] The transition structure could not be located.

**Table 5:** Empirical and calculated [a] dihedral angles (°) for compound **47**.

Dihedral Angle	X-ray	Gas Phase	EtOH [b]
C15, C19, C18, C17	0.293	−1.820	−1.470
O2, C8, C13, C14	−2.562	−3.216	−2.374
C8, C13, C14, N1	−9.124	−6.554	−7.271
C19, C15, C16, O3	−101.525	−105.189	−103.936
C19, C15, N1, C14	77.957	80.813	79.991
C15, N1, C14, C13	161.424	160.689	160.197
O3, C14, N1, C7	−79.041	−80.842	−80.237
N1, C7, C6, C1	44.052	45.921	46.721
O2, C7, C6, C1	−74.031	−72.306	−71.253

[a] M06-2X/6-311G(d,p); [b] SMD method.

**Table 6:** Empirical and calculated [a] bonding angles (°) for **47**.

Dihedral Angle (θ)	X-ray	Gas Phase	EtOH [b]
C15, N1, C7	112.046	113.041	112.43
C15, N1, C14	101.134	102.003	101.903
C14, N1, C7	110.489	110.413	110.351
Σθ	323.669	325.457	324.684
Pyramidalization	36.331	34.543	35.316

[a] M06-2X/6-311G(d,p); [b] SMD method.

**Table 7:** Calculated relative energies (kcal·mol^−1^) for all diastereomers of **47** [a].

	Chirality				Gas Phase		Ethanol [b]	
Structure	C1 [c]	C2 [c]	N [d]	Axis [e]	ΔE_rel_	ΔG_rel_	ΔE_rel_	ΔG_rel_
**47a**	*R*	*R*	*S*	*aR*	6.68	6.88	5.95	6.25
**47b**	*S*	*R*	*S*	*aR*	0.00	0.00	0.00	0.00
**47c**	*R*	*S*	*S*	*aS*	15.40	15.17	13.70	13.58
**47d**	*S*	*S*	*S*	*aS*	11.71	11.34	8.84	9.08
**47e**	*R*	*R*	*S*	*aS*	12.90	13.45	10.65	10.42
**47f**	*S*	*R*	*S*	*aS*	6.91	6.91	5.49	5.51
**47g**	*R*	*S*	*S*	*aR*	14.02	13.32	13.84	13.99
**47h**	*S*	*S*	*S*	*aR*	12.66	12.26	8.08	8.43
**47i**	*R*	*R*	*R*	*aR*	12.16	13.46	10.65	12.40
**47j**	*S*	*R*	*R*	*aR*	[f]	[f]	16.80	16.04
**47k**	*R*	*S*	*R*	*aS*	10.82	10.56	6.91	8.16
**47l**	*S*	*S*	*R*	*aS*	11.93	11.31	10.39	10.34
**47m**	*R*	*R*	*R*	*aS*	11.66	12.34	11.29	12.33
**47n**	*S*	*R*	*R*	*aS*	18.35	17.53	15.10	14.95
**47o**	*R*	*S*	*R*	*aR*	4.15	4.85	3.15	4.29
**47p**	*S*	*S*	*R*	*aR*	5.93	6.47	6.24	6.03

[a] M06-2X/6-311G(d,p); [b] SMD method; [c] chiral carbons; [d] chiral nitrogen; [e] chirality of the atropisomeric axis. [f] A minimum could not be located; it turned to be structure **47a.**

**Table 8:** Relative energies calculated for **47b** and **49** [a].

	Gas Phase		Ethanol [b]	
	ΔE_rel_	ΔG_rel_	ΔE_rel_	ΔG_rel_
**47b**	0.00	0.00	0.00	0.00
**49**	9.79	8.87	4.18	3.66

[a] M06-2X/6-311G(d,p), in kcal·mol^−1^; [b] Method SMD.

**Table 9:** Relative energies calculated in kcal·mol^−1^ [a].

Structures	Gas phase		Ethanol [b]	
	ΔE_rel_	ΔG_rel_	ΔE_rel_	ΔG_rel_
**20 + 2 × 46 − 2H_2_O**	7.81	1.83	16.03	10.01
**29 + 46 − H_2_O**	5.15	0.17	8.97	3.53
**50a + 46 − H_2_O**	−0.89	−2.42	2.53	0.62
**50b + 46 − H_2_O**	0.65	−1.36	4.69	2.87
**51a + 46 − H_2_O**	0.66	−1.90	5.53	2.49
**51b + 46 − H_2_O**	2.10	−1.15	6.75	3.77
**47a**	6.68	6.88	5.95	6.25
**47b**	0.00	0.00	0.00	0.00

[a] M06-2X/6-311G (d,p); [b] Method SMD.

**Table 10:** Yields and IR absorptions (cm^−1^) of **52**–**56**.

Compound	Yield [a]	Yield [b]	C=O	C=N
**52**	90	45	1739	1627
**53**		11	1622	
**54**	91	44	1739	1627
**55**		10	1623	
**56**	92	64	1737	1625

[a] Crude mixture (%); [b] Purified products (%) after recrystallization.

**Table 11:** Crystal and calculated [a] bond distances (Å) of **52**.

Bond Length	X-ray	Gas Phase	Pyridine [b]
N1-C2	1.288	1.274	1.275
C2-C3	1.451	1.458	1.461
C3-C4	1.420	1.412	1.413
C4-O1	1.365	1.344	1.345
O1-H	0.840	0.995	0.998

[a] M06-2x/6-311G(d,p); [b] SMD method.

**Table 12:** Relative energies for **52**, **53**, **53a** and **57** (kcal·mol^−1^) [a].

	Gas phase		Pyridine [b]	
	ΔE_rel_	ΔG_rel_	ΔE_rel_	ΔG_rel_
**52**	0.00	0.00	0.00	0.00
**53**	−1.23	1.95	−2.43	1.33
**53a**	2.46	6.11	1.48	5.81
**57**	−1.09	3.14	−2.39	2.27

[a] M06-2X/6-311G(d,p); [b] SMD method.

## Data Availability

Crystallographic data have been deposited with the Cambridge Structural Database (CSD) and can be obtained free of charge via www.ccdc.cam.ac.uk/data_request/cif, or by email at: data_request@ccdc.cam.ac.uk, or by contacting the Cambridge Crystallographic Data Centre, 12 Union Road, Cambridge CB2 1EZ, UK.

## Supplementary Materials

The following supporting information can be downloaded at: https://www.mdpi.com/article/10.3390/molecules28041670/s1; Figures and Tables of spectral and computational data as stated through the entire manuscript.

## Sample Availability

Samples of the new compounds synthesized could be available by contacting the authors.
