# Supplementary material for: Schiff Bases and Stereocontrolled Formation of Fused 1,3-Oxazolidines from 1-Amino-2-Indanol: A Systematic Study on Structure and Mechanism
Source: Molecules. 2023 Feb 9;28(4):1670. doi: 10.3390/molecules28041670 (PMC9961571; doi:10.3390/molecules28041670)
Supplement: Supplementary file 1 [file molecules-28-01670-s001.zip › molecules-2139667-supplementary.pdf]

Supplementary Materials

Schiff Bases and Stereocontrolled Formation of Fused 1,3-Oxazolidines from 1-Amino-2-indanol: A Systematic Study on Structure and Mechanism

Esther Matamoros[1,*], Mark E. Light[2], Pedro Cintas[1] and Juan C. Palacios[1,*]

1. Department of Organic and Inorganic Chemistry, Faculty of Sciences and IACYS-Green Chemistry and Sustainable Development Unit, University of Extremadura, 06006 Badajoz, Spain
2. Department of Chemistry, Faculty of Engineering and Physical Sciences, University of Southampton, Southampton SO17 1BJ, UK

* Correspondence: esthermc@unex.es (E.M.); palacios@unex.es (J.C.P.)

ORCID

Esther Matamoros: 0000-0003-4460-2065

Mark E. Light: 0000-0002-0585-0843

Pedro Cintas: 0000-0002-2608-3604

Juan Carlos Palacios: 0000-0002-5004-4744

Summary

Pages	Supporting Data
2	Tables S1-S11
5	Figures S1-S16
9	Solid-State Structures
11	IR Spectra (Fig. S17-S36)
18	NMR Spectra (Fig. S37-S84)
42	Crystallographic Data (Tables S12-S18)
49	Cartesian Coordinates and Calculated Energies at the M06-2X/6-311G(d,p) Level in Gas Phase, Ethanol, Pyridine and DMSO (SMD Model).

Table S1. Yields, IR and Raman absorptions of **23**-**37**.

Comp.	Struct.[a]	Yield[b]	OH/NH[c]	C=O/C=N[c]	C=C[c]	C=O/C=N[d]	C=C[d]
**23**	**38**	91	3174	1655	1611	1655	1603
**24**	**24**	75	3362	1647	1623,1588	1639	1619
**25**	**25**	51	3330	1648	1627,1605	1638	1625
**26**	**39**	62	3174	1642	1597	1641	--
**27**	**40**	86	3182	1644	1616	1638	1608
**28**	**41**	80	3407,3350	1650	1616	1666	--
**29**	**42**	28[e]	3015	1634	1607	1635	--
**30**	**43**	85	3209	1656	1610	1655	1603
**31**	**31**	81	3332	1645	1624,1588	1639	1619
**32**	**32**	59	3329	1648	1627,1605	1638	1625
**33**	**44**	88	3186	1644	1615	1638	1608
**34**	**45**	35	3119	1639	1611	1635	--
**37**	**37**	78[e]	3339	1627	1596	1622	1591

[a] Structure in solid state; [b] in %; [c] IR in cm^{-1}; [d] Raman in cm^{-1}; [e] Yield in ether.

Table S2. Intramolecular hydrogen bonding of compounds **24** and **40**-**41**.

Comp.	***D*-H···*A***	***d*(*D*-H)**[a]	***d*(H···*A*)**[a]	***d*(*D*···*A*)**[a]	**∠(*D*HA)**[b]
**24**	O-H···N	0.841	1.930	2.633	140.22
**40**	N-H···O	0.962	1.833	2.597	134.31
**41a**	N-H···O	0.844	1.910	2.584	136.01
**41b**	N-H···O	0.902	1.843	2.599	139.95
**42**	O-H···N	0.840	1.802	2.563	149.78

[a] In Å; [b] in º.

Tabla S3. ^{1}H and ^{13}C NMR spectroscopic data of **23**-**29**.[a]

Comp.	σ_{OH}	σ_{NH}	σ_{ef}[b]	δ_{CN}	δ_{OH}	δ_{C2}	δ_{CH}	K_T[c]	log K_T[c]	K_T[d]	log K_T[d]
**23**	1.27[e]	0.71	0.56	166.86	14.49	178.06	8.94	∞	--	12.29	1.09
**24**	-0.28	0.10	-0.38	165.19	13.05	154.62	8.67	0.00	∞	0.10	-0.99
**25**	0.26	0.37	-0.11	164.54	13.92	161.26	8.68	0.40	-0.40	0.49	-0.31
**26**	0.37	0.26	0.11	165.33	14.32	165.89	8.69	0.93	-0.03	0.97	-0.01
**27**	0.10	-0.28	0.38	164.03	14.01	168.92	8.49	1.56	0.28	1.77	0.25
**28**	2.51	1.98	0.53	167.97	13.86	169.99	9.10	1.90	-0.35	0.53	-0.27
**29**	0.00	0.00	0.00	166.24	13.73	161.80	8.71	0.44	0.19	1.50	0.18

[a] In DMSO-d_6; [b] $\sigma_{ef} = \sigma_{OH} - \sigma_{NH}$; [c] δ_e = 178.06 ppm, δ_i = 154.62 ppm; [d] δ_e = 180.12 ppm, δ_i = 152.00 ppm; [e] σ^{-}_{OH}.

Table S4. Geometric parameters of solid-state hydrogen bond of **47**.

Comp.	***D*-H···*A***	***d*(*D*-H)**[a]	***d*(H···*A*)**[a]	***d*(*D*···*A*)**[a]	**∠(*D*HA)**[b]
**47**	O-H···N	0.892	1.939	2.736	148.3

[a] In Å; [b] in º.

Table S5. Geometric parameters of hydrogen bonds for the diastereoisomers of **47**.[a]

Comp.	D-H···A	d(D-H)[b]	d(H···A)[b]	d(D···A)[b]	<(DHA)[c]
**47a**	O1-H···N1	0.972	2.021	2.837	140.266
**47b**	O1-H···N1	0.975	1.937	2.761	140.713
**47e**	O1-H···O4	0.966	1.981	2.786	139.475
**47f**	O1-H···O2	0.965	2.144	2.902	134.450
**47g**	O1-H···O2	0.964	1.989	2.777	137.494
**47h**	O1-H···O2	0.969	1.812	2.704	154.670
**47i**	O1-H···O2	0.973	1.743	2.707	170.329
**47l**	O1-H···O2	0.965	2.176	2.933	134.339
**47m**	O1-H···O2	0.965	2.055	2.840	137.128
**47o**	O1-H···N1	0.973	1.952	2.789	142.700
**47p**	O1-H···N1	0.974	1.935	2.739	138.125

[a] M06-2X/6-311G(d,p) in gas 'phase; [b] In º; [c] In Å.

Table S6. Geometric parameters hydrogen bonds for the diastereomers of **47**.[a]

Comp.	D-H···A	d(D-H)[b]	d(H···A)[b]	d(D···A)[b]	<(DHA)[c]
**47a**	O1-H···N1	0.977	1.971	2.806	141.966
**47b**	O1-H···N1	0.978	1.929	2.770	142.560
**47e**	O1-H···O1	0.968	1.971	2.775	139.004
**47f**	O1-H···O1	0.966	2.253	2.977	130.915
**47g**	O1-H···O2	0.965	2.113	2.825	129.361
**47h**	O1-H···O3	0.979	1.752	2.700	161.987
**47i**	O1-H···O3	0.981	1.692	2.668	172.611
**47m**	O1-H···O2	0.964	2.823	3.448	123.274
**47o**	O1-H···N1	1.914	1.914	2.770	144.556
**47p**	O1-H···N1	0.979	1.908	2.740	141.241

[a] M06-2X/6-311G(d,p) in ethanol; [b] In º; [c] In Å.

Table S7. Geometric data of hydrogen bonds.[a]

Comp.	D-H···A	d(D-H)	d(H···A)	d(D···A)	<(DHA)
**29**[b]	O1-H···N1	0.984	1.767	2.642	146.209
**50**[b]	O1-H···N1	0.977	1.839	2.711	147.034
**50b**[b]	O1-H···O2	0.969	1.815	2.661	144.120
**51**[b]	O1-H···N1	0.971	2.000	2.821	140.886
**51b**[b]	O1-H···O2	0.968	1.803	2.643	143.207
**29**[c]	O1-H···N1	0.992	1.728	2.618	147.212
**50**[c]	O1-H···N1	0.987	1.788	2.677	148.096
**50b**[c]	O1-H···O2	0.974	1.787	2.642	144.718
**51**[c]	O1-H···N1	0.976	1.978	2.806	141.171
**51b**[c]	O1-H···O2	0.972	1.840	2.666	141.027

[a] M06-2X/6-311G(d,p); [b] In gas phase; [c] in ethanol (SMD method).

Table S8. Selection of ^{1}H NMR chemical shifts of **52-56**.[a]

Comp.	OH	CH=N	C2-H	C1`-H	C2´-H	C3´-H_2	OAc	NAc
**52**	13.64	8.52		5.04	5.68	3.32	2.07	
**53**	9.15		6.63	5.50	5.07	3.35		2.44
**54**	13.62	8.51		5.04	5.69	3.32	2.06	
**55**	9.15		6.63	5.50	5.07	3.36		2.45
**56**	13.25	8.55		4.88	5.45	3.63, 2.93	2.08	

[a] In Cl_3CD at 400 MHz.

Table S9. Selection of ^{13}C NMR chemical shifts of **52-56**.[a]

Comp.	C=O	C=N	C2	C1´	C2´	C3´	OAc	NAc
**52**	171.01	167.00		73.17	76.06	36.84	20.97	
**53**	170.14		86.57	66.61	82.34	37.09		23.22
**54**	170.97	166.98		73.15	76.05	36.83	20.94	
**55**	170.26		86.59	66.63	82.34	37.10		23.21
**56**	170.81	167.31		77.61	81.19	37.14	21.17	

[a] In Cl_3CD at 100 MHz.

Table S10. Geometric parameters of intramolecular hydrogen bonds of **52** and **53**.[a]

Comp.	*D*-H···*A*	*d*(*D*-H)[b]	*d*(H···*A*)[b]	*d*(*D*···*A*)[b]	∠(*D*HA)[c]
**52**	O-H···N	0.840	1.822	2.577	148.68
**53**	O-H···O	0.840	1.877	2.661	154.73

[a] In solid state; [b] In Å; [c] In º

Table S11. Geometric parameters of hydrogen bonds of **52**, **53** and **53a**.

Comp.	*D*-H···*A*	*d*(*D*-H)[a]	*d*(H···*A*)[a]	*d*(*D*···*A*)[a]	∠(*D*HA)[b]
**52**[a]	O-H···N	0.995	1.674	2.578	148.857
**52**[b]	O-H···N	0.998	1.657	2.570	149.791
**53**[a]	O-H···O	0.977	1.751	2.710	166.297
**53**[b]	O-H···O	0.983	1.683	2.653	168.420
**53a**[a]	O-H···O	0.968	1.779	2.642	146.719
**53a**[b]	O-H···O	0.971	1.747	2.626	148.781

[a]Gas phase; [b] Pyridine.

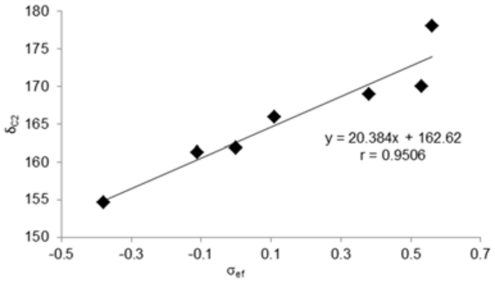

Figure S1.

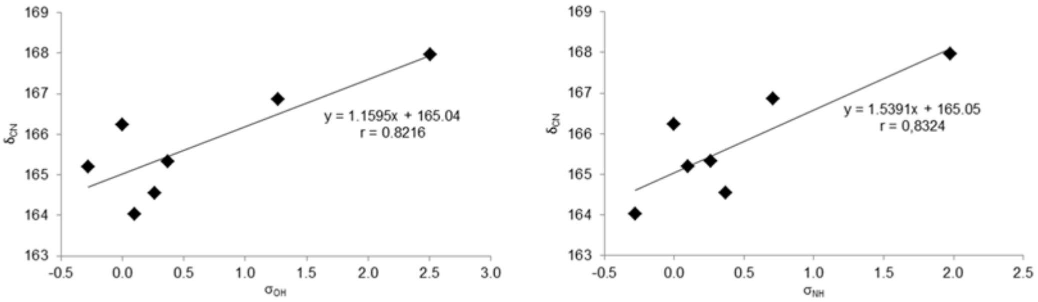

a) b)

Figure S2.

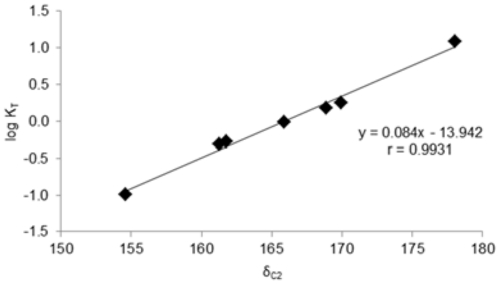

Figure S3. (δ_e = 180.12 ppm, δ_i = 152.00 ppm).

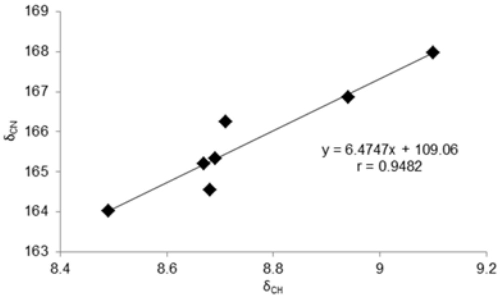

Figure S4.

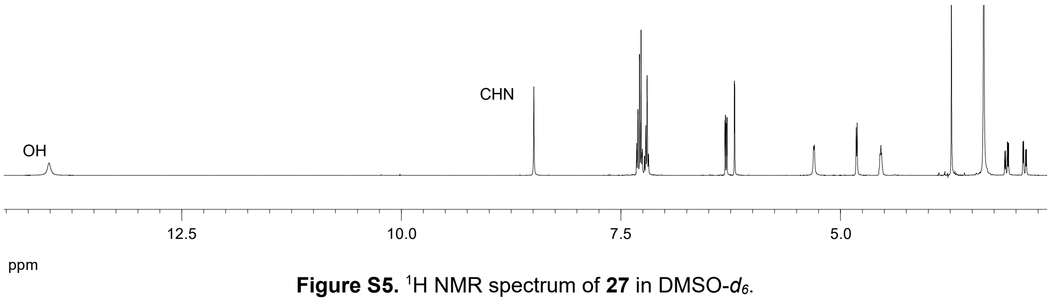

Figure S5. ^{1}H NMR spectrum of **27** in DMSO-d_6.

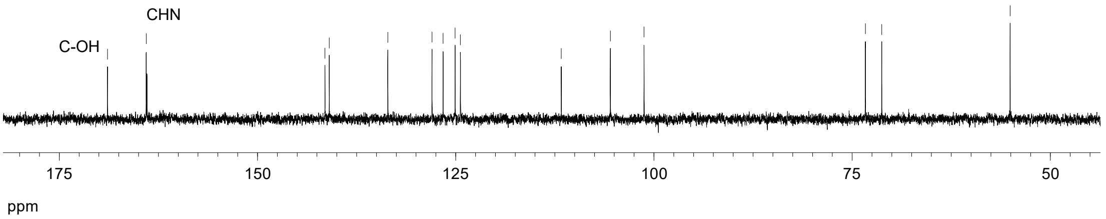

Figure S6. ^{13}C NMR spectrum of **27** in DMSO-d_6.

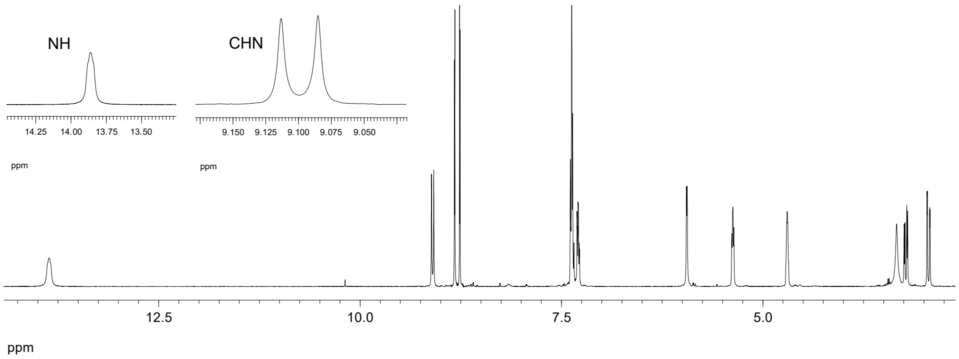

Figure S7. ^{1}H NMR spectrum of **41** in DMSO-d_6.

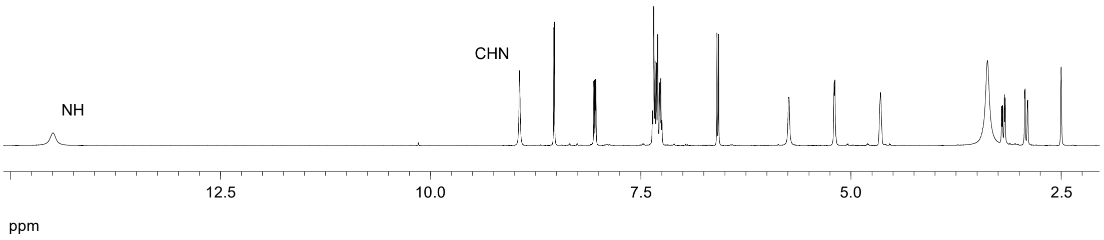

Figure S8. ^{1}H NMR spectrum of **38** in DMSO-d_6.

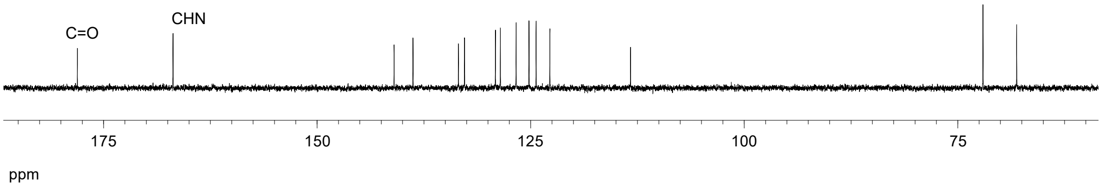

Figure S9. ^{13}C NMR spectrum of **38** in DMSO-d_6.

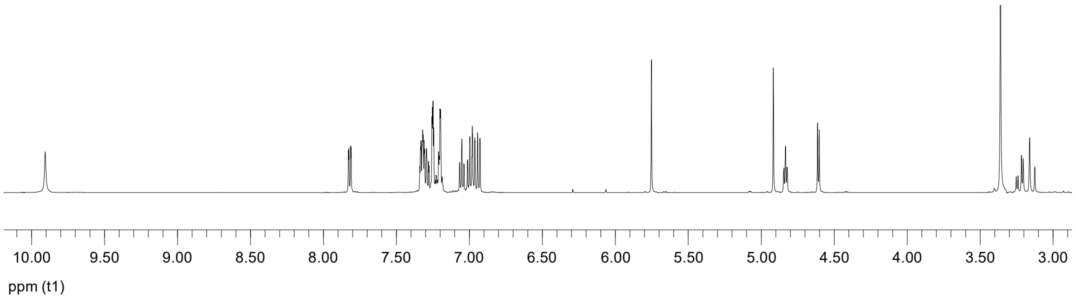

Figure S10. ^{1}H NMR spectrum of **47** in DMSO-d$_6$.

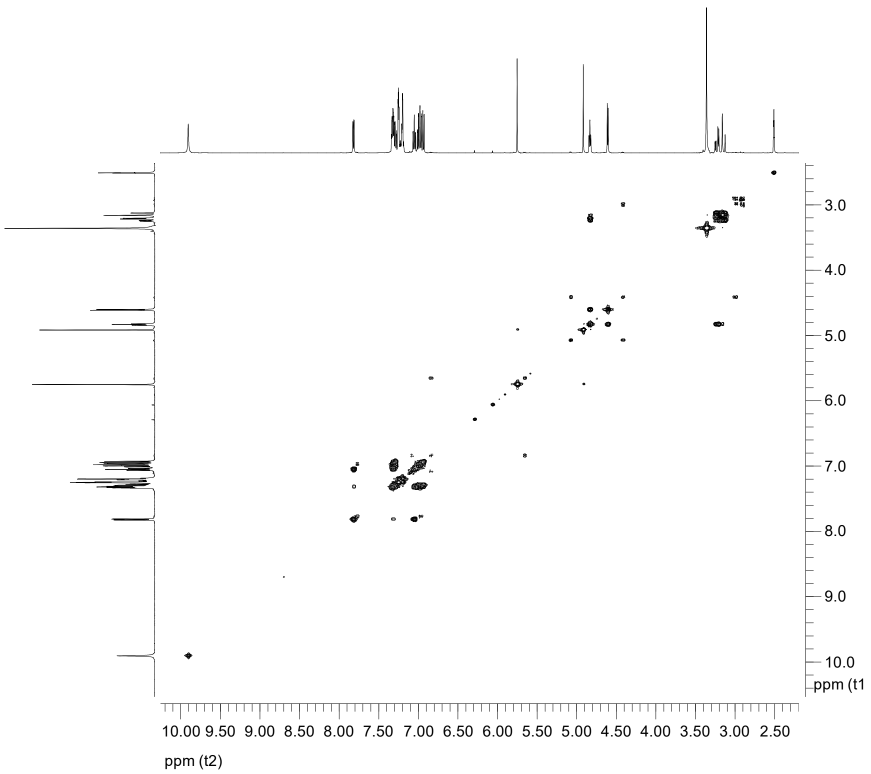

Figure S11. COSY spectrum of **47** in DMSO-*d*$_6$.

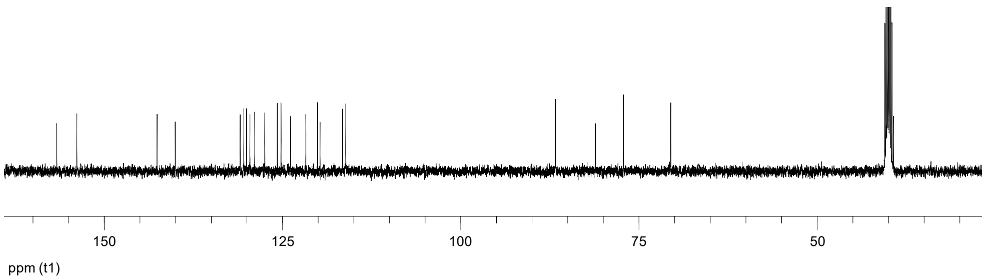

Figure S12. ^{13}C NMR spectrum of **47** in DMSO-*d*$_6$.

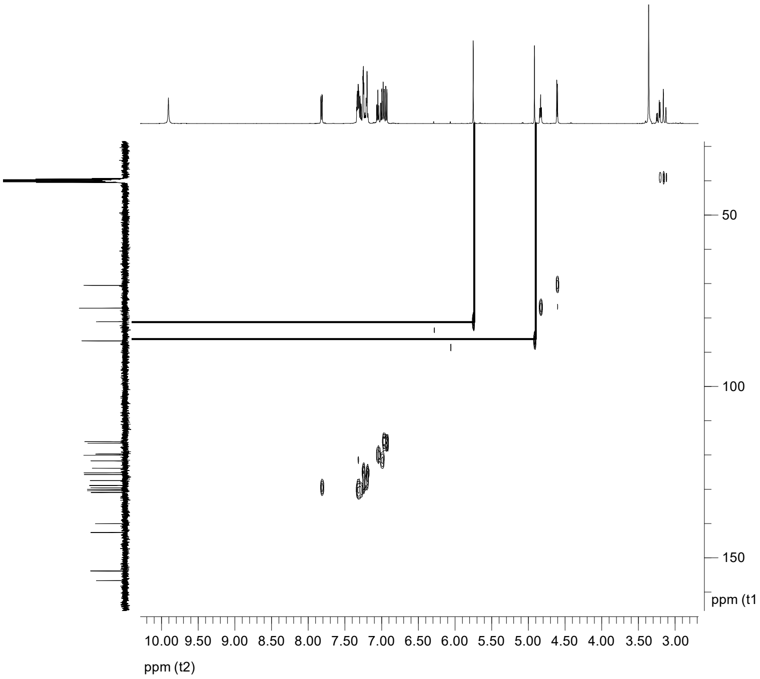

Figure S13. HMQC spectrum of **47** in DMSO-d_6.

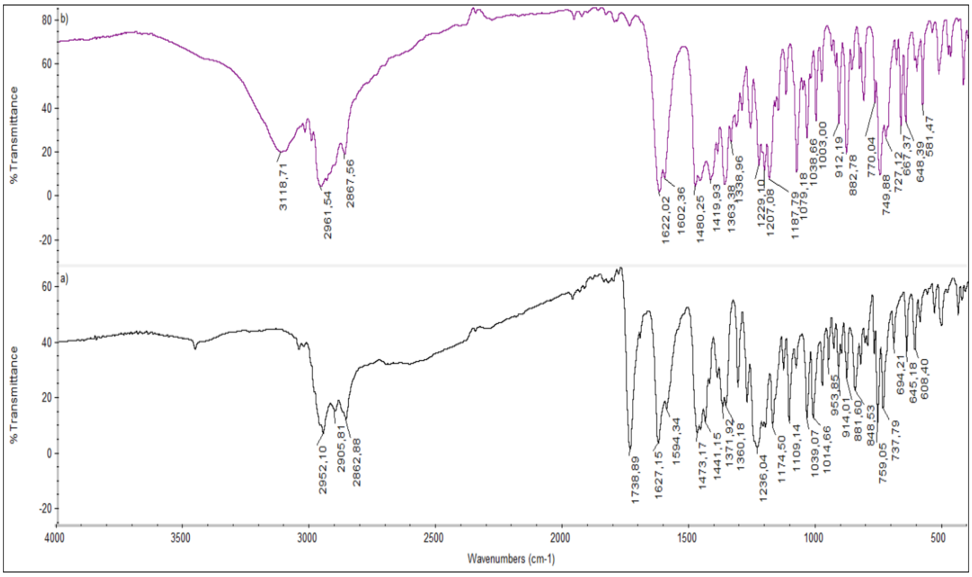

Figure S14 IR spectra of: a) imine **52**, b) oxazolidine **53**.

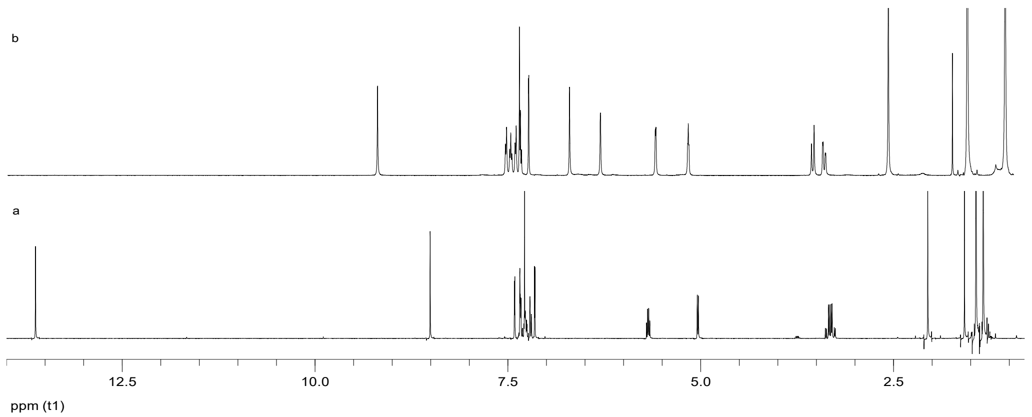

Figure S15. ^{1}H NMR spectra in Cl_3CD of: a) imine **52**, b) oxazolidine **53**.

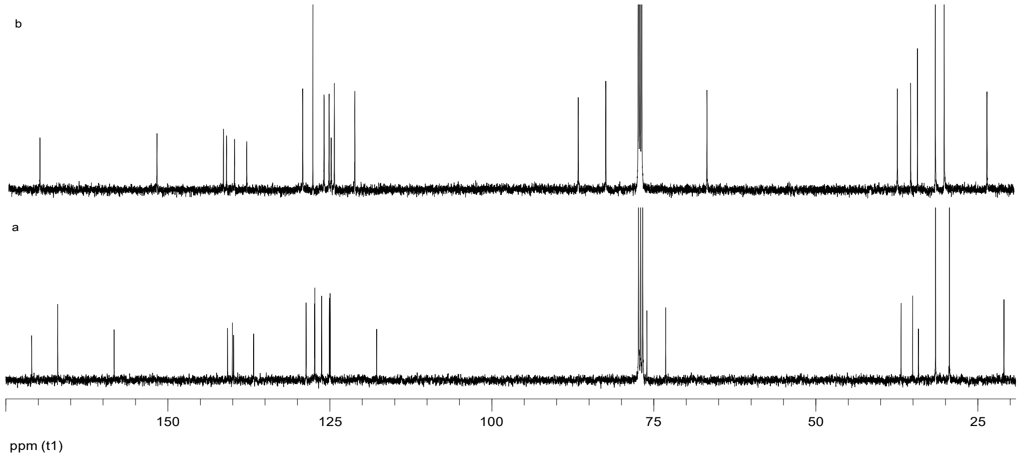

Figure S16. ^{13}C NMR spectra in Cl_3CD of: a) imine **52**, b) oxazolidine **53**.

Solid-state Structures

Solid-state structures can be inferred from FT-IR spectroscopy, although unequivocal assignments are difficult, owing to the fact that the streching vibration of the C=N bond of imines often overlaps with the C=O absorption band of enamines. However, the former shows usually a lower intensity than the carbonyl absorption due to their different polarity. In any case, peak intensity for comparative purposes should be taken with caution. In enamine tautomers, a second absorption can usually be observed at lower frequency and having a similar intensity to the first peak, arising from the streching vibration of the C=C bond, with which the carbonyl is conjugated. In contrast, one or two weak signals, coming from the benzene breathing vibration appear in iminic structures.

Raman spectra have also been recorded to complement the IR spectral data, although peaks attributable to the C=N, C=O or C=C bonds appear approximately in the same position. Table S1 shows the absorption maxima found for the new compounds, in both FT-IR and Raman spectra. A conclusive statement of these vibrational spectra is that the actual solid-state structures for compounds **23**, **26-30**, **33** and **34** correspond to enamine tautomers **38-45**, respectively.

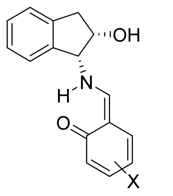

38 X = 5-NO_2
39 X = 4-Br
40 X = 4-OMe
41 X = 3,5-diNO_2
42 X = H

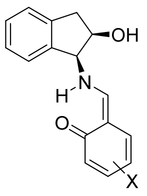

43 X = 5-NO_2
44 X = 4-OMe
45 X = H

As representative examples, IR spectra for each type of tautomeric structure are briefly assessed. Figure S22 shows the IR spectrum of enamine **38** and its enantiomer **43**. Two bands of similar intensity appear at 1655 cm^{-1} and 1611 cm^{-1}, which can be assigned to typical enamine bands. They are intense bands, much more than the streching vibration bands of the CO bonds at 1097 cm^{-1} and 1045 cm^{-1}. Only the asymmetric and symmetric stretching vibrations of the NO_2 group at ~1544 cm^{-1} and ~1330 cm^{-1} respectively, exhibit comparable or superior intensity. The spectrum of dinitro derivative **41** is similar showing weaker enamine bands at 1651 cm^{-1} and 1616 cm^{-1}; in part overshadowed by the stronger coupled absorptions of the two nitro groups. On the other hand, the IR spectrum of **37** shows low-intensity bands attributable to the C=N bond at ~1627 cm^{-1} and the CO bond at ~1085 cm^{-1} (Figure S21).

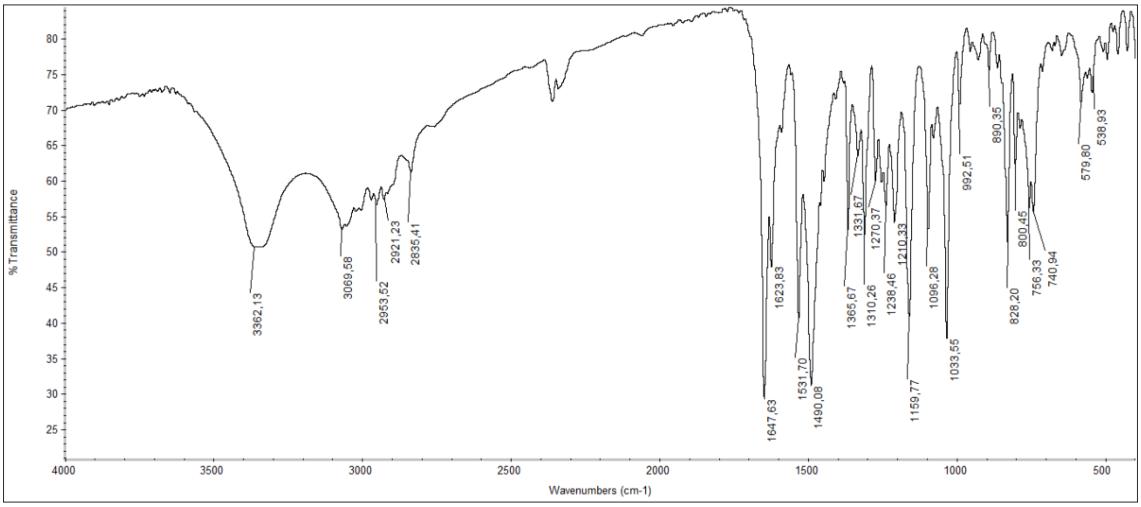

Figure S17. IR spectrum of **24**.

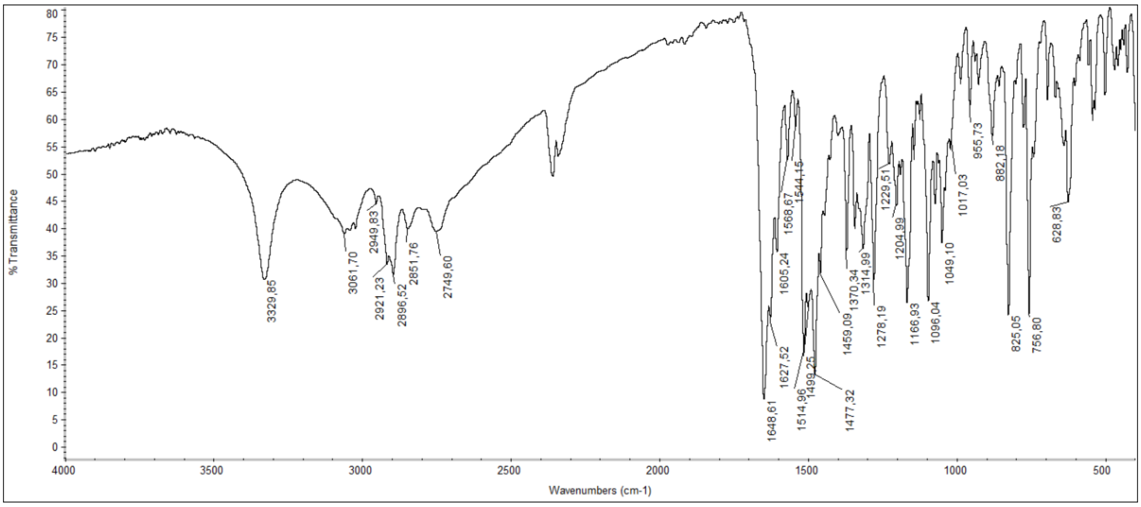

Figure S18. IR spectrum of **25**.

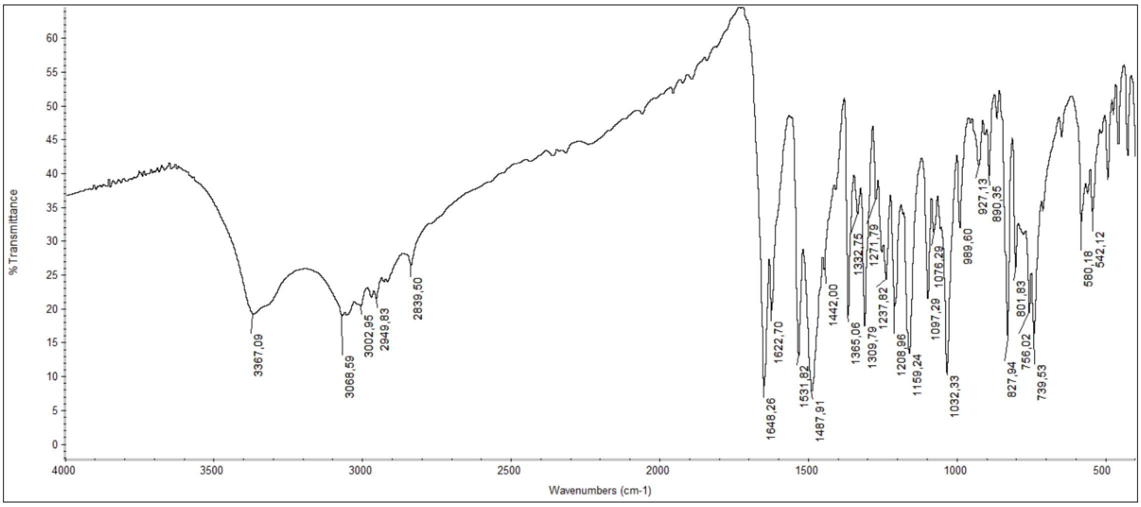

Figure S19. IR spectrum of **31**.

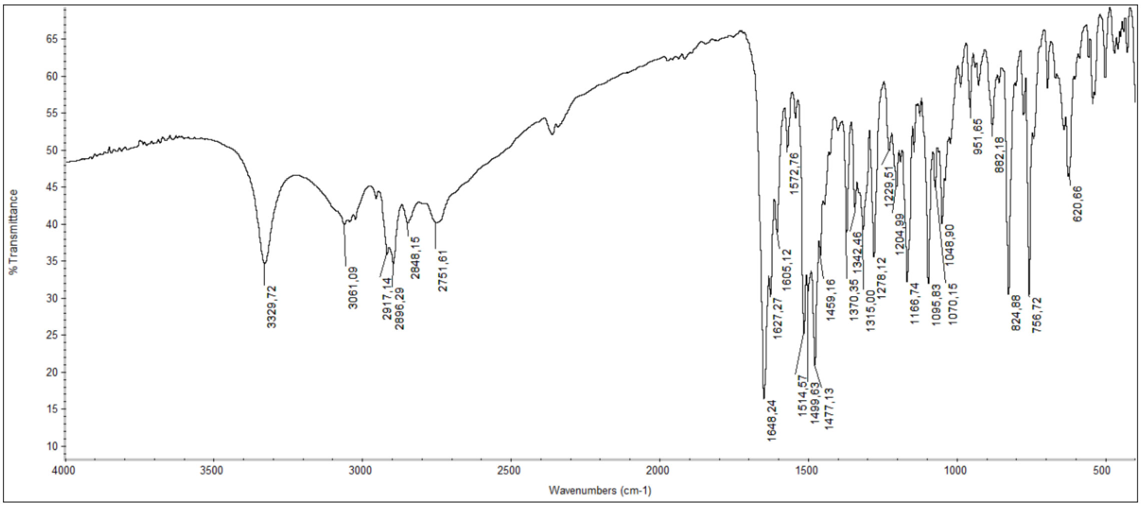

Figure S20. IR spectrum of **32**.

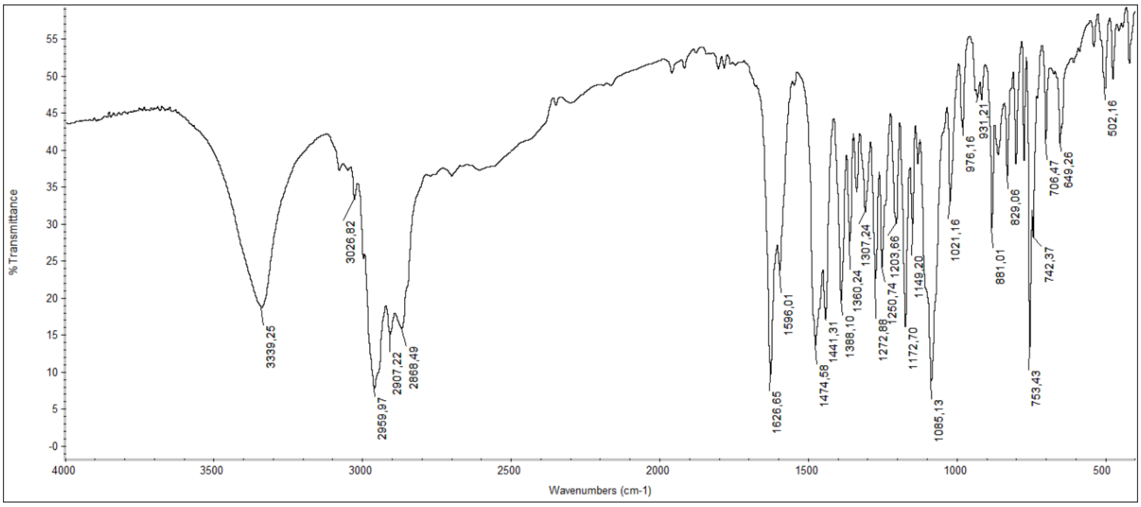

Figure S21. IR spectrum of **37**.

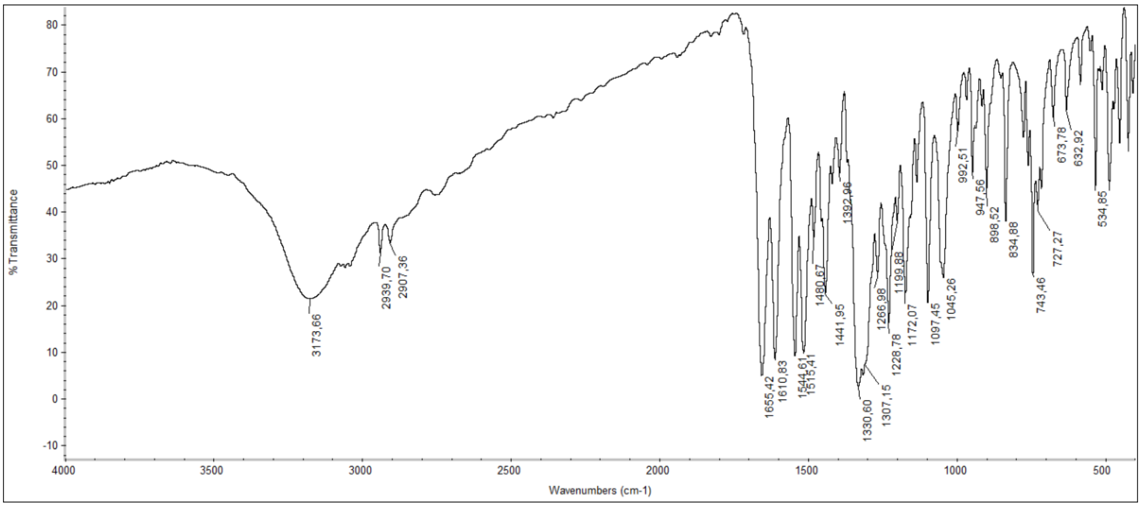

Figure S22. IR spectrum of **38**.

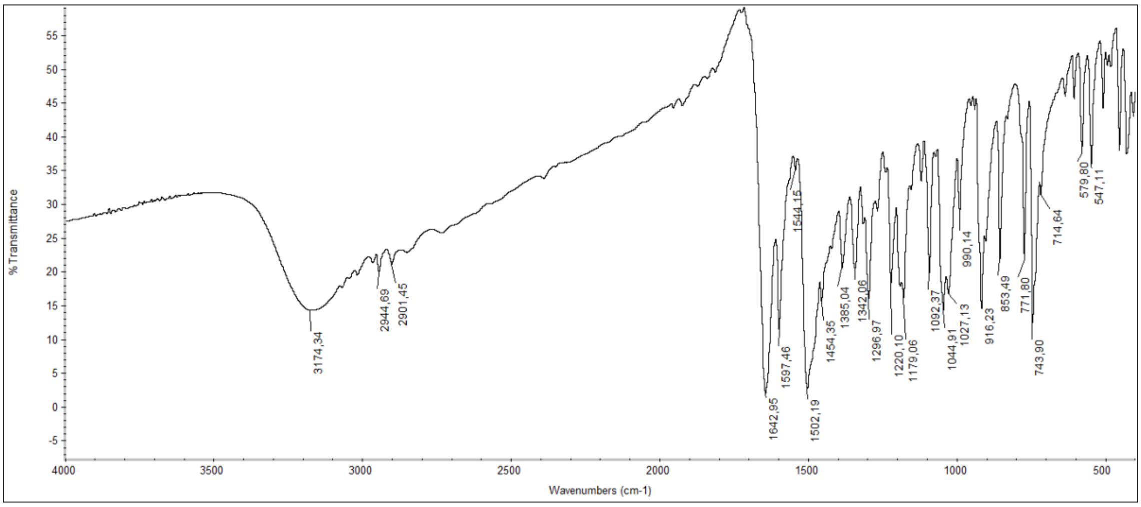

Figure S23. IR spectrum of **39**.

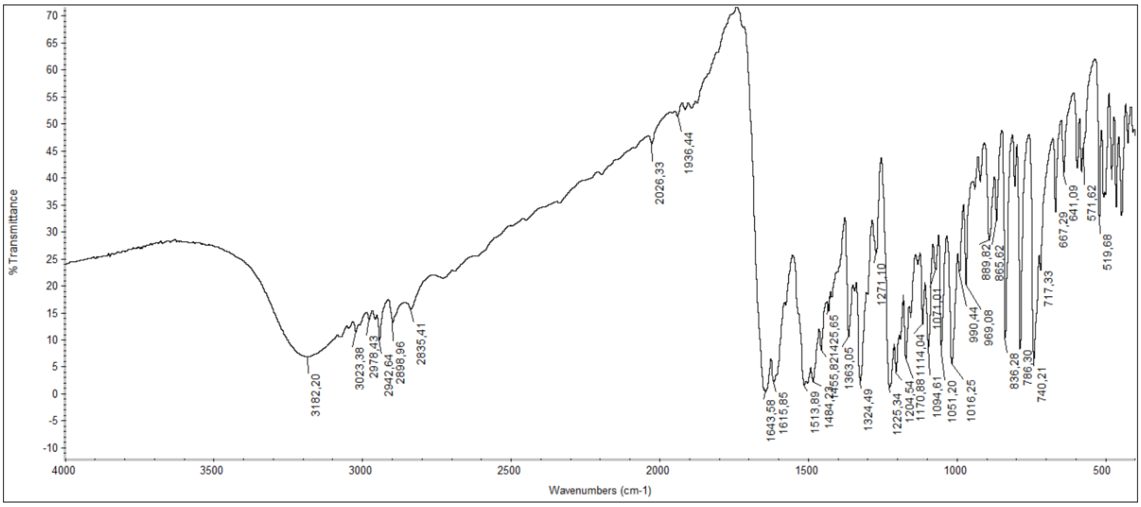

Figure S24. IR spectrum of **40**.

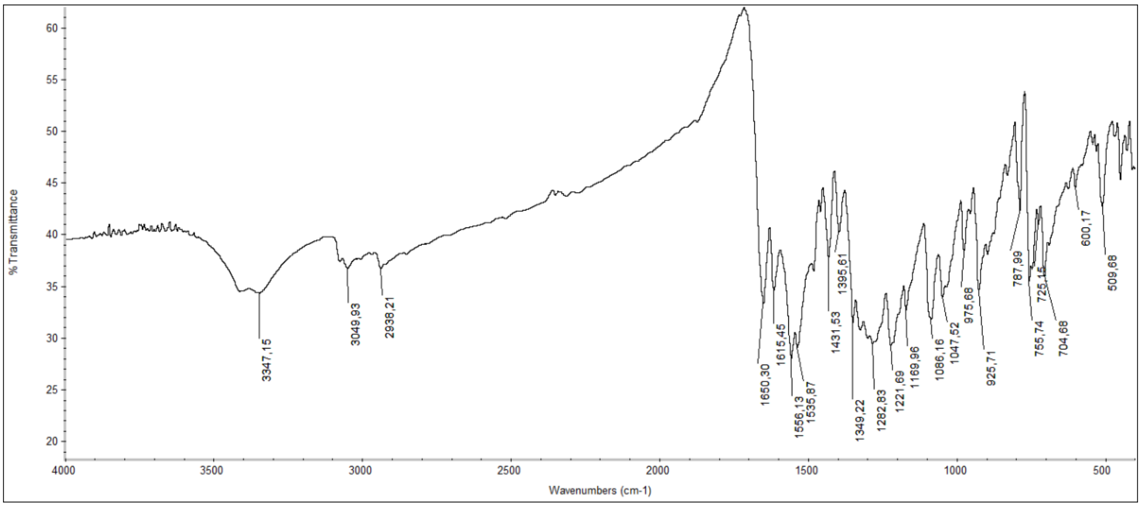

Figure S25. IR spectrum of **41**.

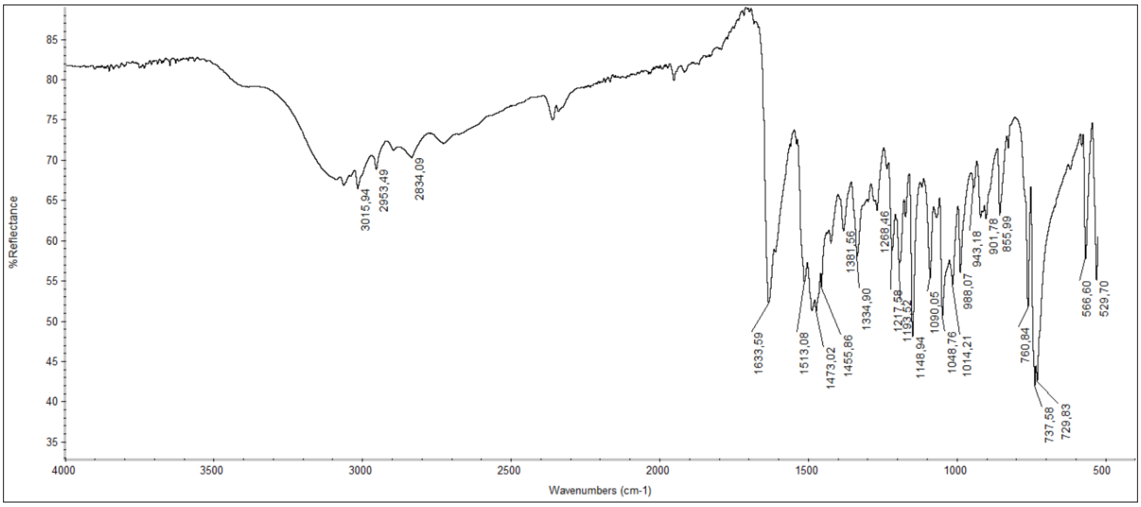

Figure S26. IR spectrum of **29** (**42**).

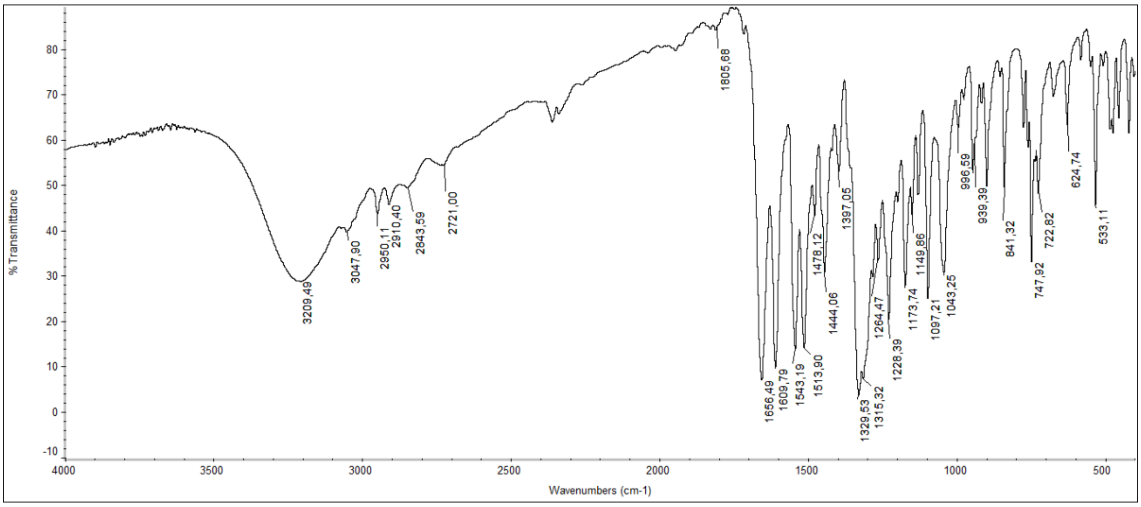

Figure S27. IR spectrum of **43**.

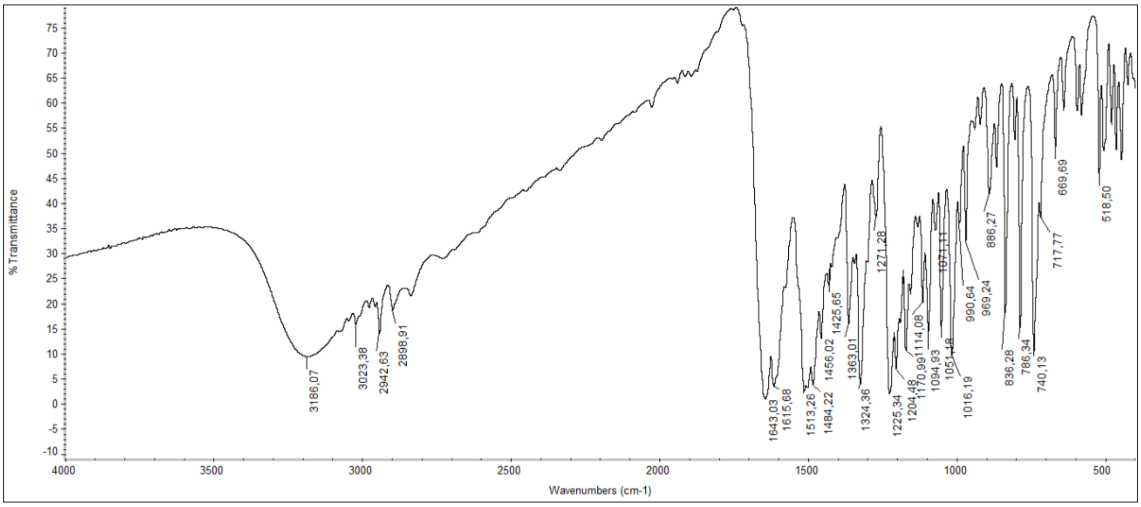

Figure S28. IR spectrum of **44.**

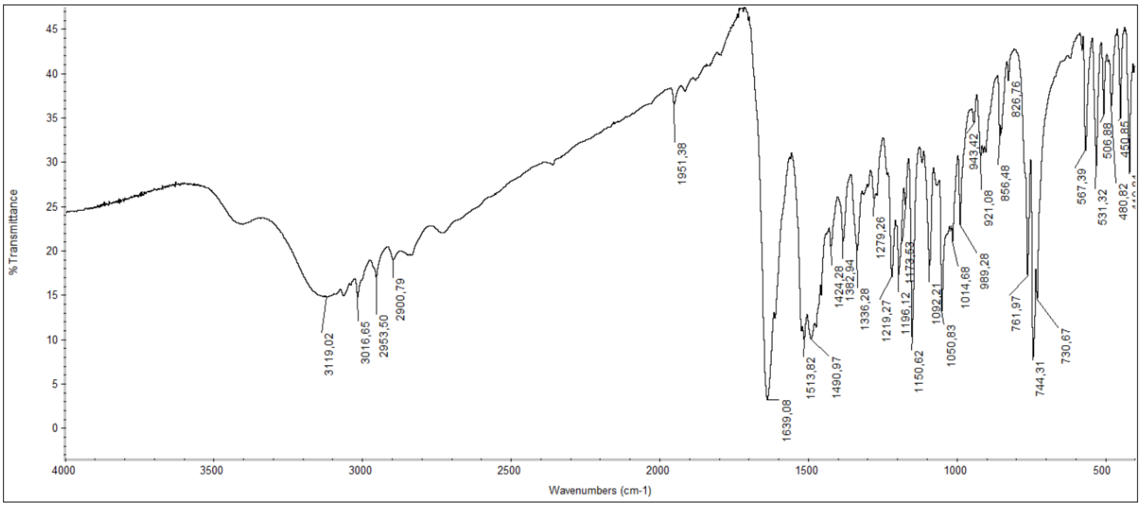

Figure S29. IR spectrum of **34** (**45**).

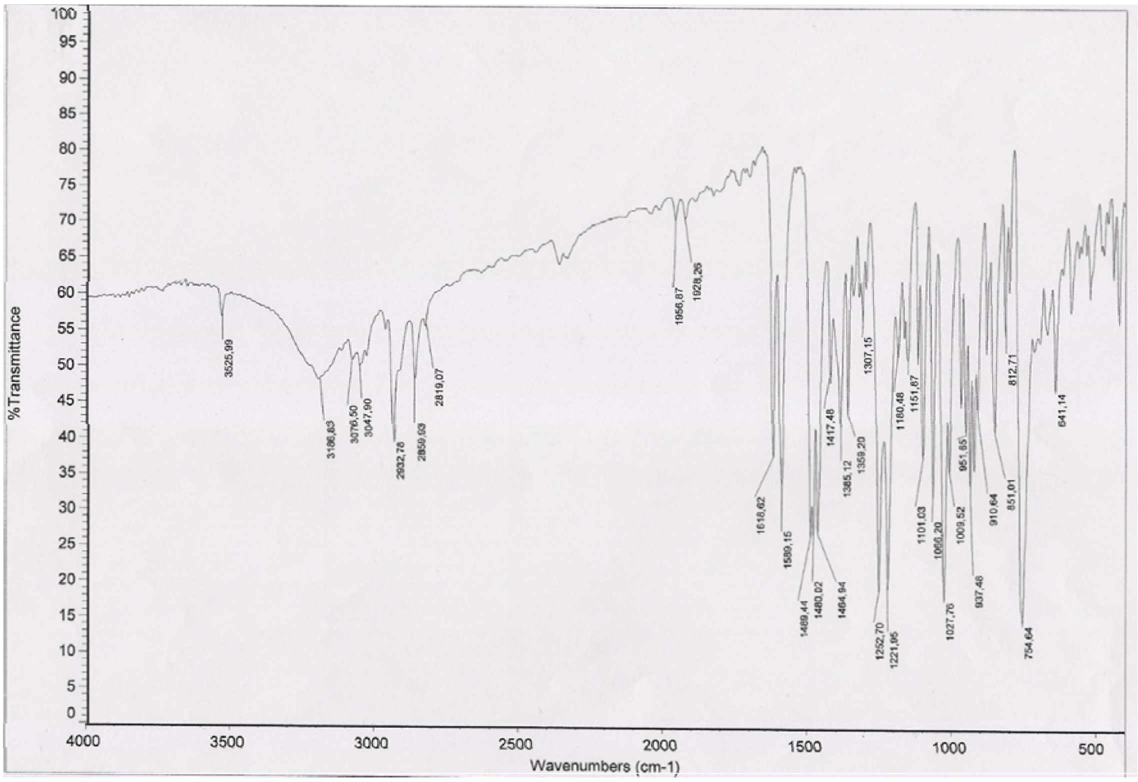

Figure S30. IR spectrum of **47**.

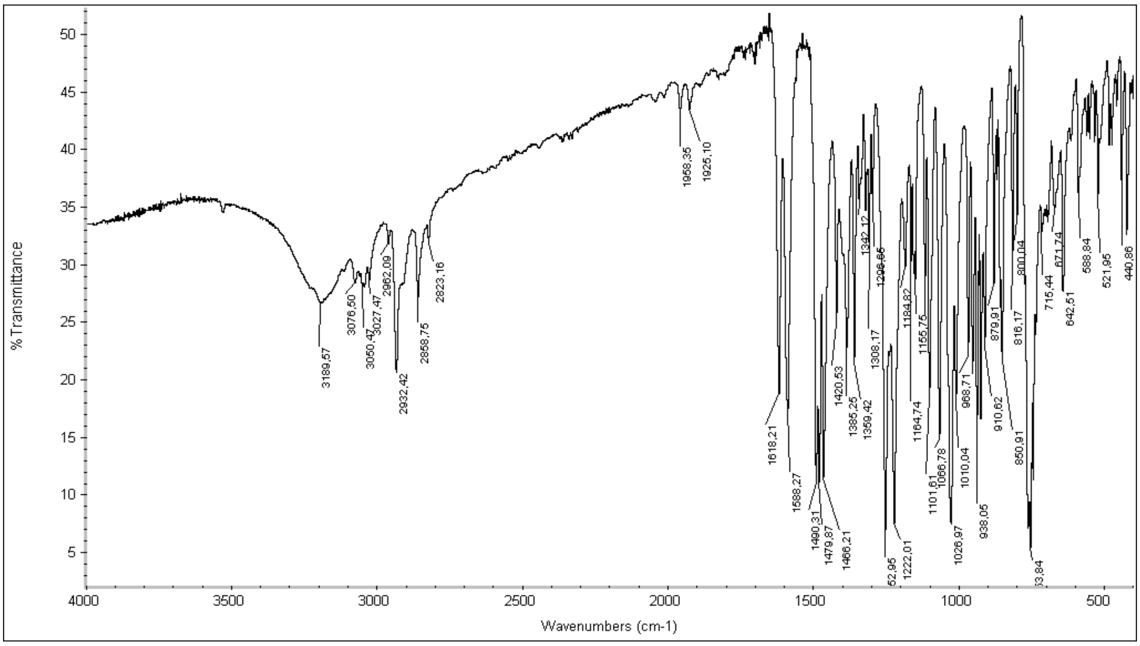

Figure S31. IR spectrum of **48**.

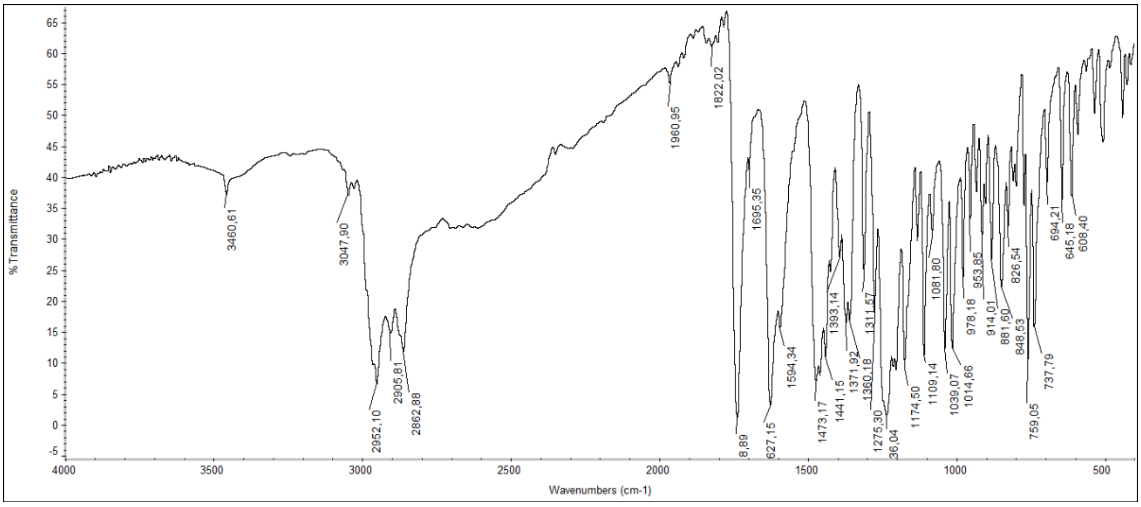

Figure S32. IR spectrum of **52**.

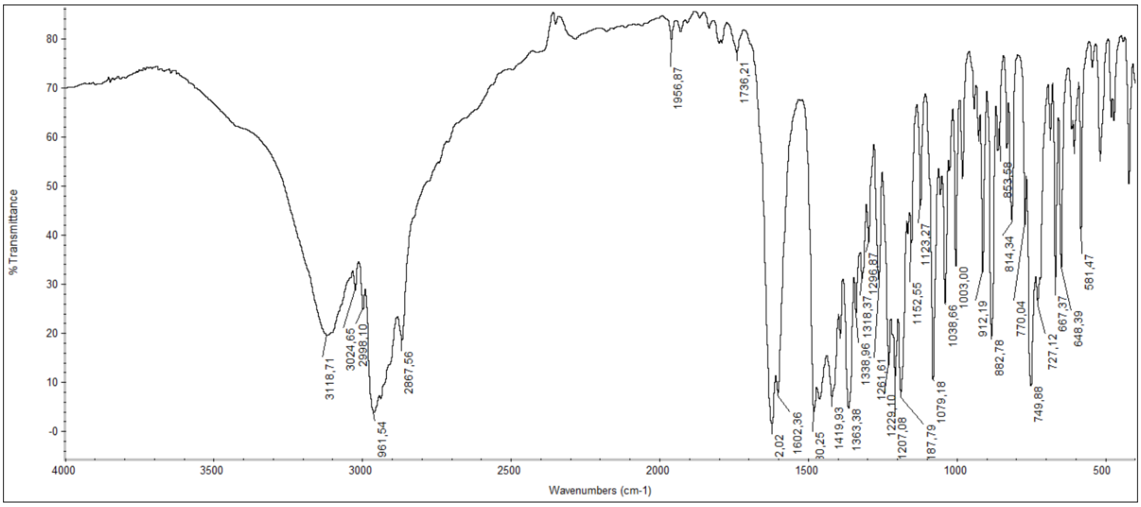

Figure S33. IR spectrum of **53**.

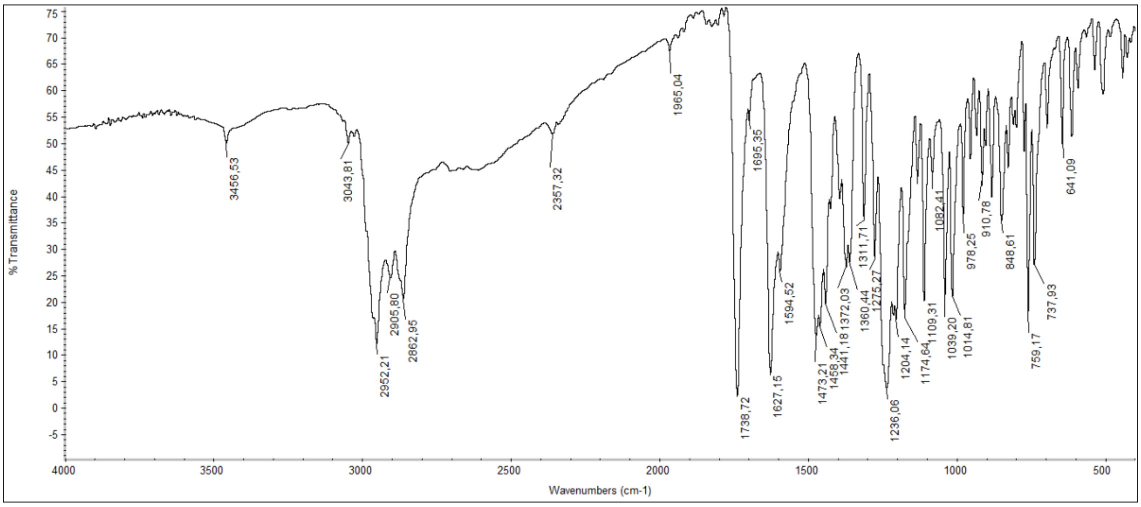

Figure S34. IR spectrum of **54**.

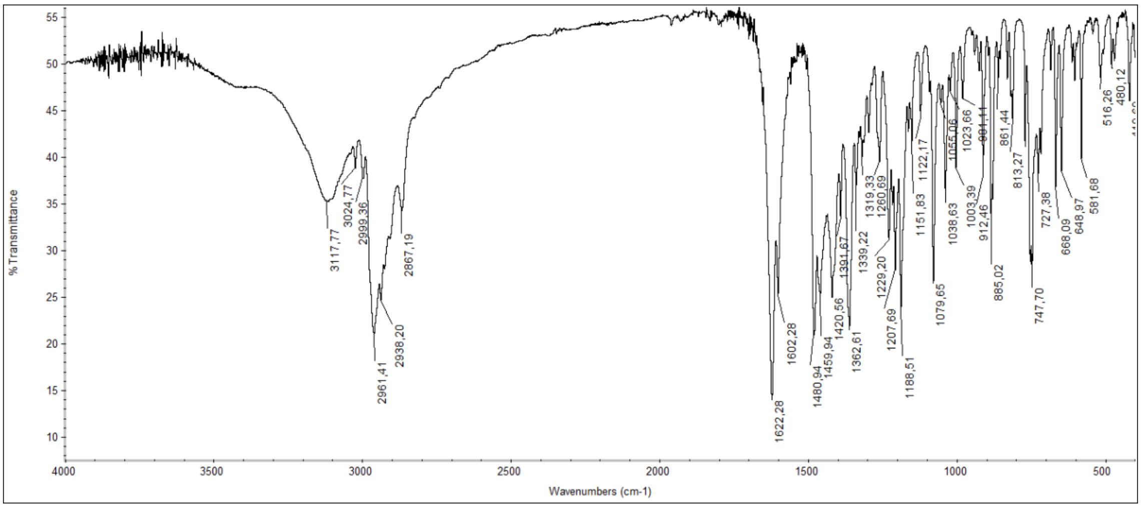

Figure S35. IR spectrum of **55**.

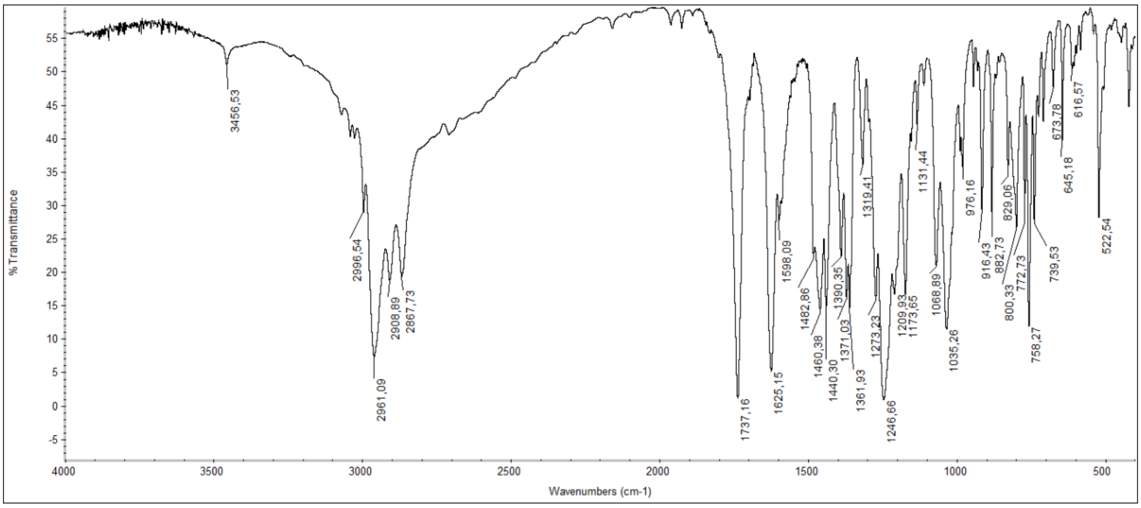

Figure S36. IR spectrum of **56**.

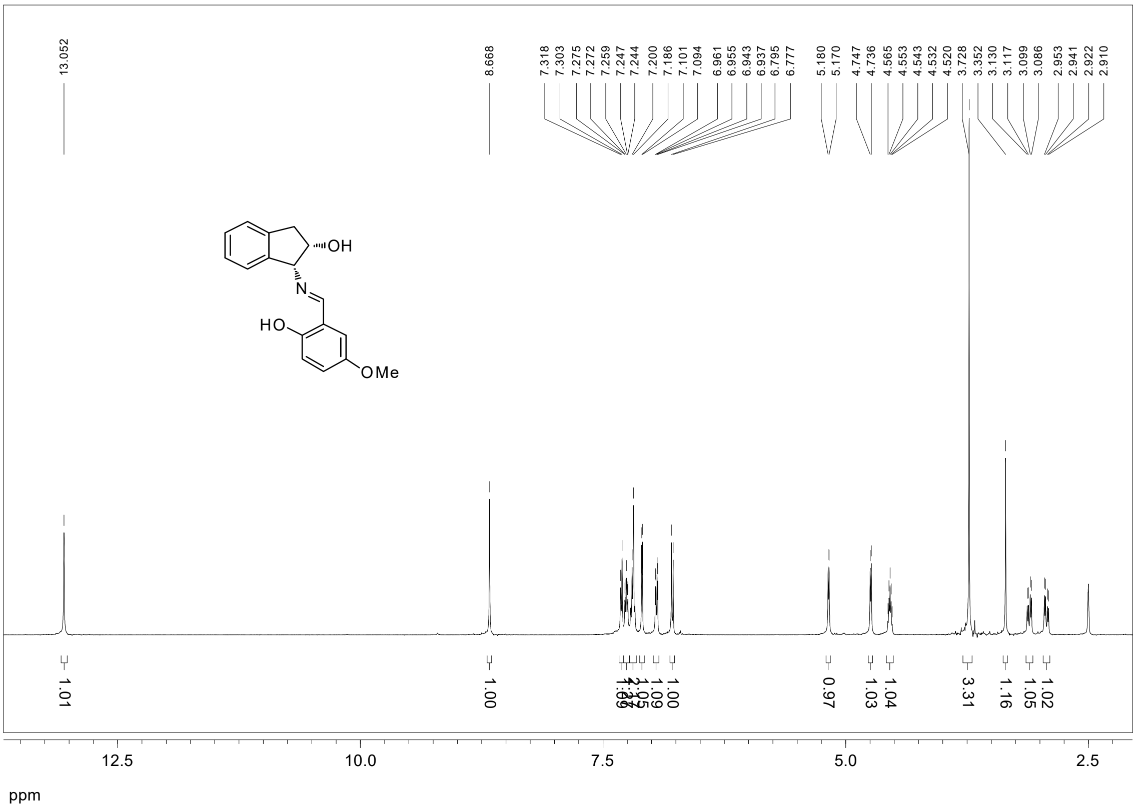

Figure S37. ^{1}H NMR spectrum of **24** in DMSO-d_6.

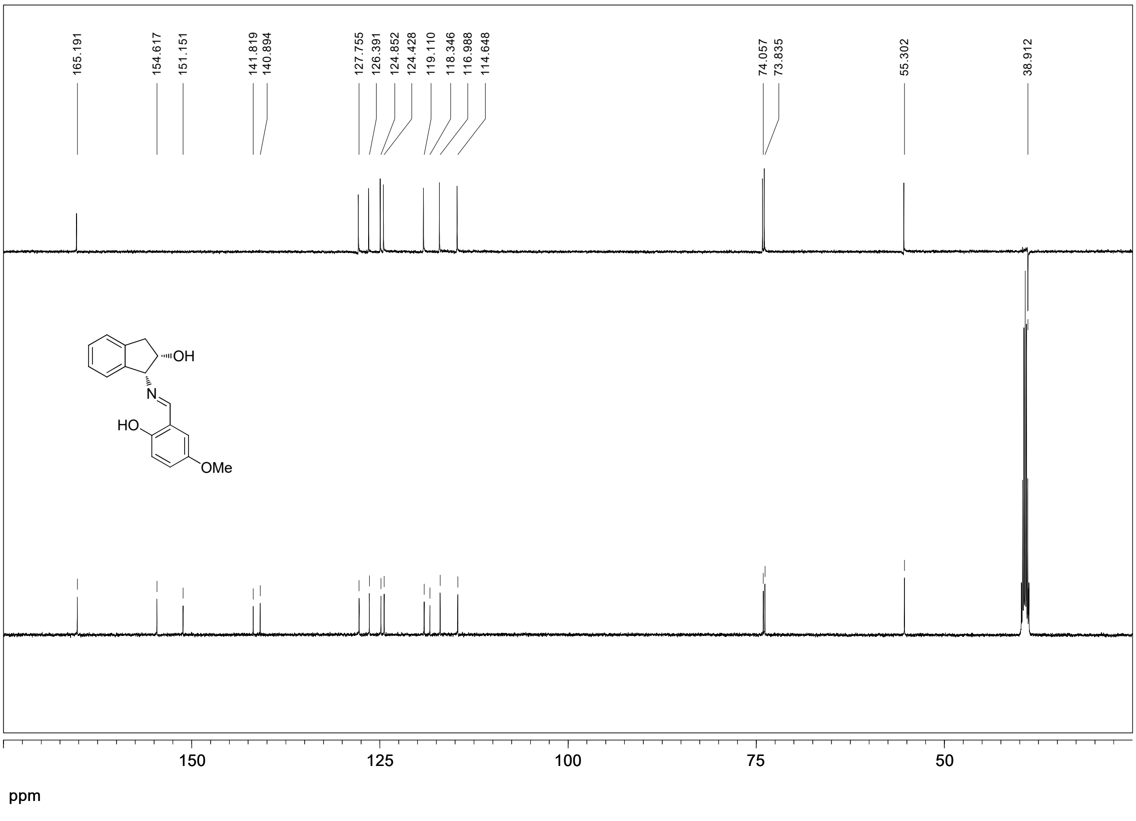

Figure S38. ^{13}C NMR and DEPT spectra of **24** in DMSO-d_6.

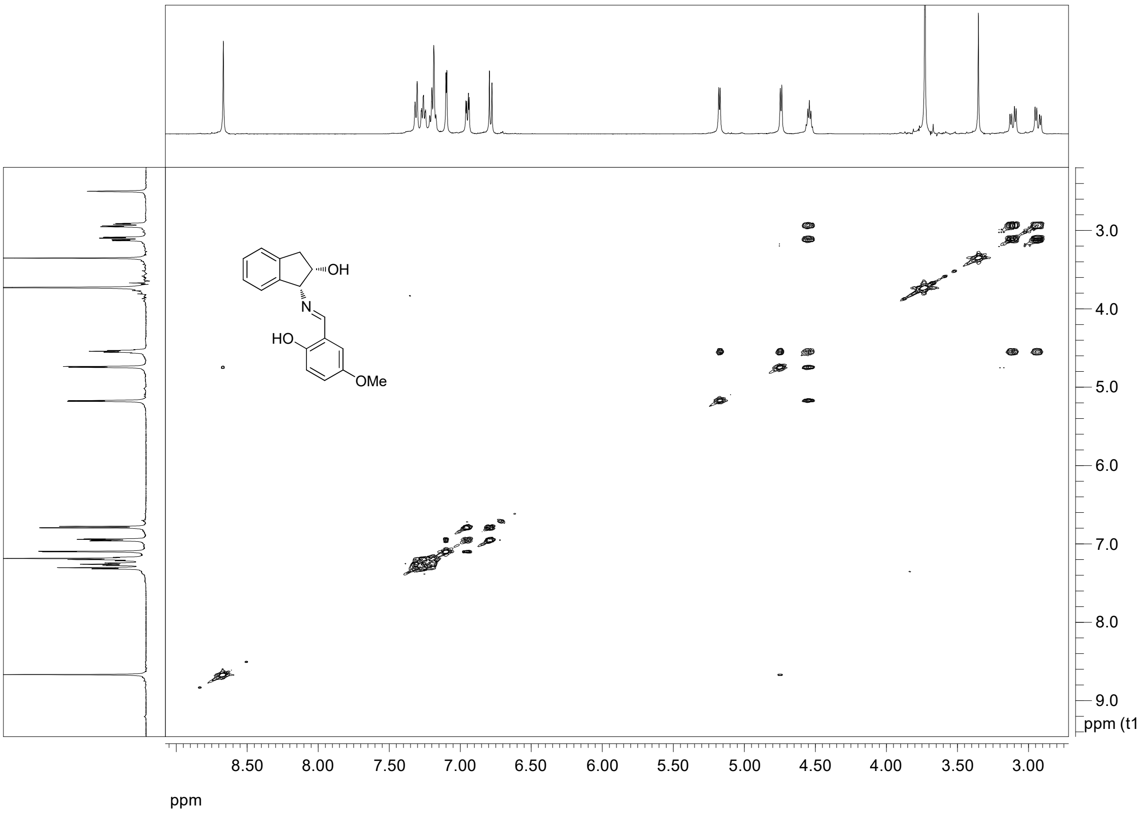

Figure S39. COSY spectrum of **24** in DMSO-d_6.

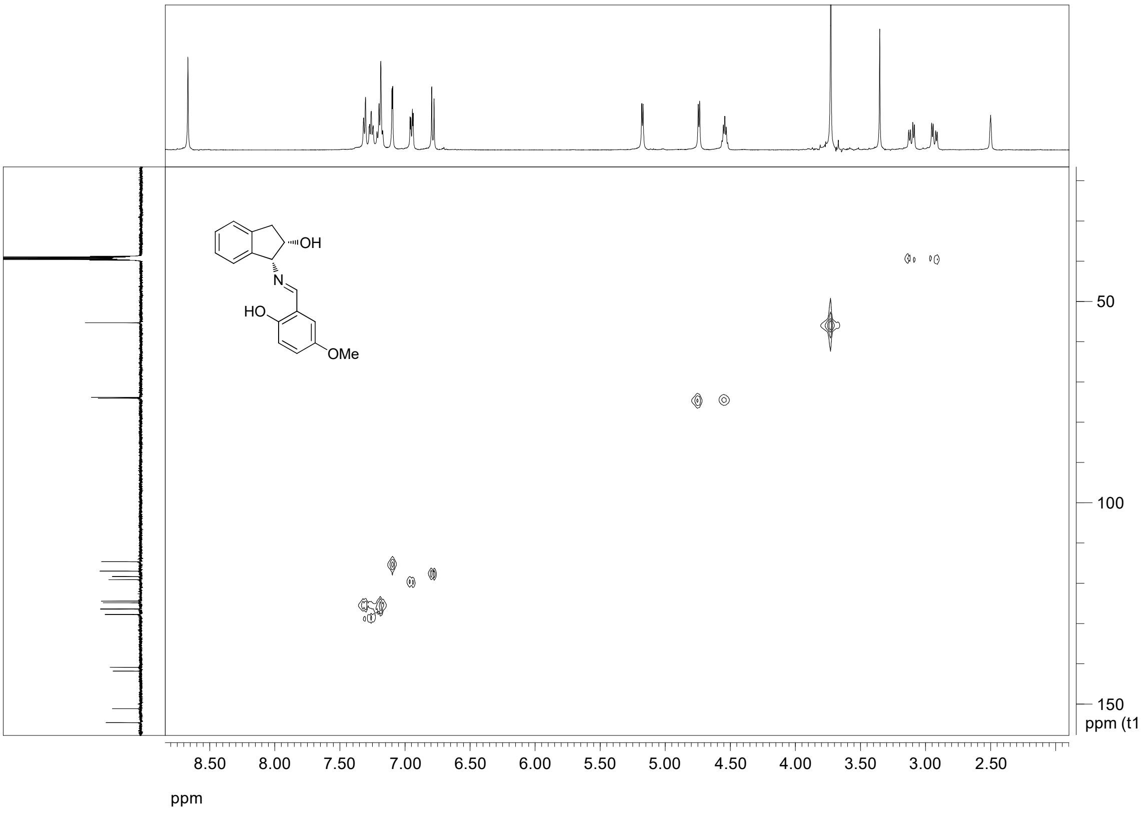

Figure S40. HMQC spectrum of **24** in DMSO-d_6.

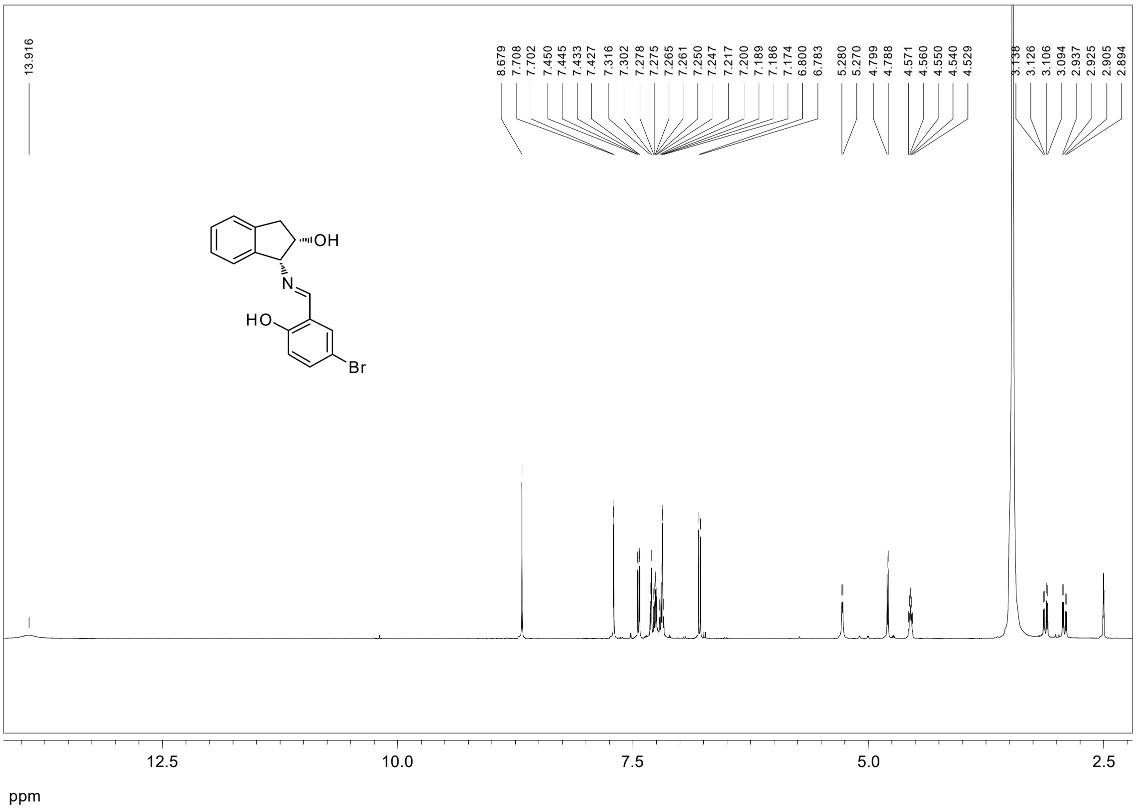

Figure S41. ^{1}H NMR spectrum of **25** in DMSO-d_6

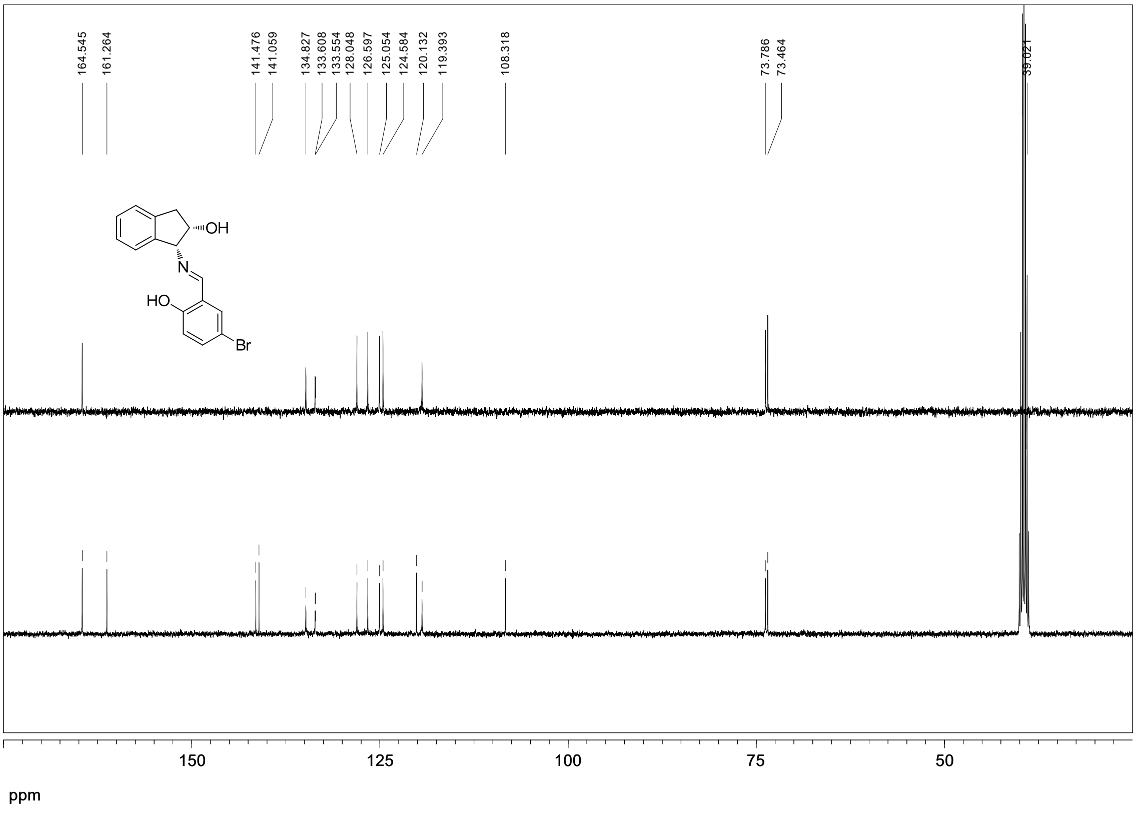

Figure S42. ^{13}C NMR and DEPT spectra of **25** in DMSO-d_6.

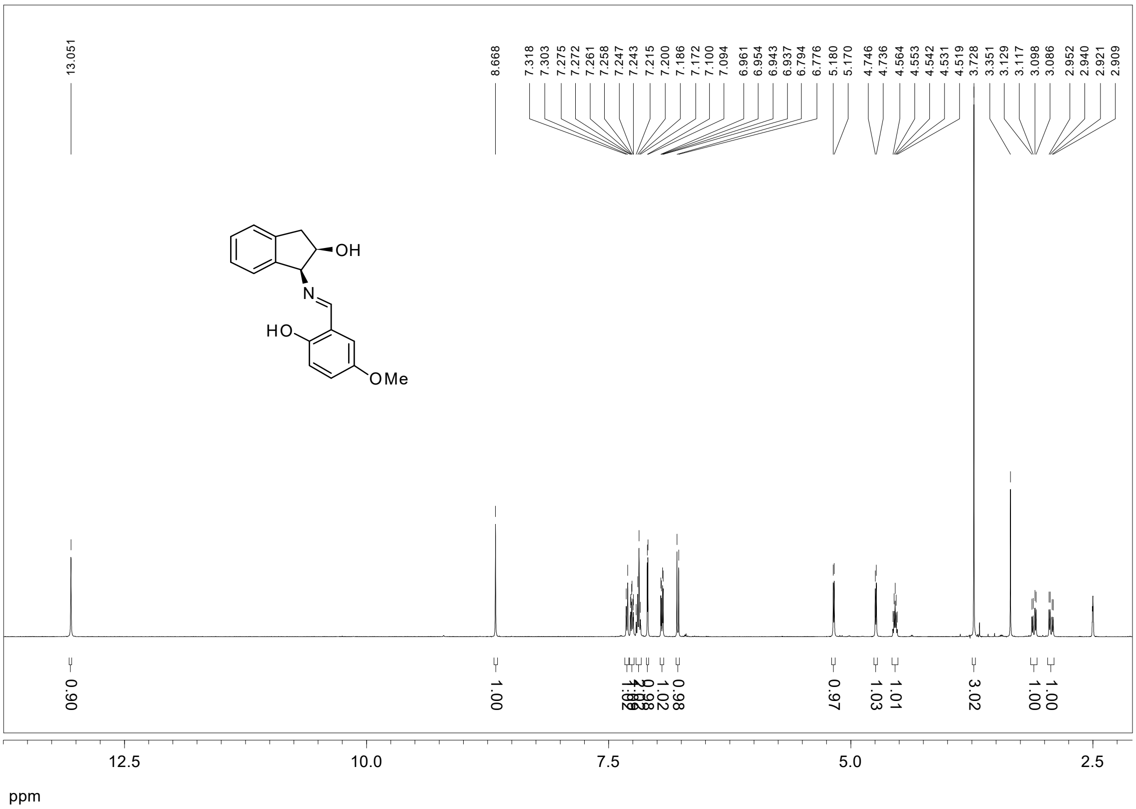

Figure S43. ^{1}H NMR spectrum of **31** in DMSO-d_6.

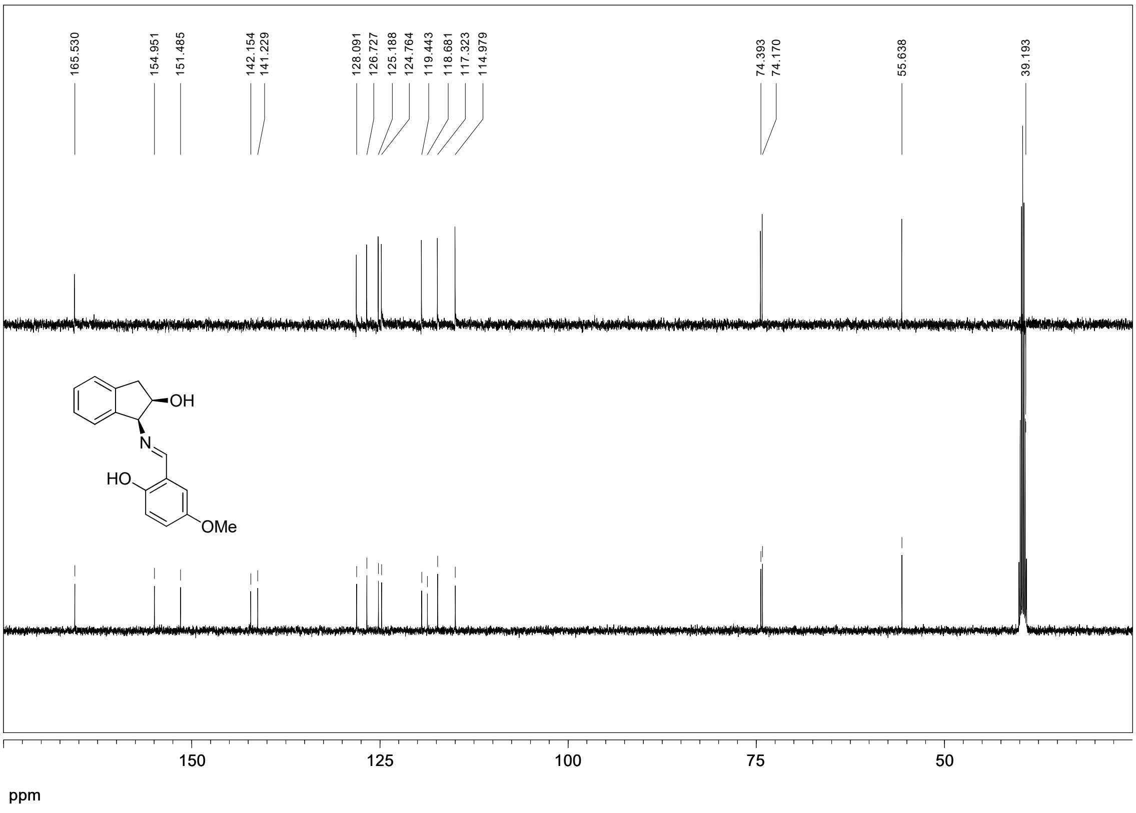

Figure S44. ^{13}C NMR and DEPT spectra of **31** in DMSO-d_6.

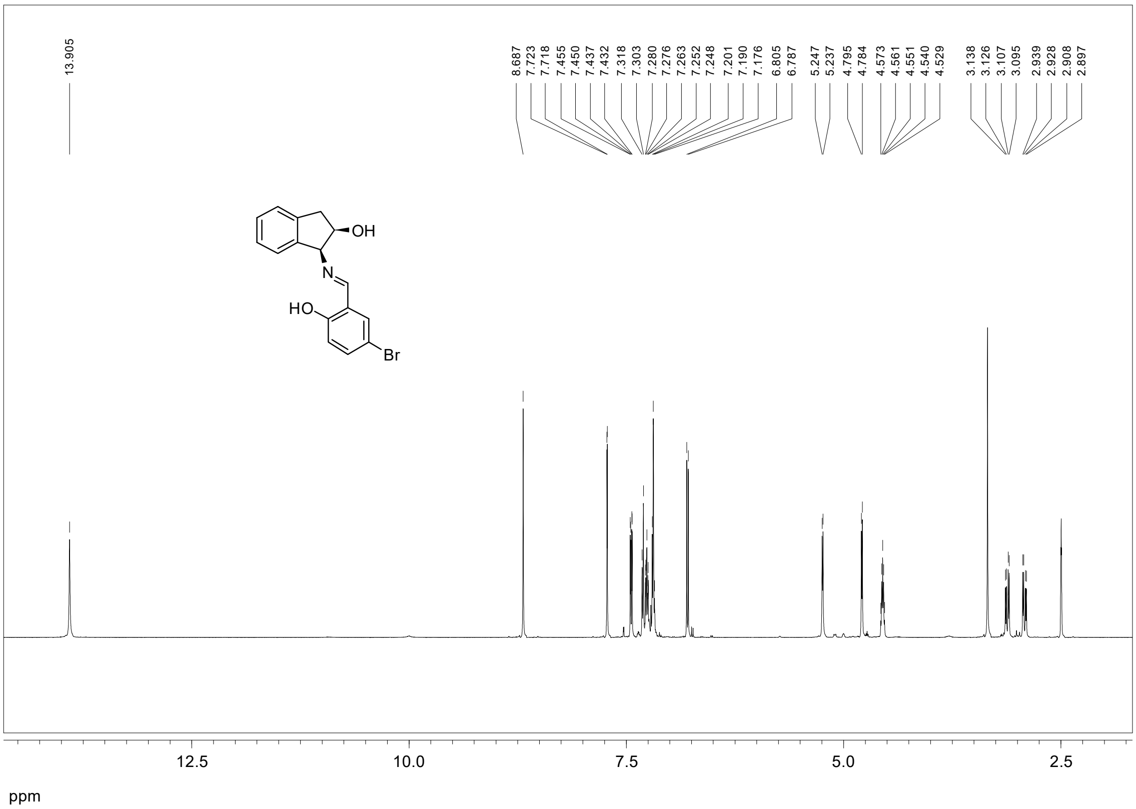

Figure S45. ^{1}H NMR spectrum of **32** in DMSO-d_6.

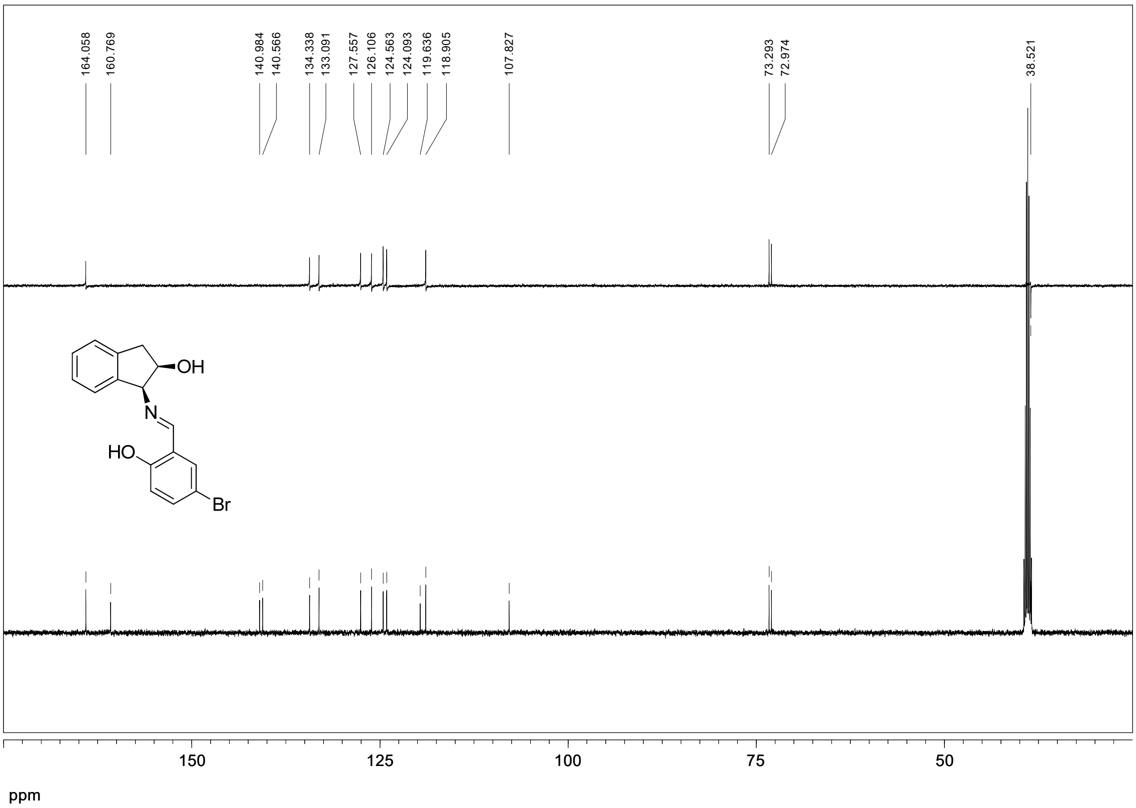

Figure S46. ^{13}C NMR and DEPT spectra of **32** in DMSO-d_6.

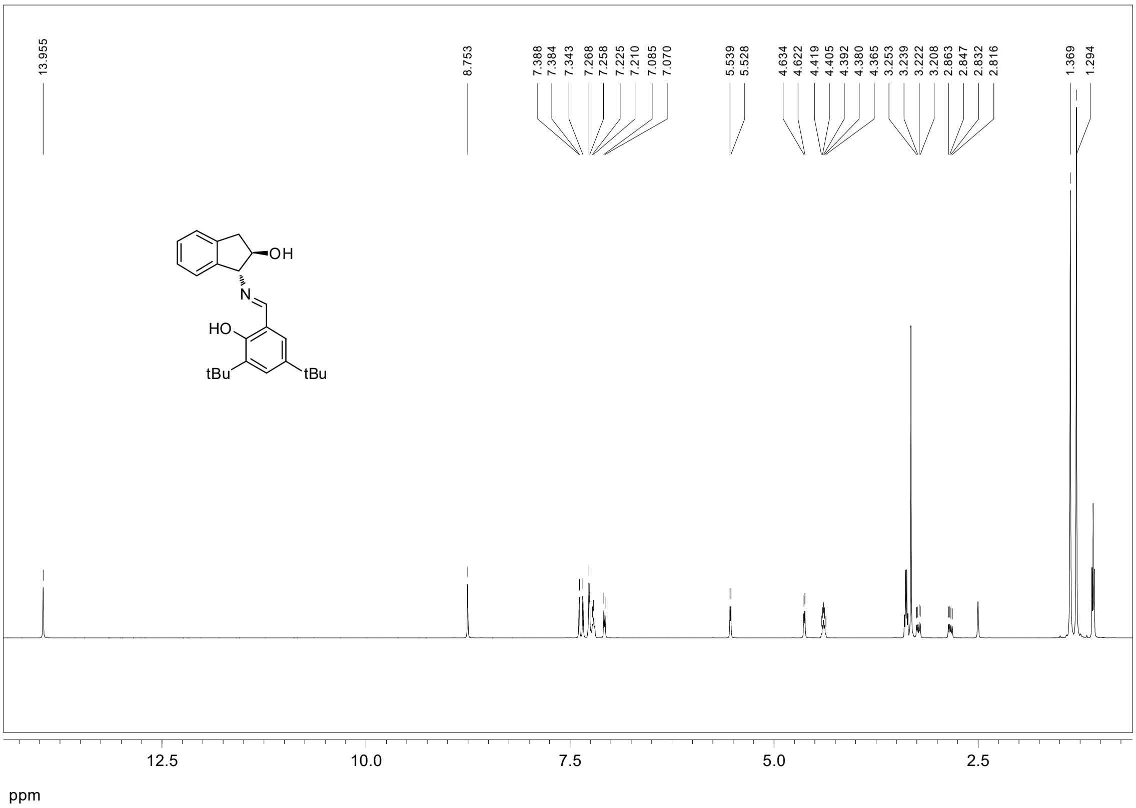

Figure S47. ^{1}H NMR spectrum of **37** in DMSO-d_6.

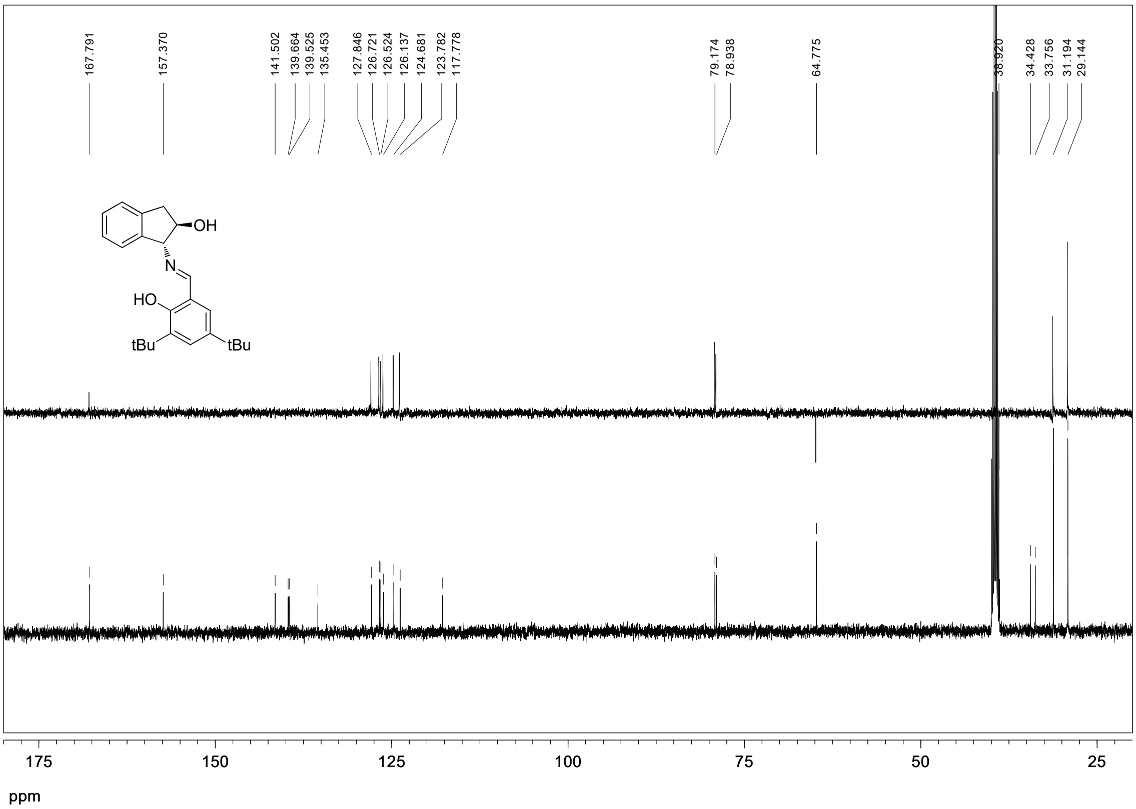

Figure S48. ^{13}C NMR and DEPT spectra of **37** in DMSO-d_6.

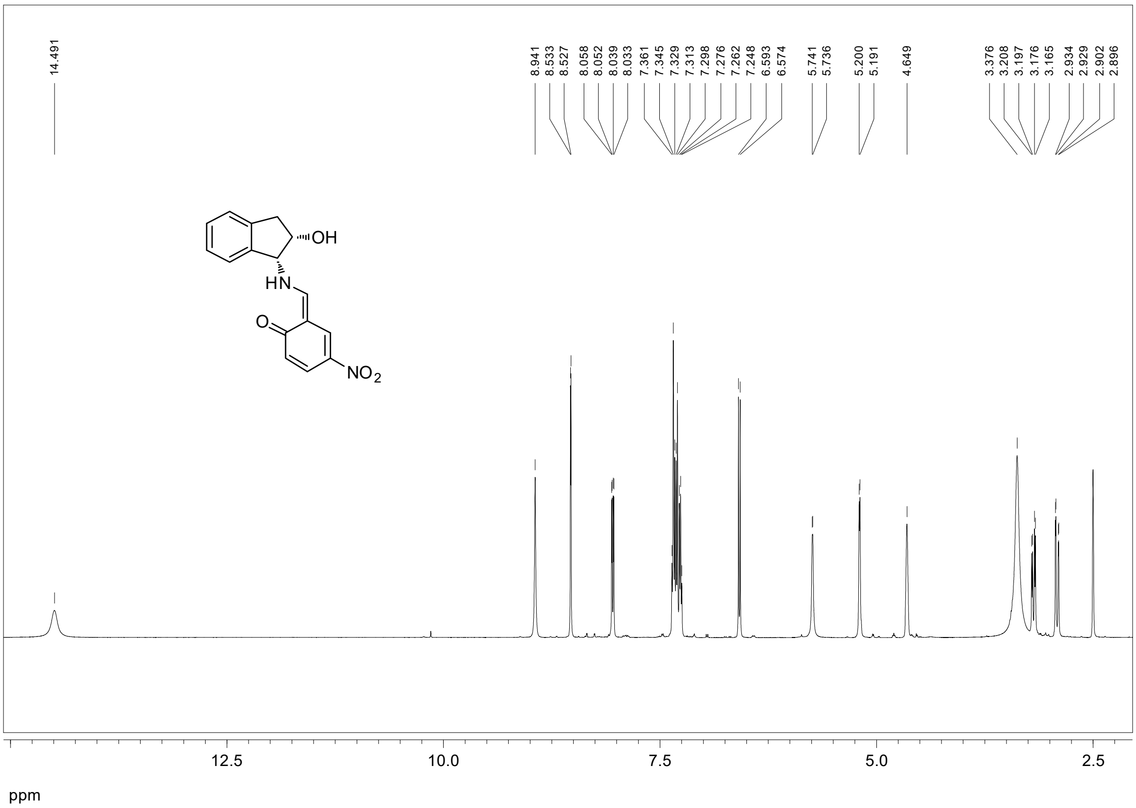

Figure S49. ^{1}H NMR spectrum of **38** in DMSO-d_6.

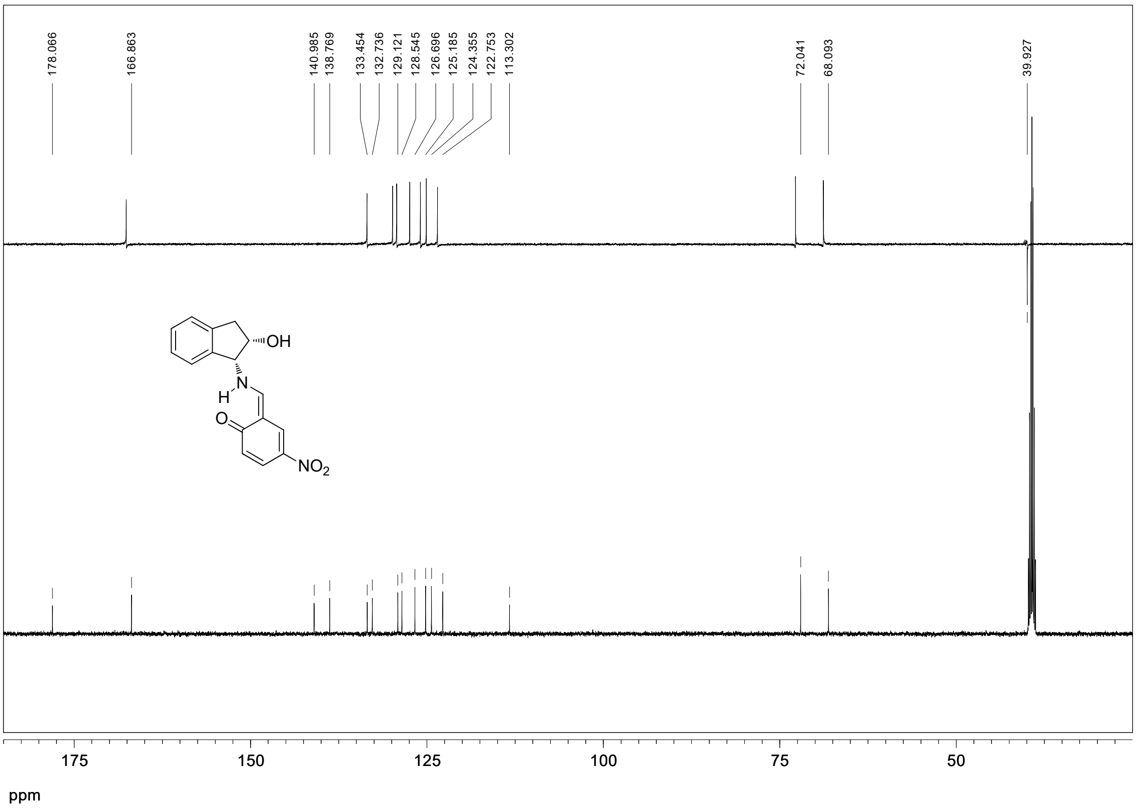

Figure S50. ^{13}C NMR and DEPT spectra of **38** in DMSO-d_6.

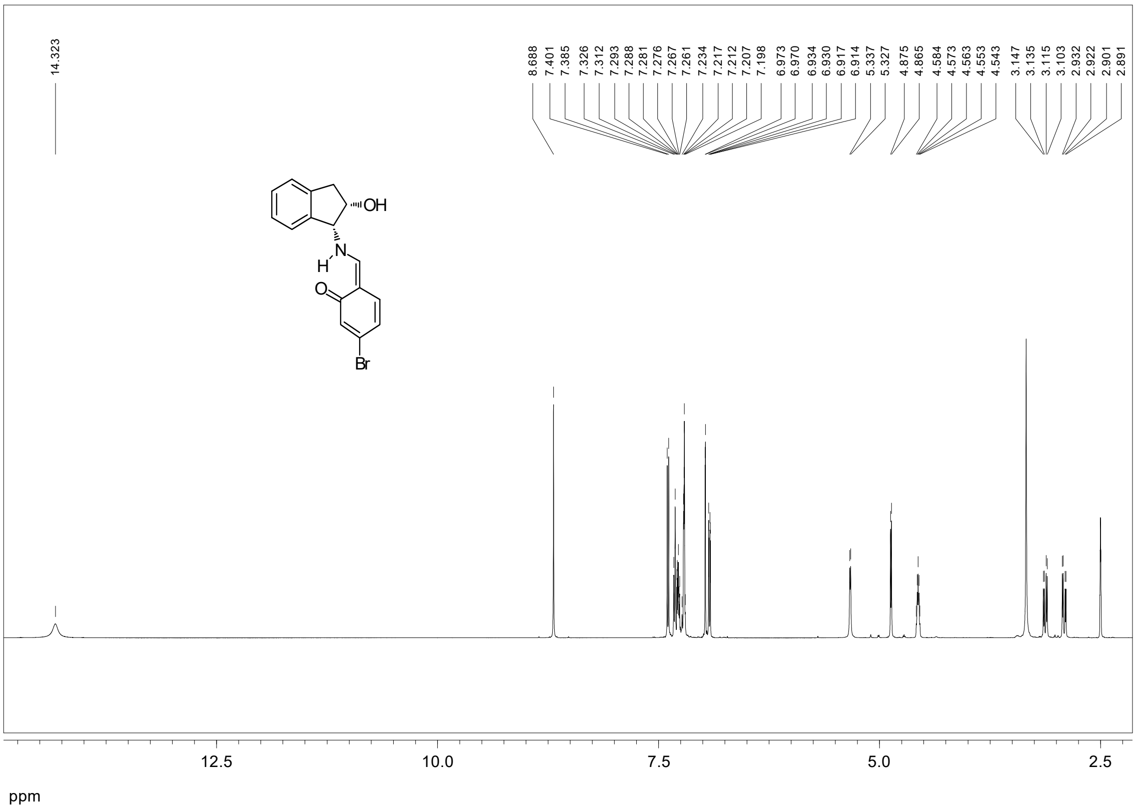

Figure S51. ^{1}H NMR spectrum of **39** (**26**) in DMSO-*d*$_6$.

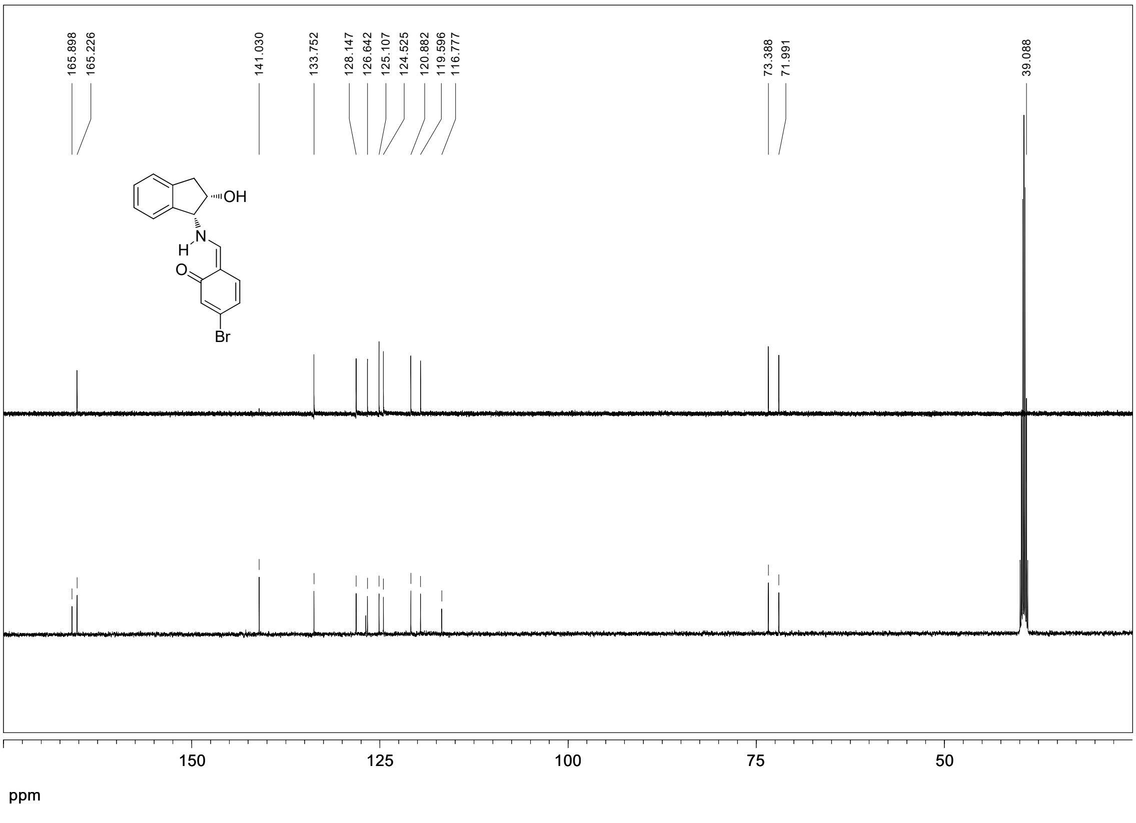

Figure S52. ^{13}C NMR and DEPT spectra of **39** (**26**) in DMSO-*d*$_6$.

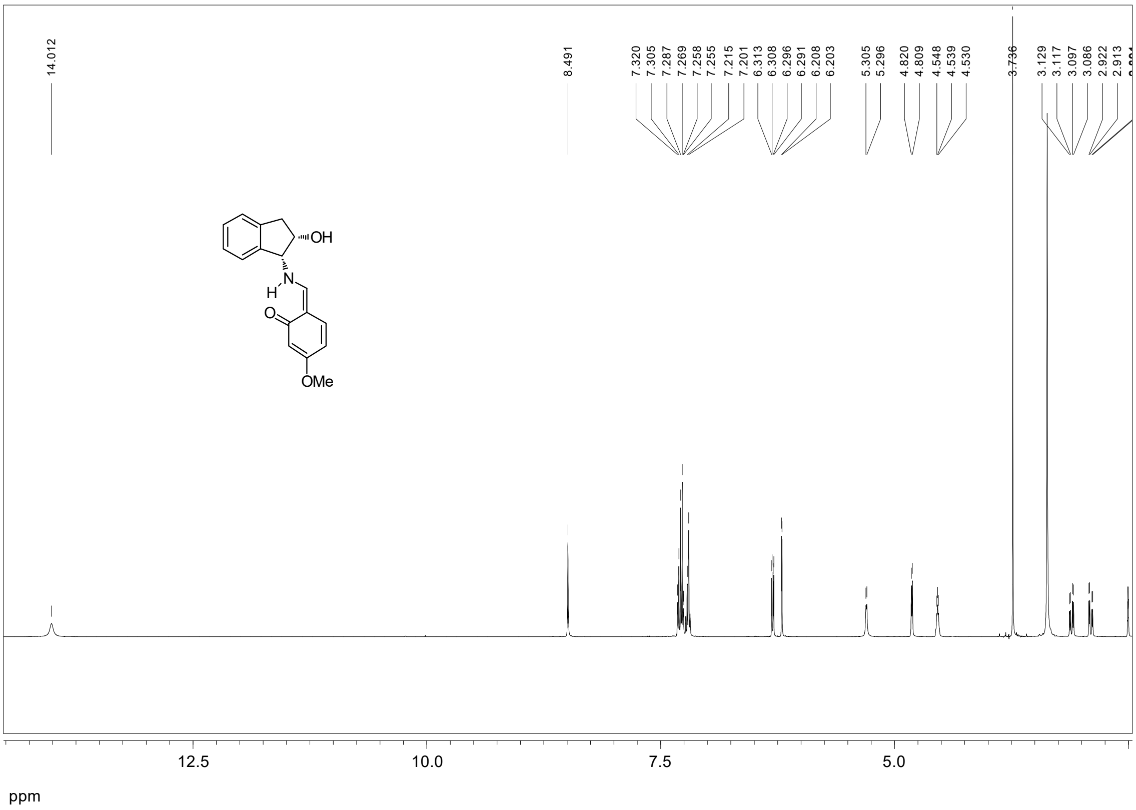

Figure S53. ^{1}H NMR spectrum of **40** (**27**) in DMSO-d_6.

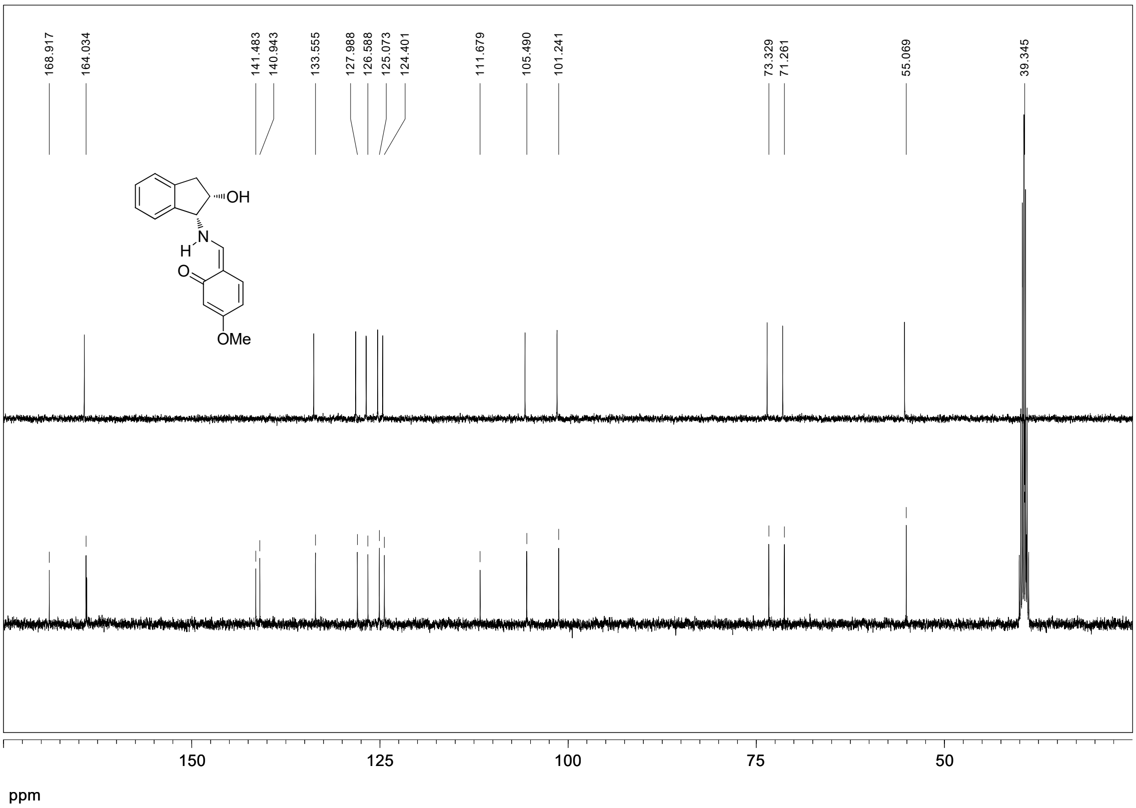

Figure S54. ^{13}C NMR and DEPT spectra of **40** (**27**) in DMSO-d_6.

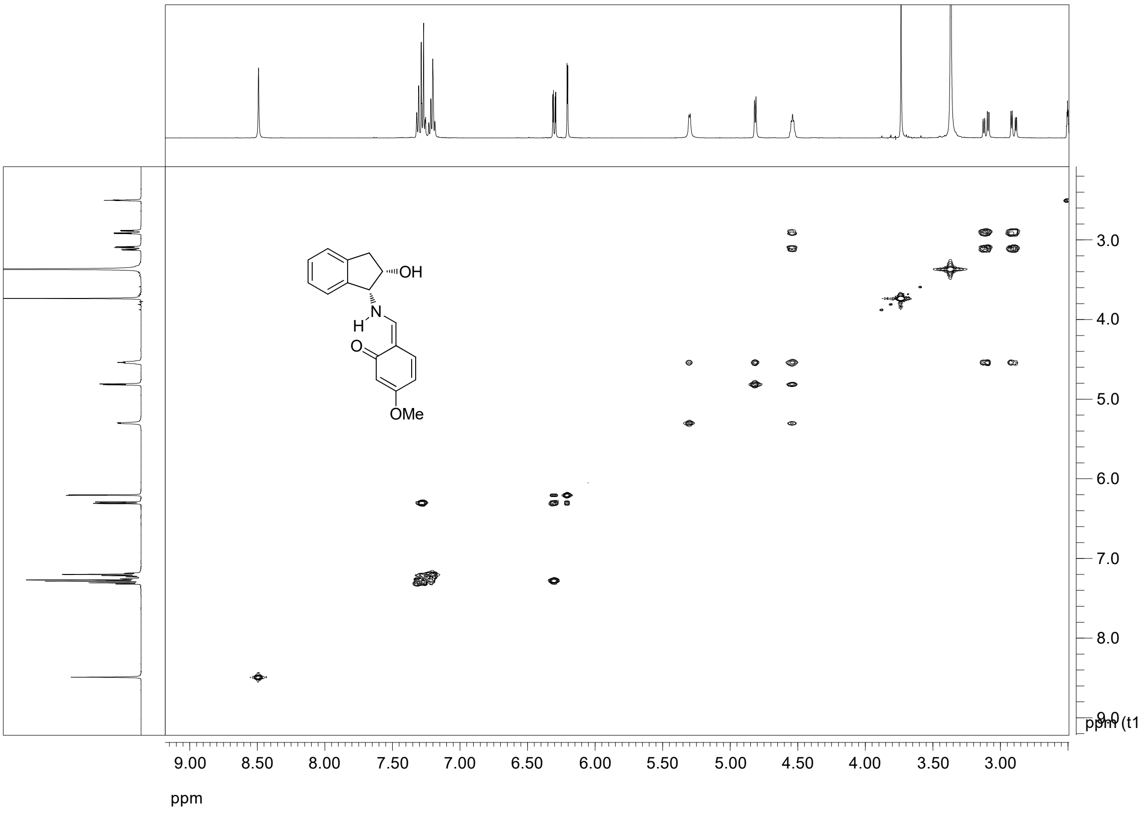

Figure S55. COSY spectrum of **40** (**27**) in DMSO-*d*6.

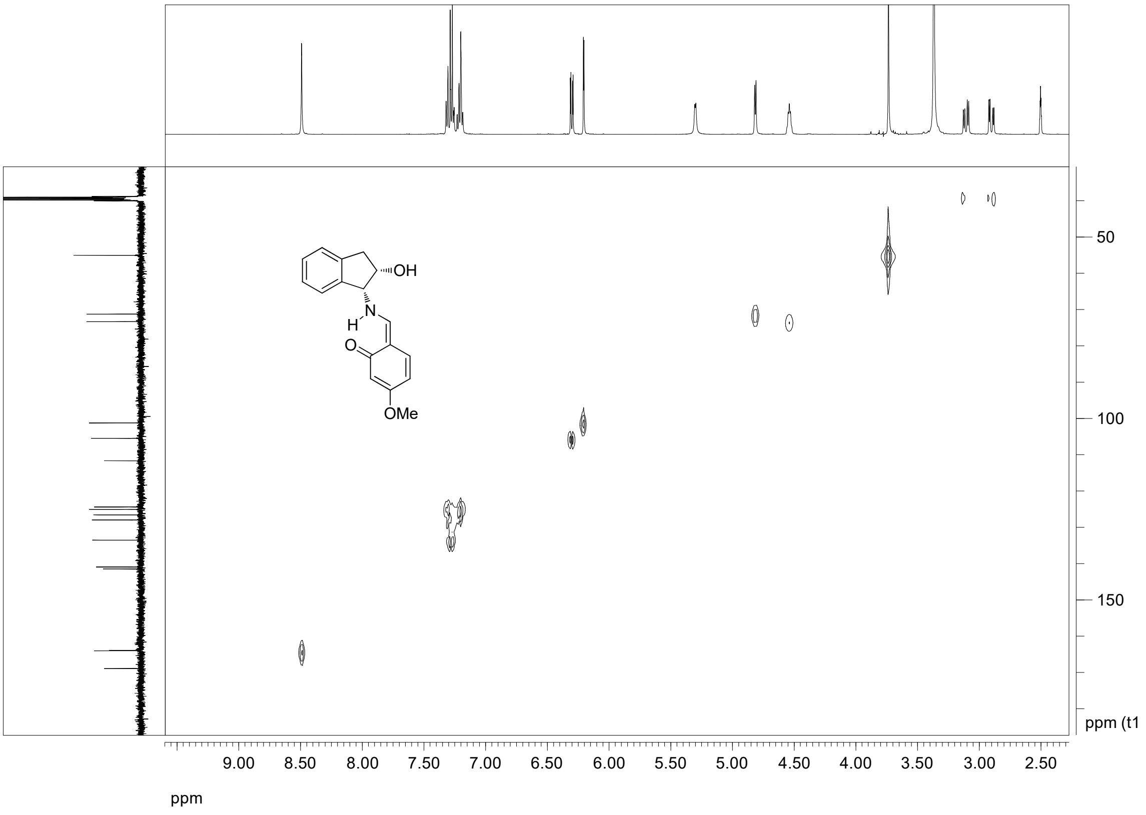

Figure S56. HMQC spectrum of **40** (**27**) in DMSO-*d*6.

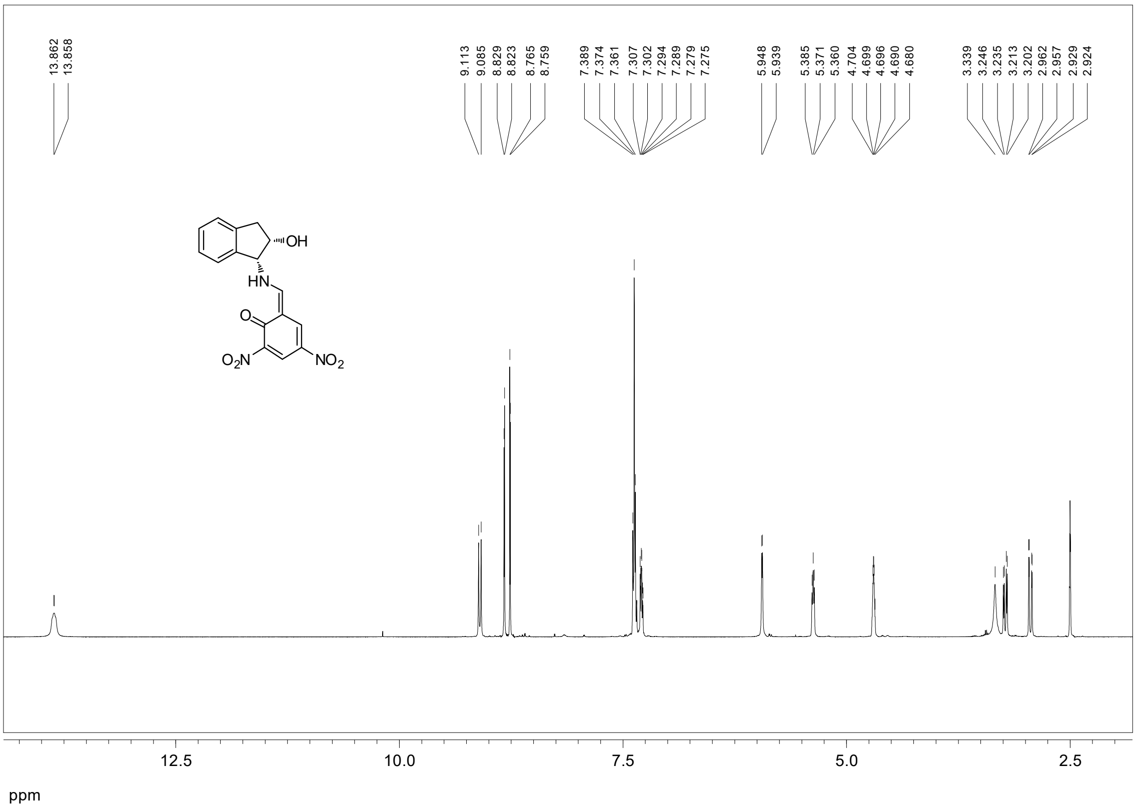

Figure S57. ^{1}H NMR spectrum of **41** in DMSO-d_6.

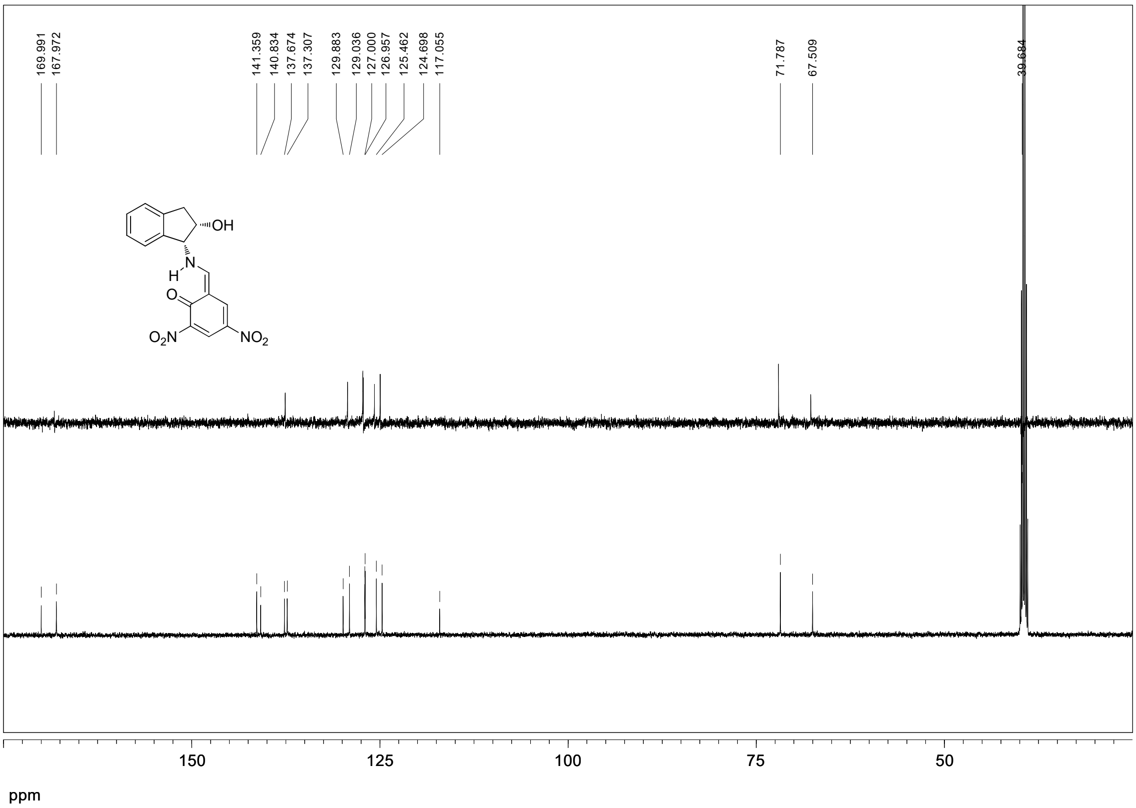

Figure S58. ^{13}C NMR and DEPT spectra of **41** in DMSO-d_6.

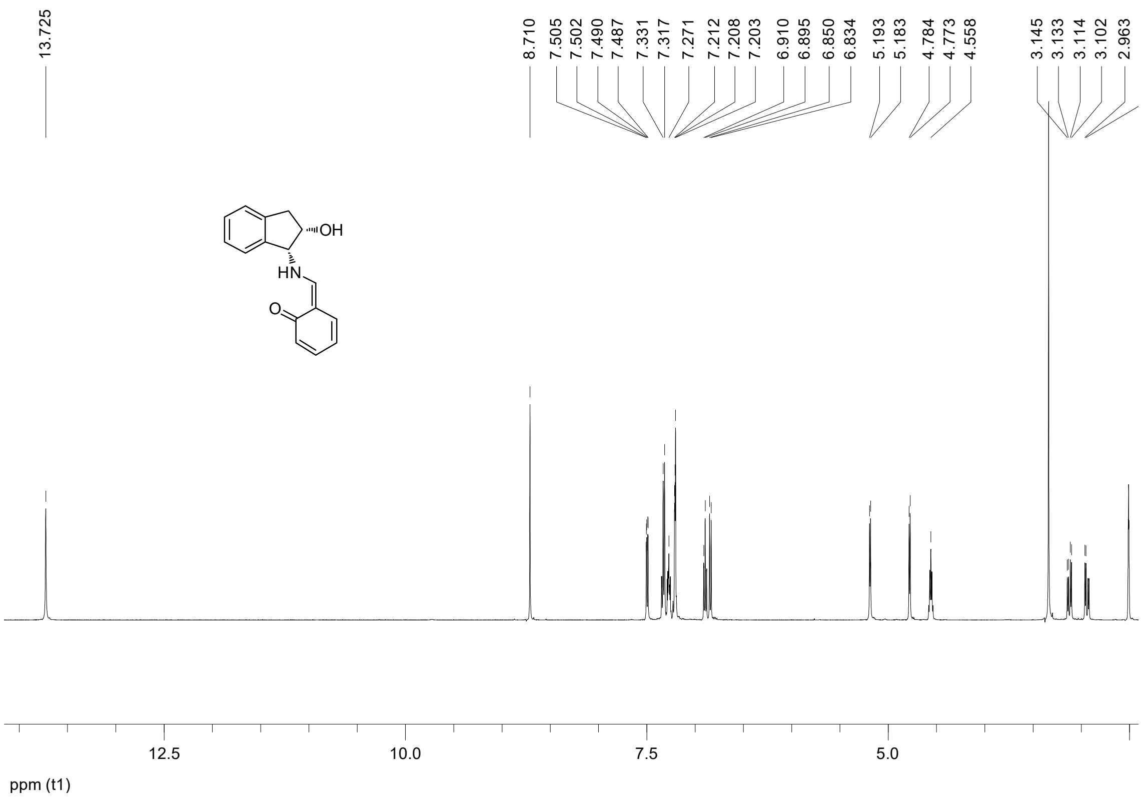

Figure S59. ^{1}H NMR spectrum of **42** (**29**) in DMSO-d_6.

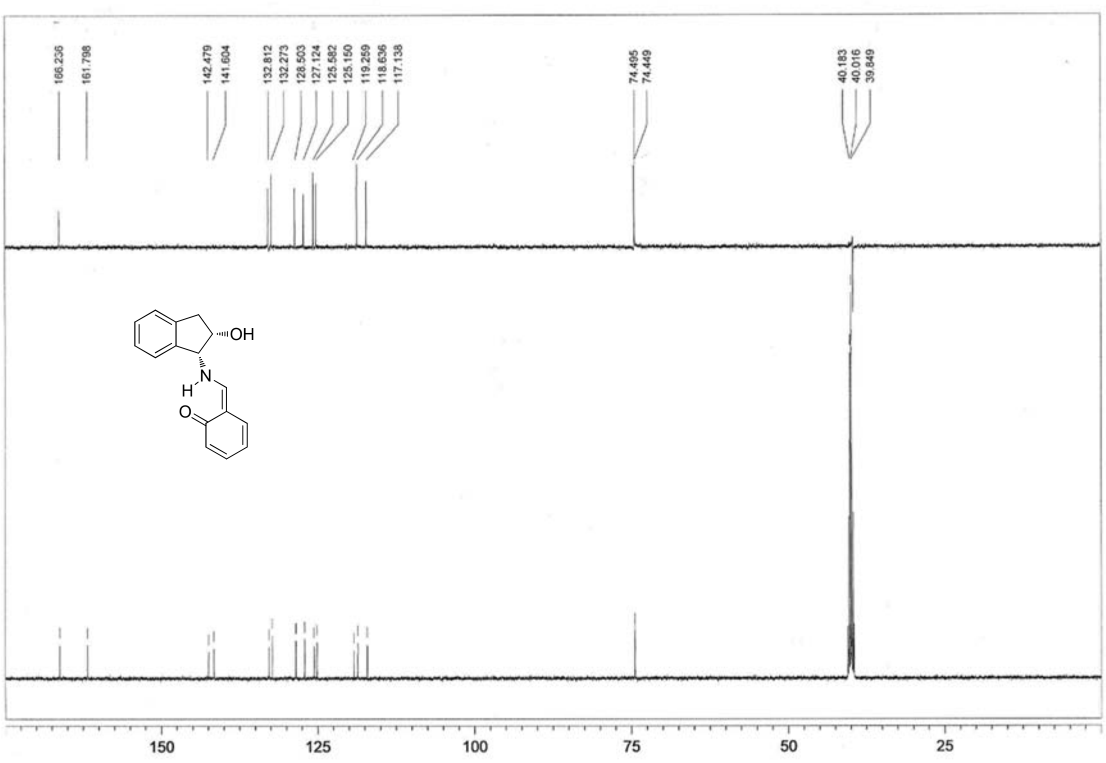

Figure S60. ^{13}C NMR and DEPT spectra of **42** (**29**) in DMSO-d_6.

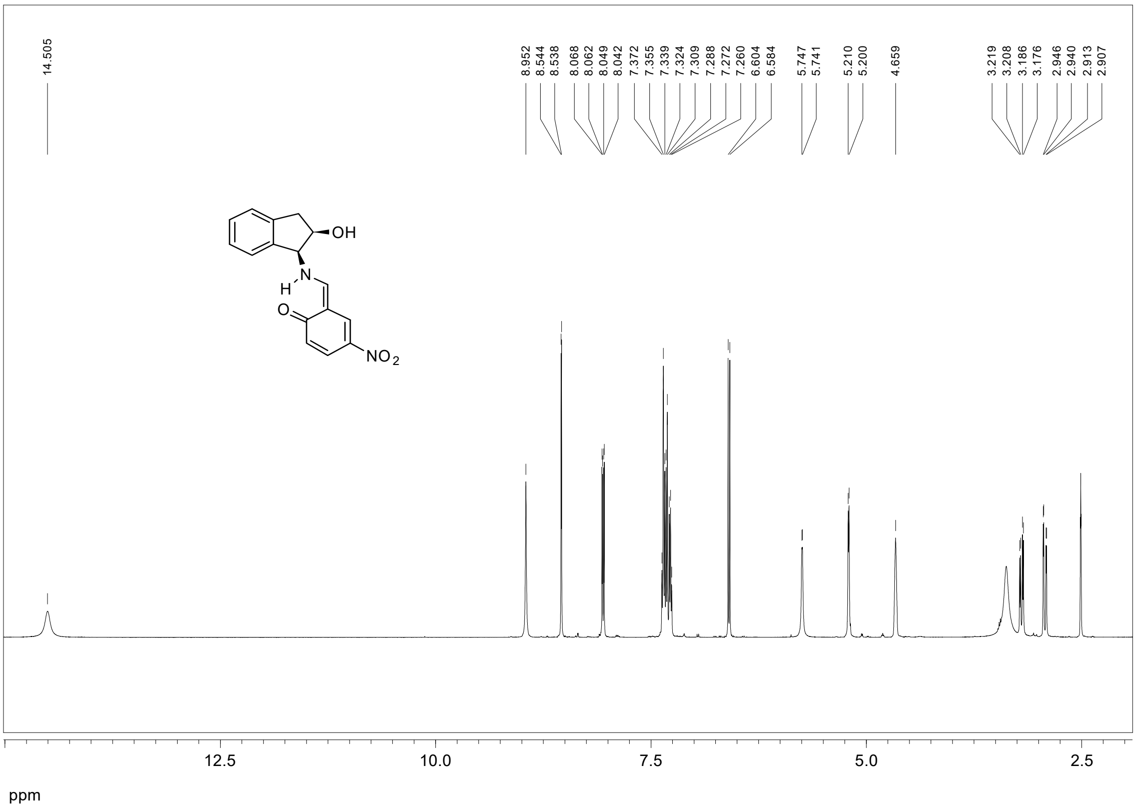

Figure S61. ^{1}H NMR spectrum of **43** in DMSO-d_6.

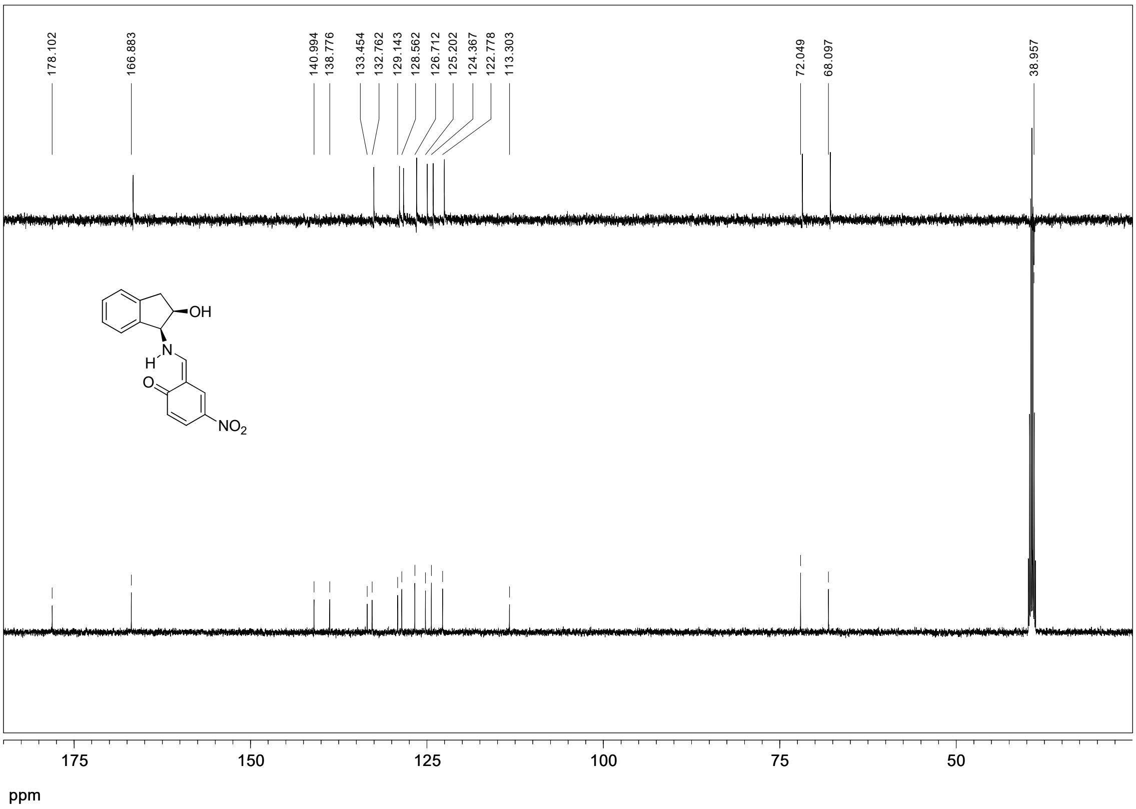

Figure S62. ^{13}C NMR and DEPT spectra of **43** in DMSO-d_6.

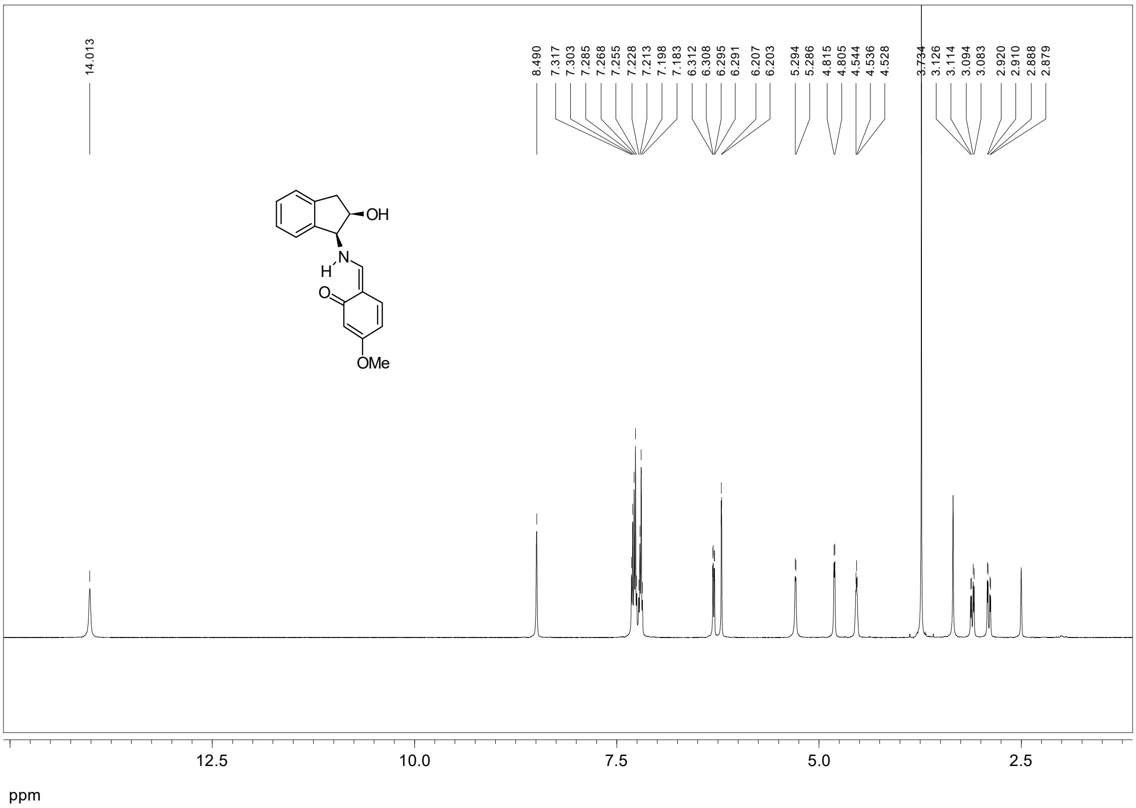

Figure S63. ^{1}H NMR spectrum of **44** (**33**) in DMSO-d_6.

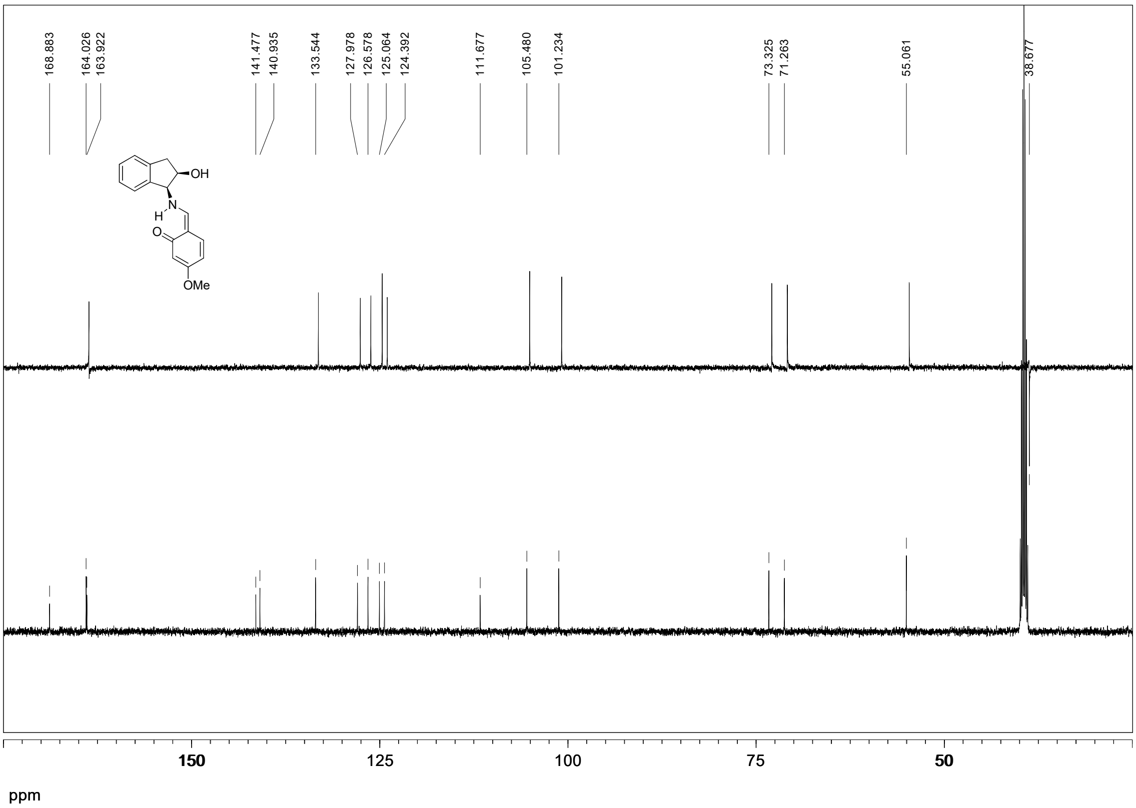

Figure S64. ^{13}C NMR and DEPT spectra of **44** (**33**) in DMSO-d_6.

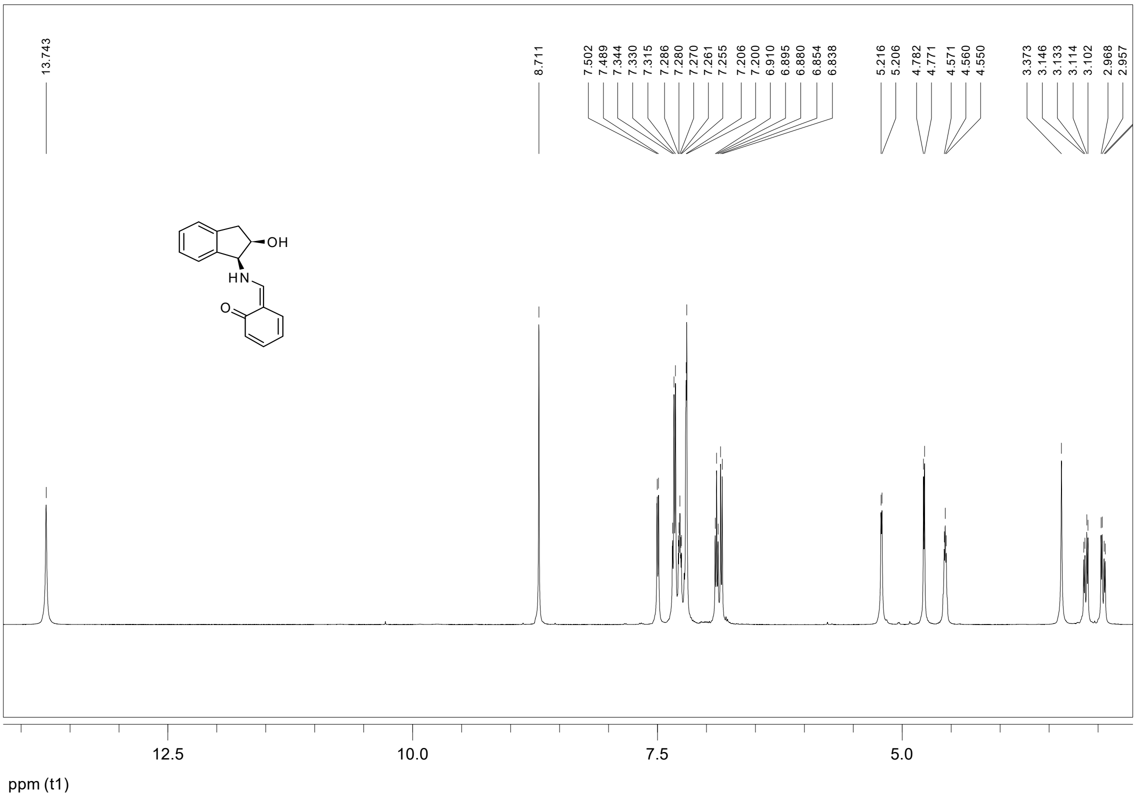

Figure S65. ^{1}H NMR spectrum of **45** (**34**) in DMSO-d_6.

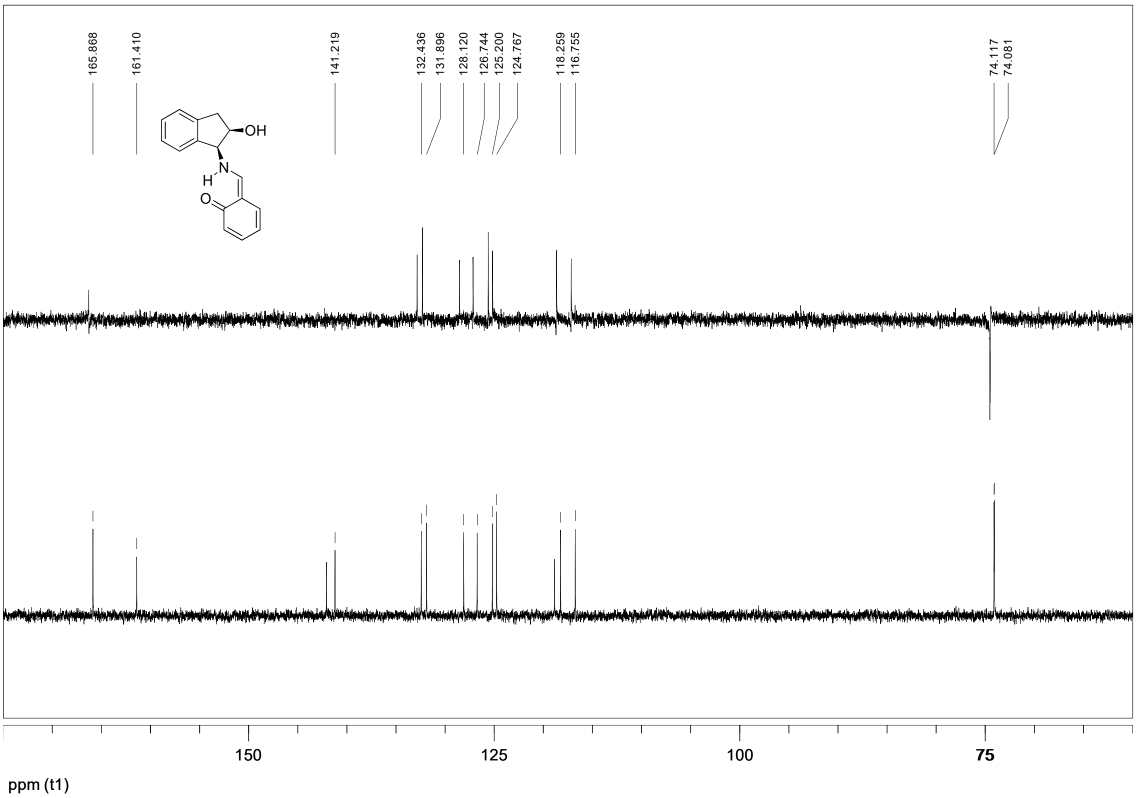

Figure S66. ^{13}C NMR and DEPT spectra of **45** (**34**) in DMSO-d_6.

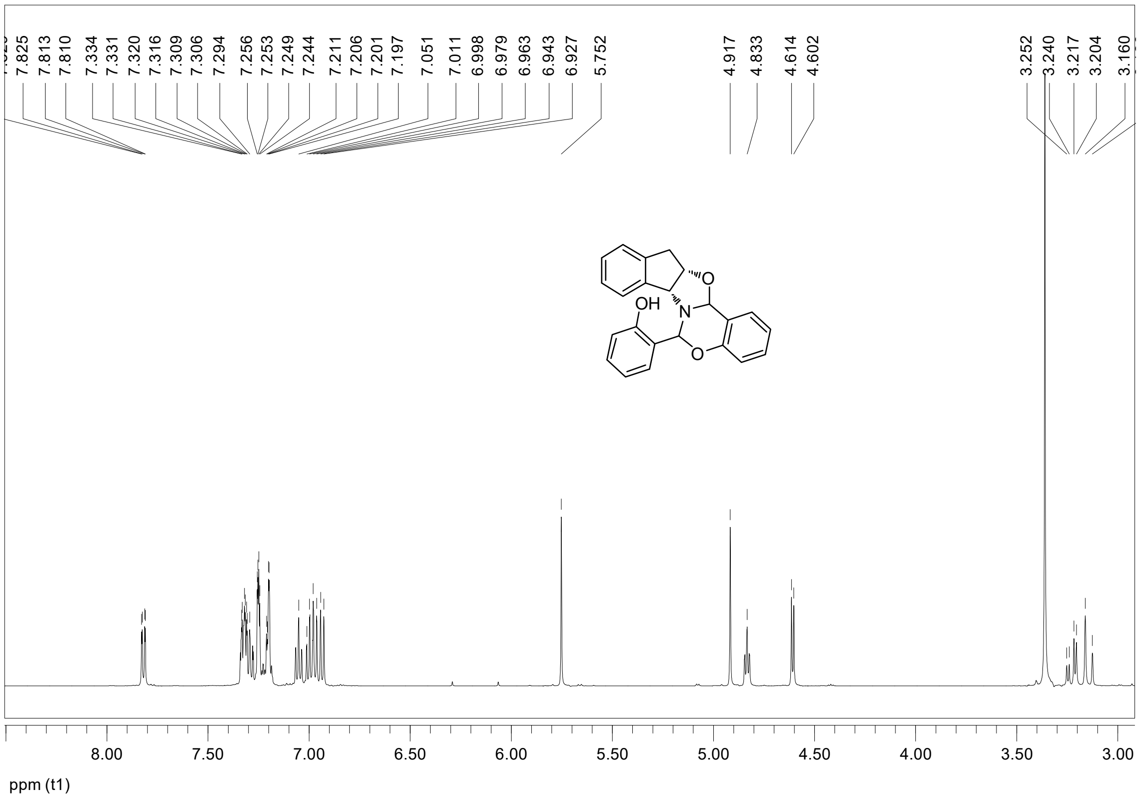

Figure S67. ^{1}H NMR spectrum of **47** in DMSO-*d*$_{6.}$

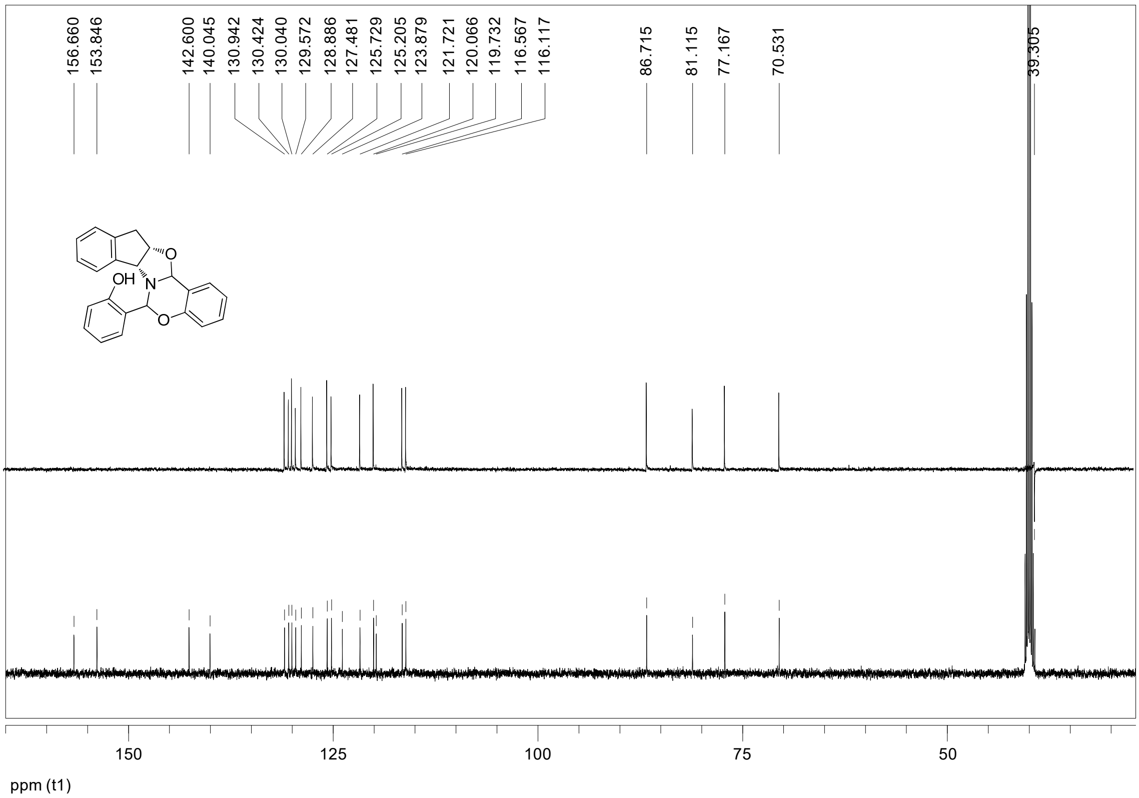

Figure S68. ^{13}C NMR and DEPT spectra of **47** in DMSO-*d*$_{6.}$

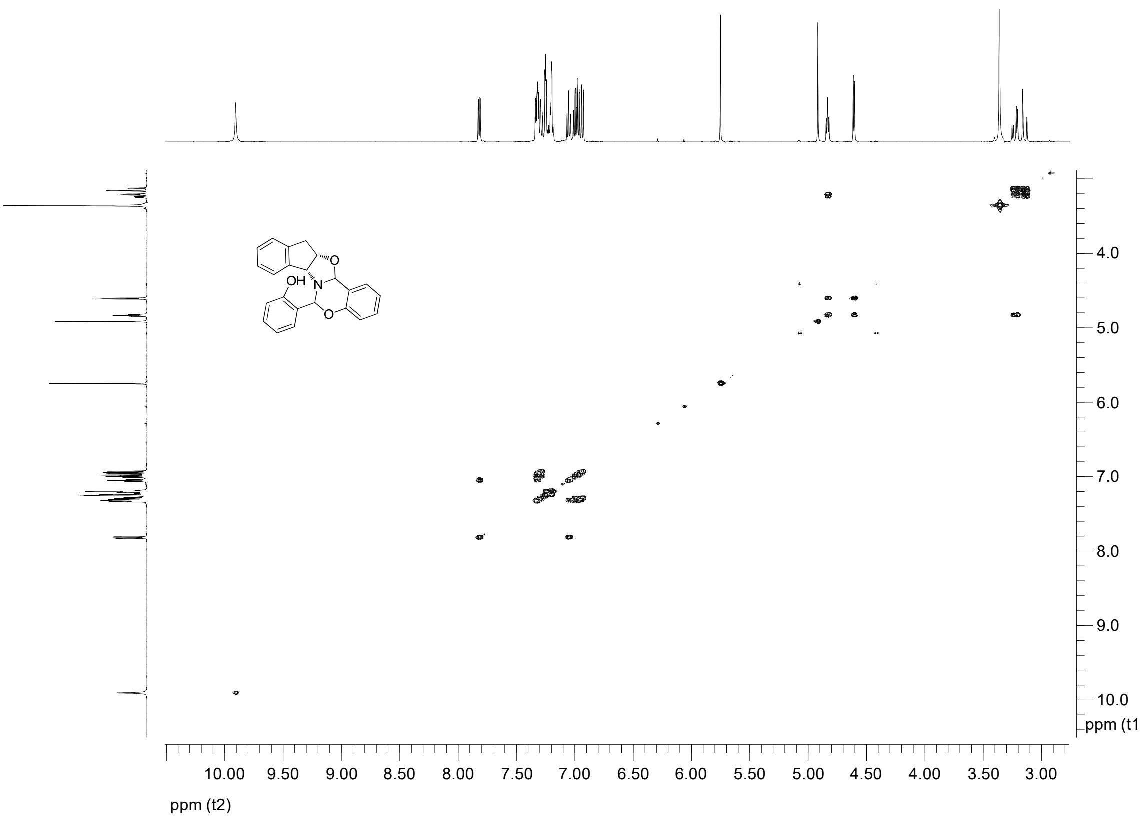

Figure S69. COSY spectrum of **47** in DMSO-d_6.

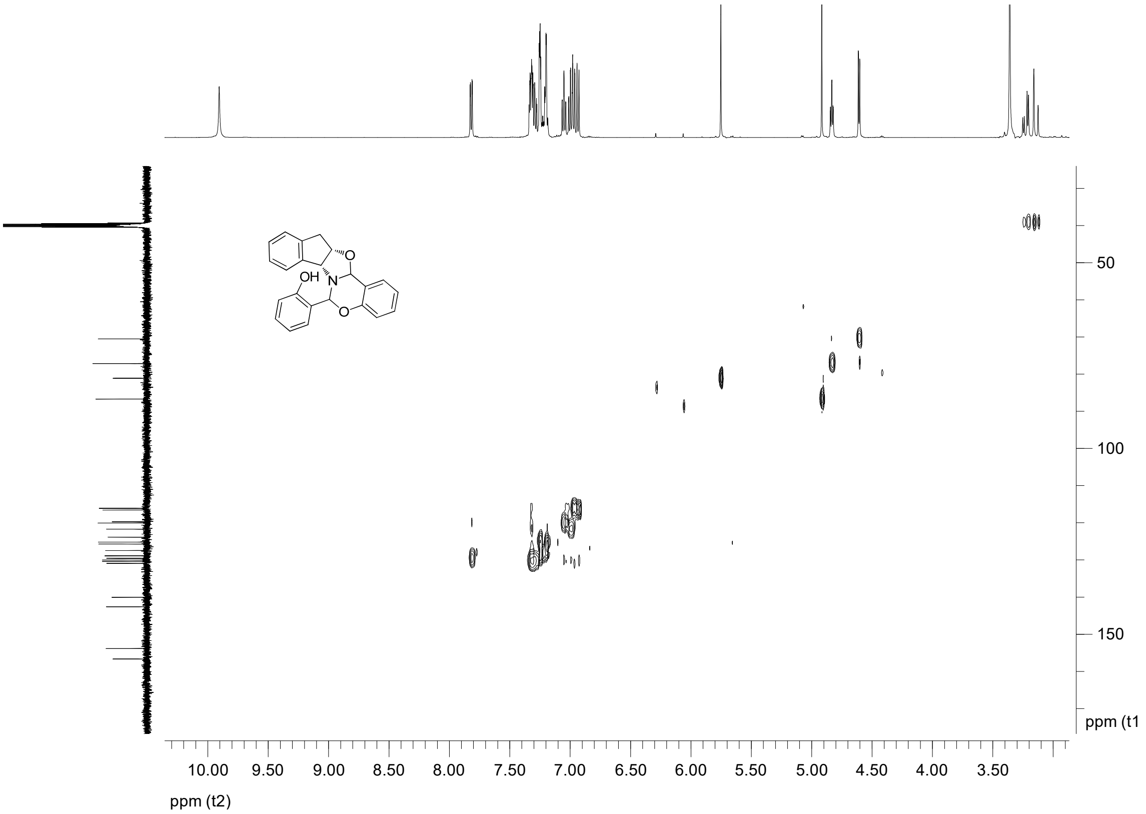

Figure S70. HMQC spectrum of **47** in DMSO-d_6.

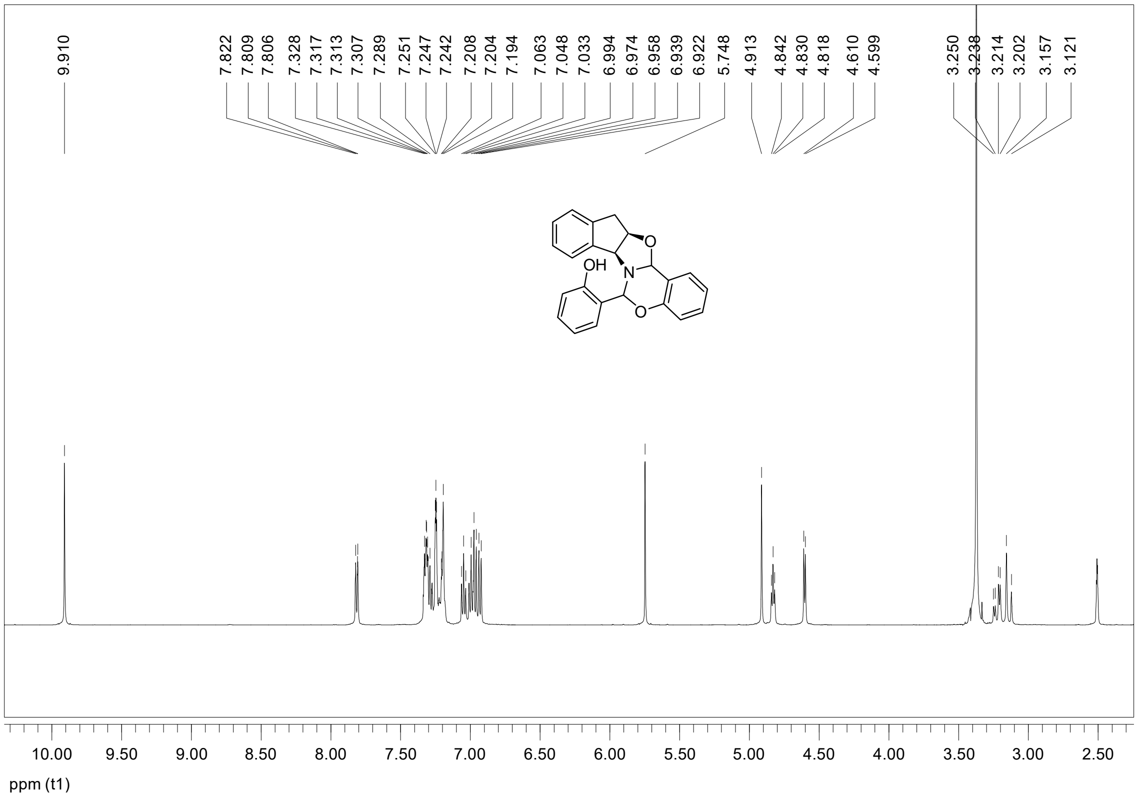

Figure S71. ^{1}H NMR spectrum of **48** in DMSO-d_6.

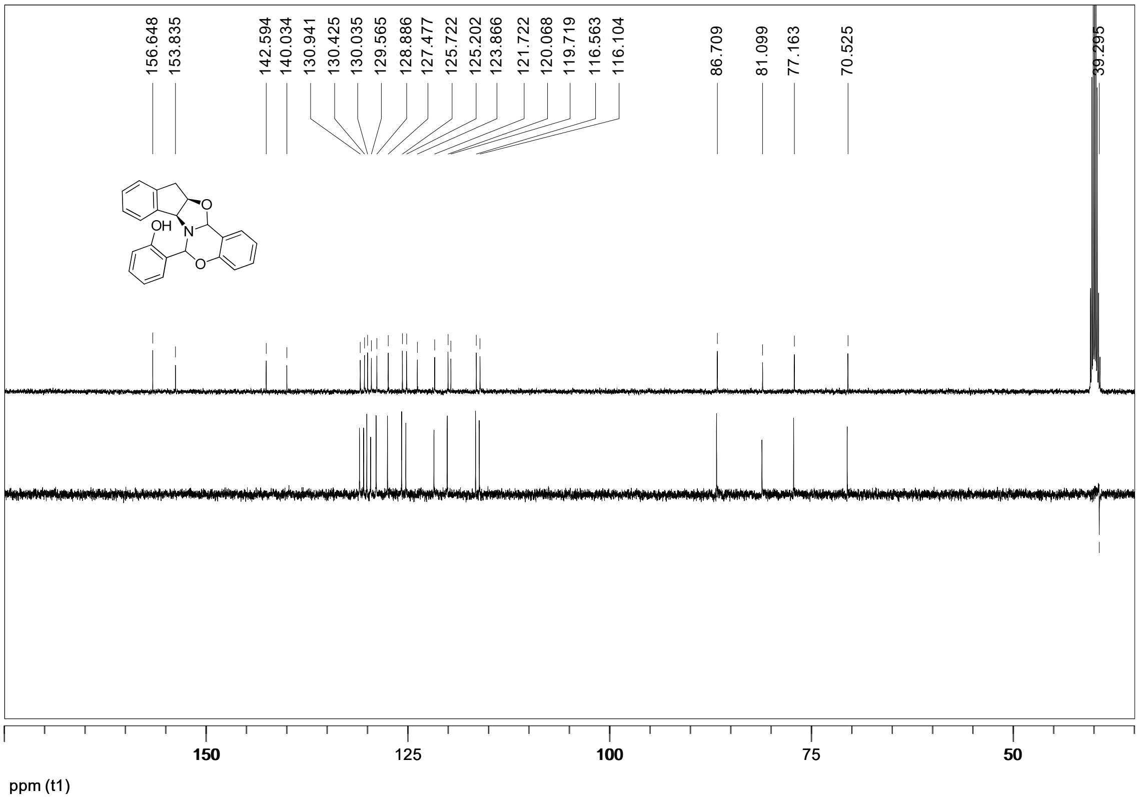

Figure S72. ^{13}C NMR and DEPT spectra of **48** in DMSO-d_6.

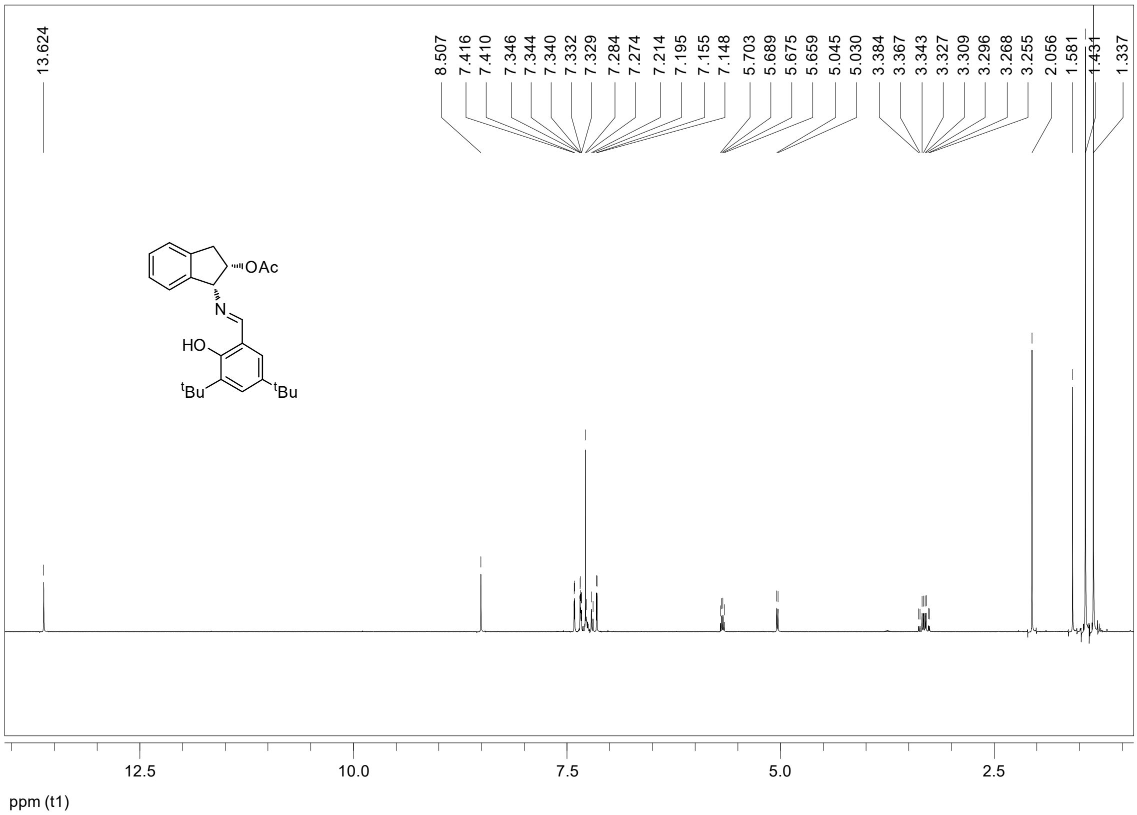

Figure S73. ^{1}H NMR spectrum of **52** in $CDCl_3$.

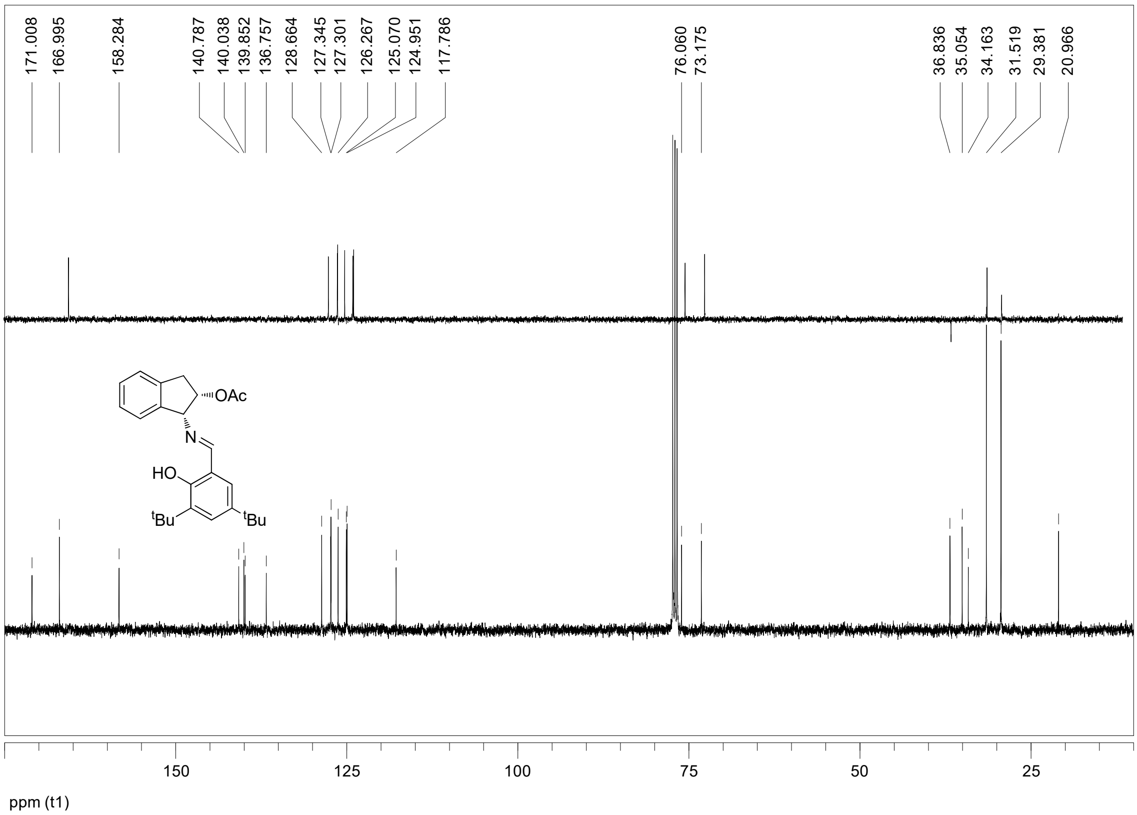

Figure S74. ^{13}C NMR and DEPT spectra of **52** in $CDCl_3$.

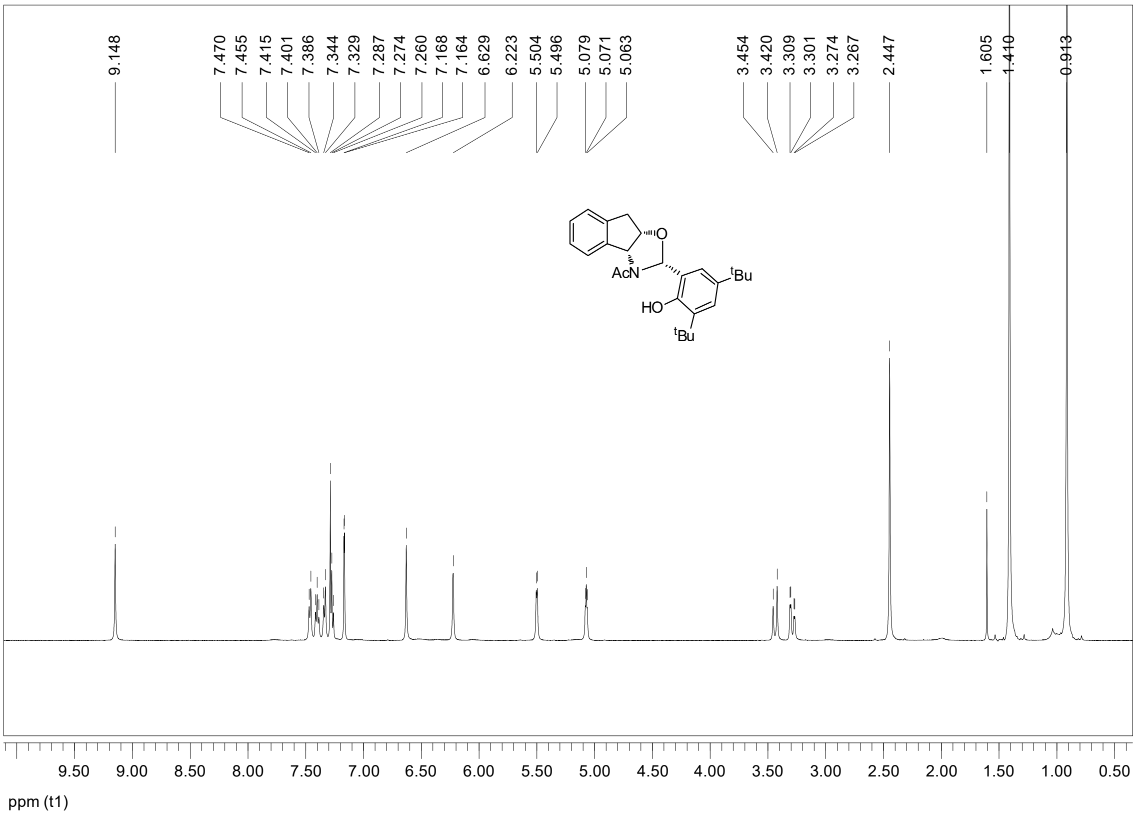

Figure S75. ^{1}H NMR spectrum of **53** in $CDCl_3$.

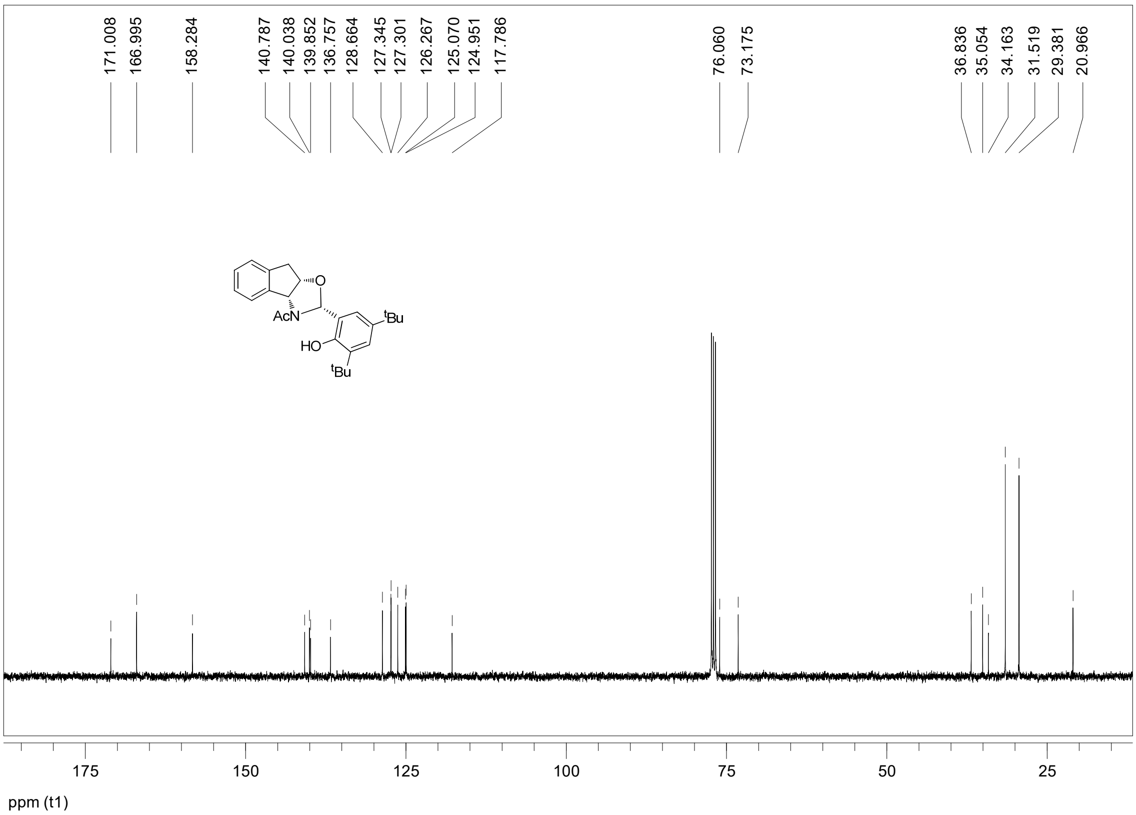

Figure S76. ^{13}C NMR spectrum of **53** in $CDCl_3$.

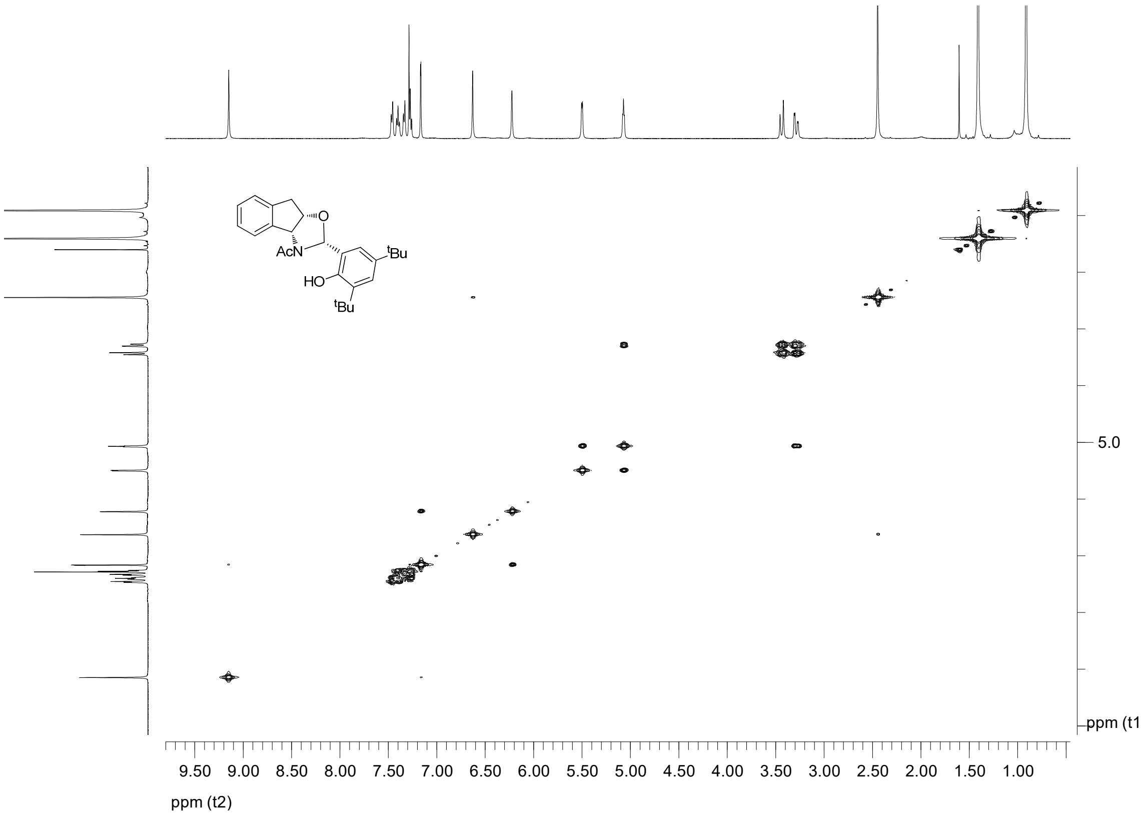

Figure S77. COSY spectrum of **53** in $CDCl_3$.

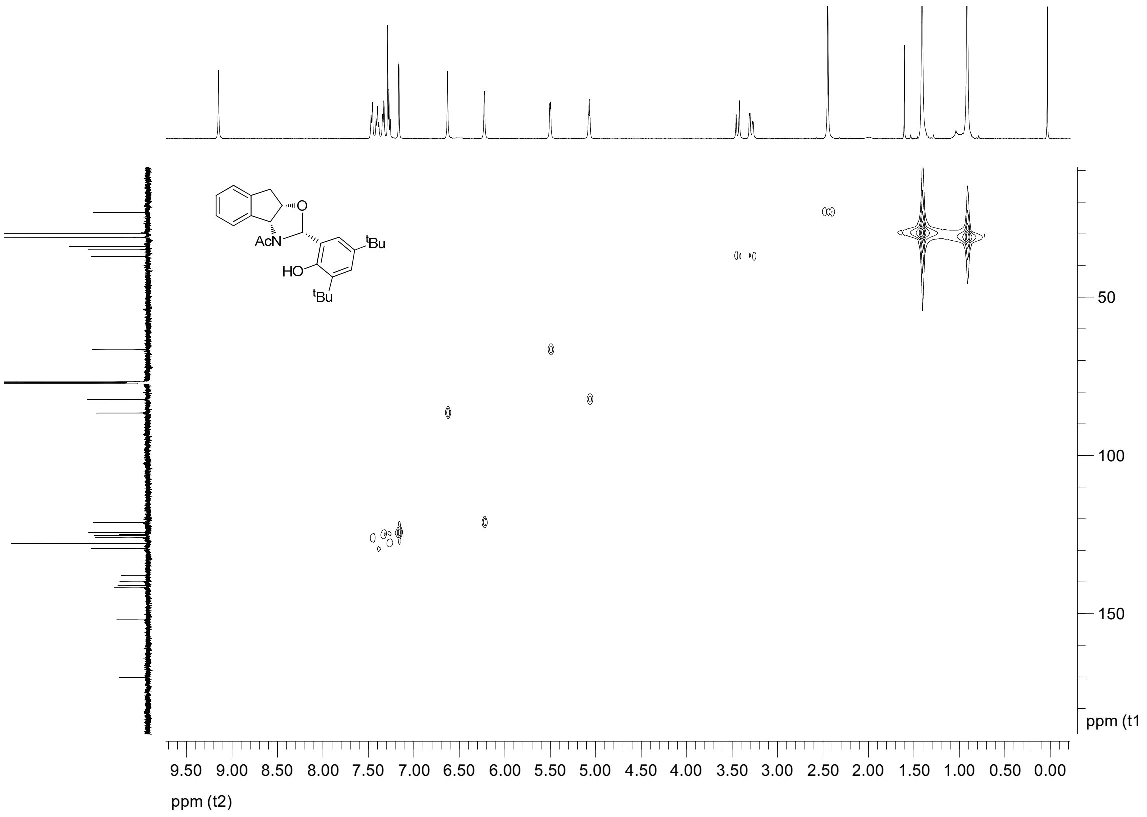

Figure S78. HMQC spectrum of **53** in $CDCl_3$.

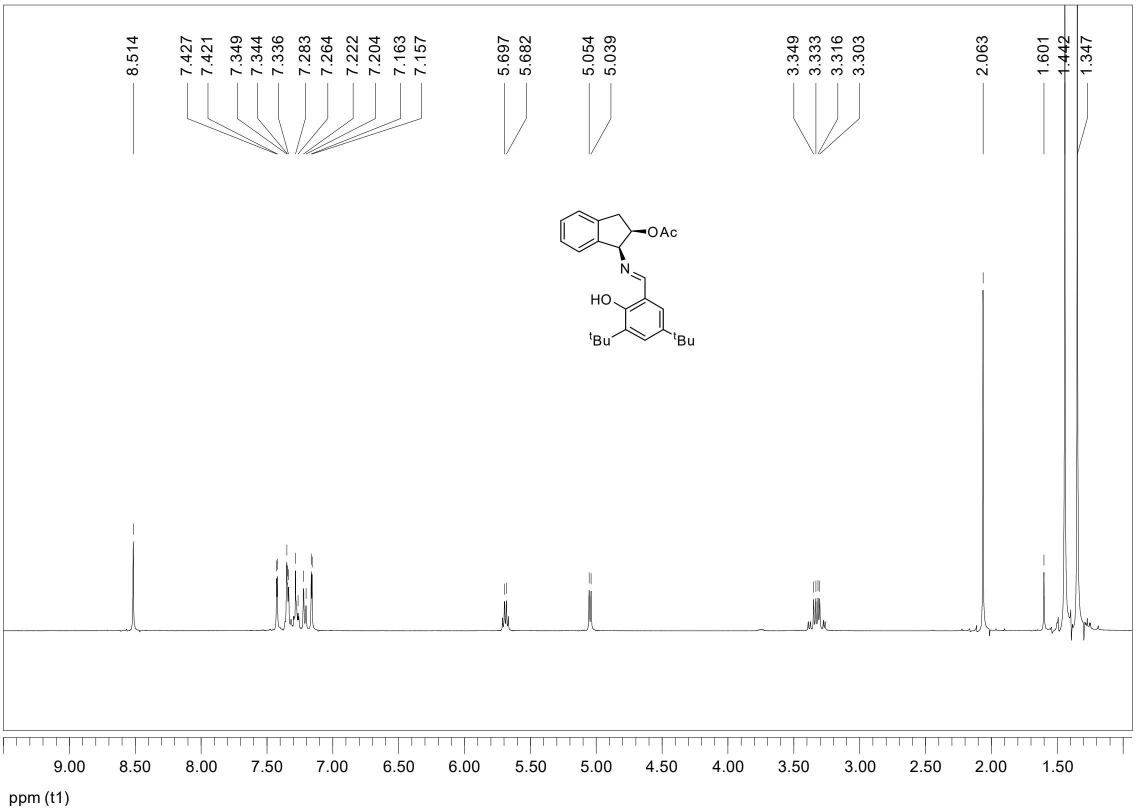

Figure S79. ^{1}H NMR spectrum of **54** in $CDCl_3$.

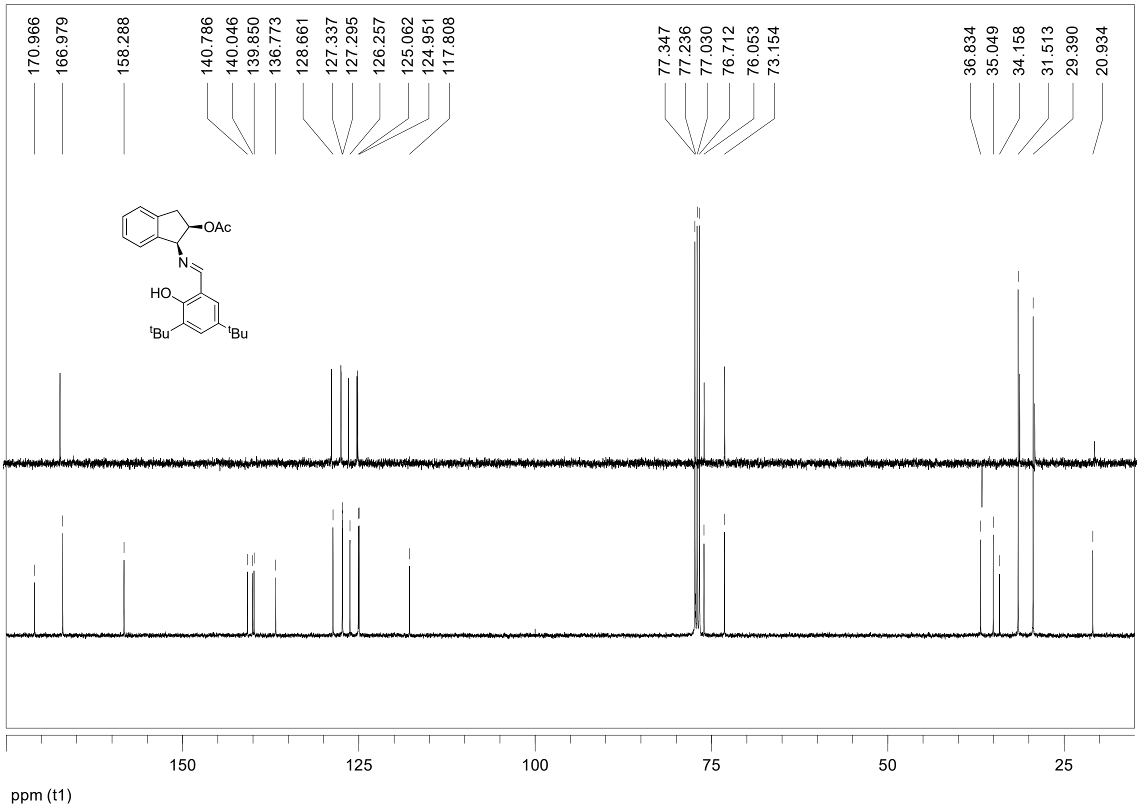

Figure S80. ^{13}C NMR and DEPT spectra of **54** in $CDCl_3$.

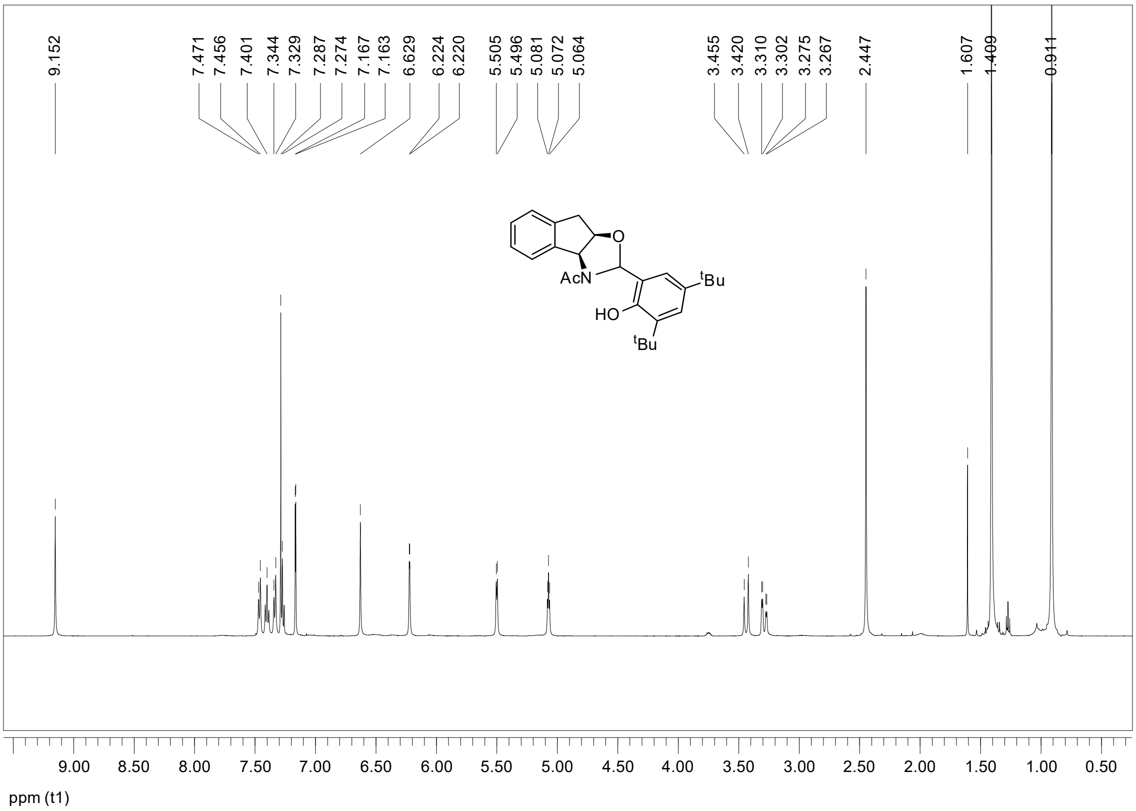

Figure S81. ^{1}H NMR spectrum of **55** in $CDCl_3$.

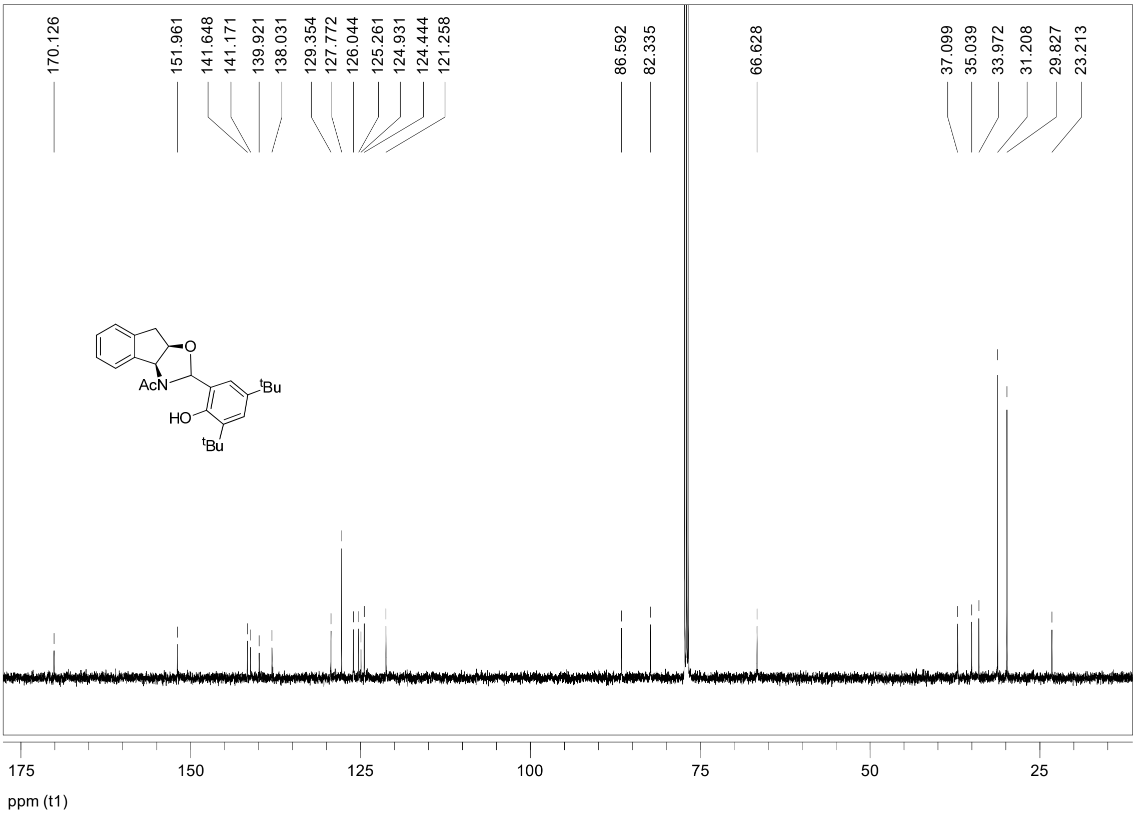

Figure S82. ^{13}C NMR spectrum of **55** in $CDCl_3$.

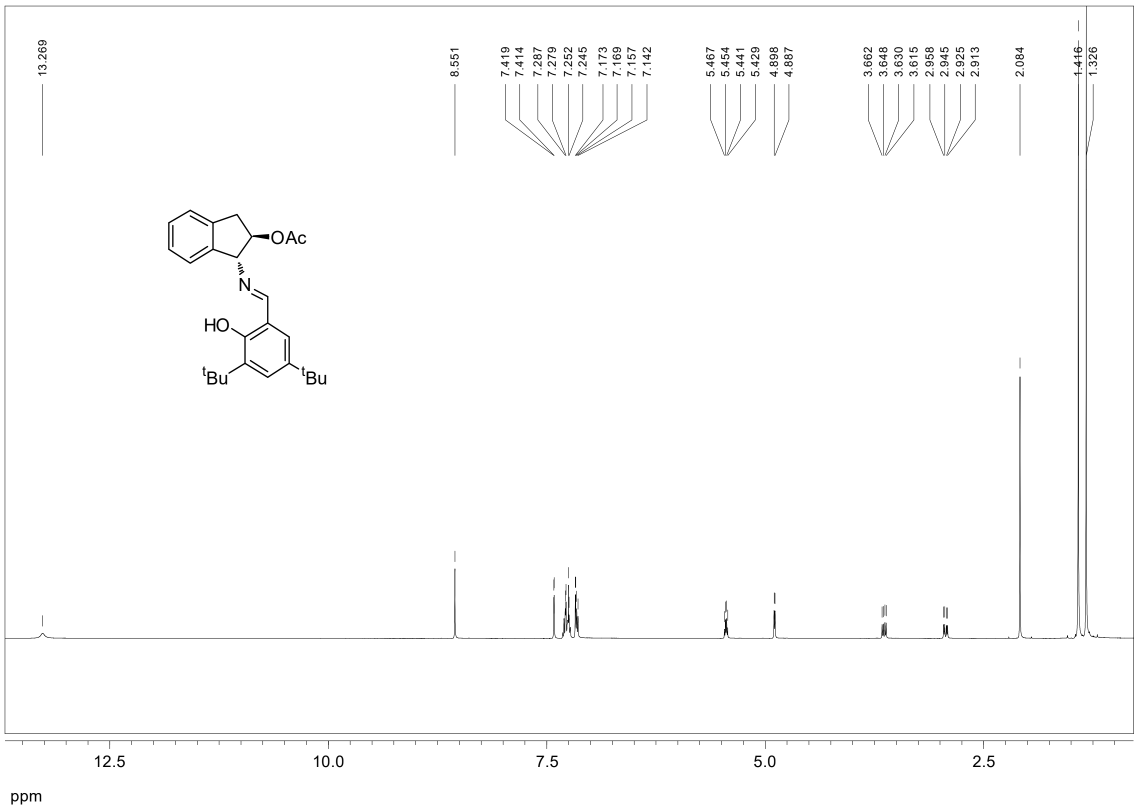

Figure S83. ^{1}H NMR spectrum of **56** in $CDCl_3$.

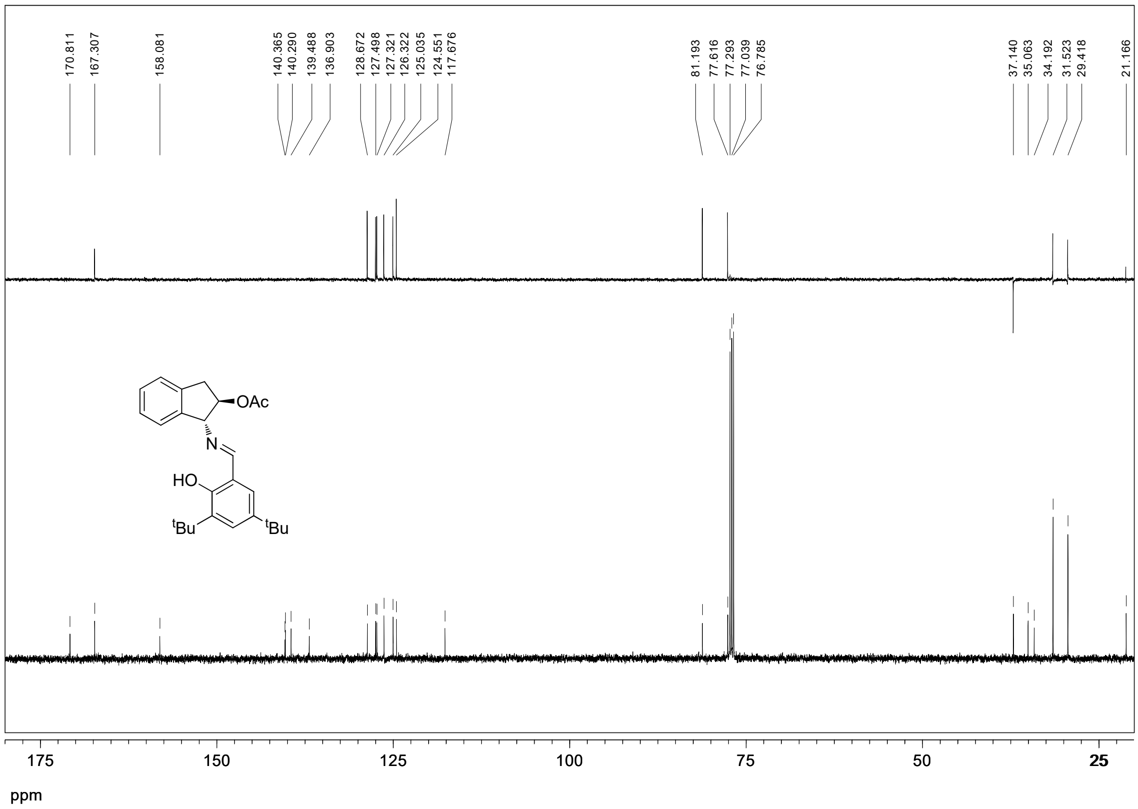

Figure S84. ^{13}C NMR and DEPT spectra of **56** in $CDCl_3$.

Table S12. Crystal data and structure refinement details for **24**

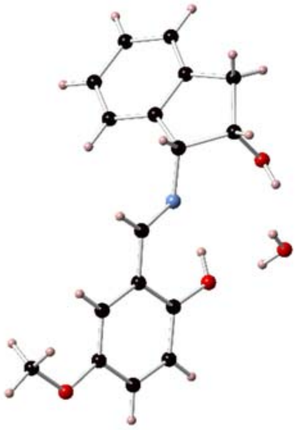

Identification code	**2012acc0034 972**
Empirical formula	$C_{17}H_{19}NO_4$
Formula weight	301.33
Temperature	100(2) K
Wavelength	0.71075 Å
Crystal system	Orthorhombic
Space group	$P2_12_12_1$
Unit cell dimensions	a = 4.507(3) Å
	b = 12.978(8) Å
	c = 25.184(15) Å
Volume	1472.9(15) $Å^3$
Z	4
Density (calculated)	1.359 Mg / m^3
Absorption coefficient	0.097 mm^{-1}
F(000)	640
Crystal	Needle; colourless
Crystal size	0.10 × 0.01 × 0.01 mm^3
θ range for data collection	3.14 − 25.01°
Index ranges	$-5 \le h \le 5$, $-15 \le k \le 11$, $-29 \le l \le 29$
Reflections collected	7279
Independent reflections	1565 [R_{int} = 0.1113]
Completeness to θ = 25.01°	99.7 %
Absorption correction	Semi−empirical from equivalents
Max. and min. transmission	0.9990 and 0.9904
Data / restraints / parameters	1565 / 10 / 208
Goodness-of-fit on F^2	1.202
Final R indices [$F^2 > 2\sigma(F^2)$]	$R1$ = 0.0927, $wR2$ = 0.1498
R indices (all data)	$R1$ = 0.1147, $wR2$ = 0.1575
Largest diff. peak and hole	0.325 and −0.289 e $Å^{-3}$

Crystal data have been deposited with the Cambridge Structural Database having registry numbers: CCDC-2224687 (**24**).

Table S13. Crystal data and structure refinement details for **40**

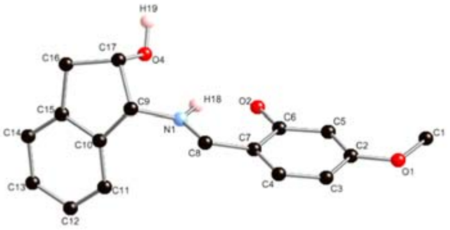

Identification code	**2012acc0035** (986)	
Empirical formula	$C_{17}H_{17}NO_3$	
Formula weight	283.32	
Temperature	100(2) K	
Wavelength	0.71075 Å	
Crystal system	monoclinic	
Space group	$P2_1$	
Unit cell dimensions	a = 12.011(2) Å	
	b = 4.6740(8) Å	β = 93.392(14)°
	c = 12.627(3) Å	
Volume	707.6(2) $Å^3$	
Z	2	
Density (calculated)	1.330 Mg / m^3	
Absorption coefficient	0.091 mm^{-1}	
$F(000)$	300	
Crystal	Fragment; Yellow	
Crystal size	0.20 × 0.20 × 0.20 mm^3	
θ range for data collection	3.23 – 27.48°	
Index ranges	$-15 \le h \le 12$, $-6 \le k \le 5$, $-16 \le l \le 12$	
Reflections collected	3637	
Independent reflections	1802 [R_{int} = 0.0260]	
Completeness to θ = 27.48°	99.1 %	
Absorption correction	Semi–empirical from equivalents	
Max. and min. transmission	0.9820 and 0.9820	
Refinement method	Full-matrix least-squares on F^2	
Data / restraints / parameters	1802 / 1 / 199	
Goodness-of-fit on F^2	1.066	
Final R indices [$F^2 > 2\sigma(F^2)$]	$R1$ = 0.0450, $wR2$ = 0.0889	
R indices (all data)	$R1$ = 0.0536, $wR2$ = 0.0927	
Largest diff. peak and hole	0.163 and –0.195 e $Å^{-3}$	

Crystal data have been deposited with the Cambridge Structural Database having registry numbers: CCDC-2224688 (**40**).

Table S14. Crystal data and structure refinement details for **41**

**Compound**	**2015acc0021-R-100K**
Formula	$C_{16}H_{13}N_3O_6$
$D_{calc.}$/ g cm^{-3}	1.542
□/mm^{-1}	0.121
Formula Weight	343.29
Colour	clear light yellow
Shape	plate
Max Size/mm	0.28
Mid Size/mm	0.16
Min Size/mm	0.01
T/K	100(2)
Crystal System	triclinic
Flack Parameter	-0.28(18)
Hooft Parameter	-0.35(14)
Space Group	P1
a/Å	7.3378(3)
b/Å	8.2964(2)
c/Å	12.2576(5)
α/°	89.000(3)
β/°	83.332(3)
γ/°	85.958(3)
V/Å^3	739.28(5)
Z	2
Z'	2
Θ_{min}/°	2.963
Θ_{max}/°	28.700
Measured Refl.	17072
Independent Refl.	7531
Reflections Used	7192
R_{int}	0.0174
Parameters	467
Restraints	3
Largest Peak	0.289
Deepest Hole	-0.156
GooF	1.035
wR_2 (all data)	0.0807
wR_2	0.0796
R_1 (all data)	0.0317
R_1	0.0299

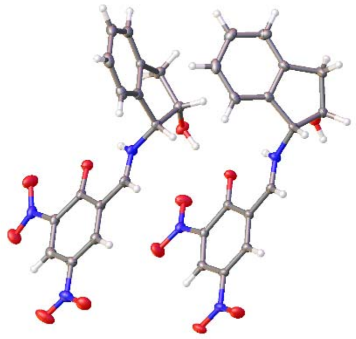

Crystal data have been deposited with the Cambridge Structural Database having registry numbers: CCDC-2224693 (**41**).

Table S15. Crystal data and structure refinement for **42**

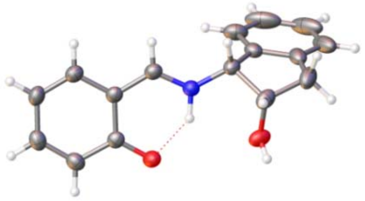

Identification code	estmc43_0m
Empirical formula	$C_{16}H_{15}No_2$
Formula weight	253.29
Temperature/K	443(2)
Crystal system	orthorhombic
Space group	$P2_12_12_1$
a/Å	4.6767(3)
b/Å	11.2227(8)
c/Å	24.5503(18)
α/°	90.00
β/°	90.00
γ/°	90.00
Volume/Å^3	1288.53(16)
Z	4
ρ_{calc}mg/mm^3	1.306
m/mm^{-1}	0.086
F(000)	536.0
Crystal size/mm^3	0.15 × 0.08 × 0.04
2Θ range for data collection	3.32 to 51.04°
Index ranges	-5 ≤ h ≤ 4, -10 ≤ k ≤ 13, -29 ≤ l ≤ 17
Reflections collected	5006
Independent reflections	2293[R(int) = 0.0210]
Data/restraints/parameters	2293/0/174
Goodness-of-fit on F^2	1.122
Final R indexes [I>=2σ (I)]	R_1 = 0.0411, wR_2 = 0.1192
Final R indexes [all data]	R_1 = 0.0486, wR_2 = 0.1387
Largest diff. peak/hole / e Å^{-3}	0.31/-0.40

Table S16. Crystal data and structure refinement for **47.**

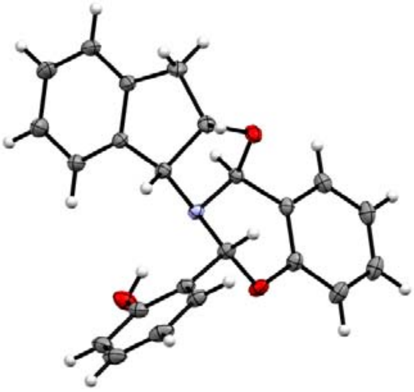

Identification code	Bisoxa_0m.cif
Empirical formula	$C_{23}H_{19}No_3$
Formula weight	357.39
Temperature/K	393(2)
Crystal system	orthorhombic
Space group	$P2_12_12_1$
a/Å	8.3784(2)
b/Å	10.9973(2)
c/Å	19.1289(4)
α/°	90.00
β/°	90.00
γ/°	90.00
Volume/Å^3	1762.53(6)
Z	4
ρ_{calc}mg/mm^3	1.347
m/mm^{-1}	0.089
F(000)	752.0
Crystal size/mm^3	0.27 × 0.16 × 0.15
2Θ range for data collection	4.26 to 60.14°
Index ranges	-11 ≤ h ≤ 11, -9 ≤ k ≤ 15, -26 ≤ l ≤ 26
Reflections collected	13665
Independent reflections	5005[R(int) = 0.0166]
Data/restraints/parameters	5005/0/261
Goodness-of-fit on F^2	1.059
Final R indexes [I>=2σ (I)]	R_1 = 0.0339, wR_2 = 0.0929
Final R indexes [all data]	R_1 = 0.0370, wR_2 = 0.0990
Largest diff. peak/hole / e Å^{-3}	0.42/-0.38
Flack parameter	0.2(7)

Table S17. Crystal data and structure refinement details for **52**

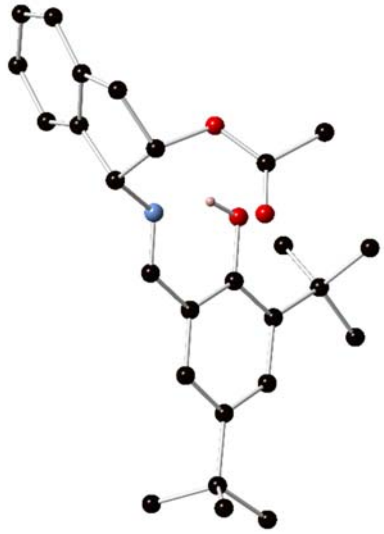

Identification code	**2012acc0036 39**
Empirical formula	$C_{26}H_{33}NO_3$
Formula weight	407.53
Temperature	100(2) K
Wavelength	0.71075 Å
Crystal system	ORTHORHOMBIC
Space group	$P2_12_12_1$
Unit cell dimensions	a = 8.777(2) Å
	b = 14.516(3) Å
	c = 17.743(4) Å
Volume	2260.7(9) $Å^3$
Z	4
Density (calculated)	1.197 Mg / m^3
Absorption coefficient	0.077 mm^{-1}
F(000)	880
Crystal	Prism; Yellow
Crystal size	0.30 × 0.09 × 0.08 mm^3
θ range for data collection	3.03 – 27.48°
Index ranges	$-5 \leq h \leq 11$, $-18 \leq k \leq 11$, $-14 \leq l \leq 22$
Reflections collected	5928
Independent reflections	2898 [R_{int} = 0.0350]
Completeness to θ = 27.48°	98.6 %
Absorption correction	Semi–empirical from equivalents
Max. and min. transmission	0.9938 and 0.9772
Refinement method	Full-matrix least-squares on F^2
Data / restraints / parameters	2898 / 0 / 279
Goodness-of-fit on F^2	1.155
Final R indices [$F^2 > 2\sigma(F^2)$]	$R1$ = 0.0535, $wR2$ = 0.1005
R indices (all data)	$R1$ = 0.0588, $wR2$ = 0.1037
Largest diff. peak and hole	0.257 and –0.256 e $Å^{-3}$

Crystal data have been deposited with the Cambridge Structural Database having registry numbers: CCDC-2224690 (**52**).

Table S18. Crystal data and structure refinement details for **53**

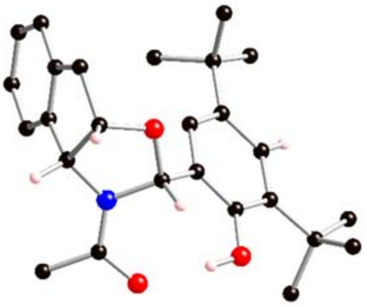

Identification code	**2009acc0653 40**	
Empirical formula	$C_{26}H_{33}NO_3$	
Formula weight	407.53	
Temperature	120(2) K	
Wavelength	0.71073 Å	
Crystal system	Monoclinic	
Space group	$P2_1$	
Unit cell dimensions	a = 9.7810(4) Å	
	b = 11.1760(4) Å	β = 100.938(2)°
	c = 10.4403(4) Å	
Volume	1120.52(7) $Å^3$	
Z	2	
Density (calculated)	1.208 Mg / m^3	
Absorption coefficient	0.078 mm^{-1}	
$F(000)$	440	
Crystal	Fragment; Colourless	
Crystal size	0.4 × 0.2 × 0.13 mm^3	
θ range for data collection	3.17 – 25.03°	
Index ranges	$-11 \le h \le 11$, $-13 \le k \le 13$, $-12 \le l \le 10$	
Reflections collected	10456	
Independent reflections	2096 [R_{int} = 0.0624]	
Completeness to θ = 25.03°	99.8 %	
Absorption correction	Semi–empirical from equivalents	
Max. and min. transmission	0.9899 and 0.9595	
Refinement method	Full-matrix least-squares on F^2	
Data / restraints / parameters	2096 / 1 / 280	
Goodness-of-fit on F^2	1.214	
Final R indices [$F^2 > 2\sigma(F^2)$]	$R1$ = 0.0584, $wR2$ = 0.1597	
R indices (all data)	$R1$ = 0.0676, $wR2$ = 0.1649	
Extinction coefficient	0.032(7)	
Largest diff. peak and hole	0.399 and –0.228 e $Å^{-3}$	

Crystal data have been deposited with the Cambridge Structural Database having registry numbers: CCDC-2010159 (**53**).

Structure 20 (M06-2X, Gas Phase)

Energy (Hartrees): =-479.4706265
No imaginary frequencies

Standard orientation:

Center Number	Atomic Number	Atomic Type	Coordinates (Angstroms) X	Y	Z
1	6	0	-1.747175	-1.387934	0.282242
2	6	0	-2.884272	-0.596399	0.118848
3	6	0	-2.770207	0.752541	-0.201850
4	6	0	-1.512703	1.334370	-0.364548
5	6	0	-0.385669	0.547078	-0.197956
6	6	0	-0.497983	-0.808286	0.126049
7	6	0	1.072977	0.936272	-0.282015
8	6	0	1.717360	-0.427717	-0.586081
9	6	0	0.876338	-1.421925	0.236173
10	1	0	-1.843264	-2.440885	0.523706
11	1	0	-3.866995	-1.038284	0.236226
12	1	0	-3.663976	1.350689	-0.333956
13	1	0	-1.422586	2.383412	-0.626859
14	1	0	1.272519	1.658005	-1.085414
15	1	0	1.570134	-0.638935	-1.653919
16	1	0	0.928868	-2.447506	-0.136534
17	1	0	1.223286	-1.394272	1.273418
18	1	0	2.542981	1.473107	1.018524
19	1	0	1.123008	2.265814	1.273114
20	1	0	3.454613	-1.266895	-0.357099
21	7	0	1.533634	1.370069	1.036940
22	8	0	3.091747	-0.382842	-0.271869

Structure 20 (M06-2X, EtOH)

Energy (Hartrees): =-479.491982
No imaginary frequencies

Standard orientation:

Center Number	Atomic Number	Atomic Type	Coordinates (Angstroms) X	Y	Z
1	6	0	-1.753097	-1.392232	0.282453
2	6	0	-2.887332	-0.593988	0.124563
3	6	0	-2.767737	0.757006	-0.194678
4	6	0	-1.507416	1.333474	-0.362920
5	6	0	-0.382283	0.537644	-0.205414
6	6	0	-0.499221	-0.818334	0.119930
7	6	0	1.075257	0.927625	-0.298087
8	6	0	1.727837	-0.440362	-0.567156
9	6	0	0.870527	-1.441551	0.228555
10	1	0	-1.853803	-2.445379	0.523762
11	1	0	-3.872317	-1.031501	0.245585
12	1	0	-3.659703	1.360378	-0.320811
13	1	0	-1.412576	2.383819	-0.620241
14	1	0	1.269136	1.628763	-1.118331
15	1	0	1.618242	-0.658601	-1.636579
16	1	0	0.922071	-2.458095	-0.165865
17	1	0	1.207693	-1.454013	1.270112
18	1	0	2.522365	1.619566	0.951562
19	1	0	1.072902	2.323896	1.182859
20	1	0	3.488009	-1.254030	-0.442036
21	7	0	1.524925	1.430814	1.006932
22	8	0	3.095537	-0.405774	-0.210253

Structure 24/31 imine (M06-2X, Gas Phase)

Energy (Hartrees): = -938.3064563
No imaginary frequencies

Standard orientation:

Center Number	Atomic Number	Atomic Type	Coordinates (Angstroms) X	Y	Z
1	7	0	0.396095	0.427591	-0.278859
2	8	0	2.232413	2.343859	-0.198481
3	1	0	2.761474	3.129894	-0.350826
4	6	0	-1.967257	0.150876	-0.065878
5	6	0	-0.683019	-0.087832	-0.714946
6	1	0	-0.690349	-0.731168	-1.606782
7	6	0	-3.114056	-0.500534	-0.558657
8	1	0	-2.997844	-1.169945	-1.402362
9	6	0	-2.067534	1.031831	1.024696

10	6	0	-3.329573	1.236922	1.602267
11	1	0	-3.397564	1.917884	2.441300
12	6	0	-4.443010	0.590212	1.114933
13	1	0	-5.419050	0.743683	1.559465
14	6	0	1.633680	0.177103	-0.968464
15	1	0	1.457429	-0.365235	-1.912912
16	6	0	2.646308	-0.590977	-0.143855
17	6	0	-4.347922	-0.289185	0.026274
18	6	0	3.931187	-0.131751	-0.422315
19	6	0	2.429310	-1.623245	0.752598
20	1	0	1.421731	-1.953880	0.979682
21	6	0	2.404678	1.477003	-1.298679
22	1	0	2.029829	1.936282	-2.220419
23	6	0	3.867572	1.003445	-1.417228
24	1	0	4.072006	0.642628	-2.431692
25	1	0	4.573141	1.810275	-1.202922
26	6	0	4.820349	-1.759950	1.090350
27	1	0	5.667701	-2.220801	1.584170
28	6	0	3.532635	-2.207902	1.372326
29	1	0	3.387718	-3.009673	2.086451
30	6	0	5.029203	-0.715206	0.190857
31	1	0	6.033305	-0.359847	-0.013490
32	8	0	-1.025359	1.691588	1.540309
33	1	0	-0.216249	1.442062	1.035539
34	8	0	-5.515801	-0.871649	-0.369297
35	6	0	-5.448682	-1.773975	-1.448866
36	1	0	-6.461926	-2.136907	-1.606052
37	1	0	-5.095391	-1.279294	-2.360609
38	1	0	-4.791856	-2.620484	-1.219241

Structure 24/31 imine (M06-2X, DMSO)

Energy (Hartrees): =-938.3334279
No imaginary frequencies

Standard orientation:

Center Number	Atomic Number	Atomic Type	Coordinates (Angstroms) X	Y	Z
1	7	0	-0.388482	-0.416563	-0.285606
2	8	0	-2.223221	-2.349211	-0.143594
3	1	0	-2.766555	-3.131057	-0.287186
4	6	0	1.968924	-0.142888	-0.070295
5	6	0	0.690959	0.079270	-0.747124
6	1	0	0.706330	0.682759	-1.662271
7	6	0	3.127380	0.471332	-0.578297
8	1	0	3.029606	1.096673	-1.457896
9	6	0	2.043987	-0.969888	1.065001
10	6	0	3.290982	-1.158705	1.674557
11	1	0	3.348117	-1.795982	2.549374
12	6	0	4.421198	-0.544940	1.172905
13	1	0	5.384781	-0.690614	1.648109
14	6	0	-1.631098	-0.194866	-0.984322
15	1	0	-1.459133	0.319029	-1.941661
16	6	0	-2.641036	0.590913	-0.172031
17	6	0	4.352556	0.278645	0.039619
18	6	0	-3.926578	0.111081	-0.425570
19	6	0	-2.425795	1.650468	0.695046
20	1	0	-1.421174	2.009479	0.894663
21	6	0	-2.397159	-1.506582	-1.271000
22	1	0	-2.027680	-1.993167	-2.178406
23	6	0	-3.860619	-1.044993	-1.394869
24	1	0	-4.063155	-0.702462	-2.415443
25	1	0	-4.562013	-1.850944	-1.166571
26	6	0	-4.815953	1.768283	1.061319
27	1	0	-5.663139	2.232380	1.553802
28	6	0	-3.529443	2.238909	1.315553
29	1	0	-3.384714	3.062763	2.005085
30	6	0	-5.024119	0.698833	0.187826
31	1	0	-6.027162	0.328807	0.002802
32	8	0	0.972425	-1.587979	1.584947
33	1	0	0.189999	-1.334472	1.025911
34	8	0	5.525844	0.832952	-0.367718
35	6	0	5.482610	1.657544	-1.520355
36	1	0	6.498973	2.013615	-1.677132
37	1	0	5.156276	1.093710	-2.400189
38	1	0	4.818841	2.514845	-1.370626

Structure 24/31 enamine (M06-2X, Gas Phase)

Energy (Hartrees): =-938.2964518
No imaginary frequencies

Standard orientation:

Center Number	Atomic Number	Atomic Type	Coordinates (Angstroms) X	Y	Z
1	7	0	0.414402	0.558444	-0.367761
2	8	0	2.332570	2.344154	-0.095586
3	1	0	2.728784	3.204360	-0.247106
4	6	0	-1.951813	0.190008	-0.100648
5	6	0	-0.722867	-0.035407	-0.700923
6	1	0	-0.656291	-0.754878	-1.516863
7	6	0	-3.110583	-0.525314	-0.567458
8	1	0	-2.983863	-1.227936	-1.382700
9	6	0	-2.045506	1.152900	0.991960
10	6	0	-3.377453	1.319070	1.558875
11	1	0	-3.472756	2.027010	2.372920
12	6	0	-4.439272	0.625397	1.089987
13	1	0	-5.428617	0.754103	1.516096
14	6	0	1.682672	0.264591	-0.984423
15	1	0	1.489241	-0.228976	-1.944606
16	6	0	2.625196	-0.592239	-0.161214
17	6	0	-4.320249	-0.314861	0.010628
18	6	0	3.938820	-0.181818	-0.367494
19	6	0	2.320874	-1.648635	0.680235
20	1	0	1.289887	-1.937236	0.855440
21	6	0	2.526672	1.533503	-1.236052
22	1	0	2.210666	2.053214	-2.146185
23	6	0	3.972296	1.003347	-1.305667
24	1	0	4.217247	0.688206	-2.326160
25	1	0	4.692159	1.769013	-1.007823
26	6	0	4.686557	-1.913955	1.105590
27	1	0	5.490209	-2.434514	1.613050
28	6	0	3.368648	-2.312044	1.315861
29	1	0	3.156837	-3.135811	1.986739
30	6	0	4.981895	-0.842859	0.263654
31	1	0	6.008724	-0.525676	0.118807
32	8	0	-1.069015	1.791704	1.416925
33	1	0	0.361850	1.239727	0.399226
34	8	0	-5.495830	-0.920563	-0.328902
35	6	0	-5.439888	-1.856849	-1.377564
36	1	0	-6.451649	-2.234154	-1.508650
37	1	0	-5.101262	-1.388502	-2.309058
38	1	0	-4.769768	-2.688427	-1.130394

Structure 24/31 enamine (M06-2X, DMSO)

Energy (Hartrees): =-938.3282616
No imaginary frequencies

Standard orientation:

Center Number	Atomic Number	Atomic Type	Coordinates (Angstroms) X	Y	Z
1	7	0	-0.423099	-0.617248	-0.437520
2	8	0	-2.359708	-2.350061	-0.052886
3	1	0	-2.871729	-3.160355	-0.142368
4	6	0	1.932044	-0.245556	-0.125985
5	6	0	0.698398	-0.007280	-0.747230
6	1	0	0.637029	0.733684	-1.543124
7	6	0	3.073781	0.504426	-0.563399
8	1	0	2.933632	1.219837	-1.365510
9	6	0	2.026754	-1.221919	0.942468
10	6	0	3.349160	-1.368713	1.520609
11	1	0	3.461378	-2.088486	2.323753
12	6	0	4.410304	-0.641619	1.080934
13	1	0	5.391708	-0.768503	1.527901
14	6	0	-1.704407	-0.303451	-1.031650
15	1	0	-1.527601	0.157809	-2.008157
16	6	0	-2.579205	0.602482	-0.185776
17	6	0	4.288830	0.313576	0.024811
18	6	0	-3.912383	0.215015	-0.310251
19	6	0	-2.207575	1.682463	0.598903
20	1	0	-1.164345	1.966879	0.695743
21	6	0	-2.588905	-1.554178	-1.206507
22	1	0	-2.337908	-2.107126	-2.114803
23	6	0	-4.019945	-0.989490	-1.215848
24	1	0	-4.297812	-0.685977	-2.230562
25	1	0	-4.746551	-1.729517	-0.873805
26	6	0	-4.541959	2.011023	1.147579

27	1	0	-5.307233	2.565803	1.678734
28	6	0	-3.206049	2.389294	1.270486
29	1	0	-2.941133	3.235018	1.894722
30	6	0	-4.905547	0.919873	0.356328
31	1	0	-5.946046	0.623892	0.274187
32	8	0	1.050264	-1.895750	1.347121
33	1	0	-0.370389	-1.313775	0.314405
34	8	0	5.447352	0.959214	-0.295279
35	6	0	5.364140	1.929098	-1.322044
36	1	0	6.365348	2.339918	-1.439166
37	1	0	5.043863	1.480087	-2.268374
38	1	0	4.671595	2.734071	-1.053151

Structure 24/31 TS (M06-2X, Gas Phase)

Energy (Hartrees): =-938.2932058
Imaginary frequency -966.78

Standard orientation:

Center Number	Atomic Number	Atomic Type	Coordinates (Angstroms) X	Y	Z
1	7	0	-0.360584	-0.400735	-0.319369
2	8	0	-2.130444	-2.320342	0.112766
3	1	0	-2.568229	-3.173456	0.084339
4	6	0	1.966818	-0.084929	-0.121704
5	6	0	0.747026	0.067307	-0.819685
6	1	0	0.722308	0.578210	-1.785600
7	6	0	3.188815	0.420902	-0.648167
8	1	0	3.165421	0.938394	-1.600069
9	6	0	1.916381	-0.781251	1.132742
10	6	0	3.165056	-0.933675	1.820172
11	6	0	4.321227	-0.438592	1.294949
12	6	0	-1.635501	-0.288198	-0.976208
13	1	0	-1.480701	0.121104	-1.984548
14	6	0	-2.652391	0.563789	-0.244023
15	6	0	4.351745	0.247653	0.049844
16	6	0	-3.926903	0.030574	-0.417060
17	6	0	-2.446090	1.719092	0.489909
18	1	0	-1.444642	2.108096	0.638970
19	6	0	-2.370112	-1.642433	-1.100046
20	1	0	-2.001948	-2.220846	-1.954569
21	6	0	-3.850814	-1.236445	-1.237830
22	1	0	-4.096509	-1.036151	-2.286849
23	1	0	-4.519503	-2.023814	-0.881942
24	6	0	-4.831118	1.831058	0.874838
25	1	0	-5.680385	2.331295	1.324948
26	6	0	-3.552396	2.353369	1.051874
27	1	0	-3.416643	3.253972	1.638446
28	6	0	-5.027931	0.662882	0.139956
29	1	0	-6.024082	0.251187	0.019774
30	8	0	0.824468	-1.239589	1.608727
31	1	0	-0.102288	-0.915457	0.688339
32	1	0	5.265710	-0.554204	1.814980
33	1	0	3.150687	-1.456570	2.768193
34	8	0	5.589398	0.679136	-0.335341
35	6	0	5.666002	1.352300	-1.568352
36	1	0	6.712307	1.613065	-1.712084
37	1	0	5.335074	0.709780	-2.392588
38	1	0	5.061251	2.266476	-1.560620

Structure 24/31 TS (M06-2X, DMSO)

Energy (Hartrees): =-938.3222567
Imaginary frequency -919.20

Standard orientation:

Center Number	Atomic Number	Atomic Type	Coordinates (Angstroms) X	Y	Z
1	7	0	-0.432381	-0.466811	-0.263905
2	8	0	-2.141628	-2.157231	0.890123
3	1	0	-2.607740	-2.955378	1.160240
4	6	0	1.896014	-0.130757	-0.289790
5	6	0	0.637451	-0.263507	-0.960004
6	1	0	0.576393	-0.192629	-2.046816
7	6	0	3.077017	0.087160	-1.021134
8	1	0	3.036945	0.154530	-2.103932
9	6	0	1.904212	-0.227281	1.141404

10	6	0	3.171713	-0.088965	1.763635
11	6	0	4.322190	0.123822	1.030455
12	6	0	-1.742825	-0.603134	-0.855601
13	1	0	-1.641451	-0.560103	-1.946242
14	6	0	-2.736503	0.443193	-0.393768
15	6	0	4.288831	0.214574	-0.377118
16	6	0	-4.006535	-0.127787	-0.309354
17	6	0	-2.524293	1.782154	-0.106679
18	1	0	-1.529296	2.211636	-0.165888
19	6	0	-2.447354	-1.921951	-0.473390
20	1	0	-2.109141	-2.753268	-1.097877
21	6	0	-3.938760	-1.594447	-0.664334
22	1	0	-4.226958	-1.751537	-1.709140
23	1	0	-4.575937	-2.225908	-0.041045
24	6	0	-4.891803	2.000579	0.348806
25	1	0	-5.732564	2.617680	0.645240
26	6	0	-3.618867	2.562352	0.269927
27	1	0	-3.477012	3.610802	0.505705
28	6	0	-5.094888	0.649698	0.060668
29	1	0	-6.086329	0.215769	0.136623
30	8	0	0.817284	-0.427140	1.810280
31	1	0	-0.122840	-0.514029	0.857935
32	1	0	3.217688	-0.154761	2.844794
33	1	0	5.262648	0.222062	1.559377
34	8	0	5.390608	0.422237	-1.158978
35	6	0	6.636886	0.565046	-0.501906
36	1	0	7.375219	0.724646	-1.286000
37	1	0	6.639841	1.427551	0.172549
38	1	0	6.902183	-0.336810	0.059514

Structure 29/34 imine (M06-2X, Gas Phase)

Energy (Hartrees): = -823.7996565
No imaginary frequencies

Standard orientation:

Center Number	Atomic Number	Atomic Type	Coordinates (Angstroms) X	Y	Z
1	7	0	0.255749	0.406734	0.279003
2	8	0	-1.338077	2.209971	-0.856182
3	1	0	-1.780070	3.019025	-1.122681
4	6	0	2.572577	-0.127851	0.518804
5	6	0	1.217545	0.044467	1.029994
6	1	0	1.066477	-0.149670	2.101699
7	6	0	3.583813	-0.580409	1.375032
8	1	0	3.327905	-0.800831	2.406977
9	6	0	2.883784	0.169218	-0.827837
10	6	0	4.200808	-0.001879	-1.269830
11	1	0	4.420253	0.231334	-2.304325
12	6	0	5.179060	-0.454844	-0.403475
13	1	0	6.191563	-0.580556	-0.769382
14	6	0	-1.058451	0.571515	0.841856
15	1	0	-1.028990	0.489563	1.941611
16	6	0	-2.080180	-0.411332	0.306887
17	6	0	4.881004	-0.749175	0.930250
18	1	0	5.652621	-1.102164	1.601715
19	6	0	-3.322393	0.209180	0.199200
20	6	0	-1.910343	-1.744711	-0.023352
21	1	0	-0.932523	-2.209120	0.043632
22	6	0	-1.698125	1.932150	0.479870
23	1	0	-1.349617	2.725190	1.151202
24	6	0	-3.210232	1.659163	0.609223
25	1	0	-3.535862	1.795428	1.646731
26	1	0	-3.799747	2.334673	-0.015895
27	6	0	-4.265194	-1.854463	-0.563768
28	1	0	-5.116857	-2.427262	-0.911389
29	6	0	-3.018709	-2.466641	-0.463177
30	1	0	-2.908913	-3.509192	-0.736524
31	6	0	-4.425777	-0.509056	-0.234598
32	1	0	-5.396162	-0.033826	-0.329277
33	8	0	1.977968	0.612590	-1.699489
34	1	0	1.105438	0.677773	-1.242218

Structure 29/34 imine (M06-2X, DMSO)

Energy (Hartrees): =-823.8247083
No imaginary frequencies

Standard orientation:

Center Number	Atomic Number	Atomic Type	Coordinates (Angstroms) X	Y	Z

1	7	0	0.262579	-0.389072	-0.297280
2	8	0	-1.317715	-2.205181	0.858298
3	1	0	-1.771642	-3.014435	1.115647
4	6	0	2.575875	0.136011	-0.525679
5	6	0	1.223269	-0.031939	-1.054741
6	1	0	1.078792	0.156631	-2.125112
7	6	0	3.600550	0.585667	-1.366483
8	1	0	3.358713	0.809444	-2.401024
9	6	0	2.867324	-0.167837	0.824371
10	6	0	4.176716	-0.007624	1.289922
11	1	0	4.388458	-0.244652	2.326279
12	6	0	5.172220	0.444430	0.437835
13	1	0	6.180295	0.562763	0.819449
14	6	0	-1.055381	-0.566946	-0.857289
15	1	0	-1.028813	-0.489882	-1.954357
16	6	0	-2.077743	0.413602	-0.316991
17	6	0	4.893919	0.745304	-0.898350
18	1	0	5.678081	1.096318	-1.557334
19	6	0	-3.315265	-0.219165	-0.190711
20	6	0	-1.917547	1.752875	0.001129
21	1	0	-0.949355	2.234145	-0.094315
22	6	0	-1.683622	-1.928830	-0.483776
23	1	0	-1.337136	-2.724004	-1.150449
24	6	0	-3.195842	-1.667589	-0.601525
25	1	0	-3.521539	-1.799921	-1.638860
26	1	0	-3.777812	-2.350075	0.022364
27	6	0	-4.266580	1.842301	0.579900
28	1	0	-5.119552	2.408478	0.937025
29	6	0	-3.027548	2.467521	0.455542
30	1	0	-2.924583	3.514528	0.717034
31	6	0	-4.419202	0.492254	0.257354
32	1	0	-5.384432	0.008598	0.365818
33	8	0	1.931691	-0.610667	1.670574
34	1	0	1.073831	-0.657855	1.164787

Structure 29/34 enamine (M06-2X, Gas Phase)

Energy (Hartrees): =-823.7889998
No imaginary frequencies

Standard orientation:

Center Number	Atomic Number	Atomic Type	Coordinates (Angstroms) X	Y	Z
1	7	0	0.254951	-0.472409	-0.312802
2	8	0	-1.364494	-2.207298	0.837709
3	1	0	-1.672965	-3.080822	1.086053
4	6	0	2.576087	0.128400	-0.513742
5	6	0	1.284284	-0.020320	-1.005120
6	1	0	1.078095	0.251323	-2.040477
7	6	0	3.603327	0.625193	-1.373286
8	1	0	3.343415	0.874649	-2.398599
9	6	0	2.866124	-0.226110	0.876097
10	6	0	4.244337	-0.027579	1.289815
11	1	0	4.480861	-0.280847	2.316272
12	6	0	5.182456	0.448784	0.431038
13	1	0	6.201378	0.580721	0.780687
14	6	0	-1.080571	-0.588737	-0.843358
15	1	0	-1.020283	-0.491836	-1.934076
16	6	0	-2.082733	0.413064	-0.303772
17	6	0	4.875916	0.785520	-0.926966
18	1	0	5.653054	1.161804	-1.578767
19	6	0	-3.333401	-0.187786	-0.191927
20	6	0	-1.885611	1.741277	0.032905
21	1	0	-0.899546	2.188617	-0.033351
22	6	0	-1.744101	-1.939449	-0.495564
23	1	0	-1.410863	-2.736666	-1.167931
24	6	0	-3.251773	-1.640589	-0.602149
25	1	0	-3.596884	-1.775071	-1.633397
26	1	0	-3.838566	-2.303811	0.037369
27	6	0	-4.235373	1.887998	0.585680
28	1	0	-5.074617	2.473654	0.941652
29	6	0	-2.978748	2.479648	0.481925
30	1	0	-2.849116	3.518172	0.761187
31	6	0	-4.421689	0.547554	0.251961
32	1	0	-5.399016	0.088196	0.352208
33	8	0	1.996377	-0.665399	1.647084
34	1	0	0.454147	-0.740870	0.661216

Structure 29/34 enamine (M06-2X, DMSO)

Energy (Hartrees): =-823.8203459
No imaginary frequencies

Standard orientation:

Center Number	Atomic Number	Atomic Type	Coordinates (Angstroms) X	Y	Z
1	7	0	0.250114	-0.458884	-0.344844
2	8	0	-1.310528	-2.202602	0.839462
3	1	0	-1.715002	-3.028177	1.125439
4	6	0	2.574780	0.117892	-0.506793
5	6	0	1.278639	-0.033049	-1.031943
6	1	0	1.095988	0.215683	-2.076130
7	6	0	3.607456	0.579984	-1.367900
8	1	0	3.353240	0.798536	-2.401546
9	6	0	2.842710	-0.194369	0.889792
10	6	0	4.212909	0.004256	1.310164
11	1	0	4.446166	-0.219207	2.345800
12	6	0	5.173193	0.448805	0.446821
13	1	0	6.188635	0.579885	0.808444
14	6	0	-1.088670	-0.597082	-0.877285
15	1	0	-1.030909	-0.516139	-1.966681
16	6	0	-2.082003	0.410514	-0.331682
17	6	0	4.885885	0.746617	-0.915930
18	1	0	5.670735	1.097903	-1.573450
19	6	0	-3.323008	-0.202510	-0.162428
20	6	0	-1.891232	1.752476	-0.042469
21	1	0	-0.917941	2.216007	-0.169975
22	6	0	-1.731849	-1.945715	-0.491587
23	1	0	-1.418093	-2.750923	-1.160448
24	6	0	-3.240930	-1.659269	-0.552972
25	1	0	-3.609084	-1.804350	-1.573854
26	1	0	-3.806284	-2.322107	0.105859
27	6	0	-4.222661	1.885977	0.594885
28	1	0	-5.057133	2.471588	0.963866
29	6	0	-2.977942	2.490975	0.427136
30	1	0	-2.852712	3.540636	0.666731
31	6	0	-4.404296	0.533701	0.301576
32	1	0	-5.373233	0.066463	0.442863
33	8	0	1.955511	-0.604974	1.673797
34	1	0	0.437075	-0.708222	0.634598

Structure 29/34 TS (M06-2X, Gas Phase)

Energy (Hartrees): =-823.7865361
Imaginary frequency -849.69

Standard orientation:

Center Number	Atomic Number	Atomic Type	Coordinates (Angstroms) X	Y	Z
1	7	0	-0.294385	0.406873	-0.362409
2	8	0	1.213098	2.103262	0.989010
3	1	0	1.555675	2.925870	1.344793
4	6	0	-2.589914	-0.077704	-0.581548
5	6	0	-1.303382	0.129413	-1.134467
6	1	0	-1.147323	0.057427	-2.213614
7	6	0	-3.705323	-0.384258	-1.394753
8	1	0	-3.560802	-0.463817	-2.468626
9	6	0	-2.721810	0.042331	0.851027
10	6	0	-4.032136	-0.168167	1.385455
11	6	0	-5.086214	-0.465021	0.564911
12	6	0	1.043031	0.632178	-0.845149
13	1	0	1.025523	0.640970	-1.943830
14	6	0	2.063528	-0.380423	-0.366261
15	6	0	-4.941288	-0.578380	-0.841450
16	6	0	3.286650	0.248857	-0.151036
17	6	0	1.904622	-1.740488	-0.162979
18	1	0	0.938518	-2.211950	-0.307421
19	6	0	1.643816	1.966603	-0.346304
20	1	0	1.307183	2.810771	-0.958013
21	6	0	3.164383	1.729416	-0.428944
22	1	0	3.535869	1.969648	-1.431442
23	1	0	3.707958	2.350849	0.286648
24	6	0	4.237175	-1.859822	0.463956
25	1	0	5.084549	-2.447979	0.795937
26	6	0	3.008067	-2.480915	0.256648
27	1	0	2.907184	-3.544966	0.433145
28	6	0	4.385253	-0.488259	0.263492
29	1	0	5.340906	-0.007675	0.442057
30	8	0	-1.723145	0.319839	1.590646
31	1	0	-0.679723	0.451483	0.721533

32	1	0	-6.066974	-0.618412	1.003172
33	1	0	-4.148648	-0.082354	2.458708
34	1	0	-5.797566	-0.814952	-1.459009

Structure 29/34 TS (M06-2X, DMSO)

Energy (Hartrees): =-823.816505
Imaginary frequency -983.08

Standard orientation:

Center Number	Atomic Number	Atomic Type	Coordinates (Angstroms) X	Y	Z
1	7	0	0.295592	-0.438793	-0.361636
2	8	0	-1.229056	-2.084336	1.067915
3	1	0	-1.646661	-2.863427	1.449592
4	6	0	2.589965	0.025147	-0.580457
5	6	0	1.296490	-0.221477	-1.147729
6	1	0	1.157529	-0.223389	-2.230135
7	6	0	3.708697	0.266884	-1.398236
8	1	0	3.573280	0.261556	-2.475877
9	6	0	2.704329	0.020064	0.850198
10	6	0	3.996770	0.270348	1.387391
11	6	0	5.072865	0.503502	0.560176
12	6	0	-1.046226	-0.681740	-0.836899
13	1	0	-1.033214	-0.736641	-1.931942
14	6	0	-2.046477	0.364404	-0.388166
15	6	0	4.946231	0.505941	-0.844044
16	6	0	-3.276462	-0.243407	-0.136206
17	6	0	-1.871313	1.731308	-0.243337
18	1	0	-0.905425	2.190787	-0.428180
19	6	0	-1.657503	-1.985352	-0.279378
20	1	0	-1.337079	-2.857993	-0.855013
21	6	0	-3.172763	-1.732854	-0.364743
22	1	0	-3.542870	-1.995863	-1.361253
23	1	0	-3.724565	-2.327656	0.366605
24	6	0	-4.197149	1.902541	0.405669
25	1	0	-5.035923	2.513167	0.720566
26	6	0	-2.963405	2.501856	0.159067
27	1	0	-2.849826	3.572443	0.286273
28	6	0	-4.362724	0.523986	0.260450
29	1	0	-5.322984	0.061646	0.463488
30	8	0	1.679238	-0.201676	1.601437
31	1	0	0.707220	-0.395725	0.751087
32	1	0	6.046063	0.689486	1.003337
33	1	0	4.106887	0.270400	2.465928
34	1	0	5.809016	0.693950	-1.470377

Structure 38/43 imine (M06-2X, Gas Phase)

Energy (Hartrees): =-1028.2866862
No imaginary frequencies

Standard orientation:

Center Number	Atomic Number	Atomic Type	Coordinates (Angstroms) X	Y	Z
1	7	0	-0.661197	-0.433508	-0.299975
2	8	0	-2.460954	-2.335602	-0.003385
3	1	0	-2.982316	-3.138973	-0.066858
4	6	0	1.695456	-0.166171	-0.119179
5	6	0	0.412510	0.038463	-0.789293
6	1	0	0.424102	0.607922	-1.728341
7	6	0	2.850464	0.405797	-0.645150
8	1	0	2.813442	1.008600	-1.544525
9	6	0	1.777893	-0.957629	1.055811
10	6	0	3.027517	-1.142225	1.670548
11	1	0	3.064801	-1.749722	2.565360
12	6	0	4.162185	-0.564873	1.144430
13	1	0	5.131341	-0.696243	1.606613
14	6	0	-1.914140	-0.242296	-0.983649
15	1	0	-1.756106	0.211490	-1.975505
16	6	0	-2.905273	0.596510	-0.203204
17	6	0	4.061433	0.205733	-0.013409
18	6	0	-4.193967	0.102368	-0.388717
19	6	0	-2.668076	1.715036	0.577343
20	1	0	-1.658483	2.080680	0.729849
21	6	0	-2.684554	-1.571220	-1.169382
22	1	0	-2.339200	-2.108613	-2.059621
23	6	0	-4.155257	-1.121069	-1.274133

24	1	0	-4.402571	-0.859312	-2.309050
25	1	0	-4.842292	-1.909614	-0.957099
26	6	0	-5.046230	1.862811	0.991184
27	1	0	-5.880146	2.362270	1.470020
28	6	0	-3.754739	2.348272	1.177984
29	1	0	-3.592930	3.219530	1.801160
30	6	0	-5.275555	0.734054	0.205833
31	1	0	-6.282065	0.352533	0.074074
32	8	0	0.722661	-1.538606	1.600695
33	1	0	-0.080597	-1.314470	1.058673
34	7	0	5.268538	0.815630	-0.578189
35	8	0	6.314661	0.635398	0.007403
36	8	0	5.147835	1.463523	-1.597175

Structure 38/43 imine (M06-2X, DMSO)

Energy (Hartrees): =-1028.3138952
No imaginary frequencies

Standard orientation:

Center Number	Atomic Number	Atomic Type	Coordinates (Angstroms) X	Y	Z
1	7	0	-0.652178	-0.423408	-0.289380
2	8	0	-2.458793	-2.338541	0.075259
3	1	0	-2.989750	-3.139955	0.018372
4	6	0	1.698587	-0.152282	-0.121592
5	6	0	0.418905	0.024152	-0.811074
6	1	0	0.431405	0.541243	-1.776545
7	6	0	2.860722	0.376635	-0.671044
8	1	0	2.819096	0.925043	-1.604321
9	6	0	1.764425	-0.875292	1.098112
10	6	0	3.004406	-1.039397	1.736597
11	1	0	3.038327	-1.594135	2.665860
12	6	0	4.149638	-0.505855	1.186969
13	1	0	5.107977	-0.628139	1.674070
14	6	0	-1.909919	-0.270619	-0.980491
15	1	0	-1.755722	0.149615	-1.984432
16	6	0	-2.905824	0.586237	-0.225070
17	6	0	4.065966	0.197409	-0.015471
18	6	0	-4.192687	0.075060	-0.398701
19	6	0	-2.679250	1.731762	0.521266
20	1	0	-1.674253	2.119261	0.654599
21	6	0	-2.670566	-1.608518	-1.122179
22	1	0	-2.319122	-2.176284	-1.988521
23	6	0	-4.140395	-1.170297	-1.251420
24	1	0	-4.371512	-0.930310	-2.294793
25	1	0	-4.828497	-1.954127	-0.926649
26	6	0	-5.060864	1.868050	0.935152
27	1	0	-5.900061	2.373011	1.400326
28	6	0	-3.773207	2.372760	1.105542
29	1	0	-3.619487	3.266854	1.698996
30	6	0	-5.280212	0.714085	0.180247
31	1	0	-6.283816	0.320364	0.058224
32	8	0	0.687257	-1.406496	1.653170
33	1	0	-0.096027	-1.186103	1.058675
34	7	0	5.280648	0.756036	-0.599882
35	8	0	6.324489	0.612567	0.007018
36	8	0	5.190022	1.337710	-1.664208

Structure 38/43 enamine (M06-2X, Gas Phase)

Energy (Hartrees): =-1028.2799092
No imaginary frequencies

Standard orientation:

Center Number	Atomic Number	Atomic Type	Coordinates (Angstroms) X	Y	Z
1	7	0	-0.671547	-0.509677	-0.351363
2	8	0	-2.513776	-2.326398	-0.017896
3	1	0	-2.954942	-3.175305	-0.089587
4	6	0	1.696842	-0.203047	-0.109160
5	6	0	0.466192	0.010314	-0.742599
6	1	0	0.429510	0.650715	-1.623312
7	6	0	2.852699	0.414701	-0.633960
8	1	0	2.799056	1.047114	-1.513010
9	6	0	1.767619	-1.061540	1.077781
10	6	0	3.092375	-1.216560	1.658687
11	1	0	3.162670	-1.845541	2.537037
12	6	0	4.181434	-0.607289	1.130259

13	1	0	5.164876	-0.726187	1.566353
14	6	0	-1.944117	-0.267862	-0.993969
15	1	0	-1.749079	0.189055	-1.970905
16	6	0	-2.898855	0.599195	-0.198441
17	6	0	4.056672	0.215839	-0.027947
18	6	0	-4.201746	0.139972	-0.366504
19	6	0	-2.615925	1.706493	0.583408
20	1	0	-1.594118	2.041524	0.725816
21	6	0	-2.753867	-1.570063	-1.185694
22	1	0	-2.436525	-2.115583	-2.080105
23	6	0	-4.213997	-1.083871	-1.253344
24	1	0	-4.482354	-0.818232	-2.281751
25	1	0	-4.907898	-1.856549	-0.914793
26	6	0	-4.982272	1.917741	1.032907
27	1	0	-5.794473	2.437548	1.527061
28	6	0	-3.675003	2.366881	1.202568
29	1	0	-3.479082	3.229628	1.827478
30	6	0	-5.255903	0.799076	0.247747
31	1	0	-6.274237	0.445323	0.131975
32	8	0	0.765964	-1.615071	1.550907
33	1	0	-0.624192	-1.124935	0.475212
34	7	0	5.243091	0.859274	-0.576551
35	8	0	6.300400	0.663531	-0.011675
36	8	0	5.110796	1.554734	-1.565397

Structure 38/43 enamine (M06-2X, DMSO)

Energy (Hartrees): =-1028.3148058
No imaginary frequencies

Standard orientation:

Center Number	Atomic Number	Atomic Type	Coordinates (Angstroms) X	Y	Z
1	7	0	-0.669358	-0.479428	-0.358047
2	8	0	-2.466836	-2.329077	0.020829
3	1	0	-2.948107	-3.161662	-0.027881
4	6	0	1.696419	-0.194946	-0.092912
5	6	0	0.465342	0.003976	-0.767143
6	1	0	0.450904	0.595342	-1.680549
7	6	0	2.850639	0.371152	-0.650510
8	1	0	2.785279	0.937748	-1.572387
9	6	0	1.751160	-0.966564	1.146986
10	6	0	3.067414	-1.091161	1.745367
11	1	0	3.136619	-1.655638	2.667732
12	6	0	4.170881	-0.531065	1.184013
13	1	0	5.144544	-0.636710	1.645847
14	6	0	-1.944562	-0.275544	-1.018674
15	1	0	-1.753493	0.157673	-2.004483
16	6	0	-2.903419	0.595003	-0.231834
17	6	0	4.062524	0.205299	-0.026057
18	6	0	-4.199712	0.099892	-0.366706
19	6	0	-2.634664	1.735266	0.508844
20	1	0	-1.620286	2.107313	0.611423
21	6	0	-2.729067	-1.596589	-1.166349
22	1	0	-2.413880	-2.159174	-2.048177
23	6	0	-4.196238	-1.141115	-1.227658
24	1	0	-4.469566	-0.893520	-2.258577
25	1	0	-4.875341	-1.920617	-0.875971
26	6	0	-4.999316	1.900360	0.998735
27	1	0	-5.816343	2.415143	1.491754
28	6	0	-3.700144	2.388473	1.129073
29	1	0	-3.515216	3.278454	1.719507
30	6	0	-5.259226	0.752099	0.249238
31	1	0	-6.271275	0.372395	0.158337
32	8	0	0.739659	-1.481533	1.658108
33	1	0	-0.639149	-1.049557	0.496863
34	7	0	5.247347	0.790544	-0.609613
35	8	0	6.308193	0.643885	-0.024888
36	8	0	5.140384	1.405197	-1.658584

Structure 38/43 TS (M06-2X, Gas Phase)

Energy (Hartrees): =-1028.2768016
Imaginary frequency -961.18

Standard orientation:

Center Number	Atomic Number	Atomic Type	Coordinates (Angstroms) X	Y	Z

1	7	0	-0.633268	-0.449033	-0.392553
2	8	0	-2.386154	-2.307497	0.158525
3	1	0	-2.835207	-3.154973	0.184210
4	6	0	1.688251	-0.135469	-0.196964
5	6	0	0.453897	0.041397	-0.890023
6	1	0	0.425823	0.587265	-1.835927
7	6	0	2.884603	0.387867	-0.699042
8	1	0	2.908424	0.948688	-1.626153
9	6	0	1.657056	-0.883902	1.040268
10	6	0	2.905889	-1.057971	1.718926
11	6	0	4.061867	-0.536748	1.213026
12	6	0	-1.929337	-0.316431	-1.012366
13	1	0	-1.798056	0.059213	-2.036051
14	6	0	-2.886515	0.583114	-0.257484
15	6	0	4.047372	0.187776	0.000963
16	6	0	-4.176744	0.066456	-0.332639
17	6	0	-2.618066	1.766717	0.408960
18	1	0	-1.605246	2.148556	0.479156
19	6	0	-2.694775	-1.658950	-1.054896
20	1	0	-2.391887	-2.265940	-1.914603
21	6	0	-4.174056	-1.230101	-1.108797
22	1	0	-4.487860	-1.063364	-2.145204
23	1	0	-4.828243	-1.993079	-0.680618
24	6	0	-4.972387	1.936310	0.932195
25	1	0	-5.785212	2.470273	1.409960
26	6	0	-3.678003	2.444263	1.007875
27	1	0	-3.492991	3.367347	1.543471
28	6	0	-5.231780	0.742204	0.261578
29	1	0	-6.239969	0.345641	0.217069
30	8	0	0.569975	-1.359673	1.493131
31	1	0	-0.354814	-1.001623	0.604576
32	1	0	5.008195	-0.664949	1.721989
33	7	0	5.294758	0.734877	-0.521168
34	8	0	6.307450	0.539323	0.119189
35	8	0	5.249864	1.354663	-1.565894
36	1	0	2.893917	-1.617689	2.645065

Structure 38/43 TS (M06-2X, DMSO)

Energy (Hartrees): =-1028.3088996
Imaginary frequency -1056.16

Standard orientation:

Center Number	Atomic Number	Atomic Type	Coordinates (Angstroms) X	Y	Z
1	7	0	-0.628716	-0.457906	-0.356151
2	8	0	-2.381534	-2.296145	0.361602
3	1	0	-2.890843	-3.107461	0.459102
4	6	0	1.689380	-0.143488	-0.185386
5	6	0	0.453390	-0.071036	-0.929185
6	1	0	0.448621	0.305139	-1.953293
7	6	0	2.891735	0.265239	-0.752321
8	1	0	2.915139	0.649060	-1.765300
9	6	0	1.638393	-0.656888	1.156837
10	6	0	2.863651	-0.730149	1.877565
11	6	0	4.041676	-0.324336	1.306750
12	6	0	-1.921526	-0.410757	-1.000620
13	1	0	-1.791252	-0.135280	-2.053719
14	6	0	-2.883737	0.546199	-0.327030
15	6	0	4.051743	0.173691	-0.008098
16	6	0	-4.171521	0.012167	-0.353875
17	6	0	-2.623526	1.784153	0.238788
18	1	0	-1.614481	2.183003	0.264822
19	6	0	-2.675329	-1.755758	-0.915161
20	1	0	-2.365472	-2.441028	-1.708628
21	6	0	-4.154406	-1.343587	-1.019085
22	1	0	-4.449579	-1.260478	-2.070361
23	1	0	-4.812083	-2.074246	-0.542882
24	6	0	-4.979345	1.974660	0.760180
25	1	0	-5.796086	2.538689	1.196414
26	6	0	-3.688738	2.499735	0.787119
27	1	0	-3.511522	3.467065	1.243279
28	6	0	-5.230835	0.725876	0.189111
29	1	0	-6.236257	0.318687	0.180546
30	8	0	0.524597	-1.033526	1.675481
31	1	0	-0.319174	-0.841162	0.768237
32	1	0	4.971378	-0.381928	1.857969
33	7	0	5.307659	0.598748	-0.598860
34	8	0	6.319400	0.506962	0.073187
35	8	0	5.294330	1.027066	-1.739541
36	1	0	2.838627	-1.115135	2.889513

Structure 40/44 imine (M06-2X, Gas Phase)

Energy (Hartrees): =-938.3128768
No imaginary frequencies

Standard orientation:

Center Number	Atomic Number	Atomic Type	Coordinates (Angstroms) X	Y	Z
1	7	0	0.506401	-0.446829	0.365309
2	8	0	1.993569	-2.055446	-1.139653
3	1	0	2.406162	-2.819489	-1.548293
4	6	0	-1.799145	-0.091263	0.861132
5	6	0	-0.409250	-0.281931	1.237352
6	1	0	-0.182015	-0.286115	2.313179
7	6	0	-2.780113	0.130916	1.840133
8	1	0	-2.473502	0.170499	2.880795
9	6	0	-2.195685	-0.150161	-0.493206
10	6	0	-3.543849	0.018439	-0.831098
11	1	0	-3.798862	-0.035322	-1.879780
12	6	0	-4.485909	0.241903	0.163277
13	6	0	1.864172	-0.665076	0.785480
14	1	0	1.931017	-0.738682	1.884369
15	6	0	2.831492	0.397330	0.305462
16	6	0	-4.104972	0.298803	1.515359
17	6	0	4.059365	-0.182040	-0.004276
18	6	0	2.627527	1.760763	0.180473
19	1	0	1.658693	2.195352	0.401086
20	6	0	2.474594	-1.953444	0.183661
21	1	0	2.193080	-2.835511	0.769843
22	6	0	3.991352	-1.676597	0.207907
23	1	0	4.415780	-1.951342	1.180214
24	1	0	4.519429	-2.248319	-0.559593
25	6	0	4.918899	1.981399	-0.561559
26	1	0	5.731013	2.609975	-0.907326
27	6	0	3.686634	2.553997	-0.257812
28	1	0	3.547951	3.622466	-0.372725
29	6	0	5.113899	0.606318	-0.438318
30	1	0	6.071394	0.163317	-0.689838
31	8	0	-1.334843	-0.368091	-1.484306
32	1	0	-0.429713	-0.472894	-1.093046
33	1	0	-4.867985	0.471119	2.262118
34	8	0	-5.804763	0.417123	-0.074506
35	6	0	-6.244488	0.379634	-1.417636
36	1	0	-6.040853	-0.594531	-1.872492
37	1	0	-7.318146	0.548671	-1.385866
38	1	0	-5.767105	1.165967	-2.010046

Structure 40/44 imine (M06-2X, DMSO)

Energy (Hartrees): =-938.3393864
No imaginary frequencies

Standard orientation:

Center Number	Atomic Number	Atomic Type	Coordinates (Angstroms) X	Y	Z
1	7	0	0.501191	-0.472122	0.358214
2	8	0	2.021405	-2.030393	-1.204705
3	1	0	2.466338	-2.776496	-1.620219
4	6	0	-1.800350	-0.134293	0.868073
5	6	0	-0.408399	-0.334455	1.243920
6	1	0	-0.179412	-0.367151	2.315752
7	6	0	-2.784176	0.057936	1.850109
8	1	0	-2.480754	0.063109	2.892451
9	6	0	-2.193106	-0.149451	-0.488756
10	6	0	-3.536803	0.034587	-0.832944
11	1	0	-3.796163	0.018686	-1.882824
12	6	0	-4.485622	0.231336	0.165553
13	6	0	1.865494	-0.695015	0.769367
14	1	0	1.933030	-0.798568	1.862747
15	6	0	2.818687	0.392391	0.315723
16	6	0	-4.108461	0.239899	1.521170
17	6	0	4.054680	-0.168646	-0.008151
18	6	0	2.601450	1.757955	0.224708
19	1	0	1.632857	2.182881	0.468250
20	6	0	2.489320	-1.957588	0.132244
21	1	0	2.215432	-2.859702	0.687272
22	6	0	4.000836	-1.667445	0.172439
23	1	0	4.414692	-1.953366	1.145417
24	1	0	4.542049	-2.221108	-0.598419
25	6	0	4.889887	2.019888	-0.519913

26	1	0	5.695695	2.664336	-0.852975
27	6	0	3.652352	2.573749	-0.199063
28	1	0	3.502855	3.644067	-0.285150
29	6	0	5.100086	0.642410	-0.426785
30	1	0	6.062756	0.215698	-0.688561
31	8	0	-1.312244	-0.341053	-1.473365
32	1	0	-0.415031	-0.456362	-1.046113
33	1	0	-4.869506	0.390951	2.276042
34	8	0	-5.797250	0.423348	-0.075154
35	6	0	-6.230256	0.450057	-1.429694
36	1	0	-6.043707	-0.507905	-1.922895
37	1	0	-7.301448	0.637255	-1.396864
38	1	0	-5.737015	1.253424	-1.983619

Structure 40/44 enamine (M06-2X, Gas Phase)

Energy (Hartrees): =-938.3058313
No imaginary frequencies

Standard orientation:

Center Number	Atomic Number	Atomic Type	Coordinates (Angstroms) X	Y	Z
1	7	0	0.527564	-0.593662	0.453716
2	8	0	2.118233	-2.206522	-0.892320
3	1	0	2.414823	-3.073766	-1.174559
4	6	0	-1.772218	-0.000269	0.839957
5	6	0	-0.445675	-0.126263	1.219458
6	1	0	-0.152369	0.179853	2.223651
7	6	0	-2.730943	0.520290	1.764003
8	1	0	-2.394319	0.801498	2.757618
9	6	0	-2.187541	-0.392995	-0.510454
10	6	0	-3.588239	-0.209101	-0.822917
11	1	0	-3.889182	-0.497396	-1.819883
12	6	0	-4.456633	0.296180	0.101897
13	6	0	1.910802	-0.642938	0.854498
14	1	0	1.951123	-0.577569	1.948630
15	6	0	2.797260	0.430796	0.254303
16	6	0	-4.032045	0.670465	1.423619
17	6	0	4.061450	-0.089489	-0.005686
18	6	0	2.489243	1.753629	-0.014271
19	1	0	1.490727	2.137795	0.165722
20	6	0	2.614153	-1.941814	0.403495
21	1	0	2.399821	-2.772049	1.083908
22	6	0	4.105895	-1.555272	0.362420
23	1	0	4.566909	-1.696738	1.346435
24	1	0	4.655889	-2.165132	-0.357630
25	6	0	4.755043	2.058809	-0.801506
26	1	0	5.516163	2.704400	-1.223326
27	6	0	3.484850	2.569972	-0.546731
28	1	0	3.267413	3.606518	-0.775069
29	6	0	5.052768	0.723105	-0.535268
30	1	0	6.038788	0.326823	-0.751693
31	8	0	-1.385022	-0.855039	-1.338022
32	1	0	0.253309	-0.884014	-0.494051
33	1	0	-4.775543	1.063860	2.103180
34	8	0	-5.774844	0.496885	-0.111158
35	6	0	-6.290314	0.153090	-1.382069
36	1	0	-6.152441	-0.913376	-1.583704
37	1	0	-7.351746	0.387374	-1.350239
38	1	0	-5.806384	0.735802	-2.171495

Structure 40/44 enamine (M06-2X, DMSO)

Energy (Hartrees): =-938.3382226
No imaginary frequencies

Standard orientation:

Center Number	Atomic Number	Atomic Type	Coordinates (Angstroms) X	Y	Z
1	7	0	0.534213	-0.584712	0.471830
2	8	0	2.076574	-2.201074	-0.908139
3	1	0	2.493987	-2.997185	-1.252814
4	6	0	-1.772245	-0.023440	0.840627
5	6	0	-0.438439	-0.156354	1.242881
6	1	0	-0.162556	0.111625	2.261613
7	6	0	-2.732763	0.455845	1.776761
8	1	0	-2.397096	0.696320	2.781392
9	6	0	-2.174832	-0.356178	-0.521454
10	6	0	-3.571192	-0.169921	-0.833285
11	1	0	-3.876150	-0.417123	-1.841182

12	6	0	-4.456564	0.294290	0.107302
13	6	0	1.921907	-0.658062	0.874285
14	1	0	1.963363	-0.612815	1.966709
15	6	0	2.797870	0.426084	0.277317
16	6	0	-4.040017	0.617721	1.438989
17	6	0	4.051223	-0.098806	-0.032413
18	6	0	2.495615	1.760670	0.057205
19	1	0	1.511565	2.154044	0.292505
20	6	0	2.607495	-1.950731	0.384704
21	1	0	2.411582	-2.792273	1.053729
22	6	0	4.096013	-1.569693	0.309661
23	1	0	4.573166	-1.723137	1.283271
24	1	0	4.630996	-2.174053	-0.426136
25	6	0	4.739871	2.066844	-0.795270
26	1	0	5.494986	2.717483	-1.221867
27	6	0	3.482968	2.583592	-0.485365
28	1	0	3.270440	3.629742	-0.673721
29	6	0	5.033890	0.720746	-0.571528
30	1	0	6.010825	0.322525	-0.824638
31	8	0	-1.361644	-0.778735	-1.374147
32	1	0	0.270838	-0.843719	-0.485453
33	1	0	-4.780425	0.983651	2.138647
34	8	0	-5.770411	0.488711	-0.114887
35	6	0	-6.279388	0.170932	-1.403918
36	1	0	-6.130199	-0.888022	-1.632259
37	1	0	-7.344396	0.390252	-1.368474
38	1	0	-5.805039	0.783242	-2.175744

Structure 40/44 TS (M06-2X, Gas Phase)

Energy (Hartrees): =-938.3021259
Imaginary frequency -995.25

Standard orientation:

Center Number	Atomic Number	Atomic Type	Coordinates (Angstroms) X	Y	Z
1	7	0	-0.470006	0.500529	0.491939
2	8	0	-1.914335	2.074446	-1.069181
3	1	0	-2.259557	2.866169	-1.486676
4	6	0	1.791244	0.049396	0.962278
5	6	0	0.449706	0.220491	1.368929
6	1	0	0.175947	0.118756	2.422387
7	6	0	2.822116	-0.262723	1.878441
8	1	0	2.571340	-0.379541	2.928568
9	6	0	2.088641	0.213019	-0.439977
10	6	0	3.444273	0.047325	-0.844567
11	6	0	4.414810	-0.258859	0.082255
12	6	0	-1.856573	0.679063	0.831124
13	1	0	-1.952717	0.727738	1.925241
14	6	0	-2.780737	-0.397115	0.297771
15	6	0	4.111256	-0.418492	1.464210
16	6	0	-4.003872	0.163832	-0.057872
17	6	0	-2.542590	-1.754326	0.166492
18	1	0	-1.574782	-2.172532	0.421308
19	6	0	-2.464447	1.964010	0.224531
20	1	0	-2.225307	2.844549	0.830602
21	6	0	-3.974711	1.657695	0.170335
22	1	0	-4.453672	1.916027	1.121480
23	1	0	-4.472230	2.225490	-0.619670
24	6	0	-4.795602	-2.009531	-0.673679
25	1	0	-5.579303	-2.649726	-1.060911
26	6	0	-3.566127	-2.562397	-0.324763
27	1	0	-3.401446	-3.626250	-0.446391
28	6	0	-5.023164	-0.640130	-0.544944
29	1	0	-5.977169	-0.213197	-0.834387
30	8	0	1.169396	0.495769	-1.279656
31	1	0	0.060724	0.575459	-0.552709
32	1	0	3.653698	0.172824	-1.897086
33	1	0	4.919644	-0.658257	2.140829
34	8	0	5.720163	-0.434072	-0.218744
35	6	0	6.107773	-0.287736	-1.570566
36	1	0	5.889290	0.720736	-1.934164
37	1	0	7.180832	-0.461825	-1.595690
38	1	0	5.600987	-1.020396	-2.205651

Structure 40/44 TS (M06-2X, DMSO)

Energy (Hartrees): =-938.3326494
Imaginary frequency -1099.91

Standard orientation:

Center Number	Atomic Number	Atomic Type	Coordinates (Angstroms) X	Y	Z
1	7	0	0.474003	-0.597427	0.428503
2	8	0	1.947639	-1.931463	-1.348452
3	1	0	2.384615	-2.611462	-1.871817
4	6	0	-1.794516	-0.269197	0.944551
5	6	0	-0.442854	-0.514094	1.336691
6	1	0	-0.186550	-0.625310	2.391952
7	6	0	-2.834808	-0.162090	1.888374
8	1	0	-2.596351	-0.276295	2.941449
9	6	0	-2.073241	-0.122532	-0.454642
10	6	0	-3.414309	0.129198	-0.841162
11	6	0	-4.409558	0.228235	0.115625
12	6	0	1.866725	-0.808662	0.741800
13	1	0	1.967018	-1.010862	1.815160
14	6	0	2.757918	0.356566	0.359852
15	6	0	-4.124942	0.082061	1.497461
16	6	0	3.990814	-0.121024	-0.084387
17	6	0	2.489321	1.714425	0.425737
18	1	0	1.521522	2.073512	0.760958
19	6	0	2.497906	-1.978614	-0.043098
20	1	0	2.288371	-2.940586	0.432301
21	6	0	3.997398	-1.631279	-0.058898
22	1	0	4.478939	-1.997604	0.853825
23	1	0	4.508792	-2.082044	-0.912404
24	6	0	4.725087	2.139307	-0.399426
25	1	0	5.490132	2.845659	-0.701183
26	6	0	3.488638	2.609271	0.039540
27	1	0	3.300391	3.676149	0.075513
28	6	0	4.985287	0.768985	-0.465619
29	1	0	5.945876	0.409061	-0.818732
30	8	0	-1.127320	-0.216940	-1.330172
31	1	0	-0.083842	-0.446608	-0.634926
32	1	0	-3.616589	0.238424	-1.897950
33	1	0	-4.936916	0.167845	2.207849
34	8	0	-5.704637	0.467624	-0.170633
35	6	0	-6.066881	0.617888	-1.537172
36	1	0	-5.856289	-0.294205	-2.102238
37	1	0	-7.137644	0.809838	-1.543365
38	1	0	-5.542847	1.462053	-1.993223

Structure 41 imine (M06-2X, Gas Phase)

Energy (Hartrees): =-1232.7559017
No imaginary frequencies

Standard orientation:

Center Number	Atomic Number	Atomic Type	Coordinates (Angstroms) X	Y	Z
1	7	0	1.119293	-0.176001	-0.654483
2	8	0	2.734203	1.713749	-1.472667
3	1	0	3.212674	2.381351	-1.969469
4	6	0	-1.213090	-0.555364	-0.416557
5	6	0	0.145725	-0.984258	-0.755167
6	1	0	0.273859	-2.019420	-1.096474
7	6	0	-2.248794	-1.476941	-0.447648
8	1	0	-2.065431	-2.507627	-0.726873
9	6	0	-1.458655	0.803754	-0.060510
10	6	0	-2.790674	1.144098	0.259691
11	6	0	-3.813890	0.219423	0.259850
12	6	0	2.457909	-0.589930	-0.990451
13	1	0	2.449656	-1.568044	-1.496812
14	6	0	3.380112	-0.648041	0.210850
15	6	0	-3.531548	-1.085464	-0.107465
16	6	0	4.649875	-0.201834	-0.145338
17	6	0	3.101828	-1.084694	1.494785
18	1	0	2.104042	-1.411179	1.767148
19	6	0	3.167795	0.440849	-1.900724
20	1	0	2.919745	0.277665	-2.955261
21	6	0	4.662605	0.219640	-1.595458
22	1	0	5.068970	-0.576903	-2.228450
23	1	0	5.248502	1.123104	-1.781491
24	6	0	5.402718	-0.635429	2.085154
25	1	0	6.189988	-0.626542	2.829457
26	6	0	4.128883	-1.074054	2.436462
27	1	0	3.933845	-1.400856	3.450628
28	6	0	5.672851	-0.195246	0.790472
29	1	0	6.663988	0.156404	0.526494
30	8	0	-0.502465	1.692916	0.000687
31	1	0	0.366263	1.242176	-0.229774

32	1	0	-4.814510	0.515415	0.545345
33	7	0	-3.148810	2.527568	0.617405
34	8	0	-4.053007	2.664156	1.414309
35	8	0	-2.545546	3.420185	0.075944
36	7	0	-4.615061	-2.075155	-0.133143
37	8	0	-5.724212	-1.695920	0.166932
38	8	0	-4.324154	-3.207522	-0.454031

Structure 41 imine (M06-2X, DMSO)

Energy (Hartrees): =-1232.7878711
No imaginary frequencies

Standard orientation:

Center Number	Atomic Number	Atomic Type	Coordinates (Angstroms) X	Y	Z
1	7	0	1.105573	-0.219969	-0.676384
2	8	0	2.756668	1.645154	-1.557862
3	1	0	3.267554	2.270797	-2.082337
4	6	0	-1.218394	-0.584989	-0.422805
5	6	0	0.136242	-1.039943	-0.748979
6	1	0	0.264526	-2.085113	-1.045694
7	6	0	-2.264864	-1.489575	-0.427060
8	1	0	-2.081281	-2.526025	-0.684209
9	6	0	-1.436653	0.787549	-0.098594
10	6	0	-2.760294	1.158680	0.227009
11	6	0	-3.801476	0.251916	0.248488
12	6	0	2.451124	-0.644225	-0.982902
13	1	0	2.446811	-1.641098	-1.444019
14	6	0	3.355139	-0.648616	0.234194
15	6	0	-3.541937	-1.063736	-0.090430
16	6	0	4.633136	-0.222600	-0.127886
17	6	0	3.058460	-1.021933	1.535215
18	1	0	2.057656	-1.340937	1.808464
19	6	0	3.175609	0.341489	-1.926446
20	1	0	2.938181	0.139489	-2.974677
21	6	0	4.663272	0.121528	-1.597677
22	1	0	5.057239	-0.718029	-2.180274
23	1	0	5.264535	1.003223	-1.831138
24	6	0	5.353996	-0.544436	2.135884
25	1	0	6.131816	-0.498754	2.889946
26	6	0	4.074028	-0.965190	2.491142
27	1	0	3.864182	-1.243200	3.517745
28	6	0	5.643404	-0.169864	0.822286
29	1	0	6.639764	0.164994	0.553738
30	8	0	-0.451160	1.643691	-0.073859
31	1	0	0.405183	1.118549	-0.307885
32	1	0	-4.796639	0.573851	0.524299
33	7	0	-3.086327	2.543963	0.576063
34	8	0	-4.008060	2.719096	1.349497
35	8	0	-2.440422	3.430203	0.065391
36	7	0	-4.641925	-2.026265	-0.085971
37	8	0	-5.749089	-1.622605	0.206106
38	8	0	-4.386128	-3.177813	-0.372844

Structure 41 enamine (M06-2X, Gas Phase)

Energy (Hartrees): =-1232.753737
No imaginary frequencies

Standard orientation:

Center Number	Atomic Number	Atomic Type	Coordinates (Angstroms) X	Y	Z
1	7	0	1.151428	-0.057426	-0.697281
2	8	0	2.783705	1.865810	-1.282503
3	1	0	3.111287	2.603332	-1.801305
4	6	0	-1.199063	-0.486732	-0.412560
5	6	0	0.118516	-0.852947	-0.748322
6	1	0	0.307900	-1.873849	-1.078164
7	6	0	-2.204951	-1.462916	-0.490740
8	1	0	-1.978263	-2.474550	-0.808502
9	6	0	-1.476870	0.891767	0.013985
10	6	0	-2.890057	1.129271	0.307630
11	6	0	-3.840394	0.160832	0.256745
12	6	0	2.505427	-0.450238	-1.032503
13	1	0	2.455768	-1.374036	-1.619409
14	6	0	3.417810	-0.634386	0.162720
15	6	0	-3.492137	-1.144180	-0.154558
16	6	0	4.691468	-0.165083	-0.144023

17	6	0	3.126429	-1.191860	1.396333
18	1	0	2.124397	-1.533458	1.633246
19	6	0	3.228947	0.649517	-1.843939
20	1	0	2.983596	0.593059	-2.908912
21	6	0	4.719112	0.401134	-1.544785
22	1	0	5.137530	-0.323696	-2.251409
23	1	0	5.297761	1.323576	-1.626464
24	6	0	5.427596	-0.820723	2.036644
25	1	0	6.210224	-0.892467	2.782384
26	6	0	4.148386	-1.281171	2.338648
27	1	0	3.946058	-1.703482	3.315262
28	6	0	5.709108	-0.257387	0.793641
29	1	0	6.703681	0.112472	0.571344
30	8	0	-0.587066	1.730719	0.132648
31	1	0	0.973244	0.910660	-0.389786
32	1	0	-4.863995	0.388384	0.526979
33	7	0	-3.329633	2.478494	0.684355
34	8	0	-4.255291	2.562008	1.466950
35	8	0	-2.762509	3.410064	0.167849
36	7	0	-4.528529	-2.167517	-0.229576
37	8	0	-5.658228	-1.842516	0.066335
38	8	0	-4.195361	-3.281108	-0.583434

Structure 41 enamine (M06-2X, DMSO)

Energy (Hartrees): =-1232.7949415
No imaginary frequencies

Standard orientation:

Center Number	Atomic Number	Atomic Type	Coordinates (Angstroms) X	Y	Z
1	7	0	1.157750	-0.214071	-0.709772
2	8	0	2.794793	1.560163	-1.662978
3	1	0	3.241881	2.174797	-2.254265
4	6	0	-1.206867	-0.526966	-0.391082
5	6	0	0.113085	-0.977312	-0.687832
6	1	0	0.265881	-2.032262	-0.906645
7	6	0	-2.220829	-1.479265	-0.391368
8	1	0	-1.992336	-2.511349	-0.632541
9	6	0	-1.451341	0.882147	-0.073785
10	6	0	-2.853091	1.166881	0.216819
11	6	0	-3.831726	0.215412	0.239418
12	6	0	2.511337	-0.674913	-0.966690
13	1	0	2.453961	-1.691455	-1.363726
14	6	0	3.398924	-0.620161	0.260133
15	6	0	-3.514818	-1.112203	-0.076885
16	6	0	4.676465	-0.201868	-0.108180
17	6	0	3.086210	-0.934822	1.573267
18	1	0	2.084356	-1.248482	1.848988
19	6	0	3.246874	0.248218	-1.961529
20	1	0	3.024850	-0.013673	-2.998479
21	6	0	4.728455	0.070350	-1.592257
22	1	0	5.149084	-0.788154	-2.125796
23	1	0	5.315782	0.952378	-1.855936
24	6	0	5.370383	-0.410057	2.176578
25	1	0	6.137897	-0.321919	2.937317
26	6	0	4.089137	-0.824591	2.536537
27	1	0	3.869134	-1.056021	3.572390
28	6	0	5.674291	-0.095945	0.851256
29	1	0	6.671076	0.234061	0.579006
30	8	0	-0.542849	1.716255	-0.026446
31	1	0	1.011837	0.783235	-0.513969
32	1	0	-4.848374	0.488442	0.491733
33	7	0	-3.262250	2.534842	0.517785
34	8	0	-4.189430	2.692563	1.293814
35	8	0	-2.675861	3.439935	-0.033556
36	7	0	-4.564602	-2.109392	-0.072598
37	8	0	-5.694131	-1.751065	0.208692
38	8	0	-4.267552	-3.257687	-0.348573

Structure 41 TS (M06-2X, Gas Phase)

Energy (Hartrees): =-1232.7494228
Imaginary frequency -957.56

Standard orientation:

Center Number	Atomic Number	Atomic Type	Coordinates (Angstroms) X	Y	Z

1	7	0	1.081011	-0.243446	-0.711569
2	8	0	2.629947	1.727938	-1.380609
3	1	0	2.991596	2.454947	-1.891793
4	6	0	-1.224195	-0.621561	-0.437415
5	6	0	0.096450	-1.067127	-0.786687
6	1	0	0.255417	-2.097464	-1.113496
7	6	0	-2.301417	-1.499893	-0.466552
8	1	0	-2.173563	-2.535324	-0.760530
9	6	0	-1.371754	0.769201	-0.055957
10	6	0	-2.712169	1.157510	0.278759
11	6	0	-3.762623	0.277870	0.277027
12	6	0	2.445645	-0.590073	-1.034155
13	1	0	2.454281	-1.540183	-1.584631
14	6	0	3.354189	-0.682866	0.175190
15	6	0	-3.553301	-1.048551	-0.106707
16	6	0	4.609987	-0.173076	-0.141607
17	6	0	3.076504	-1.199702	1.429255
18	1	0	2.088045	-1.574577	1.672454
19	6	0	3.127689	0.508575	-1.882289
20	1	0	2.896918	0.394800	-2.946654
21	6	0	4.625374	0.332108	-1.565029
22	1	0	5.076412	-0.406505	-2.236610
23	1	0	5.169789	1.271244	-1.685205
24	6	0	5.353546	-0.703894	2.070677
25	1	0	6.131928	-0.709068	2.824240
26	6	0	4.092545	-1.205390	2.382331
27	1	0	3.898949	-1.594086	3.374583
28	6	0	5.621837	-0.182734	0.806339
29	1	0	6.602156	0.218966	0.575762
30	8	0	-0.370589	1.538706	-0.005507
31	1	0	0.637771	0.812294	-0.356947
32	1	0	-4.748024	0.613287	0.573548
33	7	0	-3.007758	2.548444	0.648540
34	8	0	-3.923585	2.727889	1.425865
35	8	0	-2.342731	3.415226	0.136406
36	7	0	-4.683135	-1.975540	-0.127674
37	8	0	-5.769688	-1.542721	0.187384
38	8	0	-4.458647	-3.121650	-0.459649

Structure 46 (M06-2X, Gas Phase)

Energy (Hartrees): =-420.7434085
No imaginary frequencies

Standard orientation:

Center Number	Atomic Number	Atomic Type	Coordinates (Angstroms) X	Y	Z
1	6	0	0.000000	0.636258	0.000000
2	6	0	1.382541	0.863994	0.000000
3	6	0	2.275263	-0.188232	0.000000
4	6	0	1.775946	-1.495720	0.000000
5	6	0	0.416280	-1.748242	0.000000
6	6	0	-0.491992	-0.685289	0.000000
7	6	0	-0.921298	1.770220	0.000000
8	1	0	1.739008	1.889504	0.000000
9	1	0	3.342090	-0.008850	0.000000
10	1	0	2.465819	-2.331682	0.000000
11	1	0	0.023394	-2.756952	0.000000
12	1	0	-0.448854	2.769658	0.000000
13	1	0	-2.292445	-0.126169	0.000000
14	8	0	-2.133187	1.672229	0.000000
15	8	0	-1.797995	-0.966409	0.000000

Structure 46 (M06-2X, EtOH)

Energy (Hartrees): =-420.7546519
No imaginary frequencies

Standard orientation:

Center Number	Atomic Number	Atomic Type	Coordinates (Angstroms) X	Y	Z
1	6	0	0.000000	0.634364	0.000000
2	6	0	1.384110	0.857576	0.000000
3	6	0	2.272823	-0.200262	0.000000
4	6	0	1.770169	-1.505949	0.000000
5	6	0	0.406846	-1.753139	0.000000
6	6	0	-0.493543	-0.685947	0.000000
7	6	0	-0.903978	1.780019	0.000000
8	1	0	1.741406	1.882681	0.000000
9	1	0	3.340954	-0.024165	0.000000

10	1	0	2.456776	-2.345215	0.000000
11	1	0	0.015417	-2.763639	0.000000
12	1	0	-0.421215	2.770762	0.000000
13	1	0	-2.302537	-0.121220	0.000000
14	8	0	-2.122308	1.693169	0.000000
15	8	0	-1.808862	-0.963065	0.000000

Structure 47a (M06-2X, Gas Phase)

Energy (Hartrees): =-1168.1345903
No imaginary frequencies

Standard orientation:

Center	Atomic	Atomic	Coordinates (Angstroms)		
Number	Number	Type	X	Y	Z
1	6	0	-4.018555	-1.933982	1.060077
2	6	0	-2.887173	-2.741675	1.130100
3	1	0	-0.027435	0.267531	1.298133
4	8	0	-0.669224	0.851140	1.735769
5	6	0	-4.046733	-0.824384	0.218821
6	6	0	2.777402	0.979296	0.139280
7	6	0	4.977813	0.917054	1.096444
8	6	0	-2.937375	-0.496550	-0.554924
9	6	0	-1.800220	-1.288781	-0.463108
10	6	0	5.131624	-0.404614	0.684167
11	6	0	-1.779961	-2.416568	0.358675
12	6	0	4.100853	-1.030815	-0.004133
13	6	0	-0.465613	-3.149067	0.255706
14	1	0	-0.589349	-4.231101	0.188148
15	1	0	0.175237	-2.933576	1.115331
16	1	0	-0.608143	-0.879343	-2.253297
17	7	0	0.539540	-0.307185	-0.554857
18	6	0	-0.487441	-1.150799	-1.194197
19	6	0	0.185644	-2.544660	-1.000143
20	8	0	1.579510	-2.304436	-0.808627
21	1	0	0.039487	-3.179772	-1.877041
22	6	0	3.806042	1.611367	0.831634
23	6	0	1.774212	-0.935948	-1.021537
24	6	0	2.926673	-0.342314	-0.281566
25	1	0	-2.875164	-3.616881	1.770191
26	1	0	-4.890265	-2.178227	1.656002
27	1	0	-4.936774	-0.208718	0.166925
28	6	0	-1.211232	1.672477	0.816999
29	1	0	-2.961345	0.372240	-1.204236
30	6	0	-2.485426	3.334334	-1.046603
31	6	0	-2.992764	3.193769	0.243303
32	1	0	-3.892592	3.725410	0.530417
33	8	0	1.637120	1.700724	-0.077487
34	6	0	-2.363512	2.374605	1.168429
35	1	0	0.809418	1.285494	-1.918785
36	6	0	0.584000	1.115123	-0.851324
37	6	0	-0.683035	1.823474	-0.475061
38	1	0	1.885179	-0.755548	-2.110423
39	6	0	-1.324564	2.652996	-1.388665
40	1	0	-2.977191	3.975637	-1.766443
41	1	0	-0.900716	2.764202	-2.381674
42	1	0	5.776809	1.412714	1.634938
43	1	0	3.660503	2.634019	1.155377
44	1	0	6.045132	-0.942281	0.903627
45	1	0	4.185254	-2.063371	-0.321992
46	1	0	-2.758026	2.236424	2.167313

Structure 47a (M06-2X, Ethanol)

Energy (Hartrees): =-1168.1660334
No imaginary frequencies

Standard orientation:

Center	Atomic	Atomic	Coordinates (Angstroms)		
Number	Number	Type	X	Y	Z
1	6	0	-4.041502	-1.905526	1.054362
2	6	0	-2.916815	-2.723976	1.133621
3	1	0	-0.045972	0.223314	1.254524
4	8	0	-0.652511	0.833097	1.717421
5	6	0	-4.058807	-0.803666	0.200054
6	6	0	2.778761	0.967704	0.138504
7	6	0	4.985147	0.912208	1.087110
8	6	0	-2.942406	-0.492976	-0.573696
9	6	0	-1.809690	-1.292187	-0.469861
10	6	0	5.138148	-0.410514	0.676038

11	6	0	-1.803411	-2.414993	0.361393
12	6	0	4.103465	-1.040293	-0.006279
13	6	0	-0.501695	-3.169270	0.260092
14	1	0	-0.649819	-4.246902	0.175614
15	1	0	0.128896	-2.981959	1.134471
16	1	0	-0.601070	-0.904754	-2.256123
17	7	0	0.531994	-0.318635	-0.550690
18	6	0	-0.492666	-1.168517	-1.196626
19	6	0	0.169504	-2.563744	-0.983814
20	8	0	1.565923	-2.321259	-0.762094
21	1	0	0.044524	-3.197521	-1.863257
22	6	0	3.810147	1.604635	0.823679
23	6	0	1.767861	-0.954776	-1.010655
24	6	0	2.923345	-0.356779	-0.277111
25	1	0	-2.915444	-3.596341	1.778675
26	1	0	-4.918068	-2.137780	1.649127
27	1	0	-4.947433	-0.185769	0.135201
28	6	0	-1.198683	1.678665	0.807771
29	1	0	-2.960546	0.362063	-1.241480
30	6	0	-2.448138	3.379124	-1.028095
31	6	0	-2.948106	3.244064	0.265081
32	1	0	-3.833053	3.794742	0.564056
33	8	0	1.636784	1.688827	-0.081626
34	6	0	-2.329260	2.401666	1.180443
35	1	0	0.815275	1.265100	-1.930451
36	6	0	0.581587	1.099527	-0.868348
37	6	0	-0.675167	1.824634	-0.485690
38	1	0	1.878828	-0.797350	-2.098830
39	6	0	-1.306513	2.672213	-1.389926
40	1	0	-2.934091	4.034432	-1.740451
41	1	0	-0.891756	2.773950	-2.387729
42	1	0	5.786794	1.411761	1.619112
43	1	0	3.672280	2.631126	1.142769
44	1	0	6.055161	-0.946627	0.888253
45	1	0	4.200862	-2.070913	-0.329186
46	1	0	-2.721545	2.271240	2.182640

Structure 47b (M06-2X, Gas Phase)

Energy (Hartrees): =-1168.1452283
No imaginary frequencies

Standard orientation:

Center Number	Atomic Number	Atomic Type	Coordinates (Angstroms) X	Y	Z
1	6	0	-1.106012	2.080311	0.367177
2	6	0	-1.235144	0.586915	0.469208
3	6	0	-1.797175	-3.417096	-0.162522
4	6	0	-1.519079	2.889732	1.421615
5	6	0	-0.147408	-1.541380	0.165377
6	6	0	2.441529	-0.088965	-0.040244
7	6	0	-1.466547	4.273620	1.330316
8	6	0	1.180752	0.221424	0.733170
9	6	0	-1.526601	-2.051201	-0.118899
10	6	0	-0.625614	2.675860	-0.810283
11	6	0	4.814780	-0.943918	-1.166718
12	6	0	4.022946	-0.007338	-1.830981
13	6	0	-0.995312	4.854647	0.156282
14	6	0	-2.548674	-1.147945	-0.391157
15	6	0	-0.575310	4.066157	-0.903997
16	6	0	-4.071780	-2.956786	-0.785471
17	6	0	3.227761	-1.022699	0.626731
18	6	0	1.193990	-0.818477	1.902010
19	6	0	2.825782	0.427786	-1.272104
20	6	0	4.423161	-1.457075	0.065524
21	6	0	2.610652	-1.416679	1.946659
22	6	0	-3.064438	-3.874824	-0.487351
23	6	0	-3.821414	-1.595053	-0.737682
24	1	0	-1.450307	0.282981	1.505705
25	1	0	-4.587445	-0.861039	-0.953517
26	1	0	-3.268407	-4.937467	-0.518186
27	1	0	-5.061986	-3.306884	-1.051184
28	1	0	-0.997167	-4.115831	0.057040
29	1	0	0.584888	-2.029365	-0.488085
30	1	0	1.147596	1.259669	1.067203
31	1	0	0.888594	-0.397971	2.860999
32	1	0	-1.894631	2.417734	2.323734
33	1	0	-0.949737	5.933634	0.065957
34	1	0	-1.791364	4.889773	2.158668
35	1	0	5.039027	-2.188557	0.577009
36	1	0	5.742641	-1.276879	-1.616969
37	1	0	2.201714	1.152280	-1.784775
38	1	0	4.342192	0.380894	-2.790747
39	1	0	-0.200740	4.500675	-1.822495

40	1	0	2.558562	-2.497089	2.094737
41	1	0	3.181297	-0.993089	2.778747
42	1	0	-0.154895	1.019024	-1.612857
43	7	0	-0.032933	-0.099956	-0.036231
44	8	0	0.208161	-1.776167	1.528716
45	8	0	-0.192015	1.958252	-1.870167
46	8	0	-2.334192	0.198554	-0.340715

Structure 47b (M06-2X, Ethanol)

Energy (Hartrees): =-1168.1755135
No imaginary frequencies

Standard orientation:

Center Number	Atomic Number	Atomic Type	Coordinates (Angstroms) X	Y	Z
1	6	0	-1.214269	2.022442	0.372704
2	6	0	-1.247742	0.523776	0.481341
3	6	0	-1.600382	-3.506132	-0.185981
4	6	0	-1.636307	2.808689	1.441699
5	6	0	-0.055957	-1.541715	0.139278
6	6	0	2.456877	0.030961	-0.053672
7	6	0	-1.679720	4.194056	1.339941
8	6	0	1.180353	0.284860	0.715674
9	6	0	-1.408922	-2.126222	-0.130029
10	6	0	-0.818555	2.642825	-0.822288
11	6	0	4.887025	-0.701850	-1.152417
12	6	0	4.044072	0.172831	-1.839905
13	6	0	-1.298007	4.798662	0.144925
14	6	0	-2.489159	-1.282217	-0.367420
15	6	0	-0.865829	4.031563	-0.928984
16	6	0	-3.916334	-3.171730	-0.744203
17	6	0	3.294437	-0.842538	0.635422
18	6	0	1.244637	-0.741285	1.889938
19	6	0	2.818529	0.545272	-1.293694
20	6	0	4.518350	-1.214629	0.088801
21	6	0	2.693311	-1.245824	1.959235
22	6	0	-2.848839	-4.032787	-0.484470
23	6	0	-3.743498	-1.796685	-0.686632
24	1	0	-1.429583	0.214978	1.520181
25	1	0	-4.559989	-1.109891	-0.876519
26	1	0	-2.991993	-5.105664	-0.524708
27	1	0	-4.892210	-3.575685	-0.988019
28	1	0	-0.755480	-4.161736	-0.001253
29	1	0	0.691670	-1.990713	-0.522235
30	1	0	1.104043	1.320131	1.050108
31	1	0	0.895267	-0.335520	2.838785
32	1	0	-1.936357	2.316941	2.361555
33	1	0	-1.327764	5.878109	0.047881
34	1	0	-2.008303	4.793686	2.179898
35	1	0	5.173960	-1.898184	0.618146
36	1	0	5.836531	-0.987231	-1.591520
37	1	0	2.156555	1.219594	-1.827235
38	1	0	4.345213	0.560691	-2.806404
39	1	0	-0.555068	4.489765	-1.861039
40	1	0	2.723156	-2.322806	2.136721
41	1	0	3.223070	-0.759039	2.783462
42	1	0	-0.199429	1.020207	-1.616309
43	7	0	-0.020418	-0.094271	-0.052309
44	8	0	0.323809	-1.767925	1.501206
45	8	0	-0.382119	1.938482	-1.898593
46	8	0	-2.346038	0.076733	-0.311476

Structure 47c (M06-2X, Gas Phase)

Energy (Hartrees): =-1168.1206796
No imaginary frequencies

Standard orientation:

Center Number	Atomic Number	Atomic Type	Coordinates (Angstroms) X	Y	Z
1	1	0	2.719329	-2.846980	1.695375
2	6	0	-1.991555	-1.258192	-1.023078
3	1	0	-4.270127	-3.621756	-1.833474
4	1	0	-3.024527	-1.712335	-2.833687
5	1	0	1.210232	2.390403	-0.989266
6	6	0	2.179954	-2.155178	1.046571
7	1	0	-4.181048	2.941678	0.654339
8	1	0	0.346980	0.825985	-1.611465
9	1	0	5.944258	-0.907616	-1.565096

10	6	0	3.099150	-1.291173	0.220624
11	6	0	-3.574591	-3.053939	-1.227063
12	1	0	-0.394136	4.982652	0.625299
13	1	0	-3.900826	-4.234829	0.543662
14	6	0	2.634915	0.027796	0.174105
15	6	0	-0.794105	-0.771692	1.084800
16	1	0	5.169250	1.434331	-1.565371
17	6	0	-3.370770	-3.397904	0.107136
18	1	0	1.504273	-2.732187	0.409763
19	6	0	-3.118515	2.955178	0.447904
20	1	0	1.772101	-0.915182	2.813254
21	6	0	-0.440572	0.572785	-0.893014
22	7	0	0.161179	-0.249939	0.125482
23	6	0	5.013980	-0.649244	-1.073248
24	6	0	-2.474270	-2.663517	0.871978
25	1	0	-3.097458	0.963733	-0.343616
26	6	0	-1.136500	1.827811	-0.377203
27	6	0	3.382569	1.019935	-0.445625
28	6	0	4.574234	0.669728	-1.080772
29	6	0	1.328343	0.131932	0.921455
30	6	0	-2.506026	1.840661	-0.107647
31	6	0	-2.888419	-1.990853	-1.796298
32	1	0	-2.827431	4.963736	1.163116
33	6	0	-2.360252	4.087680	0.729743
34	6	0	1.341865	-1.123027	1.831805
35	6	0	-1.786654	-1.590766	0.317559
36	1	0	3.088028	2.061700	-0.388862
37	1	0	1.190536	1.074862	1.468136
38	6	0	-1.005178	4.108063	0.440109
39	1	0	-1.297855	0.032955	1.650056
40	8	0	-1.356291	-0.218782	-1.643634
41	6	0	4.285723	-1.635186	-0.408140
42	8	0	-0.013004	-1.518378	1.996726
43	8	0	0.941148	3.080525	-0.376872
44	6	0	-0.398069	2.985343	-0.114830
45	1	0	-2.280119	-2.921578	1.906675
46	1	0	4.653927	-2.654370	-0.368179

Structure 47c (M06-2X, Ethanol)

Energy (Hartrees): =-1168.1536743
No imaginary frequencies

Standard orientation:

Center Number	Atomic Number	Atomic Type	Coordinates (Angstroms) X	Y	Z
1	1	0	2.908111	-2.652320	1.959345
2	6	0	-2.025196	-1.244653	-1.057932
3	1	0	-4.367896	-3.551387	-1.857164
4	1	0	-3.111262	-1.652197	-2.854283
5	1	0	1.041681	2.691452	-1.337869
6	6	0	2.335801	-2.101379	1.211392
7	1	0	-4.146913	2.784805	0.947167
8	1	0	0.319090	0.862042	-1.639605
9	1	0	6.039511	-0.857309	-1.483025
10	6	0	3.207931	-1.249186	0.325610
11	6	0	-3.647418	-3.008012	-1.256625
12	1	0	-0.472372	4.995173	0.620212
13	1	0	-3.966254	-4.204555	0.506940
14	6	0	2.650965	0.020416	0.138633
15	6	0	-0.769766	-0.815285	1.027698
16	1	0	5.085598	1.404407	-1.757345
17	6	0	-3.424784	-3.374675	0.069275
18	1	0	1.765736	-2.823520	0.619685
19	6	0	-3.105945	2.848754	0.654141
20	1	0	1.673447	-0.787892	2.837852
21	6	0	-0.448715	0.578370	-0.917466
22	7	0	0.174107	-0.290768	0.055919
23	6	0	5.086980	-0.615923	-1.024625
24	6	0	-2.497418	-2.669188	0.826974
25	1	0	-3.071457	0.868479	-0.145219
26	6	0	-1.147966	1.809573	-0.345434
27	6	0	3.325975	0.987028	-0.596237
28	6	0	4.547467	0.658486	-1.183720
29	6	0	1.329101	0.120881	0.860586
30	6	0	-2.491781	1.765893	0.038428
31	6	0	-2.951895	-1.948211	-1.823908
32	1	0	-2.842992	4.862388	1.371548
33	6	0	-2.377031	4.011031	0.888914
34	6	0	1.360020	-1.087411	1.836655
35	6	0	-1.794798	-1.604543	0.273034
36	1	0	2.932633	1.991161	-0.689866
37	1	0	1.166880	1.080661	1.367607
38	6	0	-1.054280	4.091469	0.480241
39	1	0	-1.241401	-0.015618	1.624034

40	8	0	-1.385006	-0.201961	-1.672942
41	6	0	4.424141	-1.575242	-0.259833
42	8	0	0.023842	-1.599203	1.895823
43	8	0	0.861498	3.167647	-0.518532
44	6	0	-0.446684	3.003465	-0.142611
45	1	0	-2.302686	-2.944474	1.857900
46	1	0	4.860900	-2.557742	-0.113278

Structure 47d (M06-2X, Gas Phase)

Energy (Hartrees): =-1168.1265717
No imaginary frequencies

Standard orientation:

Center Number	Atomic Number	Atomic Type	Coordinates (Angstroms) X	Y	Z
1	6	0	4.659904	-0.957663	-1.318592
2	6	0	3.377011	-0.446829	-1.485948
3	6	0	-2.659857	-3.711613	0.890370
4	6	0	5.250599	-0.977122	-0.055401
5	6	0	-1.179452	-1.955359	0.142779
6	6	0	2.704977	0.048327	-0.376505
7	6	0	-2.099376	-1.613144	-0.841590
8	6	0	-3.561234	-3.371779	-0.117797
9	6	0	3.284866	0.020318	0.887139
10	1	0	-1.311136	4.845233	-0.067695
11	6	0	4.566583	-0.492321	1.054805
12	8	0	-1.886454	-0.578812	-1.703333
13	6	0	1.310785	0.632968	-0.332200
14	1	0	1.258612	1.600173	-0.836802
15	6	0	-1.472439	-3.004601	1.009710
16	7	0	0.309743	-0.290565	-0.871971
17	6	0	2.352165	0.595639	1.926378
18	1	0	2.697895	1.581996	2.250981
19	1	0	2.256588	-0.033594	2.813155
20	6	0	1.002066	0.711835	1.201613
21	8	0	0.166441	-0.418035	1.440376
22	1	0	0.440618	1.603126	1.487373
23	1	0	6.248319	-1.383478	0.062888
24	6	0	-3.287657	-2.325311	-0.984418
25	6	0	-1.152228	2.818533	-0.696192
26	8	0	-0.339134	3.232288	-1.721555
27	6	0	-1.599225	3.825731	0.156272
28	6	0	-2.395387	3.510961	1.244031
29	1	0	-2.582055	0.162730	0.834421
30	1	0	-0.627456	0.806680	-2.319302
31	6	0	-0.907704	0.379470	-1.353822
32	1	0	0.951612	-1.899596	0.243254
33	6	0	-2.762194	2.187843	1.476003
34	6	0	0.108132	-1.199016	0.239000
35	1	0	2.897414	-0.448740	-2.458383
36	1	0	5.023882	-0.523819	2.037697
37	1	0	5.201194	-1.349691	-2.171364
38	1	0	-0.400798	2.628964	-2.465302
39	6	0	-2.310752	1.189432	0.627523
40	6	0	-1.483073	1.477906	-0.462242
41	1	0	-0.753404	-3.256826	1.781643
42	1	0	-2.881222	-4.524837	1.569637
43	1	0	-4.488250	-3.922319	-0.224734
44	1	0	-3.975963	-2.031513	-1.766706
45	1	0	-2.738367	4.298224	1.904637
46	1	0	-3.391567	1.932498	2.318998

Structure 47d (M06-2X, Ethanol)

Energy (Hartrees): =-1168.1614291
No imaginary frequencies

Standard orientation:

Center Number	Atomic Number	Atomic Type	Coordinates (Angstroms) X	Y	Z
1	6	0	4.708096	-0.913618	-1.310194
2	6	0	3.419081	-0.416644	-1.485081
3	6	0	-2.648077	-3.725843	0.884821
4	6	0	5.286850	-0.941160	-0.040457
5	6	0	-1.172568	-1.958708	0.149149
6	6	0	2.726150	0.051966	-0.375071
7	6	0	-2.085626	-1.626343	-0.845703
8	6	0	-3.542572	-3.394380	-0.133580
9	6	0	3.299137	0.024654	0.893475

10	1	0	-1.375481	4.836291	-0.021051
11	6	0	4.586906	-0.472992	1.068809
12	8	0	-1.868961	-0.589955	-1.709719
13	6	0	1.326807	0.626421	-0.334911
14	1	0	1.275683	1.586458	-0.849264
15	6	0	-1.465159	-3.010844	1.014864
16	7	0	0.321137	-0.300494	-0.867801
17	6	0	2.360732	0.597553	1.927747
18	1	0	2.706502	1.583475	2.252711
19	1	0	2.267447	-0.028806	2.817333
20	6	0	1.016856	0.723611	1.195787
21	8	0	0.156202	-0.393834	1.444494
22	1	0	0.469604	1.625953	1.471236
23	1	0	6.289385	-1.335647	0.082942
24	6	0	-3.267174	-2.347400	-1.001073
25	6	0	-1.161440	2.816891	-0.671570
26	8	0	-0.319868	3.266462	-1.651731
27	6	0	-1.662551	3.809206	0.173185
28	6	0	-2.509046	3.479263	1.217365
29	1	0	-2.664712	0.140347	0.753207
30	1	0	-0.612131	0.812753	-2.316646
31	6	0	-0.892316	0.379131	-1.356360
32	1	0	0.952306	-1.895721	0.281394
33	6	0	-2.876840	2.150099	1.416114
34	6	0	0.110184	-1.195381	0.251955
35	1	0	2.962024	-0.401121	-2.468987
36	1	0	5.037699	-0.502886	2.055372
37	1	0	5.264499	-1.283861	-2.163878
38	1	0	-0.235878	2.637311	-2.376353
39	6	0	-2.373081	1.167640	0.577454
40	6	0	-1.492132	1.468994	-0.468122
41	1	0	-0.749135	-3.264744	1.790074
42	1	0	-2.869830	-4.541214	1.562510
43	1	0	-4.465126	-3.951736	-0.249186
44	1	0	-3.952819	-2.065138	-1.791327
45	1	0	-2.890511	4.257919	1.867846
46	1	0	-3.549777	1.879731	2.220705

Structure 47e (M06-2X, Gas Phase)

Energy (Hartrees): =-1168.1246707
No imaginary frequencies

Standard orientation:

Center Number	Atomic Number	Atomic Type	Coordinates (Angstroms) X	Y	Z
1	1	0	-4.172313	-1.972039	1.999571
2	7	0	0.134226	-0.761095	-0.680716
3	1	0	-5.463571	0.061294	1.421803
4	6	0	-1.780348	-2.823889	0.600984
5	6	0	-2.737652	-1.669400	0.421513
6	6	0	-3.071977	0.284048	-0.974579
7	6	0	-0.919760	-2.794130	-0.677993
8	6	0	-2.349585	-0.851296	-0.638402
9	6	0	-3.860321	-1.344658	1.171853
10	1	0	-2.757323	0.917895	-1.797105
11	1	0	-4.771284	1.494243	-0.463546
12	6	0	-1.071966	-1.355917	-1.248387
13	6	0	-4.197105	0.608124	-0.222050
14	6	0	-4.584690	-0.200391	0.844185
15	8	0	1.230444	3.239690	-1.646770
16	1	0	1.405276	-1.940660	-1.906764
17	1	0	-1.077401	4.652447	1.948706
18	6	0	-1.047615	2.512802	1.740327
19	6	0	4.516197	-0.309880	1.238408
20	1	0	0.206737	0.763166	-2.141297
21	1	0	4.691458	-2.205885	2.228806
22	8	0	0.470747	-2.970573	-0.396707
23	1	0	4.073475	1.495258	0.116413
24	6	0	0.125683	1.662393	-0.192621
25	6	0	-0.736751	3.812488	1.354501
26	1	0	2.708164	-3.191424	1.068321
27	1	0	-1.064965	-1.319525	-2.349386
28	1	0	1.810142	2.484883	-1.809663
29	1	0	5.371659	0.118534	1.746300
30	6	0	-0.612543	1.443942	0.965708
31	6	0	2.334059	-1.416629	-0.056975
32	6	0	0.559888	0.548915	-1.120562
33	6	0	3.030974	-2.177611	0.864654
34	6	0	1.130206	-1.816467	-0.840756
35	6	0	2.718096	-0.102184	-0.312476
36	6	0	3.802979	0.468226	0.328783
37	6	0	4.132748	-1.619049	1.510449
38	6	0	0.028569	4.046534	0.219557

39	1	0	-1.124365	-2.656229	1.459850
40	1	0	-2.290497	-3.777882	0.744559
41	1	0	-1.240254	-3.549648	-1.398690
42	8	0	2.003334	0.589725	-1.266209
43	1	0	-1.629379	2.329382	2.634757
44	6	0	0.473185	2.974153	-0.545600
45	1	0	-0.853157	0.425241	1.242519
46	1	0	0.308185	5.047121	-0.085773

Structure 47e (M06-2X, Ethanol)

Energy (Hartrees): =-1167.8466577
No imaginary frequencies

Standard orientation:

Center Number	Atomic Number	Atomic Type	Coordinates (Angstroms) X	Y	Z
1	1	0	-4.305333	-1.232250	2.120856
2	7	0	0.011735	-0.866551	-0.793997
3	1	0	-5.346793	0.858256	1.288479
4	6	0	-2.098346	-2.585607	0.806295
5	6	0	-2.907941	-1.348045	0.483080
6	6	0	-3.044258	0.426702	-1.175227
7	6	0	-1.214801	-2.797676	-0.436913
8	6	0	-2.457745	-0.738259	-0.694270
9	6	0	-3.950757	-0.775090	1.206382
10	1	0	-2.687872	0.888205	-2.087427
11	1	0	-4.559144	1.908773	-0.802969
12	6	0	-1.257697	-1.459780	-1.242116
13	6	0	-4.091957	1.000263	-0.449410
14	6	0	-4.536744	0.404571	0.733953
15	8	0	1.544196	3.058184	-1.617885
16	1	0	1.216244	-2.340433	-1.738793
17	1	0	-0.467058	4.514679	2.164261
18	6	0	-0.746072	2.408805	1.813072
19	6	0	4.503790	-0.402847	1.061846
20	1	0	0.066815	0.711028	-2.200326
21	1	0	4.572127	-2.156398	2.302077
22	8	0	0.201537	-3.000056	-0.063264
23	1	0	4.148773	1.254984	-0.295633
24	6	0	0.252934	1.535351	-0.217203
25	6	0	-0.262001	3.681372	1.506927
26	1	0	2.507572	-3.152472	1.332239
27	1	0	-1.283072	-1.607724	-2.326768
28	1	0	1.981872	2.244106	-1.937383
29	1	0	5.399827	0.027122	1.485819
30	6	0	-0.483722	1.343338	0.951541
31	6	0	2.198270	-1.522966	-0.024491
32	6	0	0.481988	0.430717	-1.227704
33	6	0	2.877150	-2.199553	0.980668
34	6	0	0.965596	-1.972455	-0.733173
35	6	0	2.674409	-0.286754	-0.466174
36	6	0	3.814294	0.292034	0.063330
37	6	0	4.036280	-1.634824	1.522109
38	6	0	0.503000	3.883041	0.359668
39	1	0	-1.458867	-2.420948	1.676375
40	1	0	-2.728369	-3.449233	1.015822
41	1	0	-1.529156	-3.646735	-1.037695
42	8	0	1.946171	0.347596	-1.493631
43	1	0	-1.324275	2.245582	2.711149
44	6	0	0.765771	2.812044	-0.487891
45	1	0	-0.859340	0.353742	1.166463
46	1	0	0.910020	4.853483	0.113876

Structure 47f (M06-2X, Gas Phase)

Energy (Hartrees): =-1168.1342199
No imaginary frequencies

Standard orientation:

Center Number	Atomic Number	Atomic Type	Coordinates (Angstroms) X	Y	Z
1	6	0	1.628105	-1.680734	-0.015623
2	6	0	1.198738	-0.253920	0.214904
3	6	0	0.424247	3.761870	-0.225795
4	6	0	0.915687	-2.585140	-0.796983
5	6	0	-0.498548	1.434106	0.045920
6	6	0	-2.525016	-0.678547	-0.091875
7	6	0	1.349550	-3.896848	-0.939185
8	6	0	-1.171166	-0.658104	0.582397

9	6	0	0.626230	2.383711	-0.227316
10	6	0	2.837508	-2.086314	0.559794
11	6	0	-5.140316	-0.528929	-0.978677
12	6	0	-4.150172	-1.055656	-1.807151
13	6	0	2.517252	-4.306494	-0.302268
14	6	0	1.892161	1.889077	-0.509443
15	6	0	3.267516	-3.403502	0.437172
16	6	0	2.724597	4.114289	-0.824025
17	6	0	-3.507294	-0.136387	0.731394
18	6	0	-1.405364	0.254142	1.826401
19	6	0	-2.832112	-1.129703	-1.368933
20	6	0	-4.824255	-0.060966	0.292795
21	6	0	-2.924165	0.309899	2.050936
22	6	0	1.466068	4.629544	-0.515273
23	6	0	2.944069	2.746011	-0.822195
24	1	0	1.322265	0.001576	1.282990
25	1	0	0.011475	-2.249248	-1.288873
26	1	0	-1.368315	1.689053	-0.568610
27	1	0	2.858934	-5.330113	-0.399068
28	1	0	4.199265	-3.686169	0.910767
29	1	0	3.912258	2.321547	-1.057556
30	1	0	3.541314	4.784419	-1.063757
31	1	0	1.300864	5.699290	-0.511006
32	1	0	-0.564507	4.143783	0.004130
33	1	0	-5.592928	0.364999	0.928183
34	1	0	-6.162323	-0.472634	-1.334423
35	1	0	-4.408958	-1.400668	-2.800950
36	1	0	-2.054930	-1.509944	-2.021921
37	1	0	0.780632	-4.593483	-1.541625
38	1	0	-0.831486	-1.659228	0.847312
39	1	0	-0.863802	-0.072731	2.715949
40	1	0	-3.229061	1.318235	2.336725
41	1	0	-3.219044	-0.370881	2.855579
42	1	0	3.513753	-0.337997	0.882763
43	7	0	-0.159946	0.040605	-0.226037
44	8	0	3.616314	-1.218152	1.264000
45	8	0	2.139987	0.546036	-0.505706
46	8	0	-0.869512	1.510558	1.429681

Structure 47f (M06-2X, Ethanol)

Energy (Hartrees): =-1168.1667723
No imaginary frequencies

Standard orientation:

Center Number	Atomic Number	Atomic Type	Coordinates (Angstroms) X	Y	Z
1	6	0	1.588862	-1.706399	-0.007707
2	6	0	1.190979	-0.267630	0.207135
3	6	0	0.526077	3.763220	-0.237170
4	6	0	0.868779	-2.592603	-0.805560
5	6	0	-0.461807	1.460253	0.033009
6	6	0	-2.546094	-0.630699	-0.088896
7	6	0	1.279145	-3.911862	-0.957044
8	6	0	-1.189745	-0.618287	0.581825
9	6	0	0.689179	2.378501	-0.244059
10	6	0	2.777176	-2.146940	0.588094
11	6	0	-5.167532	-0.459008	-0.963487
12	6	0	-4.192818	-1.031602	-1.781590
13	6	0	2.431403	-4.351791	-0.311025
14	6	0	1.940719	1.848292	-0.531476
15	6	0	3.184968	-3.471057	0.453351
16	6	0	2.840785	4.049665	-0.827888
17	6	0	-3.514811	-0.049384	0.726306
18	6	0	-1.404854	0.304269	1.819515
19	6	0	-2.872089	-1.119339	-1.348986
20	6	0	-4.834408	0.038078	0.294020
21	6	0	-2.920681	0.408676	2.036010
22	6	0	1.595889	4.600674	-0.520339
23	6	0	3.020345	2.674149	-0.834294
24	1	0	1.330424	0.005365	1.266273
25	1	0	-0.029124	-2.245328	-1.301866
26	1	0	-1.326032	1.743227	-0.575620
27	1	0	2.756343	-5.380766	-0.414245
28	1	0	4.100820	-3.784149	0.941231
29	1	0	3.978975	2.224389	-1.065396
30	1	0	3.678669	4.696831	-1.060402
31	1	0	1.461468	5.675414	-0.513512
32	1	0	-0.453037	4.175646	-0.015201
33	1	0	-5.591868	0.492962	0.923760
34	1	0	-6.191428	-0.394219	-1.314418
35	1	0	-4.465786	-1.404984	-2.762034
36	1	0	-2.113013	-1.550928	-1.992580

37	1	0	0.701460	-4.590555	-1.572722
38	1	0	-0.866033	-1.620538	0.859862
39	1	0	-0.876555	-0.035373	2.710394
40	1	0	-3.207731	1.427492	2.304613
41	1	0	-3.232317	-0.250298	2.851908
42	1	0	3.468803	-0.404894	1.032806
43	7	0	-0.163346	0.055850	-0.234979
44	8	0	3.564624	-1.317981	1.334148
45	8	0	2.138969	0.495631	-0.547087
46	8	0	-0.823269	1.549187	1.420880

Structure 47g (M06-2X, Gas Phase)

Energy (Hartrees): =-1168.1228889
No imaginary frequencies

Standard orientation:

Center Number	Atomic Number	Atomic Type	Coordinates (Angstroms) X	Y	Z
1	6	0	-0.271713	3.062100	-0.516895
2	6	0	-1.853844	-3.043293	0.822595
3	6	0	3.218233	-0.927494	0.314191
4	8	0	-1.472508	-0.220249	-1.506712
5	6	0	2.606596	0.316252	0.134147
6	6	0	5.011529	-0.236523	-1.119694
7	1	0	-0.028874	5.155331	-0.099869
8	1	0	-2.281127	5.355872	0.942949
9	6	0	3.201986	1.293275	-0.649636
10	1	0	-3.775511	3.367342	1.028574
11	6	0	4.422187	-1.209296	-0.313806
12	6	0	-0.715339	-0.772720	1.115104
13	1	0	-2.633763	-2.069012	-2.843323
14	6	0	1.311060	0.367891	0.904212
15	1	0	1.102160	1.338236	1.376145
16	7	0	0.181192	-0.156475	0.149363
17	6	0	4.410255	1.007450	-1.283478
18	6	0	-0.470404	0.604301	-0.880221
19	1	0	5.953817	-0.445172	-1.612404
20	1	0	4.905162	-2.169978	-0.172524
21	6	0	-2.362605	2.067357	0.114674
22	1	0	-3.335867	-4.290812	-1.958503
23	6	0	-1.483333	-1.798683	0.336157
24	1	0	0.282296	0.818681	-1.645153
25	6	0	-1.763436	-1.464562	-0.990295
26	8	0	-3.249204	1.040420	0.215720
27	6	0	-2.778062	3.301826	0.611888
28	6	0	-2.525335	-3.942734	0.001207
29	6	0	-0.679979	4.293716	-0.025913
30	6	0	-2.814058	-3.593564	-1.314288
31	1	0	2.752708	2.274188	-0.753589
32	1	0	4.888510	1.760782	-1.897672
33	8	0	0.132248	-1.315785	2.104547
34	1	0	1.827902	-0.466179	2.878444
35	6	0	-2.429775	-2.356991	-1.819604
36	1	0	1.802782	-2.513445	0.657805
37	1	0	3.009257	-2.358550	1.945830
38	6	0	1.442945	-0.799785	1.913376
39	6	0	2.400793	-1.800446	1.232613
40	6	0	-1.940264	4.403935	0.553587
41	6	0	-1.080075	1.927451	-0.433750
42	1	0	-1.598900	-3.297440	1.844760
43	1	0	-1.391191	-0.047860	1.598951
44	1	0	-3.058075	0.397540	-0.476216
45	1	0	-2.818943	-4.912239	0.383322
46	1	0	0.700864	2.966169	-0.988543

Structure 47g (M06-2X, Ethanol)

Energy (Hartrees): =-1168.1534594
No imaginary frequencies

Standard orientation:

Center Number	Atomic Number	Atomic Type	Coordinates (Angstroms) X	Y	Z
1	6	0	-0.197957	3.033769	-0.447425
2	6	0	-2.098333	-2.888166	0.836598
3	6	0	3.377026	-0.940585	0.431650
4	8	0	-1.467890	-0.184425	-1.586211
5	6	0	2.674114	0.198855	0.034731
6	6	0	5.123045	-0.389163	-1.119841

7	1	0	0.106258	5.098656	0.059385
8	1	0	-2.131230	5.316687	1.137642
9	6	0	3.192412	1.054244	-0.930470
10	1	0	-3.674127	3.367215	1.159845
11	6	0	4.605705	-1.240619	-0.144891
12	6	0	-0.656195	-0.791472	1.032174
13	1	0	-2.940541	-1.878648	-2.806015
14	6	0	1.366396	0.304266	0.778419
15	1	0	1.172413	1.304193	1.185338
16	7	0	0.226990	-0.222464	0.026625
17	6	0	4.423025	0.751363	-1.509870
18	6	0	-0.455186	0.608024	-0.937114
19	1	0	6.082527	-0.611093	-1.573615
20	1	0	5.158863	-2.122165	0.162372
21	6	0	-2.304821	2.068719	0.169768
22	1	0	-3.868714	-3.975556	-1.843140
23	6	0	-1.586156	-1.711806	0.303418
24	1	0	0.267907	0.863319	-1.714994
25	6	0	-1.893527	-1.361051	-1.013484
26	8	0	-3.221392	1.054150	0.249844
27	6	0	-2.684227	3.289513	0.724753
28	6	0	-2.922829	-3.706144	0.070621
29	6	0	-0.569475	4.253157	0.102867
30	6	0	-3.228722	-3.343072	-1.238586
31	1	0	2.658273	1.949270	-1.227170
32	1	0	4.841257	1.410434	-2.262059
33	8	0	0.216436	-1.466142	1.909151
34	1	0	1.644209	-0.320929	2.869256
35	6	0	-2.715269	-2.173582	-1.787690
36	1	0	2.210299	-2.635465	1.073111
37	1	0	3.261073	-1.982287	2.334331
38	6	0	1.474213	-0.775953	1.892345
39	6	0	2.626774	-1.713067	1.488031
40	6	0	-1.819174	4.373799	0.703386
41	6	0	-1.035643	1.915690	-0.405017
42	1	0	-1.840988	-3.159816	1.854546
43	1	0	-1.205571	-0.016707	1.594732
44	1	0	-3.170102	0.509811	-0.545295
45	1	0	-3.321085	-4.621976	0.490506
46	1	0	0.771942	2.933325	-0.923653

Structure 47h (M06-2X, Gas Phase)

Energy (Hartrees): =-1168.1250525
No imaginary frequencies

Standard orientation:

Center Number	Atomic Number	Atomic Type	Coordinates (Angstroms) X	Y	Z
1	6	0	2.924785	-1.129257	1.593627
2	1	0	2.808256	-2.187898	1.343564
3	6	0	-3.273853	3.403753	0.868284
4	1	0	5.099905	1.783954	-2.341358
5	1	0	0.965275	-2.167324	-0.342535
6	6	0	1.457211	0.514672	0.409130
7	1	0	1.095902	1.509906	0.686447
8	6	0	-3.048937	3.405452	-0.507455
9	6	0	0.229755	-1.457072	0.059517
10	6	0	-1.754272	1.527372	1.124946
11	1	0	-0.086269	1.296288	-1.806331
12	6	0	-3.011902	-3.433817	0.138525
13	6	0	1.538776	-0.451365	1.641534
14	6	0	3.679831	-0.383451	0.519730
15	6	0	-1.736678	-2.996830	0.467018
16	6	0	4.693960	1.176918	-1.541102
17	6	0	-2.169169	2.481248	-1.036811
18	1	0	5.653806	-1.238470	0.655591
19	6	0	-1.498064	1.530486	-0.253352
20	1	0	3.428854	-1.079085	2.560804
21	1	0	-3.705602	-1.196867	-2.338615
22	6	0	-2.633713	2.476610	1.664960
23	6	0	5.513140	0.258103	-0.885421
24	6	0	-0.567471	0.632619	-1.084420
25	1	0	1.363075	0.070015	2.580440
26	6	0	-1.167393	-1.925803	-0.217238
27	8	0	-1.349645	-0.235036	-1.897522
28	7	0	0.494070	-0.139717	-0.469466
29	8	0	0.453137	-1.359595	1.472153
30	6	0	2.865617	0.535182	-0.134731
31	6	0	-1.892975	-1.285853	-1.218119
32	1	0	-0.858940	-0.130457	1.694699
33	1	0	2.723534	2.037074	-1.671932
34	1	0	6.550079	0.157366	-1.183271
35	6	0	-3.719783	-2.799038	-0.881534

36	1	0	-4.715195	-3.138728	-1.141068
37	6	0	5.012238	-0.526570	0.147848
38	1	0	-1.170677	-3.475760	1.257860
39	1	0	-3.455183	-4.264997	0.672106
40	6	0	-3.169667	-1.720289	-1.557111
41	1	0	-3.951560	4.121377	1.315422
42	1	0	-3.544853	4.120932	-1.150812
43	1	0	-2.793745	2.442346	2.735473
44	8	0	-1.189761	0.709706	2.045792
45	1	0	-1.980447	2.476272	-2.106171
46	6	0	3.362806	1.320508	-1.167399

Structure 47h (M06-2X, Ethanol)

Energy (Hartrees): =-1168.1626324
No imaginary frequencies

Standard orientation:

Center Number	Atomic Number	Atomic Type	Coordinates (Angstroms) X	Y	Z
1	6	0	2.399393	0.659566	1.923262
2	1	0	2.337159	-0.067852	2.735518
3	6	0	-2.689472	3.893658	0.492749
4	1	0	5.385699	-0.661439	-2.325433
5	1	0	1.183983	-1.742766	0.233049
6	6	0	1.334892	0.785451	-0.332402
7	1	0	1.190995	1.758886	-0.802154
8	6	0	-1.939939	3.992100	-0.674125
9	6	0	0.268909	-1.145196	0.192511
10	6	0	-2.215256	1.523132	0.601767
11	1	0	-0.623655	0.819981	-2.342352
12	6	0	-2.185572	-3.985601	0.712886
13	6	0	1.046500	0.796638	1.210455
14	6	0	3.362439	0.247746	0.836646
15	6	0	-1.063961	-3.178969	0.842443
16	6	0	4.812228	-0.403211	-1.442319
17	6	0	-1.357879	2.847862	-1.204664
18	1	0	5.151362	-0.228622	1.943115
19	6	0	-1.481826	1.595307	-0.593794
20	1	0	2.697157	1.617151	2.359442
21	1	0	-3.808233	-2.211268	-1.705588
22	6	0	-2.820603	2.664761	1.124444
23	6	0	5.407733	-0.482089	-0.182155
24	6	0	-0.864337	0.416885	-1.358659
25	1	0	0.479199	1.666682	1.539686
26	6	0	-0.926131	-2.029273	0.068007
27	8	0	-1.842442	-0.582967	-1.626718
28	7	0	0.391102	-0.195473	-0.883498
29	8	0	0.208706	-0.357534	1.410475
30	6	0	2.772800	0.327109	-0.421686
31	6	0	-1.924307	-1.688966	-0.836946
32	1	0	-1.511068	-0.026945	1.475184
33	1	0	3.017345	0.058162	-2.546499
34	1	0	6.439819	-0.803412	-0.096712
35	6	0	-3.173586	-3.639842	-0.210142
36	1	0	-4.051666	-4.265331	-0.324104
37	6	0	4.687544	-0.158460	0.964657
38	1	0	-0.276005	-3.433873	1.544038
39	1	0	-2.288956	-4.879037	1.316243
40	6	0	-3.048588	-2.498220	-0.988031
41	1	0	-3.164313	4.770644	0.917679
42	1	0	-1.822076	4.944666	-1.176540
43	1	0	-3.385285	2.562102	2.044185
44	8	0	-2.393000	0.355692	1.291005
45	1	0	-0.799595	2.918925	-2.133344
46	6	0	3.486403	0.001301	-1.569742

Structure 47i (M06-2X, Gas Phase)

Energy (Hartrees): =-1168.1258506
No imaginary frequencies

Standard orientation:

Center Number	Atomic Number	Atomic Type	Coordinates (Angstroms) X	Y	Z
1	6	0	-3.688378	-0.308483	0.893692
2	6	0	-4.472645	-0.143222	-0.244525
3	6	0	-4.060566	-0.650921	-1.475005
4	6	0	-2.842788	-1.316283	-1.584965
5	6	0	-2.054979	-1.459548	-0.451358

6	6	0	-2.477695	-0.979480	0.787659
7	6	0	-0.697519	-2.093962	-0.350749
8	6	0	-0.399331	-2.133757	1.189352
9	6	0	-1.478637	-1.279795	1.881346
10	6	0	-1.754094	3.232816	0.877630
11	6	0	-0.667829	2.626160	1.483391
12	6	0	1.500785	-1.670128	-0.004115
13	6	0	2.689004	-0.776246	-0.129552
14	6	0	0.278495	0.060019	-1.294824
15	6	0	0.018425	1.573645	0.870357
16	6	0	2.631902	0.292588	-1.017368
17	6	0	3.736189	1.123432	-1.188977
18	6	0	4.884342	0.891607	-0.449460
19	6	0	4.944488	-0.162600	0.461832
20	6	0	3.846299	-0.994464	0.612724
21	6	0	-0.396419	1.110261	-0.391685
22	6	0	-1.496616	1.748722	-0.978319
23	6	0	-2.179714	2.787689	-0.368857
24	1	0	-4.018090	0.086105	1.848315
25	1	0	-5.419193	0.379361	-0.171439
26	1	0	-4.690913	-0.527203	-2.347491
27	1	0	-2.509674	-1.709108	-2.540110
28	1	0	-0.680074	-3.099208	-0.781521
29	1	0	-0.397018	-3.160676	1.562909
30	1	0	-1.945095	-1.818319	2.708822
31	1	0	-1.044385	-0.365220	2.293642
32	1	0	1.786983	-2.718696	-0.160012
33	1	0	-0.341898	0.033956	-2.189957
34	1	0	3.661102	1.943538	-1.891430
35	1	0	5.738973	1.545216	-0.577125
36	1	0	5.842015	-0.332939	1.042296
37	1	0	3.871602	-1.823490	1.311892
38	1	0	-1.829235	1.408528	-1.952963
39	1	0	-3.028669	3.243639	-0.862682
40	1	0	-2.265259	4.047471	1.377372
41	1	0	-0.307059	2.946406	2.453194
42	1	0	1.111859	0.123869	1.532563
43	7	0	0.442680	-1.350584	-0.915275
44	8	0	0.915202	-1.595129	1.322791
45	8	0	1.527712	0.546857	-1.766751
46	8	0	1.080705	1.096090	1.566625

Structure 47i (M06-2X, Ethanol)

Energy (Hartrees): =-1168.1585489
No imaginary frequencies

Standard orientation:

Center Number	Atomic Number	Atomic Type	Coordinates (Angstroms) X	Y	Z
1	6	0	-3.706459	-0.365879	0.919237
2	6	0	-4.488781	-0.132503	-0.210643
3	6	0	-4.067934	-0.559361	-1.470349
4	6	0	-2.846963	-1.213998	-1.617796
5	6	0	-2.062134	-1.427217	-0.491617
6	6	0	-2.491042	-1.024438	0.773801
7	6	0	-0.711220	-2.082853	-0.421208
8	6	0	-0.411585	-2.190757	1.113223
9	6	0	-1.499042	-1.391669	1.851856
10	6	0	-1.719929	3.239578	0.938457
11	6	0	-0.657347	2.588925	1.545091
12	6	0	1.494138	-1.677655	-0.052354
13	6	0	2.684716	-0.781053	-0.146900
14	6	0	0.280734	0.088458	-1.304173
15	6	0	0.017986	1.546181	0.905336
16	6	0	2.640385	0.312112	-1.006524
17	6	0	3.742718	1.152438	-1.141440
18	6	0	4.883665	0.902983	-0.392074
19	6	0	4.933319	-0.177816	0.488935
20	6	0	3.834483	-1.018208	0.602514
21	6	0	-0.377752	1.132894	-0.379547
22	6	0	-1.450101	1.816948	-0.968669
23	6	0	-2.125507	2.848602	-0.333489
24	1	0	-4.044318	-0.040674	1.897689
25	1	0	-5.440020	0.377943	-0.107729
26	1	0	-4.693656	-0.379176	-2.337095
27	1	0	-2.511116	-1.544916	-2.595318
28	1	0	-0.710743	-3.069936	-0.888618
29	1	0	-0.383278	-3.233081	1.434111
30	1	0	-1.966970	-1.989403	2.636352
31	1	0	-1.077930	-0.502919	2.331101
32	1	0	1.779919	-2.720498	-0.230765
33	1	0	-0.339495	0.093157	-2.198578
34	1	0	3.681898	1.991612	-1.824418

35	1	0	5.738826	1.561816	-0.491060
36	1	0	5.825513	-0.365656	1.073715
37	1	0	3.857721	-1.871289	1.273054
38	1	0	-1.766431	1.519075	-1.962900
39	1	0	-2.954089	3.341331	-0.828379
40	1	0	-2.228908	4.043267	1.458588
41	1	0	-0.320665	2.869296	2.536778
42	1	0	1.042921	0.031984	1.547862
43	7	0	0.441556	-1.330998	-0.965660
44	8	0	0.900430	-1.631303	1.271508
45	8	0	1.539819	0.581453	-1.766468
46	8	0	1.064981	1.011197	1.598114

Structure 47j (M06-2X, Ethanol)

Energy (Hartrees): =-1168.1487451
No imaginary frequencies

Standard orientation:

Center Number	Atomic Number	Atomic Type	Coordinates (Angstroms) X	Y	Z
1	1	0	-0.829170	-1.376654	-2.448058
2	8	0	1.105500	-2.542982	-0.280334
3	6	0	1.089085	-1.181539	0.124569
4	7	0	0.398425	-0.552118	-0.969056
5	6	0	-1.226990	2.806291	1.547764
6	6	0	2.475461	-0.624143	0.224778
7	1	0	-2.769969	4.116821	-1.174329
8	1	0	-4.793602	0.669537	-0.115767
9	6	0	-0.322915	1.841046	-0.484483
10	1	0	0.209690	0.948128	-2.320606
11	6	0	0.513644	0.835372	-1.276216
12	6	0	5.052997	0.422270	0.342697
13	6	0	-0.318486	1.967251	0.910371
14	6	0	-2.094719	3.510044	-0.582925
15	6	0	-2.120693	3.568842	0.806219
16	6	0	3.454637	-1.245589	0.999203
17	1	0	-2.821612	4.220117	1.315674
18	1	0	0.555908	-1.113336	1.085036
19	6	0	-2.020288	-1.202322	-0.632865
20	1	0	6.054605	0.834492	0.385455
21	8	0	1.900588	1.229272	-1.247720
22	1	0	5.497115	-1.224731	1.661916
23	6	0	4.741663	-0.733336	1.061126
24	6	0	-2.257491	-2.213250	0.294608
25	1	0	3.190990	-2.143470	1.548557
26	1	0	-1.205245	2.857446	2.630368
27	1	0	-2.784346	0.587058	-1.562513
28	6	0	-0.706056	-1.410149	-1.362240
29	6	0	-1.199909	2.650258	-1.208300
30	6	0	-3.391066	-2.177795	1.102690
31	6	0	-4.072432	-0.131323	0.004139
32	1	0	-5.174987	-1.084036	1.586248
33	1	0	4.313868	1.957214	-0.985462
34	6	0	-2.940096	-0.171230	-0.804292
35	6	0	-4.289772	-1.124623	0.961200
36	6	0	4.089943	1.056406	-0.425960
37	1	0	-3.574769	-2.963574	1.828204
38	6	0	2.795762	0.533828	-0.487226
39	6	0	-1.193954	-3.278808	0.241614
40	1	0	-1.623613	-4.246626	-0.027569
41	6	0	-0.190368	-2.797445	-0.828788
42	1	0	-0.683654	-3.404086	1.200429
43	1	0	-0.057489	-3.524462	-1.626834
44	8	0	0.528278	1.257485	1.724092
45	1	0	1.421848	1.228903	1.359865
46	1	0	-1.197220	2.575544	-2.291624

Structure 47k (M06-2X, Gas Phase)

Energy (Hartrees): =-1168.1279824
No imaginary frequencies

Standard orientation:

Center Number	Atomic Number	Atomic Type	Coordinates (Angstroms) X	Y	Z
1	6	0	-3.110048	-1.898738	1.122102
2	6	0	-4.146822	-1.203549	0.511911
3	6	0	-3.961263	-0.591201	-0.729616
4	6	0	-2.727844	-0.647370	-1.361700

5	6	0	-1.680213	-1.307119	-0.727924
6	6	0	-0.623288	-2.685688	0.934439
7	6	0	5.148989	1.259333	0.629398
8	6	0	-1.871981	-1.956560	0.489799
9	6	0	-0.754454	1.351892	0.001018
10	6	0	-0.266798	-1.479638	-1.210245
11	6	0	4.363769	-0.829356	-0.261924
12	6	0	3.862360	1.772177	0.594368
13	6	0	0.301173	-2.613658	-0.296851
14	6	0	5.405525	-0.046707	0.210853
15	6	0	-1.682831	1.446159	1.040602
16	6	0	-0.965574	2.084610	-1.166582
17	6	0	-2.816410	2.245255	0.887606
18	6	0	-3.019206	2.951861	-0.284790
19	6	0	2.821944	0.968219	0.133020
20	6	0	1.934121	-1.129695	-0.861069
21	6	0	0.508340	0.537192	0.098983
22	6	0	3.066589	-0.324264	-0.310761
23	6	0	-2.091056	2.876175	-1.323061
24	1	0	-4.783084	-0.062267	-1.197275
25	1	0	-2.570792	-0.155397	-2.314625
26	1	0	-5.113724	-1.145981	0.997758
27	1	0	-3.265686	-2.389255	2.076520
28	1	0	-0.227796	-1.757945	-2.266074
29	1	0	0.326696	-3.569723	-0.828530
30	1	0	-0.128583	-2.199076	1.782463
31	1	0	2.207724	-1.553589	-1.836438
32	1	0	-0.838387	-3.712608	1.235583
33	1	0	3.634875	2.778996	0.920922
34	1	0	-3.905124	3.567349	-0.389044
35	1	0	-2.242816	3.435625	-2.237539
36	1	0	-0.225319	2.001832	-1.954282
37	1	0	-3.522002	2.285904	1.708131
38	1	0	-0.848873	0.161296	2.195145
39	1	0	5.959725	1.880253	0.991344
40	1	0	6.411970	-0.443454	0.248712
41	1	0	4.544272	-1.843434	-0.602066
42	1	0	0.580761	-0.030191	1.043342
43	7	0	0.705123	-0.365762	-1.042830
44	8	0	1.616146	-2.203901	0.031919
45	8	0	1.565753	1.489045	0.114865
46	8	0	-1.569378	0.794359	2.229254

Structure 47k (M06-2X, Ethanol)

Energy (Hartrees): =-1168.164499
No imaginary frequencies

Standard orientation:

Center Number	Atomic Number	Atomic Type	Coordinates (Angstroms) X	Y	Z
1	6	0	-3.244500	-1.849660	1.030878
2	6	0	-4.257899	-1.188956	0.342740
3	6	0	-4.008035	-0.593925	-0.897253
4	6	0	-2.733966	-0.635196	-1.451342
5	6	0	-1.715657	-1.267969	-0.744679
6	6	0	-0.729840	-2.565877	1.021503
7	6	0	5.225156	1.182251	0.520891
8	6	0	-1.968701	-1.893193	0.475907
9	6	0	-0.699096	1.379420	0.041108
10	6	0	-0.286204	-1.476260	-1.161952
11	6	0	4.359748	-0.926205	-0.248948
12	6	0	3.953467	1.734935	0.476138
13	6	0	0.224747	-2.579280	-0.184929
14	6	0	5.433104	-0.152588	0.169296
15	6	0	-1.620877	1.437684	1.091808
16	6	0	-0.933419	2.142061	-1.104673
17	6	0	-2.767888	2.225878	0.967750
18	6	0	-2.993080	2.959079	-0.185906
19	6	0	2.882128	0.940461	0.072667
20	6	0	1.914770	-1.177740	-0.808202
21	6	0	0.559173	0.556032	0.104424
22	6	0	3.077286	-0.382681	-0.305224
23	6	0	-2.070705	2.924621	-1.231719
24	1	0	-4.813189	-0.097354	-1.427437
25	1	0	-2.533685	-0.172819	-2.412136
26	1	0	-5.256362	-1.148634	0.763853
27	1	0	-3.447819	-2.329067	1.982669
28	1	0	-0.216824	-1.805616	-2.200327
29	1	0	0.232464	-3.557321	-0.672887
30	1	0	-0.290047	-1.979468	1.835803
31	1	0	2.168410	-1.663928	-1.757109
32	1	0	-0.935847	-3.565750	1.406614
33	1	0	3.769160	2.766659	0.751878

34	1	0	-3.890329	3.562308	-0.267207
35	1	0	-2.238695	3.502204	-2.132703
36	1	0	-0.207526	2.098480	-1.909367
37	1	0	-3.469397	2.242879	1.794204
38	1	0	-0.683818	0.242673	2.293364
39	1	0	6.061318	1.795460	0.836965
40	1	0	6.428057	-0.578602	0.212442
41	1	0	4.506832	-1.959355	-0.547125
42	1	0	0.669695	0.024332	1.060278
43	7	0	0.712392	-0.377077	-1.022073
44	8	0	1.556073	-2.192608	0.143316
45	8	0	1.636139	1.496804	0.041838
46	8	0	-1.496890	0.758943	2.264976

Structure 47l (M06-2X, Gas Phase)

Energy (Hartrees): =-1168.1262123
No imaginary frequencies

Standard orientation:

Center Number	Atomic Number	Atomic Type	Coordinates (Angstroms) X	Y	Z
1	6	0	2.892874	-3.302516	-0.877475
2	6	0	3.642798	-2.473273	-1.705037
3	6	0	3.533560	-1.087410	-1.604887
4	6	0	2.660680	-0.513480	-0.685934
5	6	0	0.871746	3.004445	-0.649379
6	6	0	1.164820	-3.424495	1.071903
7	6	0	1.889110	-1.345351	0.121788
8	6	0	2.018595	-2.729975	0.039246
9	6	0	0.871715	-0.950229	1.178705
10	6	0	0.268660	-2.315575	1.647927
11	6	0	-3.884165	1.031503	-0.751728
12	6	0	-3.648513	-1.644720	-0.009638
13	6	0	0.835638	2.223572	1.626904
14	6	0	0.518416	2.026547	0.286708
15	6	0	-1.127814	-1.341893	0.142012
16	6	0	-0.235979	0.827289	-0.220937
17	6	0	1.639537	4.098732	-0.264861
18	6	0	-2.513567	-0.852728	-0.115512
19	6	0	-2.634229	0.486605	-0.479483
20	6	0	-4.901479	-1.109787	-0.282222
21	6	0	2.003275	4.248147	1.066417
22	6	0	1.583085	3.326570	2.021058
23	6	0	-5.011726	0.226862	-0.655867
24	1	0	2.992410	-4.380466	-0.944202
25	1	0	0.546950	-4.223611	0.657600
26	1	0	1.794719	-3.861253	1.852568
27	1	0	-0.666349	-1.687993	-0.804174
28	1	0	0.160614	-2.379898	2.729895
29	1	0	4.325444	-2.906472	-2.426668
30	1	0	4.134687	-0.451923	-2.244079
31	1	0	2.592917	0.566078	-0.597774
32	1	0	1.341538	-0.398685	1.992880
33	1	0	0.474520	1.511204	2.358253
34	1	0	-3.534057	-2.676699	0.299908
35	1	0	-3.950896	2.077865	-1.022079
36	1	0	-5.985523	0.651985	-0.867707
37	1	0	1.913433	4.826962	-1.017918
38	1	0	2.595500	5.105634	1.363466
39	1	0	1.831663	3.468761	3.065003
40	1	0	-5.786663	-1.727571	-0.199813
41	1	0	0.221152	0.485463	-1.166227
42	1	0	-0.364269	2.462551	-1.994311
43	7	0	-0.342229	-0.255782	0.720117
44	8	0	0.492093	2.905674	-1.954500
45	8	0	-1.550179	1.315413	-0.576121
46	8	0	-1.049966	-2.361651	1.096411

Structure 47l (M06-2X, Ethanol)

Energy (Hartrees): =-1168.1589634
No imaginary frequencies

Standard orientation:

Center Number	Atomic Number	Atomic Type	Coordinates (Angstroms) X	Y	Z
1	6	0	2.748903	-3.538068	-0.441286
2	6	0	3.469510	-2.917166	-1.458630
3	6	0	3.353839	-1.543807	-1.669269

4	6	0	2.511179	-0.774423	-0.868970
5	6	0	1.445536	2.750298	-0.730348
6	6	0	1.072605	-3.215320	1.532260
7	6	0	1.780442	-1.397535	0.138605
8	6	0	1.906121	-2.770459	0.355451
9	6	0	0.802486	-0.781814	1.130613
10	6	0	0.146376	-2.027013	1.797942
11	6	0	-3.974239	1.155039	-0.748167
12	6	0	-3.678074	-1.563697	-0.224157
13	6	0	0.435851	2.523410	1.442027
14	6	0	0.553512	2.087163	0.118124
15	6	0	-1.178849	-1.230022	0.082122
16	6	0	-0.336652	0.963162	-0.346678
17	6	0	2.226805	3.799848	-0.238900
18	6	0	-2.561715	-0.733329	-0.207615
19	6	0	-2.713815	0.630346	-0.470975
20	6	0	-4.938604	-1.046885	-0.498173
21	6	0	2.107667	4.203611	1.079542
22	6	0	1.204132	3.566905	1.931118
23	6	0	-5.079750	0.314929	-0.760110
24	1	0	2.843881	-4.605755	-0.272736
25	1	0	0.499625	-4.124737	1.340802
26	1	0	1.710570	-3.400283	2.402000
27	1	0	-0.728680	-1.657726	-0.831378
28	1	0	-0.072148	-1.875575	2.854077
29	1	0	4.126468	-3.505588	-2.089558
30	1	0	3.923297	-1.068845	-2.460218
31	1	0	2.441230	0.293408	-1.041061
32	1	0	1.303299	-0.131045	1.849282
33	1	0	-0.272113	2.015904	2.088257
34	1	0	-3.548695	-2.619439	-0.010877
35	1	0	-4.066936	2.216888	-0.944701
36	1	0	-6.058367	0.728652	-0.975430
37	1	0	2.917097	4.285825	-0.918774
38	1	0	2.719830	5.020651	1.444108
39	1	0	1.101424	3.884475	2.961464
40	1	0	-5.804426	-1.697837	-0.505383
41	1	0	-0.066535	0.600978	-1.350655
42	1	0	1.005489	1.785012	-2.355370
43	7	0	-0.387587	-0.127252	0.594223
44	8	0	1.647672	2.430501	-2.038961
45	8	0	-1.665696	1.510519	-0.452820
46	8	0	-1.117075	-2.189175	1.115459

Structure 47m (M06-2X, Gas Phase)

Energy (Hartrees): =-1168.126654
No imaginary frequencies

Standard orientation:

Center Number	Atomic Number	Atomic Type	Coordinates (Angstroms) X	Y	Z
1	6	0	-3.771668	-0.695777	1.050139
2	6	0	-4.271093	0.476504	0.493922
3	6	0	-3.764782	0.957943	-0.712206
4	6	0	-2.743010	0.279119	-1.366318
5	6	0	-2.216707	-0.870493	-0.788441
6	6	0	-2.743730	-1.367527	0.400373
7	6	0	-1.062842	-1.720971	-1.272340
8	6	0	-0.877955	-2.787579	-0.132829
9	6	0	-2.062865	-2.644034	0.827914
10	6	0	-0.787783	2.910746	1.895966
11	6	0	-0.346434	3.430180	0.686978
12	6	0	1.141705	-1.891377	-0.522853
13	6	0	2.328529	-1.123193	-0.021416
14	6	0	0.452162	0.340400	-1.331911
15	6	0	0.101861	2.568727	-0.307692
16	6	0	2.663128	0.072499	-0.653540
17	6	0	3.815873	0.764293	-0.305333
18	6	0	4.634897	0.257602	0.693959
19	6	0	4.297811	-0.921831	1.353872
20	6	0	3.148249	-1.610965	0.991100
21	6	0	0.069503	1.180647	-0.119598
22	6	0	-0.327091	0.681872	1.116434
23	6	0	-0.758013	1.538509	2.121926
24	1	0	5.532852	0.796468	0.971167
25	1	0	4.043445	1.693850	-0.812232
26	1	0	4.931250	-1.302111	2.145196
27	1	0	2.870960	-2.532894	1.489352
28	1	0	-1.300668	-2.198188	-2.228771
29	1	0	-0.776693	-3.797203	-0.543551
30	1	0	1.495331	-2.743354	-1.125544
31	1	0	-0.075604	0.748948	-2.197020
32	1	0	-5.062955	1.017893	0.997899

33	1	0	-4.172907	-1.075394	1.983599
34	1	0	-2.354718	0.660763	-2.304712
35	1	0	-4.166367	1.868255	-1.140397
36	1	0	-0.324113	4.495546	0.494441
37	1	0	-1.136619	3.583001	2.671047
38	1	0	-2.744420	-3.494272	0.741319
39	1	0	-1.702964	-2.611140	1.859299
40	1	0	1.227954	2.542410	-1.845382
41	1	0	-1.076430	1.134095	3.074749
42	1	0	-0.299506	-0.386180	1.290250
43	7	0	0.263636	-1.090275	-1.348414
44	8	0	0.542039	3.114990	-1.480202
45	8	0	0.333718	-2.423281	0.517042
46	8	0	1.840274	0.593250	-1.620645

Structure 47m (M06-2X, Ethanol)

Energy (Hartrees): =-1168.1575276
No imaginary frequencies

Standard orientation:

Center Number	Atomic Number	Atomic Type	Coordinates (Angstroms) X	Y	Z
1	6	0	-3.456546	-0.701870	1.047291
2	6	0	-4.212773	0.031553	0.134018
3	6	0	-3.857724	0.071543	-1.214043
4	6	0	-2.723243	-0.601137	-1.660986
5	6	0	-1.954166	-1.305156	-0.741055
6	6	0	-2.331020	-1.384144	0.599743
7	6	0	-0.641281	-2.005072	-0.977574
8	6	0	-0.295807	-2.668824	0.399442
9	6	0	-1.379728	-2.236034	1.403536
10	6	0	-1.417787	2.417348	2.124986
11	6	0	-1.708146	2.783594	0.818646
12	6	0	1.581106	-1.675807	-0.452378
13	6	0	2.676368	-0.685645	-0.185505
14	6	0	0.341229	0.324510	-1.243979
15	6	0	-1.092089	2.129010	-0.245503
16	6	0	2.637223	0.560995	-0.807208
17	6	0	3.668513	1.478423	-0.628641
18	6	0	4.741700	1.146624	0.186942
19	6	0	4.785271	-0.089919	0.830468
20	6	0	3.755486	-1.001667	0.637558
21	6	0	-0.213772	1.055975	-0.022362
22	6	0	0.088788	0.730386	1.302129
23	6	0	-0.500108	1.400286	2.368461
24	1	0	5.543408	1.862027	0.329129
25	1	0	3.606814	2.439196	-1.126334
26	1	0	5.621161	-0.342169	1.471697
27	1	0	3.782120	-1.972417	1.121576
28	1	0	-0.707818	-2.752389	-1.771596
29	1	0	-0.233718	-3.755099	0.304027
30	1	0	1.988933	-2.546515	-0.983987
31	1	0	-0.319255	0.538268	-2.081528
32	1	0	-5.090209	0.571268	0.473056
33	1	0	-3.743993	-0.738621	2.093202
34	1	0	-2.434973	-0.559602	-2.706680
35	1	0	-4.461164	0.638065	-1.914097
36	1	0	-2.399418	3.588235	0.595418
37	1	0	-1.898542	2.933980	2.948018
38	1	0	-1.893707	-3.101480	1.827743
39	1	0	-0.933071	-1.679146	2.231663
40	1	0	-0.675289	2.525301	-2.097127
41	1	0	-0.251875	1.115884	3.384470
42	1	0	0.759526	-0.091146	1.509718
43	7	0	0.514198	-1.123693	-1.251910
44	8	0	-1.432359	2.563733	-1.501087
45	8	0	0.989442	-2.158105	0.759287
46	8	0	1.592007	0.912169	-1.619316

Structure 47n (M06-2X, Gas Phase)

Energy (Hartrees): =-1168.1159828
No imaginary frequencies

Standard orientation:

Center Number	Atomic Number	Atomic Type	Coordinates (Angstroms) X	Y	Z
1	1	0	-0.803503	-1.415543	-2.441347
2	1	0	-0.631248	2.851593	-2.443611

3	8	0	1.102569	-2.588763	-0.216347
4	6	0	1.104051	-1.221998	0.141421
5	7	0	0.450927	-0.605443	-0.979769
6	6	0	-0.672107	2.358147	2.122240
7	6	0	2.484870	-0.653313	0.266196
8	1	0	-2.624634	4.053197	-0.082564
9	1	0	0.834991	0.950557	1.577954
10	1	0	-4.642230	0.763194	-0.000709
11	6	0	-0.182442	1.699209	-0.162683
12	1	0	0.139829	0.998743	-2.164541
13	6	0	0.539435	0.806077	-1.166635
14	6	0	5.041686	0.425572	0.442427
15	6	0	0.058740	1.609851	1.210994
16	6	0	-1.884437	3.365924	0.308195
17	6	0	-1.661564	3.224716	1.668871
18	6	0	3.456319	-1.269373	1.049152
19	1	0	-2.246117	3.804542	2.372871
20	1	0	0.536563	-1.117536	1.085527
21	6	0	-1.970512	-1.213587	-0.613325
22	1	0	6.035771	0.851564	0.508916
23	8	0	1.920280	1.182567	-1.234314
24	1	0	5.486798	-1.230160	1.746908
25	6	0	4.735008	-0.740461	1.141320
26	6	0	-2.239318	-2.242491	0.282446
27	8	0	-1.442118	2.794122	-1.931748
28	1	0	3.195060	-2.183663	1.570411
29	1	0	-0.471268	2.262805	3.181809
30	1	0	-2.648931	0.650106	-1.465246
31	6	0	-0.669459	-1.444515	-1.355610
32	6	0	-1.136523	2.623589	-0.599087
33	6	0	-3.365099	-2.187415	1.097452
34	6	0	-3.961845	-0.076319	0.078022
35	1	0	-5.096908	-1.037904	1.628632
36	1	0	4.303152	1.959737	-0.888903
37	6	0	-2.839082	-0.134526	-0.739765
38	6	0	-4.218440	-1.094369	0.996406
39	6	0	4.084695	1.052780	-0.339496
40	1	0	-3.577448	-2.986926	1.798947
41	6	0	2.801639	0.515139	-0.433119
42	6	0	-1.212338	-3.343127	0.189952
43	1	0	-1.670689	-4.276477	-0.147324
44	6	0	-0.161867	-2.837201	-0.824945
45	1	0	-0.723115	-3.546672	1.145734
46	1	0	0.013120	-3.550316	-1.628404

Structure 47n (M06-2X, Ethanol)

Energy (Hartrees): =-1168.151446
No imaginary frequencies

Standard orientation:

Center Number	Atomic Number	Atomic Type	Coordinates (Angstroms) X	Y	Z
1	1	0	-0.809522	-1.435912	-2.449701
2	1	0	-0.757150	2.710765	-2.492683
3	8	0	1.135391	-2.584191	-0.249340
4	6	0	1.118478	-1.215949	0.119672
5	7	0	0.468823	-0.609443	-1.012618
6	6	0	-0.678175	2.318445	2.122982
7	6	0	2.493902	-0.637328	0.255851
8	1	0	-2.677997	4.013591	-0.036712
9	1	0	0.831902	0.933991	1.550748
10	1	0	-4.655039	0.730885	-0.020450
11	6	0	-0.204265	1.689498	-0.181459
12	1	0	0.149113	0.998554	-2.192941
13	6	0	0.529114	0.808832	-1.188646
14	6	0	5.039297	0.472199	0.454930
15	6	0	0.049069	1.590175	1.191778
16	6	0	-1.921430	3.330556	0.332311
17	6	0	-1.681820	3.180578	1.688984
18	6	0	3.465635	-1.241649	1.049747
19	1	0	-2.266091	3.747767	2.404404
20	1	0	0.546829	-1.126463	1.059488
21	6	0	-1.954339	-1.220554	-0.612166
22	1	0	6.028367	0.909714	0.529680
23	8	0	1.915007	1.201515	-1.236521
24	1	0	5.489882	-1.177359	1.767580
25	6	0	4.738863	-0.697835	1.151722
26	6	0	-2.206438	-2.238061	0.303470
27	8	0	-1.519208	2.805874	-1.911122
28	1	0	3.210885	-2.151625	1.583320
29	1	0	-0.463101	2.211814	3.179309
30	1	0	-2.665619	0.613405	-1.498203
31	6	0	-0.661073	-1.455610	-1.367397

32	6	0	-1.178200	2.606625	-0.598932
33	6	0	-3.327735	-2.181995	1.127086
34	6	0	-3.959392	-0.095627	0.072395
35	1	0	-5.071602	-1.043189	1.650305
36	1	0	4.296885	2.003349	-0.876837
37	6	0	-2.840205	-0.156186	-0.752996
38	6	0	-4.195876	-1.099986	1.013329
39	6	0	4.079732	1.091310	-0.332947
40	1	0	-3.525869	-2.972892	1.843101
41	6	0	2.802779	0.538822	-0.434661
42	6	0	-1.173732	-3.332338	0.216667
43	1	0	-1.631847	-4.274358	-0.094464
44	6	0	-0.149938	-2.842670	-0.829581
45	1	0	-0.673216	-3.512552	1.171669
46	1	0	0.006414	-3.566453	-1.626252

Structure 47o (M06-2X, Gas Phase)

Energy (Hartrees): =-1168.1386218
No imaginary frequencies

Standard orientation:

Center Number	Atomic Number	Atomic Type	Coordinates (Angstroms) X	Y	Z
1	6	0	3.088720	-2.116826	-1.215528
2	6	0	4.145788	-1.350293	-0.738583
3	6	0	4.009177	-0.593357	0.426148
4	6	0	2.805485	-0.582254	1.117780
5	6	0	1.738972	-1.319357	0.616155
6	6	0	0.617086	-2.871403	-0.837514
7	6	0	-5.093350	1.157541	-0.833647
8	6	0	1.880390	-2.103149	-0.526109
9	6	0	0.814640	1.296217	-0.297850
10	6	0	0.346199	-1.431635	1.171613
11	6	0	-4.305608	-0.846393	0.233087
12	6	0	-3.806666	1.670709	-0.849394
13	6	0	-0.264373	-2.647359	0.404654
14	6	0	-5.348567	-0.107382	-0.302368
15	6	0	1.161264	2.056072	0.828850
16	6	0	1.610444	1.357243	-1.435620
17	6	0	2.290793	2.871547	0.784544
18	6	0	3.074320	2.918801	-0.359218
19	6	0	-2.764413	0.908795	-0.325842
20	6	0	-1.874307	-1.096572	0.843857
21	6	0	-0.446258	0.477651	-0.303131
22	6	0	-3.008419	-0.338568	0.232358
23	6	0	2.746040	2.154773	-1.475685
24	1	0	3.207816	-2.722946	-2.106965
25	1	0	5.090844	-1.349999	-1.268934
26	1	0	0.347138	-1.592917	2.252244
27	1	0	-0.290696	-3.536394	1.041797
28	1	0	-2.129554	-1.410031	1.865113
29	1	0	-0.500915	-0.151442	-1.204180
30	1	0	2.687219	0.015713	2.014993
31	1	0	4.844103	-0.006149	0.788815
32	1	0	3.362490	2.183401	-2.365055
33	1	0	3.953370	3.552850	-0.374916
34	1	0	1.337468	0.754521	-2.296122
35	1	0	2.538189	3.447921	1.667715
36	1	0	-6.355177	-0.505374	-0.301886
37	1	0	-5.905504	1.744763	-1.245209
38	1	0	-4.484272	-1.827423	0.660125
39	1	0	0.102733	-2.474887	-1.718279
40	1	0	0.816795	-3.928522	-1.022007
41	1	0	-0.189107	1.288413	1.921108
42	1	0	-3.580811	2.645004	-1.263785
43	7	0	-0.629441	-0.329285	0.922275
44	8	0	0.470409	2.000506	1.989266
45	8	0	-1.582265	-2.253513	0.064171
46	8	0	-1.506916	1.426717	-0.359917

Structure 47o (M06-2X, Ethanol)

Energy (Hartrees): =-1168.1704922
No imaginary frequencies

Standard orientation:

Center Number	Atomic Number	Atomic Type	Coordinates (Angstroms) X	Y	Z
1	6	0	3.122916	-2.075194	-1.221217

2	6	0	4.178123	-1.331246	-0.700637
3	6	0	4.028356	-0.614360	0.489476
4	6	0	2.809513	-0.614102	1.158527
5	6	0	1.747314	-1.328318	0.614953
6	6	0	0.640561	-2.828621	-0.903922
7	6	0	-5.107688	1.149900	-0.802806
8	6	0	1.903310	-2.078429	-0.549818
9	6	0	0.807468	1.295238	-0.300193
10	6	0	0.348161	-1.458173	1.148538
11	6	0	-4.310617	-0.854354	0.262599
12	6	0	-3.818674	1.661603	-0.833492
13	6	0	-0.247453	-2.650061	0.340406
14	6	0	-5.358954	-0.112966	-0.263519
15	6	0	1.159738	2.040331	0.834018
16	6	0	1.584080	1.394122	-1.449950
17	6	0	2.273844	2.876063	0.794263
18	6	0	3.040068	2.961034	-0.361371
19	6	0	-2.773778	0.898119	-0.317942
20	6	0	-1.869520	-1.115826	0.831247
21	6	0	-0.445773	0.462615	-0.307669
22	6	0	-3.011236	-0.349821	0.243259
23	6	0	2.705283	2.215475	-1.489494
24	1	0	3.253650	-2.650432	-2.131850
25	1	0	5.132830	-1.321399	-1.214797
26	1	0	0.331539	-1.648626	2.222909
27	1	0	-0.273181	-3.557054	0.948999
28	1	0	-2.114829	-1.460298	1.842337
29	1	0	-0.498878	-0.153207	-1.215479
30	1	0	2.682266	-0.055429	2.080202
31	1	0	4.866648	-0.056995	0.892189
32	1	0	3.306359	2.275450	-2.388665
33	1	0	3.909721	3.608546	-0.375711
34	1	0	1.303662	0.808552	-2.320256
35	1	0	2.532061	3.438518	1.684549
36	1	0	-6.367479	-0.507595	-0.247015
37	1	0	-5.923267	1.738183	-1.207400
38	1	0	-4.489587	-1.830337	0.702546
39	1	0	0.147106	-2.389470	-1.776928
40	1	0	0.838642	-3.878355	-1.126753
41	1	0	-0.147019	1.197590	1.940952
42	1	0	-3.601854	2.636648	-1.253915
43	7	0	-0.625571	-0.346153	0.915443
44	8	0	0.469488	1.954773	2.001011
45	8	0	-1.574915	-2.250490	0.011695
46	8	0	-1.512030	1.417043	-0.357149

Structure 47p (M06-2X, Gas Phase)

Energy (Hartrees): =-1168.1357705
No imaginary frequencies

Standard orientation:

Center Number	Atomic Number	Atomic Type	Coordinates (Angstroms) X	Y	Z
1	6	0	3.278429	-3.089601	-0.463950
2	6	0	3.926167	-2.404456	-1.486883
3	6	0	3.655756	-1.056784	-1.716068
4	6	0	2.721406	-0.381625	-0.936525
5	6	0	0.207686	2.690090	1.043114
6	6	0	1.585677	-2.930748	1.511823
7	6	0	2.054959	-1.075099	0.069258
8	6	0	2.346201	-2.415602	0.315336
9	6	0	0.994200	-0.561812	1.028359
10	6	0	0.529499	-1.851012	1.782731
11	6	0	-4.001606	0.500027	-0.987589
12	6	0	-3.354858	-2.072820	-0.143235
13	6	0	1.028475	2.843252	-1.220838
14	6	0	0.327745	2.140784	-0.243261
15	6	0	-0.924774	-1.382368	0.074766
16	6	0	-0.378100	0.861596	-0.599012
17	6	0	0.822645	3.910349	1.327118
18	6	0	-2.358747	-1.108610	-0.247034
19	6	0	-2.684300	0.181267	-0.667914
20	6	0	-4.669499	-1.761177	-0.460414
21	6	0	1.529684	4.584261	0.344534
22	6	0	1.637416	4.057490	-0.940554
23	6	0	-4.985424	-0.472080	-0.884463
24	1	0	4.180343	-0.529634	-2.504087
25	1	0	2.531727	0.672656	-1.100176
26	1	0	1.090060	2.421835	-2.219647
27	1	0	-3.084330	-3.062575	0.205768
28	1	0	-4.226388	1.511142	-1.302650
29	1	0	-6.009302	-0.219126	-1.133155
30	1	0	2.181095	4.588642	-1.711014

31	1	0	1.999631	5.531128	0.582939
32	1	0	-5.443581	-2.512822	-0.371908
33	1	0	0.721649	4.308049	2.329243
34	1	0	-0.411728	-1.831765	-0.796070
35	1	0	0.353888	-1.679274	2.844215
36	1	0	1.376129	0.213070	1.697028
37	1	0	-0.009554	0.473768	-1.562812
38	1	0	4.653784	-2.918899	-2.103291
39	1	0	3.501548	-4.133852	-0.274460
40	1	0	1.102670	-3.894586	1.342238
41	1	0	2.258355	-3.037922	2.368334
42	1	0	-0.798972	1.226284	1.725970
43	7	0	-0.282646	-0.133713	0.450108
44	8	0	-0.468965	2.086383	2.042823
45	8	0	-1.763843	1.188017	-0.761511
46	8	0	-0.732234	-2.202155	1.198311

Structure 47p (M06-2X, Ethanol)

Energy (Hartrees): =-1168.1655723
No imaginary frequencies

Standard orientation:

Center Number	Atomic Number	Atomic Type	Coordinates (Angstroms) X	Y	Z
1	6	0	3.774882	-2.566511	-0.373673
2	6	0	4.300445	-1.839361	-1.438555
3	6	0	3.805955	-0.571166	-1.741779
4	6	0	2.770844	-0.019061	-0.990641
5	6	0	-0.343717	2.706516	1.008582
6	6	0	2.062504	-2.591189	1.593515
7	6	0	2.231505	-0.755367	0.060488
8	6	0	2.740647	-2.015706	0.375116
9	6	0	1.103613	-0.376136	1.006924
10	6	0	0.842407	-1.691672	1.803228
11	6	0	-3.999966	-0.155753	-1.017631
12	6	0	-2.949841	-2.577743	-0.128480
13	6	0	0.606934	3.003090	-1.189281
14	6	0	-0.026302	2.185826	-0.255974
15	6	0	-0.656454	-1.503641	0.060019
16	6	0	-0.469198	0.798060	-0.634769
17	6	0	-0.008547	4.024286	1.317784
18	6	0	-2.117695	-1.470419	-0.257392
19	6	0	-2.647267	-0.257291	-0.700708
20	6	0	-4.300731	-2.483267	-0.439490
21	6	0	0.629787	4.820521	0.376707
22	6	0	0.944531	4.315958	-0.882656
23	6	0	-4.819383	-1.268697	-0.886000
24	1	0	4.231757	-0.010091	-2.565824
25	1	0	2.402587	0.973794	-1.222993
26	1	0	0.830228	2.595783	-2.170672
27	1	0	-2.529189	-3.512001	0.227592
28	1	0	-4.387324	0.798181	-1.356152
29	1	0	-5.871597	-1.185470	-1.132872
30	1	0	1.439353	4.938701	-1.617592
31	1	0	0.884339	5.843324	0.631382
32	1	0	-4.945633	-3.347116	-0.333979
33	1	0	-0.260318	4.403396	2.301625
34	1	0	-0.081486	-1.878823	-0.803700
35	1	0	0.613120	-1.512597	2.852394
36	1	0	1.368677	0.464793	1.651203
37	1	0	-0.026569	0.490217	-1.592521
38	1	0	5.104120	-2.259122	-2.033164
39	1	0	4.167468	-3.548333	-0.130698
40	1	0	1.761018	-3.633381	1.472235
41	1	0	2.728674	-2.536222	2.459534
42	1	0	-1.020089	1.044773	1.667412
43	7	0	-0.225836	-0.162916	0.422360
44	8	0	-0.973526	1.976468	1.963033
45	8	0	-1.896806	0.881070	-0.821613
46	8	0	-0.339417	-2.264668	1.202114

Structure 50a (M06-2X, Gas Phase)

Energy (Hartrees): = =-823.815571
No imaginary frequencies

Standard orientation:

Center Number	Atomic Number	Atomic Type	Coordinates (Angstroms) X	Y	Z

1	6	0	0.247793	2.189781	-1.466568
2	6	0	-0.316633	3.078731	-0.556905
3	6	0	-0.079267	2.945605	0.810596
4	6	0	0.720441	1.913206	1.286750
5	6	0	1.271045	1.021928	0.375201
6	6	0	1.047667	1.159034	-0.990247
7	6	0	2.081248	-0.214154	0.682279
8	6	0	2.255240	-0.917693	-0.705524
9	6	0	1.737200	0.069516	-1.772581
10	6	0	-1.500788	-0.616992	1.024011
11	6	0	0.605800	-1.986358	0.509430
12	6	0	-1.207417	-1.558852	-1.174181
13	6	0	-0.721703	-1.352640	0.116471
14	6	0	-2.721792	-0.081774	0.609440
15	6	0	-2.423352	-1.031758	-1.583800
16	6	0	-3.178578	-0.286451	-0.681669
17	1	0	0.884136	1.787413	2.351219
18	1	0	3.027336	0.021216	1.172234
19	1	0	3.281170	-1.229550	-0.902218
20	1	0	2.571921	0.477444	-2.350222
21	1	0	1.065941	-0.434749	-2.469833
22	1	0	2.002649	-1.805759	1.960535
23	1	0	0.439839	-2.991885	0.903989
24	1	0	0.050853	2.290712	-2.528009
25	1	0	-0.953784	3.879547	-0.913218
26	1	0	-0.530153	3.643366	1.505903
27	1	0	-4.127731	0.138752	-0.986532
28	1	0	-3.286247	0.498474	1.328844
29	1	0	-2.779555	-1.200820	-2.592180
30	1	0	-0.602410	-2.142076	-1.858656
31	1	0	-0.174516	-0.605638	2.368350
32	7	0	1.337777	-1.191168	1.501280
33	8	0	-1.124037	-0.385420	2.301964
34	8	0	1.482123	-2.111463	-0.601388

Structure 50a (M06-2X, EtOH)

Energy (Hartrees): =-823.8424937
No imaginary frequencies

Standard orientation:

Center Number	Atomic Number	Atomic Type	Coordinates (Angstroms) X	Y	Z
1	6	0	0.285052	2.206032	-1.445094
2	6	0	-0.281413	3.090519	-0.529306
3	6	0	-0.054935	2.940417	0.840047
4	6	0	0.730617	1.892213	1.311760
5	6	0	1.277868	1.002084	0.395072
6	6	0	1.072170	1.161022	-0.972777
7	6	0	2.070212	-0.248760	0.693861
8	6	0	2.257506	-0.927000	-0.700778
9	6	0	1.770508	0.080873	-1.760321
10	6	0	-1.512973	-0.628210	1.011347
11	6	0	0.579846	-2.007457	0.482557
12	6	0	-1.232990	-1.525802	-1.203651
13	6	0	-0.743760	-1.359408	0.091318
14	6	0	-2.723910	-0.059362	0.613049
15	6	0	-2.441621	-0.966655	-1.601491
16	6	0	-3.185592	-0.227500	-0.684853
17	1	0	0.893818	1.759044	2.376276
18	1	0	3.011531	-0.035098	1.199615
19	1	0	3.281054	-1.251721	-0.882496
20	1	0	2.623838	0.495609	-2.304532
21	1	0	1.116484	-0.399765	-2.491016
22	1	0	1.958909	-1.853922	1.950651
23	1	0	0.405245	-3.016599	0.861596
24	1	0	0.104761	2.326115	-2.508400
25	1	0	-0.905090	3.904300	-0.882501
26	1	0	-0.498618	3.640755	1.538866
27	1	0	-4.126473	0.222924	-0.980734
28	1	0	-3.283532	0.516596	1.341752
29	1	0	-2.798349	-1.104652	-2.615159
30	1	0	-0.641316	-2.099029	-1.908216
31	1	0	-0.162972	-0.667681	2.341393
32	7	0	1.304326	-1.229293	1.487072
33	8	0	-1.120309	-0.432373	2.296216
34	8	0	1.456672	-2.113860	-0.632597

Structure 50b (M06-2X, Gas Phase)

Energy (Hartrees): =-823.8131456
No imaginary frequencies

Standard orientation:

Center Number	Atomic Number	Atomic Type	Coordinates (Angstroms) X	Y	Z
1	6	0	-1.884044	0.855820	1.637423
2	6	0	-2.755467	1.614202	0.862770
3	6	0	-3.099949	1.206340	-0.425970
4	6	0	-2.561715	0.040919	-0.959351
5	6	0	-1.686909	-0.707891	-0.184033
6	6	0	-1.357980	-0.316422	1.107296
7	6	0	-0.932839	-1.945642	-0.602869
8	6	0	-0.036653	-2.290908	0.636611
9	6	0	-0.400649	-1.287747	1.754061
10	6	0	1.331659	0.144334	-0.707607
11	6	0	1.247979	-1.337648	-1.044739
12	6	0	0.648677	1.082355	-1.480572
13	6	0	2.133517	0.598673	0.350085
14	6	0	2.221131	1.964327	0.620230
15	6	0	1.526415	2.878196	-0.154806
16	6	0	0.733475	2.440198	-1.212026
17	1	0	-0.135639	-3.325029	0.964275
18	1	0	-0.879925	-1.804660	2.590076
19	1	0	0.495657	-0.798245	2.143131
20	1	0	0.166680	-2.568706	-2.204986
21	1	0	-1.610811	1.180565	2.635232
22	1	0	-3.168153	2.533106	1.262419
23	1	0	-3.780445	1.808249	-1.016488
24	1	0	-2.797954	-0.267447	-1.972278
25	1	0	-1.605517	-2.758028	-0.882742
26	1	0	1.600621	3.935607	0.070658
27	1	0	2.842298	2.275312	1.451210
28	1	0	0.182055	3.148543	-1.817164
29	1	0	0.027139	0.718041	-2.289097
30	1	0	2.106988	-1.637543	-1.649530
31	1	0	2.539675	-1.137947	1.007054
32	7	0	0.013167	-1.700596	-1.704088
33	8	0	1.299196	-2.148094	0.150726
34	8	0	2.846465	-0.230941	1.156502

Structure 50b (M06-2X, EtOH)

Energy (Hartrees): =-823.839044
No imaginary frequencies

Standard orientation:

Center Number	Atomic Number	Atomic Type	Coordinates (Angstroms) X	Y	Z
1	6	0	-1.855087	0.906786	1.657747
2	6	0	-2.602137	1.790880	0.882705
3	6	0	-2.914098	1.484595	-0.443488
4	6	0	-2.467022	0.296052	-1.013444
5	6	0	-1.710552	-0.576204	-0.240208
6	6	0	-1.417102	-0.285266	1.088383
7	6	0	-1.058709	-1.863984	-0.686724
8	6	0	-0.243031	-2.331225	0.562121
9	6	0	-0.602216	-1.384691	1.724228
10	6	0	1.357663	0.068812	-0.688788
11	6	0	1.173567	-1.402336	-1.046386
12	6	0	0.813324	1.065273	-1.498858
13	6	0	2.116593	0.459352	0.425767
14	6	0	2.292924	1.812366	0.717780
15	6	0	1.730290	2.784974	-0.095185
16	6	0	0.986421	2.413705	-1.212772
17	1	0	-0.406318	-3.377470	0.814335
18	1	0	-1.193195	-1.916117	2.475275
19	1	0	0.296190	-1.012099	2.222840
20	1	0	0.053739	-2.546368	-2.247747
21	1	0	-1.613351	1.148134	2.687964
22	1	0	-2.946214	2.725428	1.312057
23	1	0	-3.500434	2.180636	-1.032845
24	1	0	-2.686640	0.064322	-2.051038
25	1	0	-1.791121	-2.600698	-1.017136
26	1	0	1.871939	3.832487	0.145926
27	1	0	2.875228	2.075465	1.593823
28	1	0	0.543152	3.165980	-1.854375
29	1	0	0.229790	0.763319	-2.360120
30	1	0	2.027389	-1.755054	-1.628253
31	1	0	2.339328	-1.312117	1.092440
32	7	0	-0.059768	-1.667384	-1.751091
33	8	0	1.126175	-2.219384	0.144762
34	8	0	2.713065	-0.430452	1.268641

Structure 51a (M06-2X, Gas Phase)

Energy (Hartrees): =-823.8131283
No imaginary frequencies

Standard orientation:

Center Number	Atomic Number	Atomic Type	Coordinates (Angstroms) X	Y	Z
1	6	0	3.806296	-0.312722	-1.210918
2	6	0	4.267400	0.998227	-1.150749
3	6	0	3.726699	1.901302	-0.235089
4	6	0	2.717430	1.500529	0.633115
5	6	0	2.266934	0.187752	0.574589
6	6	0	2.799425	-0.714523	-0.340034
7	6	0	1.174561	-0.441064	1.405426
8	6	0	0.962172	-1.844665	0.740853
9	6	0	2.148591	-2.071246	-0.214100
10	6	0	-2.612014	1.159207	-0.066991
11	6	0	-0.628329	-0.348325	0.008008
12	6	0	-2.974059	-1.207257	-0.388062
13	6	0	-2.108439	-0.147524	-0.140424
14	6	0	-3.971479	1.386500	-0.265232
15	6	0	-4.331791	-0.980226	-0.577480
16	6	0	-4.822542	0.320787	-0.522800
17	1	0	-0.738624	-0.040886	1.995275
18	1	0	-0.984512	1.917351	0.580460
19	1	0	4.222061	-1.008443	-1.931602
20	1	0	5.049851	1.324726	-1.825548
21	1	0	4.092307	2.920640	-0.205576
22	1	0	2.280728	2.199243	1.338417
23	1	0	1.439277	-0.492577	2.462682
24	1	0	0.848655	-2.652751	1.463904
25	1	0	2.850159	-2.798787	0.204859
26	1	0	1.791691	-2.464605	-1.168045
27	1	0	-0.097207	0.135830	-0.824211
28	1	0	-2.561286	-2.206875	-0.438915
29	1	0	-4.334403	2.404994	-0.206336
30	1	0	-4.999316	-1.809703	-0.773472
31	1	0	-5.878733	0.509348	-0.675527
32	7	0	-0.113366	0.243184	1.242406
33	8	0	-0.277321	-1.722667	0.048996
34	8	0	-1.811706	2.227629	0.176770

Structure 51a (M06-2X, EtOH)

Energy (Hartrees): =-823.8377082
No imaginary frequencies

Standard orientation:

Center Number	Atomic Number	Atomic Type	Coordinates (Angstroms) X	Y	Z
1	6	0	3.827727	-0.340274	-1.199970
2	6	0	4.300705	0.968888	-1.155496
3	6	0	3.760474	1.890677	-0.256737
4	6	0	2.738857	1.511382	0.609517
5	6	0	2.272970	0.202126	0.564072
6	6	0	2.808017	-0.719668	-0.332140
7	6	0	1.174200	-0.410479	1.399655
8	6	0	0.966422	-1.824695	0.764592
9	6	0	2.149932	-2.070516	-0.186268
10	6	0	-2.632727	1.152535	-0.066230
11	6	0	-0.635534	-0.342971	0.008365
12	6	0	-2.983193	-1.210186	-0.396191
13	6	0	-2.118608	-0.150284	-0.142080
14	6	0	-3.992541	1.378148	-0.262945
15	6	0	-4.343679	-0.988076	-0.586953
16	6	0	-4.841989	0.309953	-0.526342
17	1	0	-0.729259	-0.008682	1.994813
18	1	0	-0.996923	1.917147	0.577268
19	1	0	4.246185	-1.051654	-1.904524
20	1	0	5.094293	1.277413	-1.826879
21	1	0	4.137761	2.906768	-0.235882
22	1	0	2.311880	2.225409	1.306499
23	1	0	1.432178	-0.446512	2.458126
24	1	0	0.852300	-2.614743	1.505228
25	1	0	2.846197	-2.793086	0.249023
26	1	0	1.803371	-2.479266	-1.138146
27	1	0	-0.115232	0.123917	-0.839789
28	1	0	-2.577424	-2.212914	-0.452888
29	1	0	-4.364915	2.394287	-0.200394
30	1	0	-5.006440	-1.821189	-0.787551
31	1	0	-5.899147	0.495911	-0.678642

32	7	0	-0.117583	0.268619	1.226824
33	8	0	-0.282175	-1.718539	0.067059
34	8	0	-1.832568	2.226977	0.180050

Structure 51b (M06-2X, Gas Phase)

Energy (Hartrees): =-823.8108482
No imaginary frequencies

Standard orientation:

Center Number	Atomic Number	Atomic Type	Coordinates (Angstroms) X	Y	Z
1	6	0	2.065659	-0.264920	-0.047834
2	6	0	0.580353	-0.079670	0.126678
3	6	0	2.645847	-1.502054	0.236148
4	6	0	2.872154	0.760510	-0.565752
5	6	0	-0.915848	1.300735	1.292772
6	6	0	-1.233607	-0.215698	1.490464
7	6	0	3.999128	-1.729770	0.029149
8	6	0	4.231863	0.528370	-0.774857
9	6	0	-2.831796	-1.702460	0.079902
10	6	0	-3.812135	0.684847	-1.026471
11	6	0	-2.101303	1.909996	0.528376
12	6	0	-2.333848	-0.471633	0.486135
13	6	0	4.790146	-0.704959	-0.481030
14	6	0	-4.323718	-0.544860	-1.428593
15	6	0	-3.836141	-1.731840	-0.881460
16	6	0	-2.810163	0.714056	-0.063381
17	1	0	0.050670	-0.271439	-0.814491
18	1	0	2.010614	-2.297596	0.608551
19	1	0	0.629413	-0.903088	1.955553
20	1	0	-5.102477	-0.583373	-2.181176
21	1	0	-4.184513	1.604397	-1.464546
22	1	0	-2.426926	-2.618445	0.495341
23	1	0	-4.237534	-2.681158	-1.215597
24	1	0	5.848249	-0.866093	-0.650692
25	1	0	4.826180	1.342837	-1.169907
26	1	0	4.428669	-2.697239	0.255566
27	1	0	-1.540130	-0.467079	2.507295
28	1	0	-0.696177	1.826216	2.222966
29	1	0	-2.760485	2.449412	1.215814
30	1	0	-1.754188	2.620542	-0.224248
31	1	0	1.529186	2.104734	-0.495955
32	7	0	0.004028	-0.916526	1.152186
33	8	0	0.279822	1.286121	0.514262
34	8	0	2.399141	1.982524	-0.903168

Structure 51b (M06-2X, EtOH)

Energy (Hartrees): =-823.8357652
No imaginary frequencies

Standard orientation:

Center Number	Atomic Number	Atomic Type	Coordinates (Angstroms) X	Y	Z
1	6	0	2.083274	-0.271952	-0.037543
2	6	0	0.600171	-0.106771	0.163056
3	6	0	2.727853	-1.473457	0.253740
4	6	0	2.829167	0.777829	-0.594948
5	6	0	-0.910514	1.146018	1.434449
6	6	0	-1.242996	-0.379708	1.464788
7	6	0	4.086295	-1.639743	0.006627
8	6	0	4.189795	0.612809	-0.845597
9	6	0	-2.884534	-1.699185	-0.064758
10	6	0	-3.790695	0.800225	-0.971317
11	6	0	-2.052879	1.844031	0.681143
12	6	0	-2.343934	-0.521399	0.438445
13	6	0	4.814062	-0.591179	-0.547580
14	6	0	-4.339879	-0.377222	-1.473646
15	6	0	-3.890095	-1.620240	-1.024735
16	6	0	-2.786558	0.720294	-0.011239
17	1	0	0.082540	-0.159361	-0.803220
18	1	0	2.152094	-2.293732	0.667339
19	1	0	0.594706	-1.136199	1.885030
20	1	0	-5.121038	-0.327957	-2.224263
21	1	0	-4.137939	1.764091	-1.329043
22	1	0	-2.524111	-2.662515	0.281140

23	1	0	-4.323754	-2.527870	-1.429202
24	1	0	5.873092	-0.707609	-0.748198
25	1	0	4.738442	1.443019	-1.275840
26	1	0	4.568894	-2.581205	0.239068
27	1	0	-1.552262	-0.731527	2.449222
28	1	0	-0.736692	1.570152	2.422527
29	1	0	-2.713178	2.355756	1.387618
30	1	0	-1.667846	2.594827	-0.012531
31	1	0	1.421148	2.071949	-0.478482
32	7	0	-0.003998	-1.055823	1.063751
33	8	0	0.333344	1.202759	0.724034
34	8	0	2.271993	1.971845	-0.936875

Structure 52 (M06-2X, Gas Phase)

Energy (Hartrees): =-1290.8795295
No imaginary frequencies

Standard orientation:

Center Number	Atomic Number	Atomic Type	Coordinates (Angstroms) X	Y	Z
1	6	0	-3.008657	-1.339654	0.148373
2	6	0	3.321519	-1.435282	-0.761588
3	6	0	-1.320935	0.870532	-0.446312
4	6	0	-3.979633	-2.474452	0.477003
5	6	0	2.833754	-0.069768	-1.188421
6	6	0	-3.282958	-3.838035	0.469531
7	6	0	4.233890	-3.737875	0.466320
8	6	0	-5.108220	-2.503919	-0.566834
9	7	0	1.405442	0.172267	-1.016124
10	6	0	-4.033619	2.704085	1.199404
11	6	0	-4.581409	-2.246593	1.873623
12	6	0	-1.663555	-1.530086	-0.111950
13	6	0	-0.822888	-0.453987	-0.412327
14	6	0	3.771418	-1.389227	0.560158
15	6	0	3.678593	0.017887	1.099664
16	6	0	3.782204	-3.781980	-0.850655
17	6	0	3.607144	0.834880	-0.199916
18	6	0	-4.497910	2.449647	-1.257238
19	6	0	-2.494025	3.676539	-0.476302
20	6	0	-2.688470	1.098377	-0.178685
21	6	0	-3.470717	-0.018931	0.103639
22	6	0	3.321600	-2.624972	-1.475655
23	6	0	0.590692	-0.736872	-0.653071
24	6	0	4.235136	-2.540981	1.179899
25	6	0	-3.395746	2.468222	-0.182479
26	1	0	-1.224205	-2.521177	-0.090860
27	1	0	0.909495	-1.773744	-0.493588
28	1	0	-4.523077	0.149903	0.303913
29	1	0	2.972186	-2.653579	-2.502369
30	1	0	4.598696	-2.510687	2.200903
31	1	0	0.404887	1.511642	-0.905848
32	1	0	-4.062635	2.285965	-2.246086
33	1	0	4.596034	-4.643922	0.937677
34	1	0	-3.115301	4.576588	-0.448340
35	1	0	-4.549194	3.668510	1.209749
36	1	0	-3.265748	2.717539	1.976785
37	1	0	-5.236484	1.664982	-1.079283
38	1	0	3.080584	0.179216	-2.223440
39	8	0	-0.497224	1.888810	-0.723077
40	1	0	-2.027755	3.606994	-1.458045
41	1	0	3.796359	-4.719652	-1.393031
42	1	0	-1.701059	3.780924	0.263895
43	1	0	2.752308	0.159952	1.667878
44	8	0	2.942771	2.086377	-0.081741
45	1	0	-4.762337	1.933160	1.457798
46	1	0	4.620714	0.999388	-0.575133
47	1	0	-2.852846	-4.061466	-0.510350
48	1	0	-5.806272	-3.316526	-0.346119
49	1	0	-4.700827	-2.659829	-1.568726
50	1	0	-5.673748	-1.569420	-0.575708
51	1	0	-5.276493	-3.053728	2.122457
52	1	0	-5.129471	-1.303126	1.926426
53	1	0	-3.794405	-2.221548	2.631345
54	1	0	-5.022875	3.409095	-1.268160
55	1	0	-2.487461	-3.886196	1.217763
56	1	0	-4.008577	-4.621016	0.702401
57	1	0	4.519318	0.311888	1.726188
58	1	0	2.826470	4.767976	-0.379203
59	8	0	4.665823	2.884821	1.109830
60	6	0	3.591260	3.048805	0.602753
61	1	0	1.746940	4.100484	0.841378

62	1	0	3.214882	5.007277	1.342615
63	6	0	2.790344	4.320004	0.615715

Structure 52 (M06-2X, Pyridine)

Energy (Hartrees): =-1290.9088113
No imaginary frequencies

Standard orientation:

Center Number	Atomic Number	Atomic Type	Coordinates (Angstroms) X	Y	Z
1	6	0	-3.065001	-1.299487	0.181577
2	6	0	3.285541	-1.444098	-0.758402
3	6	0	-1.355521	0.896923	-0.405955
4	6	0	-3.977215	-2.491782	0.483255
5	6	0	2.795520	-0.071880	-1.160799
6	6	0	-3.957756	-3.460479	-0.710042
7	6	0	4.250994	-3.759037	0.407502
8	6	0	-5.425487	-2.063225	0.732606
9	7	0	1.373264	0.180071	-0.943002
10	6	0	-4.138953	2.746896	1.142226
11	6	0	-3.468045	-3.225233	1.734233
12	6	0	-1.709697	-1.498075	-0.059822
13	6	0	-0.861263	-0.433978	-0.355119
14	6	0	3.809057	-1.406785	0.536674
15	6	0	3.758597	-0.001681	1.085516
16	6	0	3.723759	-3.794388	-0.882720
17	6	0	3.619003	0.822550	-0.203199
18	6	0	-4.472957	2.478938	-1.329550
19	6	0	-2.511876	3.709402	-0.451257
20	6	0	-2.720641	1.132101	-0.160136
21	6	0	-3.522268	0.017285	0.122307
22	6	0	3.235878	-2.631294	-1.476921
23	6	0	0.553849	-0.730462	-0.596326
24	6	0	4.301194	-2.564568	1.125575
25	6	0	-3.426934	2.502743	-0.201053
26	1	0	-1.281704	-2.495985	-0.026862
27	1	0	0.856314	-1.774100	-0.460809
28	1	0	-4.573689	0.200483	0.302314
29	1	0	2.829010	-2.653623	-2.482589
30	1	0	4.721822	-2.540926	2.125085
31	1	0	0.389261	1.504793	-0.838373
32	1	0	-3.992800	2.309340	-2.297583
33	1	0	4.633953	-4.669830	0.853919
34	1	0	-3.130300	4.612081	-0.438632
35	1	0	-4.649036	3.714558	1.118305
36	1	0	-3.417370	2.763214	1.963833
37	1	0	-5.220535	1.695791	-1.180922
38	1	0	3.007347	0.174734	-2.203777
39	8	0	-0.513425	1.903231	-0.685037
40	1	0	-2.011876	3.650121	-1.417478
41	1	0	3.699902	-4.730432	-1.428679
42	1	0	-1.745714	3.809171	0.318499
43	1	0	2.870427	0.142625	1.711183
44	8	0	2.963528	2.076321	-0.029926
45	1	0	-4.888143	1.983367	1.361747
46	1	0	4.606642	0.989563	-0.638243
47	1	0	-4.314227	-2.965627	-1.617949
48	1	0	-6.031317	-2.948129	0.944642
49	1	0	-5.856955	-1.565006	-0.140137
50	1	0	-5.506169	-1.389678	1.590663
51	1	0	-4.114558	-4.078853	1.959846
52	1	0	-3.466596	-2.560246	2.602480
53	1	0	-2.452118	-3.602786	1.594091
54	1	0	-4.996728	3.438843	-1.374025
55	1	0	-2.950610	-3.838996	-0.902023
56	1	0	-4.607505	-4.317834	-0.509376
57	1	0	4.638594	0.272871	1.666053
58	1	0	2.530402	4.639019	-0.280821
59	8	0	4.804514	2.912735	0.937434
60	6	0	3.668175	3.052622	0.569680
61	1	0	1.945429	4.075398	1.283123
62	1	0	3.427861	5.070852	1.202688
63	6	0	2.845580	4.298065	0.706992

Structure 53 (M06-2X, Gas Phase)

Energy (Hartrees): =-1290.8905138
No imaginary frequencies

Standard orientation:

Center Number	Atomic Number	Atomic Type	Coordinates (Angstroms) X	Y	Z
1	6	0	3.490030	1.447915	1.941612
2	6	0	3.998201	2.264633	0.933050
3	6	0	-0.574373	-0.624092	-0.593163
4	1	0	-3.255615	1.955990	0.274108
5	6	0	-4.789335	-0.024234	1.529011
6	6	0	4.069729	1.809085	-0.381000
7	1	0	-4.946299	0.196075	-1.930589
8	6	0	-0.223719	0.705630	-0.426595
9	1	0	-6.278544	0.259018	-0.763896
10	6	0	3.049390	0.160289	1.649462
11	6	0	-1.902028	-1.049080	-0.404998
12	6	0	3.146744	-0.297830	0.343256
13	6	0	-1.179232	1.664619	-0.100185
14	6	0	3.645889	0.518028	-0.669524
15	6	0	-2.492655	1.227348	0.034416
16	1	0	-1.527837	-2.885668	-0.065301
17	6	0	-2.898673	-0.106516	-0.108982
18	6	0	3.620295	-0.194397	-2.002725
19	1	0	3.222987	0.420018	-2.812497
20	1	0	4.621112	-0.532149	-2.287848
21	6	0	2.746255	-1.648595	-0.217758
22	1	0	-5.044009	1.520687	-0.763875
23	1	0	3.455316	-2.433042	0.055121
24	6	0	-4.397754	-0.404378	0.089501
25	6	0	2.701322	-1.387167	-1.737077
26	7	0	1.382808	-2.068627	0.047903
27	8	0	1.360451	-0.975179	-1.949020
28	1	0	2.931784	-2.276031	-2.332395
29	1	0	4.322354	3.270632	1.171747
30	1	0	4.444353	2.456277	-1.166233
31	6	0	-5.210678	0.450783	-0.901307
32	6	0	0.484898	-1.605963	-1.038627
33	1	0	-0.221374	2.863745	2.177435
34	6	0	-1.942715	4.041085	0.381988
35	1	0	0.021424	-2.488716	-1.491612
36	1	0	1.122724	2.594170	1.059867
37	1	0	-4.605253	1.031604	1.738716
38	1	0	2.615775	-0.459044	2.427002
39	1	0	3.416763	1.826690	2.953934
40	1	0	-4.218222	-0.617254	2.247459
41	1	0	-5.853288	-0.220717	1.689500
42	6	0	0.241342	3.210333	1.249496
43	1	0	0.572425	4.244032	1.391228
44	1	0	-2.673899	4.027825	-0.430716
45	1	0	-2.448013	3.755810	1.308400
46	1	0	-1.588949	5.068483	0.498030
47	1	0	-0.746824	3.548690	-2.054484
48	1	0	0.227892	4.670253	-1.084569
49	1	0	0.828336	3.043453	-1.428076
50	6	0	-0.069191	3.623659	-1.200477
51	6	0	-0.754563	3.123112	0.082110
52	6	0	-4.817694	-1.864978	-0.131694
53	1	0	-4.561047	-2.214763	-1.131345
54	1	0	-4.348463	-2.537412	0.584791
55	1	0	-5.903262	-1.927296	-0.008340
56	1	0	0.811522	0.988445	-0.585485
57	8	0	-2.201575	-2.369402	-0.548538
58	6	0	1.989010	-3.568807	1.888331
59	1	0	2.583525	-4.337204	1.386653
60	1	0	2.667934	-2.806337	2.268914
61	1	0	1.447822	-4.032961	2.709067
62	8	0	-0.181630	-3.432749	0.911135
63	6	0	0.971042	-3.016194	0.917105

Structure 53 (M06-2X, Pyridine)

Energy (Hartrees): =-1290.9235344
No imaginary frequencies

Standard orientation:

Center Number	Atomic Number	Atomic Type	Coordinates (Angstroms) X	Y	Z
1	6	0	3.452128	1.400613	2.034733
2	6	0	3.975096	2.248770	1.058579
3	6	0	-0.568272	-0.613604	-0.628348
4	1	0	-3.265745	1.937741	0.302329
5	6	0	-4.752426	-0.044385	1.582800
6	6	0	4.066576	1.834750	-0.269322
7	1	0	-4.982788	0.154650	-1.873667
8	6	0	-0.233955	0.722683	-0.474044
9	1	0	-6.288759	0.202281	-0.677766

10	6	0	3.019182	0.119713	1.696989
11	6	0	-1.889339	-1.053751	-0.413240
12	6	0	3.135198	-0.295864	0.377126
13	6	0	-1.193976	1.672060	-0.125256
14	6	0	3.646454	0.552256	-0.604546
15	6	0	-2.499080	1.219238	0.043062
16	1	0	-1.452925	-2.882454	-0.115330
17	6	0	-2.890477	-0.121981	-0.092010
18	6	0	3.647069	-0.124966	-1.956278
19	1	0	3.274324	0.513063	-2.759805
20	1	0	4.653485	-0.456917	-2.227106
21	6	0	2.754863	-1.631779	-0.230587
22	1	0	-5.070462	1.479675	-0.703782
23	1	0	3.459879	-2.421465	0.031534
24	6	0	-4.383285	-0.433482	0.140308
25	6	0	2.733988	-1.330600	-1.740424
26	7	0	1.386048	-2.063411	0.007278
27	8	0	1.382912	-0.926854	-1.966667
28	1	0	2.980028	-2.199698	-2.354778
29	1	0	4.297874	3.246582	1.333841
30	1	0	4.456791	2.505331	-1.027657
31	6	0	-5.225963	0.407066	-0.837517
32	6	0	0.505113	-1.584166	-1.081963
33	1	0	-0.054325	2.877009	2.070452
34	6	0	-1.961180	4.030349	0.436161
35	1	0	0.054186	-2.460144	-1.560797
36	1	0	1.187810	2.658612	0.828790
37	1	0	-4.571865	1.014702	1.780407
38	1	0	2.590445	-0.533604	2.449549
39	1	0	3.369987	1.745786	3.059008
40	1	0	-4.170139	-0.628460	2.301122
41	1	0	-5.813258	-0.244316	1.761579
42	6	0	0.307936	3.243880	1.105394
43	1	0	0.621462	4.285476	1.228739
44	1	0	-2.755157	4.021037	-0.316032
45	1	0	-2.389508	3.719665	1.393375
46	1	0	-1.614348	5.061440	0.543394
47	1	0	-0.962775	3.577313	-2.100455
48	1	0	0.079887	4.701315	-1.209776
49	1	0	0.663649	3.079697	-1.606260
50	6	0	-0.217336	3.652229	-1.303658
51	6	0	-0.784925	3.139052	0.030371
52	6	0	-4.795247	-1.898695	-0.058975
53	1	0	-4.569316	-2.253681	-1.064921
54	1	0	-4.301326	-2.563903	0.648766
55	1	0	-5.876294	-1.969229	0.097446
56	1	0	0.793571	1.018583	-0.657765
57	8	0	-2.175207	-2.375961	-0.549345
58	6	0	1.995853	-3.613112	1.794822
59	1	0	2.612984	-4.343244	1.262994
60	1	0	2.653312	-2.850175	2.210761
61	1	0	1.464278	-4.123001	2.595453
62	8	0	-0.175015	-3.466600	0.810833
63	6	0	0.982868	-3.041351	0.840298

Structure 53a (M06-2X, Gas Phase)

Energy (Hartrees): =-1290.8846441
No imaginary frequencies

Standard orientation:

Center Number	Atomic Number	Atomic Type	Coordinates (Angstroms) X	Y	Z
1	6	0	-1.515615	1.200837	3.086169
2	6	0	-0.519301	0.365728	3.589828
3	6	0	-0.048850	-0.395905	-1.065106
4	1	0	3.167602	1.368688	0.031799
5	6	0	4.711066	-0.600324	-1.232481
6	6	0	-0.318028	-0.900260	3.050574
7	1	0	3.393422	-1.295697	1.902847
8	6	0	-0.048375	0.967099	-0.818355
9	1	0	5.088231	-1.367366	1.387149
10	6	0	-2.311704	0.783378	2.025399
11	6	0	1.143466	-1.136741	-0.941164
12	6	0	-2.108126	-0.482457	1.491567
13	6	0	1.109757	1.642101	-0.439044
14	6	0	-1.126527	-1.322856	2.002420
15	6	0	2.256532	0.875285	-0.279091
16	1	0	0.259544	-2.772087	-1.366694
17	6	0	2.317228	-0.506745	-0.501230
18	6	0	-1.086268	-2.642381	1.274457
19	1	0	-0.089684	-2.878145	0.892820
20	1	0	-1.387741	-3.460902	1.933430
21	6	0	-2.804418	-1.106509	0.300408

22	1	0	4.228656	0.174175	1.389879
23	1	0	-3.879267	-1.212979	0.449401
24	6	0	3.678648	-1.188190	-0.253351
25	6	0	-2.089881	-2.485305	0.112256
26	7	0	-2.530648	-0.432490	-0.961853
27	8	0	-1.470808	-2.406474	-1.172718
28	1	0	-2.791889	-3.316051	0.059600
29	1	0	0.105433	0.708158	4.406272
30	1	0	0.462247	-1.545566	3.438453
31	6	0	4.121658	-0.893789	1.193239
32	6	0	-1.348294	-1.032214	-1.552068
33	1	0	1.332548	3.613227	-2.343035
34	6	0	2.446240	3.717934	0.159698
35	1	0	-1.427333	-0.978437	-2.638557
36	1	0	-0.358859	3.498448	-1.849747
37	1	0	4.817373	0.480218	-1.115897
38	1	0	-3.065665	1.448338	1.617096
39	1	0	-1.658210	2.186864	3.512553
40	1	0	4.414190	-0.801897	-2.264566
41	1	0	5.690112	-1.057246	-1.061089
42	6	0	0.630727	3.837251	-1.535932
43	1	0	0.591853	4.922429	-1.402973
44	1	0	2.802528	3.293091	1.102058
45	1	0	3.193435	3.523583	-0.614210
46	1	0	2.372707	4.800613	0.288557
47	1	0	0.371263	3.013261	1.822926
48	1	0	0.047193	4.581204	1.048846
49	1	0	-0.930111	3.161866	0.641385
50	6	0	0.079327	3.499312	0.888556
51	6	0	1.075521	3.157409	-0.230203
52	6	0	3.704837	-2.714862	-0.420604
53	1	0	2.999497	-3.207189	0.249756
54	1	0	3.468656	-3.020252	-1.438313
55	1	0	4.712577	-3.063825	-0.177110
56	1	0	-0.980649	1.507060	-0.942846
57	8	0	1.169941	-2.464479	-1.248892
58	6	0	-4.738013	0.639434	-1.102666
59	1	0	-5.364064	-0.256483	-1.109235
60	1	0	-4.655400	0.986720	-0.072526
61	1	0	-5.209155	1.404052	-1.714597
62	8	0	-3.067596	0.738906	-2.810366
63	6	0	-3.388073	0.330423	-1.715718

Structure 53a (M06-2X, Pyridine)

Energy (Hartrees): =-1290.9172936
No imaginary frequencies

Standard orientation:

Center Number	Atomic Number	Atomic Type	Coordinates (Angstroms) X	Y	Z
1	6	0	-1.767824	1.321355	3.005308
2	6	0	-0.814284	0.505354	3.616338
3	6	0	0.006568	-0.433744	-1.021146
4	1	0	3.218017	1.351540	0.070757
5	6	0	4.792282	-0.592195	-1.103412
6	6	0	-0.572096	-0.780623	3.140544
7	1	0	3.392357	-1.358332	1.979150
8	6	0	0.003668	0.934039	-0.797833
9	1	0	5.099500	-1.402673	1.506448
10	6	0	-2.481612	0.864099	1.901172
11	6	0	1.197964	-1.174328	-0.866918
12	6	0	-2.235575	-0.420349	1.430326
13	6	0	1.161526	1.616234	-0.419611
14	6	0	-1.296614	-1.241992	2.046048
15	6	0	2.309668	0.853287	-0.241124
16	1	0	0.292822	-2.813847	-1.197619
17	6	0	2.372311	-0.534592	-0.440679
18	6	0	-1.211425	-2.588850	1.373265
19	1	0	-0.190161	-2.846627	1.080946
20	1	0	-1.567037	-3.379234	2.038620
21	6	0	-2.848463	-1.092389	0.219506
22	1	0	4.222446	0.130790	1.507725
23	1	0	-3.930234	-1.191336	0.294487
24	6	0	3.734645	-1.207894	-0.169449
25	6	0	-2.128816	-2.474714	0.139592
26	7	0	-2.481510	-0.463831	-1.046790
27	8	0	-1.412279	-2.445439	-1.103022
28	1	0	-2.828015	-3.304673	0.068690
29	1	0	-0.256047	0.877973	4.467683
30	1	0	0.172468	-1.411468	3.614391
31	6	0	4.133015	-0.936232	1.293533
32	6	0	-1.272374	-1.091137	-1.538336
33	1	0	1.446102	3.539808	-2.365273

34	6	0	2.489576	3.700608	0.172181
35	1	0	-1.282597	-1.092840	-2.629088
36	1	0	-0.260796	3.449415	-1.908550
37	1	0	4.892914	0.485846	-0.960982
38	1	0	-3.207328	1.506514	1.413289
39	1	0	-1.942911	2.321587	3.384841
40	1	0	4.535195	-0.774623	-2.150616
41	1	0	5.767885	-1.048077	-0.909285
42	6	0	0.725768	3.786560	-1.580174
43	1	0	0.693858	4.875330	-1.475195
44	1	0	2.820318	3.290085	1.130417
45	1	0	3.259896	3.494670	-0.576170
46	1	0	2.413253	4.785519	0.282005
47	1	0	0.356321	3.058809	1.788884
48	1	0	0.072838	4.601188	0.959370
49	1	0	-0.906482	3.184075	0.555876
50	6	0	0.098781	3.514821	0.829137
51	6	0	1.128898	3.137294	-0.245878
52	6	0	3.782911	-2.729005	-0.371120
53	1	0	3.082572	-3.250472	0.281948
54	1	0	3.562723	-3.014438	-1.399488
55	1	0	4.794829	-3.067904	-0.129166
56	1	0	-0.926375	1.475168	-0.935890
57	8	0	1.212948	-2.507970	-1.142462
58	6	0	-4.653634	0.629611	-1.349255
59	1	0	-5.282018	-0.264860	-1.335684
60	1	0	-4.614345	1.030120	-0.335720
61	1	0	-5.095029	1.363885	-2.019068
62	8	0	-2.885474	0.648463	-2.963724
63	6	0	-3.281183	0.282362	-1.871436

Structure 57 (M06-2X, Gas Phase)

Energy (Hartrees): =-1290.8902931
No imaginary frequencies

Standard orientation:

Center Number	Atomic Number	Atomic Type	Coordinates (Angstroms) X	Y	Z
1	6	0	-3.447881	2.135787	-0.991761
2	6	0	3.650354	-2.748390	-1.987467
3	6	0	2.057696	3.870934	0.575479
4	6	0	2.920728	-0.961220	-0.375048
5	6	0	-6.343201	-0.389887	-1.151406
6	6	0	-5.905962	-1.253717	-0.150145
7	6	0	-0.835772	-0.237733	-0.468566
8	6	0	1.167778	1.200785	0.003179
9	6	0	3.376864	0.323559	-0.108156
10	6	0	5.349920	-1.685066	-0.530295
11	6	0	-3.997660	0.144288	0.215987
12	6	0	-4.424795	1.000525	-0.796384
13	6	0	-4.724500	-0.994237	0.538439
14	6	0	3.975224	2.728595	1.673316
15	6	0	0.656532	-0.069941	-0.264331
16	6	0	3.674233	-3.197687	0.496492
17	6	0	1.522405	-1.151246	-0.431484
18	6	0	-2.685649	0.636302	0.804832
19	6	0	3.135272	2.798379	0.387045
20	6	0	2.530796	1.422442	0.097672
21	6	0	-2.205128	1.657337	-0.239836
22	7	0	-1.602519	-0.333145	0.796898
23	6	0	3.884706	-2.133501	-0.595723
24	6	0	-5.601590	0.739719	-1.485500
25	6	0	4.035269	3.220622	-0.786294
26	1	0	-5.931665	1.397526	-2.281703
27	1	0	-7.260876	-0.607959	-1.684439
28	1	0	-6.482926	-2.139348	0.087006
29	1	0	-4.379265	-1.686915	1.297753
30	1	0	0.471947	2.022623	0.098611
31	1	0	4.444614	0.490062	-0.053133
32	1	0	0.307020	-2.545067	-0.026744
33	1	0	4.388597	-4.014365	0.356119
34	1	0	-2.808592	1.082109	1.793841
35	1	0	5.609380	-1.280509	0.451734
36	1	0	-1.620264	2.471435	0.198733
37	1	0	3.842216	-2.763929	1.486005
38	1	0	2.666970	-3.607829	0.461563
39	1	0	5.992990	-2.549019	-0.713761
40	1	0	-3.828908	3.065037	-0.557392
41	1	0	4.784793	2.001616	1.582711
42	1	0	-1.031234	-1.118487	-1.085445
43	8	0	-1.404942	0.881797	-1.120855
44	1	0	1.398211	3.630537	1.413873

45	1	0	4.470473	4.206088	-0.595793
46	1	0	4.854075	2.513982	-0.935935
47	1	0	5.577540	-0.933072	-1.290718
48	1	0	4.420648	3.704500	1.888133
49	1	0	2.639058	-3.138633	-2.084588
50	1	0	3.812768	-1.997534	-2.765621
51	1	0	4.358389	-3.566258	-2.150849
52	8	0	1.022263	-2.394487	-0.676764
53	1	0	3.353955	2.436675	2.523687
54	1	0	1.449278	3.989122	-0.325086
55	1	0	2.532352	4.832020	0.787491
56	1	0	3.459121	3.269050	-1.713501
57	1	0	-3.208032	2.321207	-2.039869
58	6	0	-1.458588	-1.392665	1.622584
59	6	0	-2.197427	-1.381838	2.941623
60	1	0	-2.453420	-2.410313	3.190531
61	1	0	-3.090172	-0.760403	2.956137
62	1	0	-1.503254	-1.014915	3.701512
63	8	0	-0.701810	-2.319799	1.358286

Structure 57 (M06-2X, Pyridine)

Energy (Hartrees): =-1290.9234652
No imaginary frequencies

Standard orientation:

Center Number	Atomic Number	Atomic Type	Coordinates (Angstroms) X	Y	Z
1	6	0	-3.454634	2.138636	-0.975166
2	6	0	3.640684	-2.767727	-1.982147
3	6	0	2.053409	3.878019	0.541459
4	6	0	2.918339	-0.959864	-0.386232
5	6	0	-6.342687	-0.395912	-1.168192
6	6	0	-5.910329	-1.262325	-0.165417
7	6	0	-0.846624	-0.244420	-0.465211
8	6	0	1.161106	1.202095	-0.003543
9	6	0	3.375344	0.328393	-0.127497
10	6	0	5.350163	-1.676273	-0.560754
11	6	0	-4.001124	0.134527	0.212822
12	6	0	-4.425919	0.996392	-0.798026
13	6	0	-4.731588	-1.004115	0.531304
14	6	0	3.941065	2.728677	1.684388
15	6	0	0.650188	-0.071004	-0.267059
16	6	0	3.693681	-3.181423	0.504886
17	6	0	1.519189	-1.152375	-0.432534
18	6	0	-2.693369	0.630779	0.810712
19	6	0	3.130507	2.801436	0.380285
20	6	0	2.526675	1.425748	0.082915
21	6	0	-2.213179	1.654928	-0.228799
22	7	0	-1.608485	-0.342789	0.799753
23	6	0	3.886334	-2.130905	-0.602999
24	6	0	-5.600513	0.737512	-1.494157
25	6	0	4.064149	3.216816	-0.768277
26	1	0	-5.929511	1.400999	-2.286757
27	1	0	-7.258605	-0.612262	-1.706342
28	1	0	-6.490210	-2.147460	0.069434
29	1	0	-4.395289	-1.693248	1.298846
30	1	0	0.465018	2.022430	0.106964
31	1	0	4.443018	0.498576	-0.077719
32	1	0	0.289843	-2.513210	0.003825
33	1	0	4.406069	-4.000390	0.364710
34	1	0	-2.819777	1.068579	1.801141
35	1	0	5.623398	-1.261779	0.413571
36	1	0	-1.621548	2.462852	0.207333
37	1	0	3.877029	-2.738532	1.488343
38	1	0	2.685826	-3.594240	0.493512
39	1	0	5.993490	-2.540296	-0.746697
40	1	0	-3.838594	3.054715	-0.516828
41	1	0	4.746767	1.993748	1.614030
42	1	0	-1.040854	-1.126951	-1.080607
43	8	0	-1.415347	0.870580	-1.119986
44	1	0	1.379036	3.650908	1.371929
45	1	0	4.496021	4.201368	-0.564471
46	1	0	4.887842	2.509955	-0.893719
47	1	0	5.566533	-0.928304	-1.328748
48	1	0	4.390340	3.702315	1.903043
49	1	0	2.630278	-3.165691	-2.066287
50	1	0	3.791983	-2.031076	-2.776984
51	1	0	4.349994	-3.585683	-2.141791
52	8	0	1.013670	-2.397871	-0.652187
53	1	0	3.300254	2.450523	2.526026
54	1	0	1.456578	3.991108	-0.367938
55	1	0	2.529973	4.838983	0.751877
56	1	0	3.517155	3.272258	-1.713724

57	1	0	-3.224729	2.352774	-2.020259
58	6	0	-1.439453	-1.381216	1.640933
59	6	0	-2.172645	-1.376117	2.955656
60	1	0	-2.450708	-2.401229	3.197780
61	1	0	-3.052398	-0.737790	2.980553
62	1	0	-1.469852	-1.027565	3.717641
63	8	0	-0.654273	-2.296277	1.380166
